# Patient-Specific Implant Design: Current Evidence for Artificial Intelligence, Imaging and Computational Planning—A Systematic Review of the Biomedical-Indexed Literature

**DOI:** 10.3390/bioengineering13101149

**Published:** 2026-10-01

**Authors:** Adrian Barbilian, Marius Moga, Mark Edward Pogarasteanu, Vlad Tau, Alex Lucaci, Constantin-Andreas Gorgan, Alexandru Diaconu, Robert Daniel Dobrotă, Razvan Adam, Cristian Stoica

**Affiliations:** 1 Faculty of Medicine, University of Medicine and Pharmacy “Carol Davila”, 020021 Bucharest, Romania; adrian.barbilian@umfcd.ro (A.B.);; 2 Eden Health SRL, 012256 Bucharest, Romaniaalex.lucaci92@gmail.com (A.L.); 3 “Prof. Dr. Al. Obregia” Psychiatry Clinical Hospital, 041914 Bucharest, Romania; 4 CG Tech Labs SRL, 011754 Bucharest, Romania; 5 Humans Nexus Ltd., THE CORE, Triq Il-Wied Ta’ L-Imsida, MSD 9021 Msida, Malta; 6 Orthopedic Department, Clinical University Emergency Hospital Elias, 011461 Bucharest, Romania; adamrazvan@yahoo.com

**Keywords:** patient-specific implant, custom implant, additive manufacturing, artificial intelligence, deep learning, finite element analysis, topology optimisation, virtual surgical planning, systematic review

## Abstract

Background and objectives: Patient-specific skeletal implants are designed through a pipeline of imaging, computational planning and, increasingly, artificial intelligence. Each pillar has its own validation literature, but no review has asked what evidence supports each one in implant design itself. We mapped that evidence from the biomedical-indexed literature in this paper. Methods: A systematic review was conducted under a protocol committed before retrieval (PRISMA 2020, PRISMA-S, SWiM). MEDLINE, Europe PMC, Crossref and OpenAlex were searched on 2 and 3 September 2026 for primary studies from 2015 onwards applying artificial intelligence, an evaluated imaging pipeline or computational planning to an implant whose geometry derives from the individual patient’s anatomy. Engineering indexes were unavailable, so this review maps biomedical-indexed literature, and a bounded probe was used to estimate what that omits. Two artificial intelligence screeners from different vendors screened every record; every clinical study was appraised twice, independently, by assessors from different vendors. Claims about validation quality are reported on the studies read in full. All screening, charting and appraisals were machine-performed and no human-verified subsample exists. Results: Of 5059 records, 639 studies were included: 373 technical studies, 179 clinical series, 75 comparative studies and 12 randomised trials. Computational planning was applied in 601, an evaluated imaging step in 219 and artificial intelligence in 36. Randomised evidence came from 12 small trials, 10 of them craniomaxillofacial, which evaluated the delivered implant rather than the planning method; virtual surgical planning was used in 11 of them. When restricting to the four studies that measure deviation from the virtual plan, with smaller meaning better, patient-specific implants deviated less from the plan (standardised mean difference −1.21, 95% CI −1.69 to −0.74). Revision or removal was less frequent (3 of 135 against 25 of 149; risk ratio 0.28, 95% CI 0.11 to 0.72); operative time, complications and infection gave intervals compatible with a difference either way. No randomised trial was at low risk of bias, and 69 of 75 comparative studies were at serious risk, mostly of unadjusted confounding, so all estimates are of very low certainty. Among technical studies read in full, independent validation material was used in fewer than half, and files were shared in about one in fourteen. Of 36 artificial intelligence studies, 4 were evaluated on independent data, 2 were used for a real patient, none were clinically comparative, and 8 of 17 cranial studies relied on AutoImplant family benchmarks. Conclusions: The design pipeline is unequally validated: planning has clinical evidence concentrated in one region, computational methods are widely applied but rarely validated independently or shared, and artificial intelligence has benchmark evidence for cranial defect completion and little else. Outcome studies of patient-specific implants should be read as evidence about whole pipelines, and future studies should evaluate the components.

## 1. Introduction

Implants whose geometry is derived from the individual patient’s imaging are now routine in cranioplasty, orbital and midface reconstruction, mandibular and maxillary reconstruction, pelvic and limb-salvage oncology and complex revision arthroplasty, and are spreading into primary joint replacement, spinal reconstruction and foot and ankle surgery [1,2,3,4]. The clinical literature is large and has been reviewed repeatedly by anatomical region: cranioplasty [5], orbital reconstruction [6], orthognathic surgery [7], maxillary reconstruction [8], mandibular reconstruction [9,10], the pelvis [3], the spine [1,11] and segmental long-bone defects [12]. Those reviews ask whether a patient-specific implant performs at least as well as a conventional one. They rarely ask how the implant was designed and whether the methods that produced it have themselves been validated.

The design pipeline has three technical pillars. The first is imaging: acquisition, segmentation and surface modelling produce the anatomy the implant is fitted to, and errors here propagate into every downstream step. The second is computational planning: virtual surgical planning positions the resection and the implant, statistical shape modelling or mirroring restores the missing anatomy, parametric or automated design generates the geometry, topology or lattice optimisation tunes stiffness and mass, and finite element analysis is used to verify the result before manufacture. The third and most recent is artificial intelligence, applied to segmentation, to defect and shape completion, to design generation and to prediction of design parameters or outcomes; the AutoImplant challenges made automatic cranial implant design a benchmark task for deep learning [13], and generative approaches have followed [14]. Each pillar has an engineering validation literature of its own, with reporting and credibility frameworks such as the risk-based credibility framework for computational models [15], the TRIPOD + AI and CLAIM guidelines for prediction models and imaging AI [16,17], and reviews of statistical shape model accuracy [18]. Clinical papers on patient-specific implants tend to invoke these methods as though their accuracy in the implant-design setting were settled.

No review, to our knowledge, has set out the evidence for each pillar in the specific setting of patient-specific skeletal implant design: which methods have been applied, for which regions and implant types, at which tier of evidence (in silico, bench, cadaver or phantom, retrospective imaging dataset, clinical series, comparative clinical study, randomised trial), against which comparators, with what outcomes, and with what risk of bias or technical credibility. A registry search run for this review found many region-specific registrations on patient-specific implant outcomes and a few on single methods, but none covering the three pillars across skeletal regions with evidence tiers. This review fills that gap for the literature indexed in biomedical databases. Its questions are what methods within each pillar have been applied and evaluated, at what tier and against what comparator, what outcomes they report, whether artificial intelligence methods have been evaluated on independent data or in clinical use, how credible the clinical and technical studies are, and where the evidence stops. The boundary matters and is stated at the outset: finite element, optimisation and machine learning work on implant design is published partly in engineering venues that the biomedical indexes do not cover, and the engineering indexes were not available for this review. What follows is therefore a map of what the biomedical literature contains, not a census of the field, and Section 3.8 measures how much sits outside it.

## 2. Materials and Methods

### 2.1. Design and Registration

This is a systematic review with a structured narrative synthesis and an evidence map, reported per PRISMA 2020 [19], with the search reported per PRISMA-S [20] and the synthesis per the Synthesis Without Meta-analysis guideline [21]. The protocol was written and committed to a version-controlled repository on 2 September 2026 before any record was retrieved or screened; eight PubMed hit-count queries, returning counts only, were run before the protocol to size the concept blocks and are archived with it. Every subsequent change is recorded in a dated amendments table in the protocol (Text S2). A PROSPERO record was prepared from the protocol but was not submitted, so the review was not prospectively registered.

### 2.2. Eligibility Criteria

We included primary studies published from 1 January 2015 to the search date, in any language, concerning a skeletal implant, that is a device implanted in or onto bone in orthopaedic, spinal, cranial, craniomaxillofacial or jaw reconstruction, including joint replacement components, tumour endoprostheses, cranioplasty plates, orbital and midface plates, mandibular and maxillary reconstruction implants, spinal cages and vertebral body replacements, and patient-specific subperiosteal or reconstruction jaw implants. Two concept criteria had to hold. First, the implant, or a load-bearing part of it, had to be patient-specific: its geometry derived from the individual patient’s imaging or anatomy. Off-the-shelf implants, including 3D-printed but non-individualised designs, did not qualify. Second, at least one of three pillars had to be applied to the implant’s design or planning and evaluated: artificial intelligence (machine or deep learning at any step of the pipeline); an evaluated imaging pipeline (segmentation accuracy for the design, the effect of imaging protocol or modality, registration or mirroring accuracy, statistical or template shape modelling for the design); or computational planning (finite element or other biomechanical simulation of the specific implant, topology, lattice or shape optimisation, parametric or automated design, or virtual surgical planning in which the implant itself is planned). Studies had to report at least one measured quantity about the method or the implant (geometric deviation or fit, mechanical response, design or production time, or a clinical outcome).

Eligible study types were in silico studies, bench or mechanical testing, cadaveric or phantom studies, retrospective imaging-dataset studies, clinical series of at least five patients, comparative non-randomised studies and randomised trials. Preprints carrying a full report were eligible and, where a journal version was also in the corpus, the preprint was treated as a duplicate of it (amendment A3); anatomy-derived jaw implants were eligible, but implants customised only by parameter choice without anatomy-derived geometry were not (amendment A2). We excluded case reports and series of fewer than five patients (counted in the flow, not charted), reviews, editorials, letters, protocols and conference abstracts, non-human studies, studies of patient-specific instrumentation or guides without a patient-specific implant, standard endosseous dental implants and prosthodontic restorations, external prostheses and orthoses, non-skeletal implants, and tissue-engineering studies without an implant designed for a specific patient.

### 2.3. Information Sources and Search

MEDLINE via PubMed (E-utilities), Europe PMC (title and abstract fields) and Crossref (a bounded relevance sweep, disclosed as supplementary rather than as a full-retrieval source) were searched by script on 2 September 2026, with raw responses archived. OpenAlex was in the protocol but could not be searched on 2 September: its API now meters a daily budget, and every request was refused (amendment A1). The prepared OpenAlex queries were run on 3 September 2026 once the budget had reset as a documented supplementary run (amendment A9); records not already in the corpus entered the same rule layer, dual triage and charting, and their counts are folded into the database arm of the flow and listed separately in Text S3. The main search was the intersection of four concept blocks, individualisation (patient-specific, custom, personalised, 3D-printed and synonyms), device, skeletal region and method (the union of the three pillars’ vocabularies), limited to publication dates from 2015 and with no outcome, language or study-type filter, so as to protect recall; a supplementary strategy used explicit patient-specific-implant phrases. Full strings, hit counts and retrieval counts are in Text S3. PROSPERO and OSF Registries were searched for competing reviews, and published systematic and scoping reviews on the topic were identified to position this review (searches/registry-and-prior-reviews.md in the repository). Reference lists of systematic and scoping reviews identified by the search or the registry search were chased backward through the NCBI reference links because the Europe PMC reference service was under maintenance on the run date; reference lists are indexed only for articles deposited in PubMed Central, so this chase is partial and is reported as such. Embase, Scopus, Web of Science, IEEE Xplore and CINAHL were unavailable to the project, a limitation declared in the protocol; the Crossref sweep is supplementary discovery of unknown recall, not a substitute for them. Because that omission bears directly on what this review can claim, its size was measured rather than asserted. Two checks were run at revision and are reported in Section 3.8. The first asks what one broader index already contributed by counting how many included studies entered only through the OpenAlex arm and were invisible to the three biomedical sources. The second runs the review’s own concept blocks against engineering-type sources in OpenAlex, identifies the records absent from this review’s corpus, screens a random sample of them against the eligibility criteria, and turns the observed eligible proportion into an estimate, with a confidence interval, of how many eligible studies the engineering route would add and of which kind they would be.

### 2.4. Selection

Selection had three layers, each with published per-record reasons. A rule layer excluded records by explicit, reproducible text rules: no individualisation term, no device term, a non-human indexing signal without an accompanying human signal (the exception, an implementation detail not stated in the committed rule, retained 138 records carrying both signals; amendment A4), a review, editorial, letter, comment or protocol publication type or a review-type title (these were retained separately for citation chasing), or the Case Reports publication type. A seeded random sample of 25 rule-excluded records was re-screened against the full criteria to bound the false-exclusion rate. The remaining records were then classified independently by two AI screeners from different vendors, a GPT-based agent run through the Codex command-line interface and a Claude model run as Claude Code subagents, each reading the same written instruction file (Text S4) and blind to the other, as to include, exclude or uncertain; a record was retained unless both excluded it (recall-union). Outputs were reconciled against the input identifier set so that no record could be dropped silently, and the full cross-tabulation, raw agreement and Cohen’s kappa are reported. Records were deduplicated by identifier at retrieval; both independent reviews of the first draft found that the same article could survive under a second identifier (a DOI-keyed copy beside the PubMed copy) or as a preprint beside its journal version, and such copies were excluded at assessment as duplicate reports, with the copy carrying full text then the PubMed copy retained (amendments A3 and A6). Retained records, together with citation-chase candidates screened in the same way, were assessed against the criteria at full record by an AI charter from open full text where it could be retrieved (PubMed Central, Europe PMC, Unpaywall) and otherwise from the abstract, with the text source recorded per record; a record with neither an abstract nor retrievable text was held as unresolved whatever the charter returned. All screening and assessment were AI-performed under the written instructions.

### 2.5. Data Charting

One row per report was charted by the AI charter from the written charting instructions (Text S4): identifiers, year, country, anatomical region, implant type, material and manufacturing route; the pillars applied, with method detail for each (artificial intelligence task and model family; imaging modality and method; computational method); the highest evidence tier reached; numbers of patients treated and of analysed units (cases, images, specimens, models or designs); comparator and its type; headline results by outcome domain (geometric accuracy or fit, mechanical, design or production time, clinical) with units; follow-up; for artificial intelligence studies, whether evaluation used data independent of development, whether any clinical or prospective evaluation exists, and whether code or data are public; and a verbatim sentence supporting the tier assignment. Funding source, conflict-of-interest declarations and industry authorship, prespecified but omitted from the charting form, were charted in a separate pass from the records with full text (amendment A7). A field the text did not settle was charted as unclear, never as absent. Three targeted re-adjudication passes, each from a written instruction file (Text S4), re-examined the records first charted as unclear, the jaw and dental records against amendment A2, and the evaluation-data source of every artificial intelligence study; their outputs were applied as corrections. A seeded random sample of 60 assessed records was charted a second time, independently, by a Claude model from the same instructions, and agreement on the key fields is reported; disagreements were adjudicated by the orchestrating system against the source text, and the adjudicated values were recorded as corrections separate from the charters’ output. Counts in this manuscript are computed by script from the charting file. A verification script checks that every numeric literal in the text and tables is a value the charting data produce; it bounds numerical drift but does not check that a value sits in the right sentence, which the independent reviews and the authors’ reading address separately.

### 2.6. Appraisal

Two different questions run through this review, and they need different evidence, so the target of every appraisal and every pooled estimate is stated before the tools are named. The first question is whether a delivered patient-specific implant performs better than a conventional one. Randomised and comparative clinical studies answer that, and RoB 2, ROBINS-I and GRADE are the right instruments for it. The second question, the one this review was written to ask, is whether the imaging, planning and artificial intelligence methods that produced the implant have themselves been validated. No clinical outcome study answers that, because every arm of such a study receives a whole pipeline rather than one component of it. Risk-of-bias appraisal and certainty rating in this review therefore describe the delivered-implant comparison and nothing more; the validation status of the design methods is assessed separately against the credibility items below, and the two are never combined into a single judgement. Clinical studies were appraised with the tool matching their design: RoB 2 for randomised trials [22], ROBINS-I for comparative non-randomised studies [23] and the JBI checklist for case series for non-comparative clinical series [24]. Technical studies (in silico, bench, cadaver or phantom, imaging dataset, artificial intelligence development) were charted against a six-item credibility set derived from the risk-based credibility framework for computational models [15] and from TRIPOD + AI and CLAIM [16,17]: validation data or specimens independent of those used to develop or tune the method; quantitative comparison against a physical experiment or a ground truth; uncertainty or sensitivity analysis; reproducibly specified inputs (imaging protocol, material properties, loads and boundary conditions, or training data description); availability of code, model or design files; and evaluation on more than one anatomy or specimen. Items are reported as counts, not as a score, because the six are not interchangeable and an aggregate would hide which one failed. Each item is coded ‘yes’ where the text establishes it, ‘no’ where the text establishes its absence, and ‘unclear’ where the text settles neither; silence is ‘unclear’ and never ‘no’, which is what allows the abstract-only route to be separated from a genuine deficiency in Section 3.5. Two further coders, from different vendors, applied the same six items independently to a seeded random sample of 90 technical studies so that the reproducibility of the set could be estimated rather than assumed; per-item agreement is reported in Section 3.5.

The six items were chosen to cover what is common to simulation, imaging and learned models, and they do not cover what TRIPOD + AI, CLAIM and PROBAST + AI ask specifically of prediction models. For the 36 artificial intelligence studies, a second, prediction-specific set was therefore charted separately: the named benchmark or private dataset the model was evaluated on, the risk that evaluation data were not independent of development data, whether calibration was reported, which discrimination or error metrics were reported, whether missing data handling was described, whether the sample size was justified, and whether validation was external in the TRIPOD + AI sense. Calibration is recorded as not applicable where a model outputs a geometry rather than a probability, because recording a false deficiency would be as misleading as missing a real one. PROBAST + AI itself was not applied: it appraises risk of bias in a single prediction model against an intended clinical use, and most of these studies report a reconstruction benchmark with no stated clinical use to appraise against. That is a limitation of what can be claimed here, not a judgement that the tool is unsuitable. Every clinical study was appraised twice. The first assessment was made by a GPT-based assessor and the second, independently and from the same written instruction file (Text S4), by a Claude-based assessor from a different vendor, which was blinded to the first assessor’s judgements and was instructed not to read them. Agreement on the overall judgement is reported per tool in Section 3.7, together with the full distribution of each assessor’s judgements, and every disagreement is listed per study in Table S4. The first draft of this review carried a single appraisal, and the duplicate assessment was added in response to peer review. Certainty of evidence was rated per outcome for the clinical comparisons that could be pooled or narratively synthesised (geometric accuracy, operative time, complications), using the GRADE domains of risk of bias, inconsistency, indirectness, imprecision and publication bias [25], starting from low certainty for the non-randomised body of evidence and from high for randomised trials, with each downgrade named (Section 3.7).

### 2.7. Synthesis

The synthesis is structured and narrative, grouped by pillar and then by anatomical region, with an evidence map of the pillar by tier and tables of included studies. Within each group, studies are presented in order of tier (randomised, comparative, series, then technical tiers) and, among artificial intelligence studies, those with evaluation on independent data are described first; results are summarised as counts of studies per category and as the range of reported values in their own units, without transformation, and without vote counting on direction or statistical significance. Heterogeneity is explored descriptively by tier, region, comparator class and whether the record was charted from full text.

The two kinds of claim this review makes are supported by different evidence and are reported on different denominators. Whether a study exists, what it studied, in which region, at which tier and with which pillar can be read from an abstract, so the evidence map uses all included studies and says so. Whether a study validated its method independently, compared it against a physical experiment, specified its inputs reproducibly or shared its files usually cannot be read from an abstract, so every claim of that kind is reported primarily on the studies read in full, with the abstract-only studies given separately rather than pooled with them. For each credibility item we also report the share met among only those records where the item could be read at all, which separates a method a study did not use from a method its abstract did not mention. The same split is applied to the risk-of-bias judgements, which for abstract-only records rest on absence of information and are labelled as such. The protocol permitted a random-effects meta-analysis only if at least three comparative clinical studies reported the same outcome against the same class of comparator with extractable estimates. That condition was tested on a dedicated extraction of arm-level estimates from the comparative studies and trials with full text (amendment A8): where it held, a DerSimonian–Laird random-effects model gave a pooled mean difference (operative time, minutes) or standardised mean difference (geometric accuracy, because measures differ), with tau-squared, I-squared and a t-based 95% prediction interval, for the delivered patient-specific implant against its conventional comparator; such an estimate is evidence about the implant as delivered, not about one design method against another, and is labelled as such. Membership of each pool was taken from the charted comparator class and, for accuracy, from the measure (deviation of the delivered result from the preoperative plan). Sensitivity analyses were a Hartung–Knapp confidence interval, leave-one-out estimates and, where ten or more studies were pooled, an Egger regression test for small-study effects; with fewer studies, no small-study test was attempted. At revision, three further analyses were added. Each pool was examined within an anatomical region. For deviation from the plan, two adjudicators from different vendors read each pooled paper’s methods to establish what its accuracy measure actually compared the delivered result against and whether a smaller value meant a better result; a study entered the primary estimate only where both adjudicators found a virtual-plan reference and a smaller-is-better direction, and the quoted sentence behind each decision is in Text S6. For the binary outcomes, events and denominators in both arms, the absolute risk difference and the number needed to treat are reported beside the risk ratio, because a ratio alone cannot be interpreted without the underlying risk. No minimal important difference has been established for deviation from plan in this setting; a deviation of about 2 mm is often treated as clinically acceptable in craniomaxillofacial reconstruction, but that threshold is a convention rather than a validated one, so differences are described in the measured units, and no study is called clinically important on the strength of a pooled standardised difference. Where the condition failed, the disposition is reported. The first draft’s decision not to pool although the condition was met was a post hoc deviation and was withdrawn on independent review.

### 2.8. Use of Artificial Intelligence and Verification

The search code, the rule layer, both title–abstract screening passes, the full-record assessment and charting, the second-charter sample, the output pipeline, the figures, the manuscript drafting and two rounds of independent adversarial pre-review (four reports) were performed with large language model agents (Claude Fable 5.1 and Claude Opus 5, Anthropic, San Francisco, CA, USA; GPT-5.6-sol via the Codex CLI, OpenAI, San Francisco, CA, USA) on 2 and 3 September 2026, orchestrated by human authors under the committed protocol; a contemporaneous log of which system did which stage on which date is shown in Text S5. Counts, meta-analyses and figures were computed with scripts in Python 3.14 (Python Software Foundation, Wilmington, DE, USA) using NumPy 2.5, SciPy 1.18 and Matplotlib 3.11. A first second-screening pass run with a smaller Claude model was superseded the same day by a pass with Claude Opus 5 at the authors’ direction; the superseded outputs are archived and did not enter the reported flow. The authors reviewed the instruction files, the reconciliation logic, the adjudicated corrections and every section of this manuscript and are accountable for its content. No AI system is reported as an author.

## 3. Results

### 3.1. Study Selection

**Figure 1:**
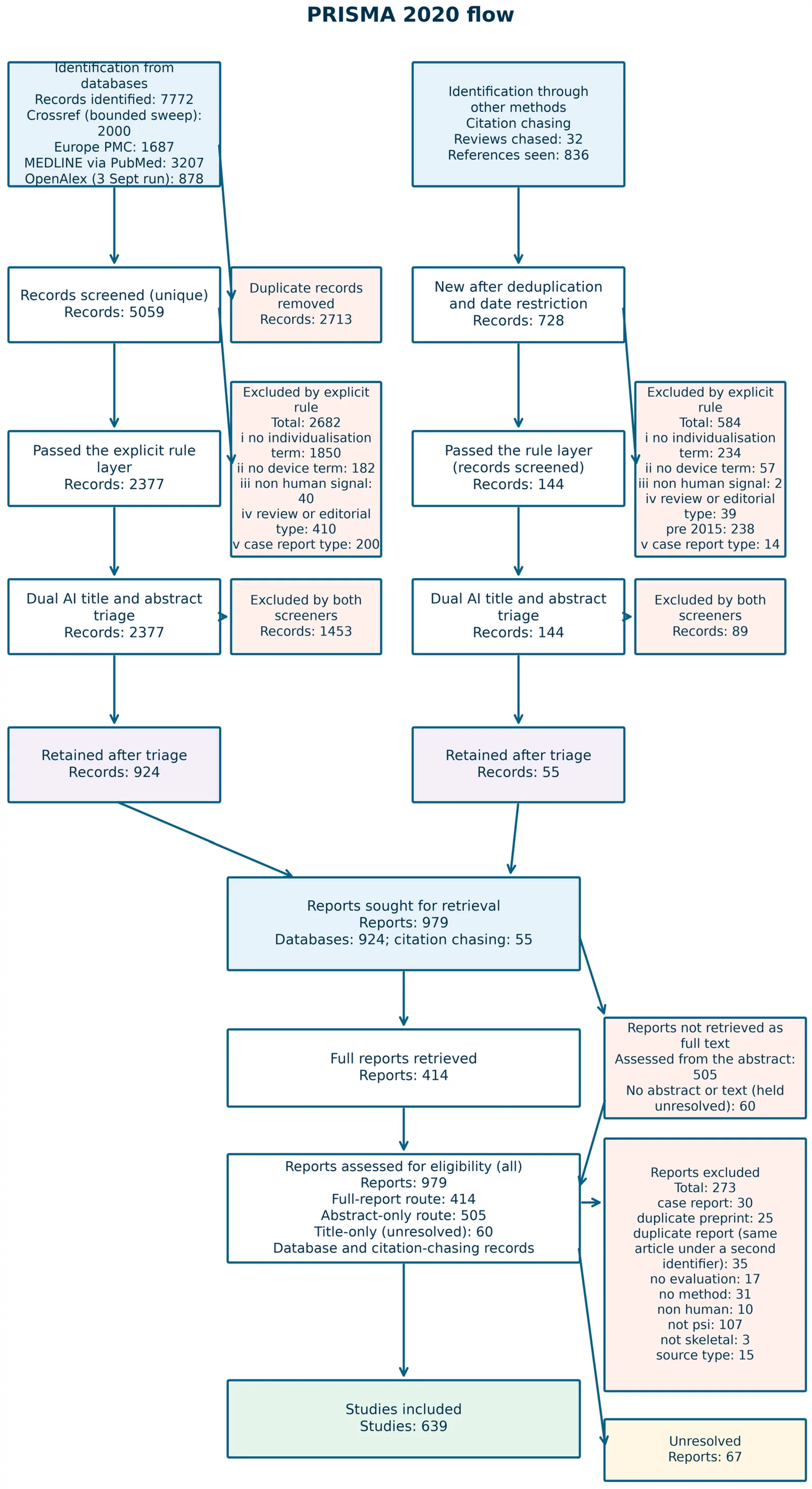
PRISMA 2020 flow. Parallel database and citation-chasing identification arms show deduplication, rule-based screening and dual independent AI triage before merging at report retrieval and eligibility assessment. Reports unavailable in full text were assessed from abstracts. Residual duplicate reports remain in both the assessed and excluded totals. Every junction is asserted against the machine-readable flow before rendering.

The database arm retrieved 7772 records: 3207 from MEDLINE via PubMed across the two strategies, 1687 from Europe PMC, 2000 from the bounded Crossref sweep and 878 from the OpenAlex queries run on 3 September (amendment A9). After deduplication by identifier, 5059 unique records remained (2713 duplicates removed). The rule layer excluded 2682: 1850 with no individualisation term, 182 with no device term, 40 with a non-human signal and no human signal, 410 review, editorial or protocol records (set aside for citation chasing) and 200 records indexed as case reports. The seeded audit of 25 rule-excluded records found no wrongly excluded records. The sample was drawn from the 2566 records the rule layer excluded in the main run of 2 September before the supplementary OpenAlex run of 3 September added a further 116 rule exclusions to give the 2682 total; the audit therefore bounds the main run only, and the supplementary exclusions are unaudited. Because 25 records is a small sample, the exact one-sided 97.5% upper bound on the false-exclusion proportion is 13.7%, which would permit up to 353 wrongly excluded records among the 2566 audited exclusions. The audit is therefore a check that the rule layer was not grossly wrong, not a demonstration that recall was adequate, and the reader should carry that uncertainty into every count in this review. The 2377 remaining records entered dual AI triage. On the main run, the two screeners agreed on 516 inclusions and 1300 exclusions, with raw three-class agreement 0.893 and Cohen’s kappa 0.781, and for inclusion against non-inclusion agreement 0.947 and kappa 0.866; on the OpenAlex increment they agreed on 95 inclusions, with three-class agreement 0.879 and kappa on inclusion 0.874 (full cross-tabulations in Text S1). Under the recall-union rule, 924 records were retained. The citation-chasing arm covered 32 systematic and scoping reviews whose 836 references were available through the NCBI links; 728 were new after deduplication, 144 passed the rule layer, 89 were excluded by both screeners, and 55 were retained (kappa on inclusion 0.592). Of the 979 reports sought, full text was obtained and verified against the record title for 414; 505 were assessed from the abstract alone, and 60 had neither an abstract nor retrievable text and were held as unresolved, whatever the charter returned (Figure 1). After full-record assessment, 273 reports were excluded: 107 with no patient-specific implant, 31 without an evaluated design method, 30 case reports or series below five patients, 17 without a measured quantity, 15 by source type, 10 non-human, 3 non-skeletal, and 60 copies of a report already assessed (25 preprint, repository or version copies of a journal article and 35 copies of the same article under a second identifier; amendments A3 and A6, with the grouping rerun over all assessed records after independent review found the first pass incomplete). Sixty-seven reports remain unresolved: 47 of the 60 records without text (the other 13 were second copies of assessed reports and are counted among the duplicates) and 20 reports with text whose eligibility, patient denominator or patient-specificity it did not settle; 639 studies were included. Two measurements describe how reliable the charting is. The first shows agreement between the two charters on the seeded 60-record sample: eligibility 87% (kappa 0.70), evidence tier 87% (kappa 0.84), region 90% (kappa 0.86) and pillars applied 83% (kappa 0.73). Agreement, however, only shows that two readers coincide, not that either is right, so at revision a reference standard was built for the same sample. Two adjudicators from different vendors read each record’s own text, decided each field themselves and recorded the sentence that decided it. Where both adjudicators reached the same value, that value is the reference standard, which was available for between 83% and 100% of records depending on the field. Against it, the primary charter was correct for eligibility in 89.3% of records, evidence tier 89.3%, region 87.5%, pillars 88.7%, whether artificial intelligence evaluation was independent 100%, whether it was clinical 98.3%, and availability of code or data 85.0%; the second charter was correct in 71.7% to 98.3% across the same fields. On 15 records, the two adjudicators did not agree, so no reference standard exists there, and those records are listed for resolution by the authors. This represents agreement between independent machine readings of the same text, and it is not a human gold standard; no human-verified subsample exists for this review, and Section 4.3 says what that leaves open. The 68 field-level disagreements across 33 records were adjudicated against the source text, and those adjudications, the format repairs, the re-charting of records whose eligibility or patient denominator was questioned, the re-assessment of the 92 records first charted as unclear, the dental-boundary and AI-validation re-adjudications and the duplicate exclusions are all recorded as corrections separate from the charters’ output (Text S1). Of the included studies, 295 were charted from verified full text and 344 from the abstract only (clinical tiers 119 and 147; technical tiers 176 and 197); 16 are preprints. An audit of every cached full text, prompted by the second assessor noticing one, found 14 retrievals that had returned a repository or publisher landing page rather than the article. Such a page reprints the title and therefore passes a title-match guard while containing none of the paper. Those records were reclassified as abstract-only, which is what they always were, and two of them, having no abstract either, moved to unresolved; that is why 639 studies are included here where the submitted version reported 641.

### 3.2. Evidence Map

**Figure 2:**
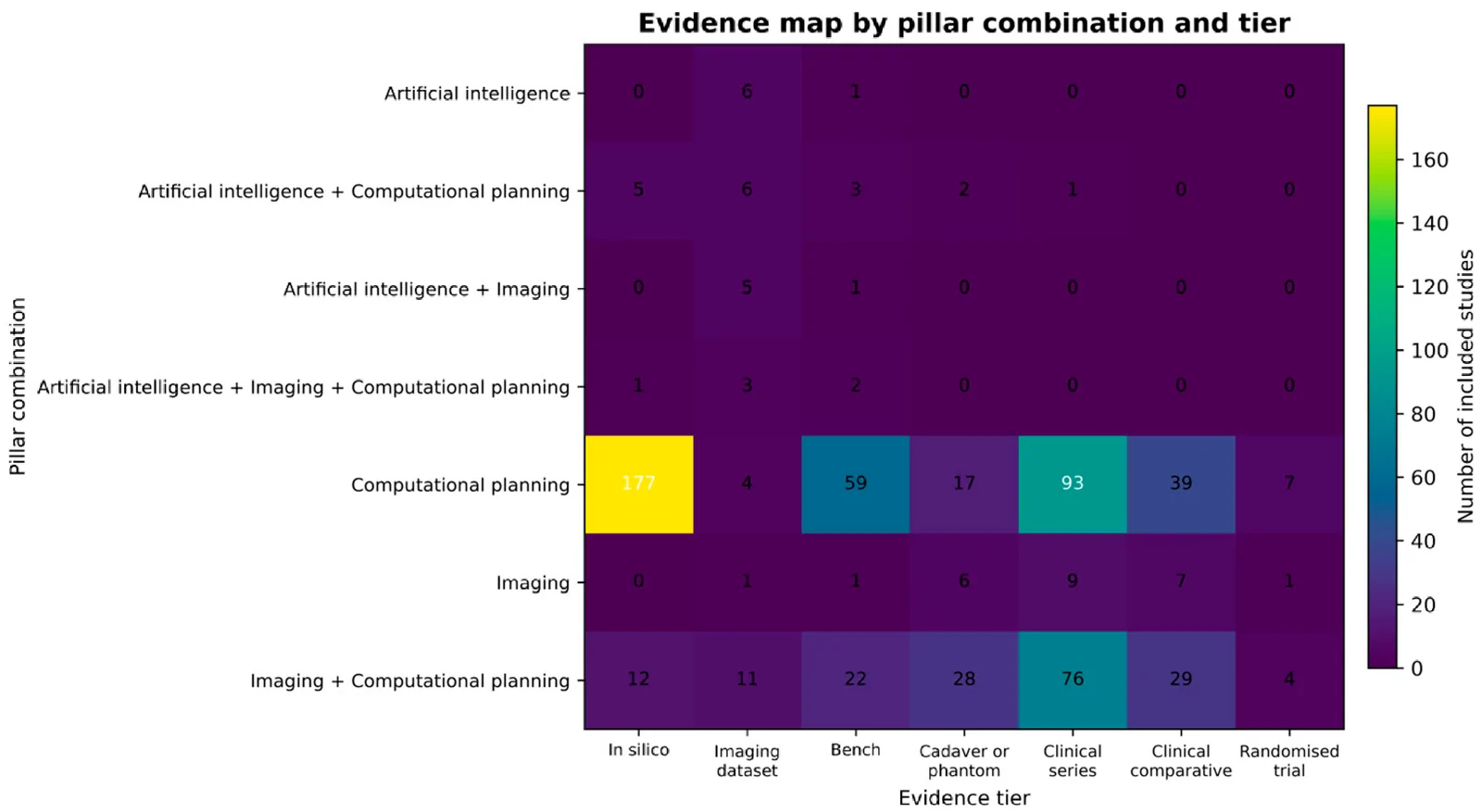
Evidence map. Included studies by pillar combination and highest evidence tier. Cell annotations are study counts derived from the canonical dataset. The sequential viridis scale is designed to remain distinguishable for common forms of colour-vision deficiency.

The included studies applied computational planning in 601 cases, an evaluated imaging step in 219 and artificial intelligence in 36; 396 applied computational planning alone, 182 imaging with computational planning, 25 imaging alone, 7 artificial intelligence alone and 29 artificial intelligence with one or both of the other pillars (Table 1, Figure 2). By tier, 195 studies were in silico, 89 bench, 53 cadaver or phantom, 36 imaging-dataset, 179 non-comparative clinical series, 75 comparative non-randomised clinical studies and 12 randomised trials; 266 studies carry a clinical evaluation, and 373 are technical. The craniomaxillofacial region accounts for 290 studies, the cranial vault for 92, the pelvis and acetabulum for 55, the knee for 48, other lower-limb sites for 40, the spine for 35, other upper-limb sites for 25, the hip for 23, the shoulder for 7 and the chest wall for 5, with 14 multi-region and 5 other. Clinical evaluation concentrates in the craniomaxillofacial region, where 164 of 290 studies are clinical, and 10 of the 12 randomised trials sit, whereas the knee (38 of 48 technical), the spine (31 of 35), the hip (22 of 23), the cranial vault (57 of 92) and the shoulder (5 of 7) are evaluated mainly in silico, on the bench or on imaging datasets. Publications rose from 14 studies in 2015 to 78 in 2021 and 98 in 2025, with 76 already in 2026 at the search date. The 266 clinical studies reported 7444 treated patients, 5524 of them in the craniomaxillofacial region; median analysed units were 3 for in silico and bench studies, 10 for cadaver or phantom, 71 for imaging-dataset, 12 for clinical series, 36 for comparative studies and 25.5 for randomised trials. Every count in this review is a count of studies, and studies are of very unequal size, so the two should not be read as the same thing. Among the technical studies, the median number of analysed units is 3, and half analyse three or fewer, which means a large part of the finite element and optimisation literature consists of single-anatomy demonstrations; among clinical studies, the median is 18, and almost none analyse three or fewer; among artificial intelligence studies it is 161.5, because a benchmark dataset supplies many cases at once. A region with many small technical studies therefore looks busier on the evidence map than a region with fewer but larger ones, and the map should be read as showing where work has been done rather than how much evidence it carries. We have not weighted the map by size, because the unit of analysis differs between tiers; an anatomy, a specimen, a scan and a patient are not commensurable, and a weighted count would imply a comparability the data do not support. A comparator was present in 533 studies: an alternative design or method in 222, a conventional implant in 153, a ground truth in 117, no treatment in 17, a manual design in 14 and another comparator in 10. Titanium was the stated material in 219 studies, and 233 did not state one; 158 of the 195 in silico studies designed an implant that was never manufactured, 147 studies used metal additive manufacturing and 49 used polymer additive manufacturing. A public-code statement was charted in 13 studies (4 of them with data as well) and a public-data statement in 39; 181 stated that nothing was available and 412 made no statement.

### 3.3. Artificial Intelligence in Implant Design

Thirty-six studies applied machine or deep learning to a step of the design pipeline (Table 2); 25 of them were charted from the abstract only. Their tasks, which overlap, were shape completion of a skeletal defect in 17, generation of the implant geometry in 15, segmentation for design in 9, prediction of design parameters in 6, prediction of an outcome such as stress, displacement or alignment in 5 and other tasks in 3. Seventeen concerned the cranial vault, where the AutoImplant benchmark datasets have made automatic cranioplasty design a standard task: convolutional, U-Net, conditional generative adversarial, masked-autoencoder and latent-diffusion models reconstruct the missing skull from the defective CT and report Dice coefficients above 0.9 with 95th-percentile Hausdorff distances between about 1 and 2 mm on held-out challenge cases [26,27,28,29]. Five were craniomaxillofacial, including an nnU-Net segmentation of the skull, mandible, teeth and mandibular canal for computer-assisted surgery evaluated on 153 internal test scans and a separate external set of 10 (the preprint was treated as a duplicate of the journal article) [30], an encoder–decoder network for maxillary and zygomatic defect completion, developed on single-centre scans and evaluated on 2377 synthetically defected test volumes, with one printed case checked for physical fit [31], and a neural-network surrogate that predicts plate stress and displacement from design parameters in place of repeated finite element runs [32]. Four were knee, three spinal, and two each hip and chest wall, with one each for another lower-limb site, a multi-region pipeline and another site.

The evidence tier is low. Twenty of the 36 were imaging-dataset studies, 7 bench, 6 in silico and 2 cadaver or phantom; one was a clinical series, in which a model predicting postoperative lumbar lordosis and pelvic tilt was used alongside the design of patient-specific spinal rods for 34 patients, without the text establishing that the model determined the rod design [33]. Under the review’s definition, evaluation on data independent of the development source (a different centre, an external public benchmark or a prospective set) was established for 4 studies (2 on another centre’s data, 1 on a public benchmark and 1 prospectively); 16 evaluated on a split of their own source (10 by a patient-level or random split, 5 on synthetically generated defects and 1 by a split not otherwise described), and 16 gave no usable description of where the evaluation data came from. Clinical use of a model was established for one study: a cranioplasty pipeline that evaluated its network on 1930 internally generated test volumes and then compared the automated with the manual design in one representative patient, whose implant was manufactured and implanted, reporting asymmetry indices of 2.22% for the automated and 2.05% for the manual design [34]. Code or model files were stated to be public in 6 studies and data in 9. No artificial intelligence study was comparative at the clinical tier, and none were randomised.

**Table 2:** Artificial intelligence studies.

Study	Region	Task	Model	Analysed Units	Independent Evaluation	Clinical Evaluation	Headline Accuracy Result	Code or Data
Chaubey et al. (2025) [35]	chest_wall	design_generation	3D U-Net with EfficientNet-B0 encoder	160	internal_only	no	DSC 0.2524; Hausdorff distance 148.90 mm	code
Shash et al. (2025) [36]	chest_wall	outcome_prediction	LR/Ridge/SVR/DT/RF/FCN/LightGBM	8	internal_only	no	AI model accuracy > 99.9%	unclear
Karlagina et al. (2022) [37]	cmf	other	U-Net	7	unclear	no	.	data
Dot et al. (2022) [30]	cmf	segmentation	nnU-Net	163	yes	no	test vDSC/sDSC: upper skull 96.22%/96.92%, mandible 94.19%/97.92%, upper teeth 94.83%/98.87%, lower teeth 94.38%/98.53%, mandibular canal 81.59%/97.90%	both
Beisekenov et al. (2025) [32]	cmf	parameter_prediction	feed-forward ANN + Bayesian network	.	internal_only	no	held-out test MAE 7.8 MPa for peak plate stress, 0.062 mm for displacement, and 6.4 MPa for screw stress	both
Juneja et al. (2026) [31]	cmf	shape_completion + design_generation	3D encoder–decoder CNN	2377	internal_only	no	DSC 0.778; HD95 3.453 mm	unclear
Carlos et al. (2026) [38]	cmf	segmentation	nnU-Net	.	unclear	no	Pseudo Dice near 1	unclear
Morais et al. (2018) [39]	cranial	shape_completion + design_generation	volumetric convolutional denoising autoencoder	.	unclear	no	.	data
Oldřich et al. (2019) [40]	cranial	segmentation	encoder–decoder CNN	.	unclear	no	.	unclear
Li et al. (2021) [41]	cranial	segmentation	voxel rearrangement network	.	unclear	no	.	code
Li et al. (2021) [26]	cranial	shape_completion + design_generation	CNN	167	internal_only	no	.	code
Sulakhe et al. (2022) [42]	cranial	shape_completion + design_generation	3D conditional GAN	.	unclear	no	.	unclear
Wodzinski et al. (2022) [27]	cranial	shape_completion + design_generation	modified U-Net	.	internal_only	no	Dice 0.91; boundary Dice 0.94; 95th-percentile Hausdorff distance 1.53 mm	both
Wu et al. (2023) [34]	cranial	shape_completion + design_generation	3D U-Net	1930	yes	yes	ACVAI 2.22% vs. 2.05% manual; PCVAI 2.14% vs. 6.03% manual	data
S et al. (2024) [43]	cranial	shape_completion + design_generation	3D U-Net with vision transformer	.	unclear	no	higher Dice than 3D U-Net and attention 3D U-Net	unclear
Kesornsri et al. (2024) [44]	cranial	shape_completion + design_generation	ConvNeXt	.	unclear	no	Dice 0.7969 ± 0.13	unclear
Zhang et al. (2024) [45]	cranial	design_generation + parameter_prediction	neural network	.	unclear	no	.	unclear
Wodzinski et al. (2024) [28]	cranial	shape_completion	latent diffusion + VQ-VAE + GAN	.	yes	no	Dice > 0.94 on SkullBreak and >0.96 on SkullFix	unclear
Wodzinski et al. (2024) [46]	cranial	shape_completion	masked autoencoder	.	internal_only	no	.	unclear
Wodzinski et al. (2025) [29]	cranial	shape_completion	deep neural network	.	yes	no	DSC 0.94; bDSC 0.94; HD95 1.31 versus 0.84/0.76/2.43 baseline	data
Juneja et al. (2025) [47]	cranial	shape_completion + design_generation	unclear	.	internal_only	no	DSC 0.889; Hausdorff distance 1.856 mm; SSIM 0.897	unclear
Salem et al. (2026) [48]	cranial	shape_completion + design_generation	3D U-Net	40	internal_only	no	mean Dice 0.901; Boundary Dice 0.908; HD95 1.52 mm	data
Nguyen et al. (2026) [49]	cranial	shape_completion	3D U-Net	250	internal_only	no	Dice 0.94; Hausdorff distance 3.8 mm	unclear
Guo et al. (2026) [50]	cranial	segmentation + design_generation + parameter_prediction + outcome_prediction	BASNet + XGBoost + U-Net + conditional diffusion	.	unclear	no	segmentation accuracy 94.7%; porosity error 3.2 ± 1.8%; elastic modulus error 4.1 ± 2.3%	unclear
Banis et al. (2025) [51]	hip	segmentation + other	UNETR	1300	unclear	no	FEA-DIC total displacement difference 0.17 ± 0.09 mm	unclear
Yáñez González-Cuéllar et al. (2026) [52]	hip	outcome_prediction	dual-CNN	392	unclear	no	mean absolute error reduced approximately 30% vs. random forest	unclear
Kanagalingam et al. (2023) [53]	knee	design_generation	unclear	2	unclear	no	mean surface deviation −0.10 mm for plate A and −0.11 mm for plate B	none
Burge et al. (2023) [54]	knee	segmentation + shape_completion + design_generation + other	CNN + U-Net + SSM	165	internal_only	no	femur model RMSE 0.87 (SD 0.20) mm and component RMSE 0.85 (SD 0.21) mm, OUH ≥3 mm in 6/84; tibia model RMSE 0.80 (SD 0.15) mm and component RMSE 0.82 (SD 0.27) mm, OUH ≥3 mm in 1/81	unclear
Grothues et al. (2023) [55]	knee	parameter_prediction	artificial neural network	673	internal_only	no	prediction error below each parameter’s standard deviation; regression had the lowest errors	unclear
Guezou-Philippe et al. (2025) [56]	knee	segmentation + shape_completion	2D U-Net + SSM	40	internal_only	no	segmentation Dice 98.5% (SD 0.8%); full-bone RMSE 1.0 mm (SD 0.3); implant-fit RMSE 0.9 mm (SD 0.5)	none
He et al. (2023) [57]	lower_limb_other	segmentation	nnU-Net	.	internal_only	no	DSC > 0.95; HD95 < 0.6 mm	unclear
Mazzocchetti et al. (2024) [58]	multi	shape_completion	point-cloud completion networks	71	internal_only	no	PointAttN accuracy 1.4468 mm; Chamfer distance 3.2731 mm; F-score 0.8929	data
Zhou et al. (2025) [59]	other	outcome_prediction	neural network + random forest	80	internal_only	no	prediction error 2–6% for nonlinear machine-learning models	unclear
Biswas et al. (2020) [60]	spine	parameter_prediction	artificial neural network	.	unclear	no	.	unclear
Von Atzigen et al. (2022) [61]	spine	parameter_prediction	stereo neural network	.	unclear	no	.	unclear
Ngo et al. (2026) [33]	spine	outcome_prediction	unclear	34	unclear	unclear	postoperative-minus-predicted lumbar lordosis −3.8° and pelvic tilt −0.1° at 2 years	unclear

cmf, craniomaxillofacial; ACVAI and PCVAI, anterior and posterior cranial vault asymmetry index; ANN, artificial neural network; bDSC, boundary Dice similarity coefficient; CNN, convolutional neural network; DSC, Dice similarity coefficient (vDSC, volumetric; sDSC, surface); DT, decision tree; FCN, fully connected neural network; FEA-DIC, finite element analysis compared with digital image correlation; GAN, generative adversarial network; HD95, 95th-percentile Hausdorff distance; LR, linear regression; MAE, mean absolute error; OUH, maximum over- or underhang of the implant edge; RF, random forest; RMSE, root-mean-square error; SD, standard deviation; SSIM, structural similarity index; SSM, statistical shape model; SVR, support vector regression; VQ-VAE, vector-quantised variational autoencoder. U-Net, nnU-Net, UNETR, ConvNeXt, EfficientNet, BASNet, PointAttN, XGBoost and LightGBM are the architecture or software names used by the original authors. Region, task and evaluation labels (e.g., shape_completion, internal_only) are the charting vocabulary defined in Text S4; internal_only, evaluated only on a split of the development source; a full stop (.) indicates not reported.

Because these 36 studies do quite different things, they are also reported by task rather than as one set (Table S7). Shape completion accounts for 17 of them, design generation 15, segmentation 9, prediction of a design parameter 6 and prediction of an outcome 5, and the tasks overlap where a study segments an anatomy and then generates an implant from it. Evaluation independent of the development source was established for 3 of the 17 shape-completion studies, 1 of the 15 design-generation studies, 1 of the 9 segmentation studies and none of the 11 prediction studies, so the maturity of the subfields differs and the aggregate figure conceals that.

The cranial studies were tabulated by the dataset they evaluated on, because this literature reuses a small number of public benchmarks and reuse carries a leakage risk. Two coders from different vendors read each study. Across the 17 cranial studies, the named evaluation data were SkullFix in 6, SkullBreak in 4, AutoImplant in 2, another named public dataset in 5 (most often MUG500+), institutional data in 5, and synthetically generated defects in 7; 5 named no evaluation dataset at all. Eight of the seventeen, or 47.1%, evaluate on data from the AutoImplant family, which means a substantial part of what is known about automatic cranial implant design rests on one benchmark lineage, and a method that performs well there has not thereby been shown to generalise. On the risk that evaluation data were not independent of development data, the two coders agreed on low risk for 2 of the 36 studies and on high risk for 2, agreed that 17 could not be judged from the text, and differed on 11; the dominant finding is that most of these papers do not describe how their evaluation cases relate to their training cases, which is a reporting failure rather than a demonstrated defect. Against the prediction-model reporting items, calibration is not an applicable concept for 26 of the 36, whose output is a geometry rather than a probability, and among those where it does apply, it was reported in 2 and not reported in 4; handling of missing data was described in a minority and a sample size was justified in fewer still. PROBAST + AI was not applied for the reason given in Section 2.6.

### 3.4. Imaging Pipeline Evaluations

An imaging step was itself evaluated in 219 studies. The most common was mirroring of the contralateral anatomy to generate the target shape (99 studies, 72 of them clinical, mostly orbital and midface reconstruction), followed by registration of the postoperative result to the plan or of intraoperative to preoperative data (65, 49 clinical), segmentation accuracy for the design (13, all technical), template or atlas fitting (12), statistical shape modelling (10, all technical) and the effect of the imaging protocol or modality on the implant (6). The clinical imaging studies mostly report the geometric accuracy of the implanted result against the virtual plan: of the 266 clinical studies, 181 report an accuracy measure, typically a mean surface or landmark deviation of the order of 1 mm for orbital, orthognathic and mandibular implants, and 85 report a design, planning or operative time. Only 6 studies, analysing between 10 and 83 units each, examined how acquisition parameters propagate into implant geometry, and the 13 segmentation-accuracy studies evaluated segmentation against a reference without carrying the error through to an implant. Statistical shape models appeared as design tools in 13 studies and as imaging evaluations in 10, all below the clinical tier. Of the studies with an imaging pillar, 131 were charted from full text and 88 from the abstract.

### 3.5. Computational Planning

Computational planning was applied in 601 studies. Finite element analysis was the most frequent method (312 studies, 281 technical and 31 clinical), followed by virtual surgical planning of the implant (261, of which 223 clinical), parametric design (113), topology optimisation (49), automated design (43), lattice optimisation (42), shape optimisation (21), musculoskeletal modelling (16) and statistical-shape-model-driven design (13). Table 3 summarises the technical evidence by region and method. The finite element literature is dominated by single-anatomy studies: median analysed units were 3 in silico, and of the 373 technical studies, 284 report a mechanical result, usually peak von Mises stress, displacement or stiffness under a static load case, whereas 104 report a geometric accuracy result. Against the six credibility items, the distributions among the 373 technical studies were as follows: validation data or specimens independent of development—119 yes, 181 no, 73 unclear; quantitative comparison against a physical experiment or ground truth—182 yes, 179 no, 12 unclear; uncertainty or sensitivity analysis—187 yes, 142 no, 44 unclear; inputs specified reproducibly—174 yes, 17 no, 182 unclear; code, model or design files available—17 yes, 134 no, 222 unclear; and more than one anatomy or specimen evaluated—149 yes, 183 no, 41 unclear.

Those totals mix two different things and should not be read as they stand, because an item recorded as unclear in an abstract-only record says nothing about the study. The primary reading is therefore restricted to the studies read in full, and within those to the records where the item could be read at all. On that basis, independent validation material was used in 48.8% of the 168 full-text records where the item could be read, against 28.0% of the 132 readable abstract-only records; a physical or ground-truth comparison in 58.5% of 176 against 42.7% of 185; an uncertainty or sensitivity analysis in 64.4% of 174 against 48.4% of 155; inputs specified reproducibly in 94.1% of 170 against 66.7% of only 21 readable; files available in 7.2% of 138 against 53.8% of only 13 readable; and more than one anatomy in 44.5% of 173 against 45.3% of 159. Two of these deserve care rather than a headline. Reproducible input specification and file availability are unclear in 89.3% and 93.4% of abstract-only records, so the abstract percentages rest on a handful of records, and the full-text figures are the only usable ones; the apparently higher file availability in abstracts is due to selection, since an abstract mentions shared data only when the authors treat it as a selling point. The substantive finding survives the restriction and is a full-text finding: among technical studies that can actually be read, independent validation material is used in fewer than half, a physical comparison in about three in five, and code, model or design files are available in about one in fourteen.

How reproducible are these judgements? Two further coders from different vendors applied the same six items independently to a seeded random sample of 90 technical studies. They agreed with each other on 75.6% to 98.9% of records per item (kappa 0.32 to 0.91) and with the original coding on 51.1% to 88.9%. Agreement was highest for file availability (98.9% between the new coders) and for reproducible inputs (86.7%) and lowest for independent validation material and for uncertainty analysis, where the new coders matched the original coding only about half the time. Those two items are exactly the ones carrying the review’s strongest claims about validation, so the counts above should be read as approximate, with the direction more reliable than the precise figure. The recurring source of disagreement is what counts as varying an input and whether comparing a computed result against published experimental values from another study is a physical comparison. Within the 281 technical finite element studies, inputs were specified reproducibly in 126 (8 no, 147 unclear), independent validation material was used in 76 (163 no, 42 unclear), a physical or ground-truth comparison was made in 108 (166 no, 7 unclear) and files were available in 6 (98 no, 177 unclear); the precise reading is therefore that input specification is usually present where it can be read, whereas independent validation, physical comparison and file availability are usually absent where they can be read. Topology, lattice and automated design studies were almost entirely technical (46 of 49, 39 of 42 and 40 of 43), with 3 clinical studies each; virtual surgical planning was the only computational method whose evidence lies mainly at the clinical tier.

### 3.6. Clinical Evidence

Clinical evaluation of a patient-specific implant designed with an evaluated pillar was reported in 266 studies: 179 non-comparative series (Table S1), 75 comparative non-randomised studies and 12 randomised trials (Table 4). The comparative studies concentrated in the craniomaxillofacial region (60 of 75), with 5 pelvic and acetabular, 4 cranial, 2 upper-limb and 1 each spinal, hip, knee and shoulder; 49 compared against a conventional implant, 22 against an alternative design or planning method, 3 against a manual design and 1 against another comparator. The 12 randomised trials randomised between 10 and 58 patients each (median 25.5); 10 were craniomaxillofacial (patient-specific orthognathic or maxillary osteosynthesis plates against conventional plates or splint-based planning in 5, orbital implants in 3, a maxillary ridge augmentation mesh in 1, mandibular fracture plates in 1), one compared a patient-specific acetabular plate with intraoperative contouring and one a custom osteosynthesis plate for the distal humerus with conventional plating. The implant was designed by virtual surgical planning in 11 trials and by mirroring-based mesh adaptation without a stated planning comparison in one orbital trial; an imaging step was itself evaluated in 5. Reported outcomes were operative time or blood loss, accuracy of the transferred plan, occlusal or orbital position, complications and, in the distal humerus trial, bone union and elbow function; serious adverse events were not consistently reported, and their absence from a headline result is not evidence of no difference. Outside the craniomaxillofacial region the clinical evidence is predominantly non-comparative but not exclusively so: the cranial vault has 31 series and 4 comparative studies, the pelvis and acetabulum 17 series, 5 comparative studies and 1 randomised trial, other upper-limb sites 4 series, 2 comparative studies and 1 randomised trial, other lower-limb sites 11 series, the knee 9 series and 1 comparative study, the spine 3 series and 1 comparative study, the shoulder 1 series and 1 comparative study, the hip 1 comparative study and multi-region series 9. Of the 266 clinical studies, 224 report a clinical outcome, 181 a geometric accuracy outcome and 85 a time outcome; follow-up was stated in 159. By text source, a clinical outcome was charted for 113 of the 119 clinical studies with full text and 111 of the 147 charted from abstracts, an accuracy outcome for 75 and 106, and a time outcome for 53 and 32.

**Figure 3:**
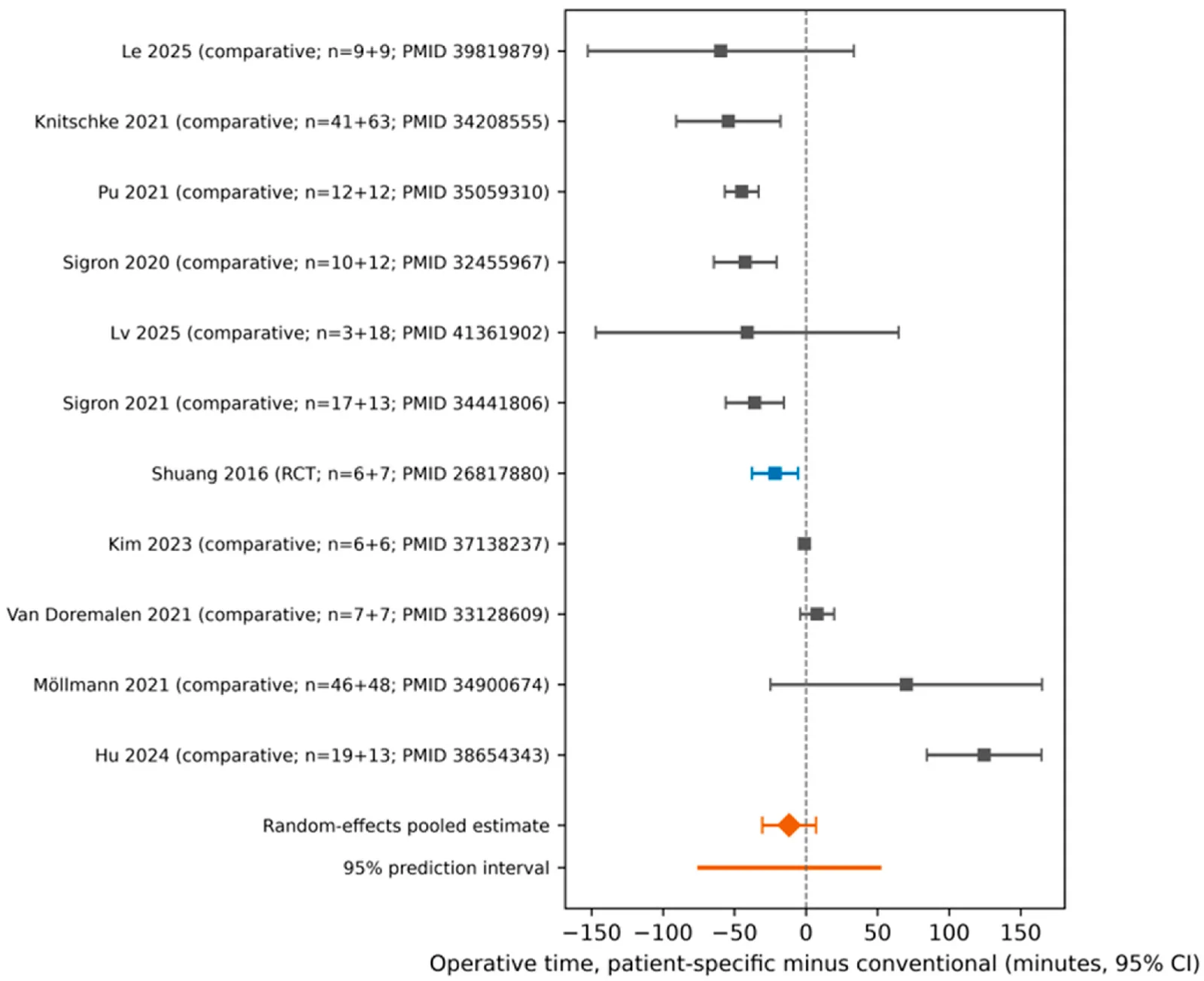
Forest plot of operative time. Difference in operative time (minutes) between the patient-specific implant arm and the conventional comparator arm in the studies with extractable arm-level estimates (amendment A8), with the DerSimonian–Laird random-effects pooled estimate and its 95% prediction interval. Squares are individual studies with 95% confidence intervals (grey, comparative non-randomised studies; blue, the randomised trial); the orange diamond is the pooled estimate and the orange bar its 95% prediction interval; the dashed vertical line marks no difference. Studies, top to bottom: Le 2025 [119], Knitschke 2021 [93], Pu 2021 [141], Sigron 2020 [85], Lv 2025 [146], Sigron 2021 [94], Shuang 2016 [73], Kim 2023 [108], van Doremalen 2021 [147], Möllmann 2021 [96] and Hu 2024 [142]. The comparison concerns the delivered implant, not a design method.

The prespecified meta-analysis condition was tested on a dedicated extraction of arm-level estimates, one row per outcome, from the 40 included comparative studies and trials with full text, of which 26 reported at least one listed outcome for both arms (Table 5; the outcome-by-study disposition is Table S6). Membership was taken from the charted comparator class (conventional implant) and, for accuracy, from the measure (deviation of the delivered result from the preoperative plan; clinical symmetry measures were not pooled), with one measure per study, preferring an overall or total deviation. Deviation from the plan was extractable from 7 studies (1 randomised, 6 comparative; 243 patients), and pooling those 7 gives a standardised mean difference of −0.72 (95% CI −1.32 to −0.13) favouring the patient-specific arm, with I-squared 76%. That estimate was the one reported in the submitted version, and examining it at revision showed that it should not be read, because the pool mixed measures of different things. Two adjudicators read each of the seven papers’ methods. In one study the measure compares postoperative with preoperative anatomy and never references the plan at all, and in a second the adjudicators disagreed about whether it does; both are therefore outside a pool defined as deviation from plan. In two further studies, the deviation is reported as a signed value with zero as the target, so the arm that sat closer to the plan carries the numerically larger mean: one of these entered the submitted pool contributing +0.32, that is, in favour of the conventional implant, although its own authors report the patient-specific arm as more accurate. Restricting the pool to the five studies whose measure references the virtual plan gives −0.85 (−1.69 to −0.01) with I-squared 78%, and restricting it further to the four in which a smaller value also means a better result gives −1.21 (−1.69 to −0.74) with I-squared 0%. The heterogeneity that made the original estimate hard to interpret was therefore largely an artefact of pooling incommensurable measures rather than a property of the studies. We report −1.21 from 4 studies as the primary estimate, all of it craniomaxillofacial, and the per-study adjudication with its quoted evidence is in Text S6. The certainty of this estimate remains very low, and its direction should not be read as established: four small non-randomised or single-trial comparisons agreeing with one another is a weak basis for a claim about accuracy. Operative time was extractable from 11 studies (1 randomised, 10 comparative; 384 patients): the pooled mean difference was −11.8 min (95% CI −30.5 to 6.9) with I-squared 93%, a Hartung–Knapp interval of −46.3 to 22.7, a 95% prediction interval of −74.6 to 51.0 min and leave-one-out estimates from −24.0 to −7.3 (Figure 3); direction varied within and between regions, with pelvic reconstructions and one mandibular study longer in the patient-specific arm, and the Egger intercept was −1.26 (*p* = 0.601). Revision or removal of the implant was extractable from 8 comparative studies (284 patients), none of them randomised: the pooled risk ratio was 0.28 (95% CI 0.11 to 0.72) favouring the patient-specific arm, with I-squared 0%, a Hartung–Knapp interval of 0.12 to 0.66 and a prediction interval of 0.09 to 0.91. In absolute terms, 3 of 135 patients in the patient-specific arms were revised or had the implant removed (2.2%) against 25 of 149 in the conventional arms (16.8%), a difference of 14.6 percentage points (95% CI 8.1 to 21.1), or about 146 fewer revisions per 1000 patients treated, which corresponds to one revision avoided for every 7 patients. Those absolute figures come entirely from non-randomised studies in which the two arms were assigned by period, surgeon or case complexity without adjustment, so the certainty is very low and the true difference could be substantially smaller, absent or in the opposite direction. Any complication was extractable from 8 studies (1 randomised; 242 patients), risk ratio 1.05 (95% CI 0.8 to 1.36), 41 of 126 against 41 of 116 patients, an absolute difference of −2.8 percentage points (95% CI −14.7 to 9.1); and infection from 6 (2 randomised; 133 patients), risk ratio 0.94 (95% CI 0.3 to 2.99), 3 of 66 against 3 of 67, a difference of 0.1 percentage points (95% CI −7.0 to 7.1). Neither interval excludes a difference in either direction, and with 6 to 8 small studies and few events, these are findings of imprecision rather than evidence that the arms are alike. The same applies to operative time, where the interval runs from 30.5 min saved to 6.9 min lost: the review cannot tell whether patient-specific implants shorten operating time and does not show that they do not. Blood loss and function were reported for both arms in 5 and 4 studies respectively but without extractable dispersion, so they were not pooled. All pooled estimates describe the delivered patient-specific implant against a conventional one; no study compared one design pipeline against another on the same implant type.

### 3.7. Risk of Bias, Technical Credibility and Certainty

All 266 clinical-tier studies were appraised twice, independently, by assessors from different vendors. The two agreed on the overall judgement for 75.0% of the 12 randomised trials, 86.7% of the 75 comparative studies and, for the case series, 32.4% exactly and 60.3% within one item of the ten. Where they differed on the comparative studies, the second assessor was the more severe: the first recorded 2 studies at low and 4 at moderate risk, with 69 at serious; the second recorded none below serious and placed 4 at critical. Both therefore reach the same conclusion about the body of evidence: that almost every non-randomised comparison is at serious risk of bias from confounding, and neither supports a claim that any particular study is sound. Agreement on the case-series checklist is poor, and the median item count reported below should be read as an indication rather than a measurement. Cohen’s kappa is not quoted for the comparative studies because one category accounts for almost every judgement, which drives kappa towards zero despite high observed agreement; the full distribution of each assessor’s judgements is given instead, and every disagreement is listed per study in Table S4. The figures below are the first assessor’s, with the second assessor’s in Table S4 (Table 6; per-study judgements in Table S4). Of the 12 randomised trials, 10 were at some concerns and 2 at high risk of bias under RoB 2; none were at low risk because none reported a prespecified analysis plan (selection of the reported result was at some concerns in all 12) and randomisation was adequately described in 2. Of the 75 comparative non-randomised studies, 69 were at serious, 4 at moderate and 2 at low overall risk under ROBINS-I; confounding was serious in 68, because patient-specific and conventional implants were assigned by period, surgeon or case complexity without adjustment, and 68 gave no information on selection of the reported result. The 179 non-comparative series met a median of 5 of the 10 JBI items; 100 met five or fewer, and 35 met eight or more, and the items least often met were consecutive inclusion (35 series) and complete inclusion (31), against 169 that reported outcomes clearly. Judgements for abstract-only records rest on absence of information and are labelled as such in Table S4. Funding and conflict statements could be read only in the full-text records: a funding source was stated in 34 studies as public, in 4 as industry, in 5 as mixed and in 19 as none, a conflict of interest was declared in 9 and denied in 57, and an author with a manufacturer or commercial design-service affiliation was identified in 5 (Table S5); for the remaining full-text records, no statement was found, and abstract-only records are unclear. Technical credibility is summarised in 3.5 and per study in Table S3. Certainty of evidence per outcome is set out in Table 7: very low for deviation from the plan, because the body of evidence is mostly non-randomised, at serious risk of bias, measured in different ways across studies and indirect to the review question; very low for operative time, with very serious inconsistency added; very low for revision or removal, any complication and infection, where events are few, definitions differ, and reporting is incomplete; and very low for the clinical outcomes outside the craniomaxillofacial region, where comparative and randomised studies exist but are sparse and unpooled.

Domain-level judgements. RoB 2 (12 trials): randomisation, low 2, some concerns 10; deviations from intended interventions, low 2, some concerns 9, high 1; missing outcome data, low 5, some concerns 6, high 1; measurement of the outcome, low 5, some concerns 7; selection of the reported result, some concerns 12. ROBINS-I (75 studies): confounding, low 1, moderate 5, serious 68, no information 1; selection of participants, low 9, moderate 19, serious 16, no information 31; classification of interventions, low 75; deviations from intended interventions, low 9, moderate 5, serious 1, no information 60; missing data, low 17, moderate 5, serious 10, no information 43; measurement of outcomes, low 9, moderate 64, serious 1, no information 1; selection of the reported result, moderate 6, serious 1, no information 68. JBI checklist items met (179 series): Q1 96, Q2 84, Q3 77, Q4 35, Q5 31, Q6 83, Q7 116, Q8 169, Q9 61, Q10 102 (item wording in Text S4). JBI, Joanna Briggs Institute; RoB 2, Cochrane risk-of-bias tool for randomised trials, version 2; ROBINS-I, Risk Of Bias In Non-randomised Studies of Interventions.

Starting level: The starting level was high for randomised bodies and low for non-randomised or mixed bodies (the non-randomised studies carry most of the weight). Deviation of the delivered implant from the preoperative plan: Primary estimate from the four studies that measure deviation from the virtual plan with smaller meaning better (Section 3.6 and Text S6); the prespecified seven-study pool, −0.72 (−1.32 to −0.13), is given in Table 5. The pooled standardised mean difference favours the patient-specific arm, but its applicability to the design methods is indirect. Operative time: Direction differs by region; no summary claim is warranted. Any complication: Event outcomes were pooled from a single extraction pass; reporting of adverse events was incomplete across the corpus. Infection: Event outcomes were pooled from a single extraction pass; reporting of adverse events was incomplete across the corpus. Revision or removal of the implant: Event outcomes were pooled from a single extraction pass; reporting of adverse events was incomplete across the corpus. Clinical outcomes of computationally designed implants outside the craniomaxillofacial region: Comparative and randomised evidence exists outside the craniomaxillofacial region but is sparse and unpooled; the series predominate.

### 3.8. How Much Lies Outside the Searched Databases?

The review searched biomedical indexes and OpenAlex and not Embase, Scopus, Web of Science, IEEE Xplore or CINAHL. Two measurements bound what that omits.

The first uses the review’s own data. OpenAlex, the one source that is not biomedical-only, was the sole route to 68 of the 639 included studies, or 10.6%. Those 68 are overwhelmingly technical: 57 sit at the in silico, bench, cadaver or imaging-dataset tiers against 11 at clinical tiers, and 11 carry an artificial intelligence pillar. Adding one broader index therefore changed the technical map appreciably and the clinical map very little.

The second measurement probes the literature the review did not reach. The review’s own concept blocks were run against engineering-type sources in OpenAlex, retrieving 989 unique records, of which 379 were already in this review’s corpus, and 610 were not. A random sample of 40 of the 610 was screened against the eligibility criteria: 2 were clearly eligible, 34 clearly not, and 4 could not be settled from the title and abstract. Taking only the clear includes, 5.0% (95% CI 1.4% to 16.5%) of the absent records would have been eligible, which projects to about 31 further studies (95% CI 8 to 101); counting the uncertain records as eligible raises that to about 92 (43 to 177). Both estimates are small beside 639, and both consist of technical work: the two clear includes were a topology-optimised mandibular plate and an accuracy study of additively manufactured bone plates. The probe found no sign of a hidden body of randomised or comparative clinical evidence in engineering venues.

The conclusion we draw is specific. The review’s clinical findings, which rest on randomised and comparative studies indexed in MEDLINE, are unlikely to be changed by the missing databases. Its technical counts are an undercount, probably by something between 1% and 15%, concentrated in finite element and optimisation work, so statements such as “finite element analysis appeared in 312 studies” should be read as a floor. This is why the title and the aims of this review describe the biomedical-indexed literature rather than the field as a whole.

## 4. Discussion

### 4.1. Principal Findings

Across 639 studies published since 2015, the evidence for the three pillars of patient-specific implant design is deep in one place and shallow almost everywhere else. Virtual surgical planning, usually with mirroring or registration, is the only method whose evidence reaches the randomised tier, and it does so mostly in craniomaxillofacial surgery, in small trials that evaluate the delivered implant rather than the planning method. Pooled across the four studies that measure deviation from the virtual plan in a direction where smaller is better, the delivered patient-specific implant deviated less from its plan than a conventional implant did, and across eight comparative studies it was revised or removed less often, namely 3 of 135 against 25 of 149 patients; operative time, any complication and infection gave intervals too wide to tell whether a difference exists. Every estimate is of very low certainty, every one of them comes from craniomaxillofacial surgery, and none compare one design method with another. Finite element analysis is the most frequent method in the corpus but is applied overwhelmingly in single-anatomy in silico studies; where the text can be read, inputs are usually specified, but independent validation material, comparison against a physical experiment and availability of model or design files are usually absent. Topology, lattice and shape optimisation, automated design, musculoskeletal modelling and statistical shape modelling remain almost entirely technical. Artificial intelligence contributes 36 studies, none comparative at the clinical tier, 2 in which a model was used in the care of a real patient, 4 with evaluation on data independent of the development source, and 6 with public code; favourable offline reconstruction or segmentation metrics are not evidence of clinical performance, and on the present literature no claim about clinical benefit from artificial intelligence in implant design can be supported at all; its strongest evidence is benchmark performance for cranial defect completion, a task made tractable by the AutoImplant datasets. Outside the craniomaxillofacial region, comparative and randomised evidence for computationally designed patient-specific implants exists in the pelvis, the cranial vault and the upper limb but is sparse, and most of the clinical evidence there rests on series that do not report consecutive or complete inclusion.

### 4.2. Relation to Prior Reviews

The region-specific reviews and registrations identified in the registry search ask whether patient-specific implants perform as well as conventional ones in cranioplasty, the orbit, orthognathic surgery, the jaws, the pelvis, the spine and long bones [1,3,5,6,7,8,9,11,12]. Their positive answers are compatible with the present map and with its pooled plan-deviation estimate: the same craniomaxillofacial trials and comparative studies populate our clinical tier. What those reviews do not show is that the implants they evaluate were produced by a pipeline whose components are validated to very different degrees and that outside their regions the same components have little clinical evaluation. The nearest AI-oriented review on three-dimensional templating for arthroplasty [149] and the deep learning cranial implant design study that frames that task as a benchmark problem [14] are each confined to one task; the present review places those tasks in the wider design pipeline and shows that the deficit of independent and clinical evaluation is general. Reviews of statistical shape model accuracy [18] and the credibility framework for computational models [15] provide the standards against which the technical studies here fall short.

### 4.3. Strengths and Limitations

The review’s strengths are procedural: a protocol committed before retrieval with every deviation dated; a recall-oriented four-block search with a rule layer whose exclusions are published and audited; two independent screeners from different vendors under a recall-union rule with the full cross-tabulation reported; charting from a written instruction file, with an independent second charter on a seeded sample and every adjudicated correction recorded separately; a prespecified meta-analysis test run on a dedicated extraction with sensitivity analyses; machine-generated counts checked by a verification script; and two rounds of independent adversarial pre-review, whose findings and their disposition ship with the package. The limitations are substantial and several are specific to the workflow. Screening, assessment, charting and appraisal were performed by AI systems. At revision, every clinical study was appraised a second time by an assessor from a different vendor, the six credibility items were coded again on a sample by two further coders, and a reference standard was built for the charting sample by two adjudicators reading the source text. Those additions measure the problem rather than remove it. Against the adjudicated reference standard, the primary charter was correct on 85% to 100% of records depending on the field, which is reassuring but represents agreement between independent machine readings of the same text, not verification by a person. No human-verified subsample exists for this review, and both screeners read the same instruction file, so a blind spot written into that file would be invisible to every check reported here. The credibility items proved only moderately reproducible, with the two items carrying the strongest claims matching the original coding about half the time, and agreement on the case-series checklist was poor. A reader should treat the direction of these findings as better supported than any individual count and should treat the evidence map as a machine-generated artefact that has been checked by machines. The two rounds of independent review found residual duplicate reports, an unevenly applied dental boundary, overstated descriptions of three artificial intelligence studies, clinical-tier rows without a verified patient denominator, records charted from a title alone, and a pooled outcome that mixed constructs; all were corrected and the corrections are recorded, but they show that the machine-generated corpus needed independent checking and that further errors of the same kind may remain. Full text was available for fewer than half of the reports sought, so 344 of the 639 included studies were charted from the abstract, which inflates the unclear categories (material, manufacturing, funding, availability and, above all, the credibility items), can misstate a tier or an evaluation source in either direction, and limits the risk-of-bias judgements for abstract-only clinical studies to the absence of information; the artificial intelligence set is the most affected, with 25 of 36 charted from abstracts. Sixty reports had neither an abstract nor retrievable text: 47 of these are held unresolved and the other 13 were excluded as second copies of reports already assessed, so they are counted among the duplicate exclusions rather than among the unresolved. A first full-text retrieval attached the wrong articles to most records through an incorrect link type; the defect was caught by the second charters, the affected outputs were discarded and every text was re-retrieved with a title-match guard, but the episode illustrates the fragility of automated retrieval and is reported for that reason. Embase, Scopus, Web of Science, IEEE Xplore and CINAHL were unavailable, OpenAlex was searched only in a supplementary run a day after the main search, the Crossref sweep was to provide supplementary discovery of unknown recall, and the citation chase covered only reference lists indexed in PubMed Central. The review’s coverage and gap claims are therefore claims about the biomedical indexes and OpenAlex, not about the engineering literature as a whole, which is why the title says so. Section 3.8 measures the shortfall rather than asserting it: one broader index was the sole route to 10.6% of the included studies, almost all technical, and a probe of engineering-indexed records absent from this corpus projects roughly 31 further eligible studies (95% CI 8 to 101), again technical. That is consistent with what is known more generally about database selection in systematic reviews, where a substantial minority of eligible records are found in only one database, and the records missed by a narrow search are not a random sample of the literature [150,151], although the effect of extending beyond one index on pooled effect estimates is often modest [152]. Applied here, that means the descriptive proportions in the evidence map are conditional on the databases searched and the technical counts are a floor, while the clinical findings, which rest on MEDLINE-indexed randomised and comparative studies, are unlikely to move. The underlying problem, that a structure inferred from an incompletely observed network can differ systematically from the whole, has been formalised in network science, where how far a sampled subgraph preserves the properties of the full graph depends both on how much is missing and on whether it is missing at random [153]. That work treats random and non-uniform random sampling, and the omission here is neither: it is correlated with discipline, venue and indexing age, which is the less forgiving case, so the bound we report rests on the empirical screening in Section 3.8 rather than on an analytical result. The rule-layer audit bounds the false-exclusion rate only loosely. Sixty-seven reports remain unresolved and 16 included studies are preprints. The pooled estimates rest on between 6 and 11 studies from a single extraction pass (a first pass that returned one outcome per study was found incomplete by the third independent review and was repeated per outcome), compare the delivered implant rather than any design method, and are very low certainty; publication bias could not be assessed for the smaller pools and the small-study test on the operative-time pool is weak under its heterogeneity. Finally, the review counts studies, not implants or patients, and a region with many small series may appear better evidenced than a region with a single large registry.

### 4.4. Implications and Research Agenda

Three implications follow. For the interpretation of the clinical literature, the accuracy and complication figures reported for patient-specific implants are properties of an entire pipeline whose upstream components are rarely evaluated in the same study; a clinical series that reports a 1 mm deviation from plan says nothing about whether a different segmentation, a different mirroring procedure or a different finite element model would have produced a different implant, and the pooled plan-deviation and revision advantages found here are evidence about delivered implants, not about which pipeline produced them. For engineering studies, the credibility items that were most often unmet where they could be read—independent validation material, comparison against a physical experiment and availability of files—are exactly the items that make an in silico result transferable to the next patient, and the reporting frameworks that already exist for computational models and for imaging AI could be adopted without waiting for a design-specific standard. For artificial intelligence, the field has benchmark-level evidence for cranial shape completion and almost nothing else: the next studies needed are not further architectures on the same datasets but evaluation on independent centres, prospective use in design workflows with the manual design as comparator, and release of models and data under governance. Comparative clinical studies of design methods, in which the same implant type is produced by two pipelines, would answer the question that the clinical trials of implants cannot.

## 5. Conclusions

The evidence for artificial intelligence, imaging and computational planning in patient-specific skeletal implant design is unevenly distributed across a large body of literature. Virtual surgical planning with mirroring or registration has randomised and comparative clinical evidence, concentrated in craniomaxillofacial surgery and evaluating the delivered implant rather than the method; pooled across the studies with extractable estimates, delivered patient-specific implants deviate less from their plan and are revised less often than conventional ones, at very low certainty. Finite element analysis and optimisation methods are applied widely, validated against independent material or physical experiments in a minority of the studies that can be read, and shared almost never. Artificial intelligence has benchmark evidence for cranial defect completion, little evaluation on independent data and almost no clinical use. The clinical evidence is also weak in itself, and this should carry as much weight as the effect estimates: no randomised trial reached low risk of bias, and almost every non-randomised comparison was at serious risk from confounding, because patient-specific and conventional implants were assigned by period, surgeon or case complexity and the analyses did not adjust for it. Clinicians reading outcome studies of patient-specific implants should read them as evidence about pipelines whose components are unequally validated, and investigators should direct the next studies at those components rather than at the implants alone.

## Figures and Tables

**Table 1:** Evidence map by pillar and tier.

Pillar Combination	In Silico	Imaging Dataset	Bench	Cadaver or Phantom	Clinical Series	Clinical Comparative	Randomised Trial	Total
Artificial intelligence	0	6	1	0	0	0	0	7
Artificial intelligence + Computational planning	5	6	3	2	1	0	0	17
Artificial intelligence + Imaging	0	5	1	0	0	0	0	6
Artificial intelligence + Imaging + Computational planning	1	3	2	0	0	0	0	6
Computational planning	177	4	59	17	93	39	7	396
Imaging	0	1	1	6	9	7	1	25
Imaging + Computational planning	12	11	22	28	76	29	4	182
Total	195	36	89	53	179	75	12	639

**Table 3:** Technical evidence by region and method.

Region	Pillar	Method	Studies	Tier Breakdown	Analysed Units Range
chest_wall	computational	fea	3	In silico: 2; Bench: 1	1 to 8
chest_wall	computational	parametric_design	1	Bench: 1	3 to 3
chest_wall	computational	ssm_design	1	Cadaver or phantom: 1	288 to 288
cmf	computational	automated_design	7	In silico: 2; Imaging dataset: 1; Bench: 1; Cadaver or phantom: 3	1 to 21
cmf	computational	fea	107	In silico: 73; Imaging dataset: 2; Bench: 28; Cadaver or phantom: 4	1 to 2377
cmf	computational	lattice_opt	9	In silico: 3; Bench: 6	1 to 8
cmf	computational	musculoskeletal_model	4	In silico: 2; Bench: 2	1 to 7
cmf	computational	parametric_design	23	In silico: 12; Bench: 9; Cadaver or phantom: 2	1 to 40
cmf	computational	shape_opt	9	In silico: 8; Bench: 1	2 to 25
cmf	computational	ssm_design	3	Imaging dataset: 3	19 to 35
cmf	computational	topology_opt	23	In silico: 10; Imaging dataset: 1; Bench: 10; Cadaver or phantom: 2	1 to 35
cmf	computational	vsp	17	In silico: 6; Imaging dataset: 2; Bench: 1; Cadaver or phantom: 8	1 to 160
cmf	imaging	mirroring	12	In silico: 1; Imaging dataset: 4; Bench: 5; Cadaver or phantom: 2	3 to 160
cmf	imaging	other	11	In silico: 3; Bench: 4; Cadaver or phantom: 4	1 to 40
cmf	imaging	protocol_effect	1	Cadaver or phantom: 1	10 to 10
cmf	imaging	registration	2	Cadaver or phantom: 2	10 to 36
cmf	imaging	segmentation_accuracy	3	In silico: 1; Imaging dataset: 1; Bench: 1	5 to 163
cmf	imaging	ssm	3	Imaging dataset: 3	19 to 35
cmf	imaging	template	2	Bench: 2	30 to 30
cranial	computational	automated_design	15	In silico: 2; Imaging dataset: 5; Bench: 4; Cadaver or phantom: 4	1 to 1930
cranial	computational	fea	27	In silico: 14; Imaging dataset: 1; Bench: 10; Cadaver or phantom: 2	1 to 144
cranial	computational	lattice_opt	4	In silico: 1; Bench: 3	1 to 6
cranial	computational	parametric_design	9	In silico: 4; Imaging dataset: 1; Bench: 4	1 to 30
cranial	computational	shape_opt	5	In silico: 5	1 to 6
cranial	computational	topology_opt	1	Bench: 1	.
cranial	computational	vsp	6	Bench: 2; Cadaver or phantom: 4	1 to 11
cranial	imaging	mirroring	9	In silico: 1; Imaging dataset: 1; Bench: 3; Cadaver or phantom: 4	1 to 20
cranial	imaging	other	5	Imaging dataset: 2; Bench: 1; Cadaver or phantom: 2	2 to 30
cranial	imaging	registration	7	Imaging dataset: 1; Bench: 1; Cadaver or phantom: 5	1 to 15
cranial	imaging	segmentation_accuracy	5	Imaging dataset: 3; Bench: 1; Cadaver or phantom: 1	23 to 23
cranial	imaging	template	3	Imaging dataset: 1; Bench: 1; Cadaver or phantom: 1	1 to 1
hip	computational	automated_design	3	Bench: 2; Cadaver or phantom: 1	1 to 1300
hip	computational	fea	19	In silico: 10; Bench: 8; Cadaver or phantom: 1	1 to 1300
hip	computational	lattice_opt	10	In silico: 3; Bench: 7	1 to 9
hip	computational	musculoskeletal_model	3	In silico: 2; Bench: 1	1 to 57
hip	computational	parametric_design	8	In silico: 3; Bench: 4; Cadaver or phantom: 1	1 to 1300
hip	computational	ssm_design	1	Imaging dataset: 1	.
hip	computational	topology_opt	4	In silico: 1; Bench: 3	1 to 9
hip	imaging	other	2	Bench: 2	1 to 1
hip	imaging	ssm	1	Imaging dataset: 1	.
knee	computational	automated_design	3	Imaging dataset: 2; Bench: 1	2 to 165
knee	computational	fea	30	In silico: 25; Bench: 4; Cadaver or phantom: 1	1 to 28
knee	computational	lattice_opt	2	Bench: 2	9 to 24
knee	computational	musculoskeletal_model	4	In silico: 4	4 to 28
knee	computational	parametric_design	15	In silico: 9; Imaging dataset: 2; Bench: 3; Cadaver or phantom: 1	1 to 673
knee	computational	ssm_design	2	Imaging dataset: 2	40 to 165
knee	computational	topology_opt	3	In silico: 2; Bench: 1	1 to 24
knee	computational	vsp	3	Imaging dataset: 1; Bench: 1; Cadaver or phantom: 1	6 to 19
knee	imaging	mirroring	1	In silico: 1	3 to 3
knee	imaging	other	4	In silico: 1; Bench: 2; Cadaver or phantom: 1	1 to 24
knee	imaging	registration	1	Cadaver or phantom: 1	6 to 6
knee	imaging	segmentation_accuracy	2	Imaging dataset: 2	40 to 165
knee	imaging	ssm	2	Imaging dataset: 2	40 to 165
lower_limb_other	computational	automated_design	2	Bench: 2	1 to 2
lower_limb_other	computational	fea	22	In silico: 15; Bench: 6; Cadaver or phantom: 1	1 to 720
lower_limb_other	computational	lattice_opt	6	Bench: 5; Cadaver or phantom: 1	1 to 6
lower_limb_other	computational	musculoskeletal_model	2	In silico: 2	1 to 1
lower_limb_other	computational	parametric_design	10	In silico: 5; Imaging dataset: 1; Bench: 4	1 to 140
lower_limb_other	computational	shape_opt	1	In silico: 1	720 to 720
lower_limb_other	computational	ssm_design	2	In silico: 1; Imaging dataset: 1	4 to 140
lower_limb_other	computational	topology_opt	5	In silico: 1; Bench: 4	1 to 6
lower_limb_other	computational	vsp	2	In silico: 1; Cadaver or phantom: 1	3 to 12
lower_limb_other	imaging	mirroring	1	Bench: 1	.
lower_limb_other	imaging	other	4	Bench: 3; Cadaver or phantom: 1	1 to 1
lower_limb_other	imaging	segmentation_accuracy	1	Imaging dataset: 1	.
multi	computational	automated_design	1	Imaging dataset: 1	71 to 71
multi	computational	fea	3	In silico: 1; Bench: 2	2 to 60
multi	computational	lattice_opt	1	In silico: 1	6 to 6
multi	imaging	mirroring	1	Cadaver or phantom: 1	3 to 3
other	computational	fea	5	In silico: 3; Bench: 1; Cadaver or phantom: 1	80 to 80
other	computational	parametric_design	2	In silico: 1; Bench: 1	80 to 80
other	computational	shape_opt	1	Bench: 1	.
other	computational	vsp	1	In silico: 1	.
other	imaging	other	1	Bench: 1	.
pelvis_acetabulum	computational	automated_design	1	In silico: 1	15 to 15
pelvis_acetabulum	computational	fea	25	In silico: 19; Bench: 6	1 to 10
pelvis_acetabulum	computational	lattice_opt	2	In silico: 1; Bench: 1	2 to 2
pelvis_acetabulum	computational	musculoskeletal_model	2	In silico: 2	1 to 1
pelvis_acetabulum	computational	parametric_design	5	In silico: 4; Bench: 1	1 to 6
pelvis_acetabulum	computational	ssm_design	2	Imaging dataset: 2	90 to 90
pelvis_acetabulum	computational	topology_opt	5	In silico: 4; Bench: 1	1 to 3
pelvis_acetabulum	computational	vsp	5	Imaging dataset: 2; Bench: 1; Cadaver or phantom: 2	10 to 111
pelvis_acetabulum	imaging	mirroring	1	Imaging dataset: 1	13 to 13
pelvis_acetabulum	imaging	other	3	In silico: 2; Imaging dataset: 1	1 to 13
pelvis_acetabulum	imaging	registration	3	Imaging dataset: 1; Cadaver or phantom: 2	10 to 111
pelvis_acetabulum	imaging	ssm	2	Imaging dataset: 2	90 to 90
shoulder	computational	fea	4	In silico: 3; Bench: 1	1 to 19
shoulder	computational	parametric_design	1	In silico: 1	19 to 19
shoulder	computational	vsp	1	Cadaver or phantom: 1	10 to 10
shoulder	imaging	other	1	In silico: 1	2 to 2
spine	computational	automated_design	6	In silico: 4; Cadaver or phantom: 2	7 to 1080
spine	computational	fea	26	In silico: 20; Bench: 4; Cadaver or phantom: 2	1 to 24
spine	computational	lattice_opt	5	In silico: 3; Bench: 1; Cadaver or phantom: 1	4 to 24
spine	computational	parametric_design	10	In silico: 7; Bench: 2; Cadaver or phantom: 1	1 to 1080
spine	computational	shape_opt	2	In silico: 2	3 to 1080
spine	computational	ssm_design	1	In silico: 1	10 to 10
spine	computational	topology_opt	5	In silico: 2; Bench: 2; Cadaver or phantom: 1	2 to 16
spine	computational	vsp	1	Cadaver or phantom: 1	5 to 5
spine	imaging	other	4	Bench: 1; Cadaver or phantom: 3	3 to 18
spine	imaging	ssm	1	In silico: 1	10 to 10
upper_limb_other	computational	automated_design	1	In silico: 1	6 to 6
upper_limb_other	computational	fea	10	In silico: 6; Bench: 1; Cadaver or phantom: 3	1 to 19
upper_limb_other	computational	musculoskeletal_model	1	Bench: 1	1 to 1
upper_limb_other	computational	parametric_design	4	In silico: 2; Cadaver or phantom: 2	1 to 19
upper_limb_other	computational	shape_opt	2	In silico: 1; Bench: 1	1 to 6
upper_limb_other	computational	ssm_design	1	Cadaver or phantom: 1	2 to 2
upper_limb_other	computational	vsp	2	Cadaver or phantom: 2	5 to 20
upper_limb_other	imaging	mirroring	2	Cadaver or phantom: 2	2 to 11
upper_limb_other	imaging	other	2	Cadaver or phantom: 2	5 to 20
upper_limb_other	imaging	protocol_effect	3	Imaging dataset: 1; Cadaver or phantom: 2	20 to 20
upper_limb_other	imaging	registration	3	In silico: 1; Cadaver or phantom: 2	6 to 20
upper_limb_other	imaging	segmentation_accuracy	2	Imaging dataset: 1; Cadaver or phantom: 1	20 to 20
upper_limb_other	imaging	ssm	1	Cadaver or phantom: 1	2 to 2

cmf, craniomaxillofacial; fea, finite element analysis; lattice_opt, shape_opt and topology_opt, lattice, shape and topology optimisation; multi, multi-region; protocol_effect, effect of the imaging protocol or modality on the implant; ssm, statistical shape model; ssm_design, design driven by a statistical shape model; vsp, virtual surgical planning. Region and method labels are the charting vocabulary defined in Text S4.

**Table 4:** Randomised trials and comparative clinical studies (non-comparative series are listed in Table S1; a clinical-result cell without figures reproduces the authors’ qualitative statement and is not an effect estimate).

Study	Design	Region	Implant Type	Pillars	Patients	Comparator Type	Clinical Result	Follow-Up
Raisian et al. (2017) [62]	RCT	cmf	orbital titanium mesh	imaging	10	manual_design	preoperative vertical dystopia or diplopia resolved in 2/5 intervention patients	4 months
Schneider et al. (2019) [63]	RCT	cmf	pre-bent orthognathic fixation plate	comp	21	conventional_implant	.	.
Li et al. (2021) [64]	RCT	cmf	maxillary osteosynthesis plate	comp	58	alternative_method	infection 1/27 vs. 1/31; no significant difference in serious adverse events	within 1 week
Sánchez-Jáuregui et al. (2022) [65]	RCT	cmf	maxillary osteosynthesis plate	comp	30	alternative_method	.	.
Van der Wel et al. (2023) [66]	RCT	cmf	maxillary osteosynthesis plate	comp	58	conventional_implant	.	1 year
Bevini et al. (2025) [67]	RCT	cmf	orthognathic fixation implant	comp	20	alternative_method	no surgical complications [authors’ qualitative statement; no figures]	1 month
Sheta et al. (2025) [68]	RCT	cmf	orbital wall implant	imaging + comp	28	conventional_implant	residual enophthalmos 1/14 vs. 3/14; residual diplopia 0/14 vs. 1/14	3 months
Alshikh et al. (2026) [69]	RCT	cmf	maxillary ridge augmentation mesh	comp	14	conventional_implant	gained bone volume 499.47 ± 80.46 mm^3^ PEEK vs. 370.82 ± 51.69 mm^3^ titanium (*p* = 0.004); graft loss 174.83 ± 40.78 mm^3^ vs. 127.73 ± 35.55 mm^3^ (*p* = 0.040); mesh exposure 1/14 sites vs. 2/14 sites	6 months
El Morsy et al. (2026) [70]	RCT	cmf	orbital floor implant	imaging + comp	24	alternative_method	complications: 3 in titanium group and 0 in zirconia group	.
Abdellatif et al. (2026) [71]	RCT	cmf	mandibular fracture fixation plates	imaging + comp	26	conventional_implant	wound dehiscence 0/13 vs. 1/13; immediate occlusal disharmony 0/13 vs. 2/13	4 weeks
Maini et al. (2018) [72]	RCT	pelvis_acetabulum	acetabular fixation plate	comp	25	conventional_implant	total blood loss lower than conventional contouring [authors’ qualitative statement; no figures]	.
Shuang et al. (2016) [73]	RCT	upper_limb_other	custom osteosynthesis plate	imaging + comp	13	conventional_implant	bone union 13/13; good or excellent elbow function 83.1% vs. 71.4%; no wound infections	10.6 months mean (range 6–13 months)
Ciocca et al. (2015) [74]	Comparative	cmf	mandibular reconstruction plate	comp	10	conventional_implant	.	.
Tarsitano et al. (2016) [75]	Comparative	cmf	customised mandibular reconstruction plate	imaging + comp	30	conventional_implant	.	.
Tarsitano et al. (2016) [76]	Comparative	cmf	mandibular reconstruction plate	comp	40	conventional_implant	microvascular complications 0/20 vs. 2/20; mean hospital stay 13.8 vs. 17 days	.
Zimmerer et al. (2016) [77]	Comparative	cmf	orbital reconstruction implant	comp	195	conventional_implant	individualisation and navigation provided clinical benefit [authors’ qualitative statement; no figures]	12 weeks
De Maesschalck et al. (2017) [78]	Comparative	cmf	mandibular reconstruction plate	imaging + comp	18	conventional_implant	CAS and freehand reconstruction were morphologically comparable except nonaffected-side axial angle (1.0° vs. 2.9°; *p* = 0.03)	.
Kim et al. (2017) [79]	Comparative	cmf	pre-bent orbital-wall mesh	imaging	82	conventional_implant	.	.
Fan et al. (2017) [80]	Comparative	cmf	orbital Medpor-titanium implant	imaging	56	manual_design	postoperative clinical evaluation superior to control [authors’ qualitative statement; no figures]	.
Kim et al. (2018) [81]	Comparative	cmf	orbital floor and medial wall implant	imaging	28	alternative_method	diplopia improved in 9/10 patients; moderate-to-severe enophthalmos improved in 18/18 patients	6 months
Rückschloß et al. (2019) [82]	Comparative	cmf	maxillary patient-specific fixation implant	comp	18	alternative_method	.	.
Kim et al. (2019) [83]	Comparative	cmf	orbital wall implant	imaging + comp	56	alternative_method	.	.
Liu et al. (2020) [84]	Comparative	cmf	maxillary osteosynthesis plate	comp	18	alternative_method	.	.
Sigron et al. (2020) [85]	Comparative	cmf	hybrid orbital floor mesh	imaging + comp	22	conventional_implant	postoperative stay 4.6 (3.9) d vs. 3.8 (3.0) d; no significant difference	.
Lee et al. (2020) [86]	Comparative	cmf	mandibular reconstruction plate	comp	126	conventional_implant	hardware complications 5.5% vs. 15.5%; *p* = 0.091; customised plates independently predicted fewer overall complications *p* = 0.03	23.5 months mean
Badiali et al. (2020) [87]	Comparative	cmf	mandibular osteosynthesis plate	comp	22	alternative_method	system successful in 22/22 patients; outcome clinically satisfactory in 22/22	14 months average (range 5–29 months)
Rahimov et al. (2020) [88]	Comparative	cmf	customised orbital plate	imaging + comp	77	conventional_implant	residual diplopia 4/42 (9.5%) vs. 12/35 (34.3%) at 6 months	6 months
Yang et al. (2021) [89]	Comparative	cmf	maxillary or mandibular reconstruction plate	comp	33	conventional_implant	plate removal 0/17 vs. 2/16; no major postoperative adverse events in either group	CT follow-up 1.8 ± 0.8 months vs. 17.3 ± 11.2 months
Olate et al. (2021) [90]	Comparative	cmf	mandibular angle contour implant	comp	21	conventional_implant	infections in both groups; *p* > 0.05	6 months
Karanxha et al. (2021) [91]	Comparative	cmf	maxillary and mandibular fixation plates	comp	16	alternative_method	.	.
Neuhaus et al. (2021) [92]	Comparative	cmf	patient-specific total TMJ prosthesis	imaging + comp	21	alternative_method	mouth opening 21.69 ± 2.80 mm preop to 36.40 ± 1.25 mm; pain 6.18 ± 0.74 to 1.06 ± 0.52	15.3 ± 3.0 months mean
Knitschke et al. (2021) [93]	Comparative	cmf	custom osteosynthesis plate	comp	104	conventional_implant	bone R0 margins 95.1% VSP vs. 96.8% non-VSP (*p* = 0.516); soft-tissue R0 margins 63.4% vs. 73.0% (*p* = 0.470)	.
Sigron et al. (2021) [94]	Comparative	cmf	hybrid patient-specific orbital floor mesh	imaging + comp	30	conventional_implant	complete diplopia resolution 9/12 vs. 5/7; no major complications	6 months mean (range 1 day to almost 2 years)
Kang et al. (2021) [95]	Comparative	cmf	pre-bent midface reconstruction plate	imaging + comp	34	conventional_implant	dental implantation 50.0% vs. 11.1%; all patients satisfied with facial symmetry	.
Möllmann et al. (2021) [96]	Comparative	cmf	mandibular reconstruction plate	comp	94	conventional_implant	complications 33/46 PSI vs. 31/48 CR; no significant difference	.
Harding et al. (2022) [97]	Comparative	cmf	custom mandibular osteosynthesis plate	comp	55	conventional_implant	.	.
Timoshchuk et al. (2022) [98]	Comparative	cmf	orbital reconstruction implant	comp	85	conventional_implant	complications 8.3% vs. 26.2% (*p* = 0.08)	.
Jones et al. (2022) [99]	Comparative	cmf	maxillary osteosynthesis plate	comp	82	alternative_method	.	.
Liokatis et al. (2022) [100]	Comparative	cmf	midfacial osteosynthesis implant	imaging + comp	8	alternative_method	no major complications; plate removal 2/8	13–79 months
Rodríguez-Arias et al. (2022) [101]	Comparative	cmf	mandibular reconstruction plate	comp	37	conventional_implant	5% fewer complications with CAD/CAM; flap survival 100%	.
Chepurnyi et al. (2022) [102]	Comparative	cmf	orbital wall patient-specific implant	imaging + comp	50	manual_design	no implant malposition or enophthalmos; diplopia 13.0% vs. 11.1% at 3 months	3 months
Cassoni et al. (2022) [103]	Comparative	cmf	maxillary fixation plate	imaging + comp	10	conventional_implant	.	1 month
Spalthoff et al. (2022) [104]	Comparative	cmf	mandibular reconstruction plate	imaging + comp	83	alternative_method	surgery postponed in 16/83; 15/16 postponed cases were complex, *p* = 0.001	.
Kerkfeld et al. (2022) [105]	Comparative	cmf	orthognathic plates and facial augmentation implants	imaging + comp	30	alternative_method	PEEK implant exposure 2/8; no other PEEK complications	at least 9 months
Knitschke et al. (2022) [106]	Comparative	cmf	jaw reconstruction plate	comp	133	conventional_implant	incomplete osseous union 26.6% PSI vs. 8.7% non-PSI at patient level; adjusted OR 3.518 (95% CI 1.223–10.124), *p* = 0.02	54 months mean to final scan (range 2–216)
Landau Prat et al. (2023) [107]	Comparative	cmf	orbital floor implant	imaging	27	conventional_implant	primary or vertical gaze diplopia improved in both groups (*p* = 0.03)	1.1 years mean
Kim et al. (2023) [108]	Comparative	cmf	orthognathic fixation plate	comp	12	conventional_implant	plate exposure requiring replacement 1/6 customised patients	6 months
Pietzka et al. (2023) [109]	Comparative	cmf	maxillary osteosynthesis plate	comp	126	conventional_implant	dental root injuries 10/651 screws in conventional cohort vs. 0/773 PSI screws (*p* < 0.001)	13 months mean
Pietzka et al. (2023) [110]	Comparative	cmf	orbital implant	imaging + comp	75	conventional_implant	intraoperative revision 26.6% vs. 13%; postoperative revision 11.1% vs. 0% PSI	.
Kuehle et al. (2023) [111]	Comparative	cmf	maxillary osteosynthesis plate	imaging + comp	50	conventional_implant	.	first postoperative week
Block et al. (2024) [112]	Comparative	cmf	mandibular reconstruction plate	imaging + comp	29	alternative_method	.	.
Merta et al. (2024) [113]	Comparative	cmf	mandibular osteotomy fixation plate	comp	53	conventional_implant	no difference in skeletal stability between VSP-PSI and conventional mini-plate groups [authors’ qualitative statement; no figures]	.
Consorti et al. (2024) [114]	Comparative	cmf	customised orbital implant	imaging + comp	52	conventional_implant	clinical improvements and complications similar between groups [authors’ qualitative statement; no figures]	.
Krasovsky et al. (2024) [115]	Comparative	cmf	orbital reconstruction implant	imaging + comp	23	conventional_implant	.	.
D’Alpaos et al. (2024) [116]	Comparative	cmf	orbital floor implant	imaging + comp	23	conventional_implant	no implant-related complications or reoperations in 13 PSI patients	30.69 months mean PSI; 25.30 months mean control
Ettinger et al. (2024) [117]	Comparative	cmf	mandibular reconstruction plate	comp	130	conventional_implant	.	.
Yildirim et al. (2026) [118]	Comparative	cmf	bimaxillary orthognathic fixation implant	comp	46	alternative_method	no conversion to splint-based transfer 0/46	≤4 weeks
Le et al. (2025) [119]	Comparative	cmf	mandibular reconstruction plate	comp	18	conventional_implant	.	.
Mohamed et al. (2025) [120]	Comparative	cmf	mandibular reconstruction plate	comp	265	conventional_implant	hardware-related complications in 72/265; stock plates vs. patient-specific implants HR 1.80, 95% CI 1.11–2.91, *p* = 0.0171	≥1 year
Chen et al. (2025) [121]	Comparative	cmf	absorbable orbital reconstruction plate	imaging + comp	94	manual_design	no infection, implant displacement, or vision deterioration; superior diplopia resolution *p* = 0.023 and ocular mobility restoration *p* = 0.049	6–11 months
Ren et al. (2025) [122]	Comparative	cmf	absorbable orbital wall plate	imaging + comp	106	conventional_implant	residual diplopia 9.2% vs. 31.6%; no implant-related infection or displacement in absorbable group	6 months minimum
Ismailov et al. (2026) [123]	Comparative	cmf	pre-contoured mandibular reconstruction plate	comp	12	other	hardware destabilisation 1/12	.
Zoabi et al. (2026) [124]	Comparative	cmf	orbital floor implant	imaging + comp	23	alternative_method	orbital volume 108.6% to 99.5% with mirroring and 107.3% to 101.1% with virtual reduction; diplopia resolved in most cases	.
Adell-Gómez et al. (2026) [125]	Comparative	cmf	orthognathic fixation plates	imaging + comp	90	conventional_implant	mandibular plates replaced intraoperatively in 1/15 PSI patients	.
Troise et al. (2026) [126]	Comparative	cmf	orbital floor mesh	comp	52	conventional_implant	persistent diplopia 1/24 (4%) vs. 8/28 (28.6%); *p* = 0.028	12 months
Yang et al. (2026) [127]	Comparative	cmf	mandibular reconstruction plate	imaging + comp	111	conventional_implant	plate-related complications 43/110; plate exposure 24/110	59.8 months mean
Mäntynen et al. (2026) [128]	Comparative	cmf	orthognathic fixation plate	imaging + comp	40	alternative_method	reoperation for postoperative malocclusion 1/40	.
Prikule et al. (2026) [129]	Comparative	cmf	orbital floor mesh	imaging + comp	74	conventional_implant	reoperation 5/34 control vs. 0/40 3D, *p* = 0.017; persistent diplopia 4/34 vs. 1/40 at 6 months	6 months
Aguilar et al. (2026) [130]	Comparative	cmf	customised maxillary fixation plate	comp	64	alternative_method	instability 28.6% minimally invasive vs. 33.3% conventional; OR 1.23, 95% CI 0.32–4.65	6 months
Rouhiainen et al. (2026) [131]	Comparative	cmf	maxillary fixation plate	comp	59	conventional_implant	.	12.4–13.2 months median
De Oliveira Gorla et al. (2026) [132]	Comparative	cmf	orbital implant	comp	10	alternative_method	clinical success 6/9; persistent diplopia 3/9	.
Bertram et al. (2026) [133]	Comparative	cmf	patient-specific maxillary osteosynthesis plate	imaging + comp	26	conventional_implant	.	.
Desai et al. (2019) [134]	Comparative	cranial	cranioplasty implant	comp	30	alternative_method	infection 13.3%; patient satisfaction excellent in 69%; 1 resurgery in alginate-impression group	.
Baldia et al. (2022) [135]	Comparative	cranial	cranioplasty prosthesis	imaging	70	alternative_method	3D-printed prostheses had better cosmetic scores than hand-moulded and bone-impression prostheses, *p* < 0.001	.
Kim et al. (2023) [136]	Comparative	cranial	cranioplasty implant	imaging	35	conventional_implant	complications 2/12 vs. 6/23; reoperation 2/12 vs. 5/23	.
Kim et al. (2026) [137]	Comparative	cranial	cranioplasty flap	imaging	36	conventional_implant	CSF collection 0/16 modified vs. 2/20 control; haemorrhage 0/16 vs. 2/20 control	1 month
Zhao et al. (2024) [138]	Comparative	hip	custom hip joint prosthesis	comp	33	conventional_implant	33/33 had no infection, deep vein thrombosis, or wound nonunion; implants stable without loosening or osteolysis at 6 months	6 months
Hu et al. (2022) [139]	Comparative	knee	strut-type bone-defect prosthesis	comp	10	conventional_implant	MSTS 28.4 ± 1.8 versus 26.2 ± 2.8, *p* = 0.176; all 5 prosthesis patients achieved bone graft fusion	24.7 ± 2.4 months mean
Liu et al. (2019) [140]	Comparative	pelvis_acetabulum	pelvic reconstruction implant	comp	38	alternative_method	lower tumour recurrence but higher implant loosening than common 3D-printed template	.
Pu et al. (2021) [141]	Comparative	pelvis_acetabulum	sacroiliac reconstruction prosthesis	comp	24	conventional_implant	MSTS-93 24.1 ± 2.8 vs. 18.9 ± 2.6; complications 1/12 vs. 1/12; disease-free survival 9/12 vs. 9/12	21 months median for 3D-printing and 20 months median for control; range 6–90 months
Hu et al. (2024) [142]	Comparative	pelvis_acetabulum	hemipelvic endoprosthesis	comp	32	conventional_implant	MSTS 24.3 ± 1.8 vs. 21.8 ± 2.0; HHS 79.8 ± 5.2 vs. 75.3 ± 3.5	41.6 months mean
Zhang et al. (2024) [143]	Comparative	pelvis_acetabulum	acetabular augment/triflange cup	comp	109	conventional_implant	.	6.0 ± 1.3 years augment; 2.8 ± 0.6 years triflange
Huang et al. (2025) [144]	Comparative	pelvis_acetabulum	hemipelvic prosthesis	imaging + comp	28	alternative_method	implant-related complications: breakage 4/28, aseptic loosening 4/28, dislocation 2/28; MSTS-93 18.2 ± 5.3	36.2 months mean
Guo et al. (2022) [145]	Comparative	shoulder	scapular fixation plate	comp	21	conventional_implant	no complications in 21/21; follow-up functional outcome similar between groups	.
Lv et al. (2025) [146]	Comparative	spine	artificial vertebral body	comp	21	conventional_implant	2-yr VAS 0.7 ± 0.6 vs. 1.8 ± 0.6; fusion 100% vs. 38.9%; rod fracture 0/3 vs. 6/18	38.5 months mean
Van Doremalen et al. (2021) [147]	Comparative	upper_limb_other	contoured clavicle fixation plate	imaging + comp	14	conventional_implant	discomfort-related implant removal 0/6 vs. 5/7; *p* = 0.012; infection 1/7	4 years minimum
Gong et al. (2023) [148]	Comparative	upper_limb_other	distal radius tumour endoprosthesis	comp	22	alternative_method	subluxation rate significantly lower with 0° than biological-angle design	60 months median (range 19–82)

cmf, craniomaxillofacial; comp, computational planning; imaging, evaluated imaging step; CAD/CAM, computer-aided design and manufacturing; CAS, computer-assisted surgery; CI, confidence interval; CR, conventional reconstruction plate; CSF, cerebrospinal fluid; CT, computed tomography; HHS, Harris Hip Score; HR, hazard ratio; MSTS, Musculoskeletal Tumor Society score; OR, odds ratio; PEEK, polyetheretherketone; PSI, patient-specific implant; R0, microscopically clear resection margin; RCT, randomised controlled trial; TMJ, temporomandibular joint; VAS, visual analogue scale; VSP, virtual surgical planning. Values are reproduced as reported by the original authors. Region and comparator labels (e.g., pelvis_acetabulum, conventional_implant) are the charting vocabulary defined in Text S4; a full stop (.) indicates not reported.

**Table 5:** Prespecified meta-analysis test (amendment A8): outcomes reported for both arms against a conventional comparator.

Outcome	Studies with Extractable Estimates	Pooled Estimate (95% CI)	95% Prediction Interval	I-Squared	Sensitivity (HKSJ CI; Leave-One-Out Range; Small-Study Test)	Disposition
accuracy	7 (RCT 1, comparative 6)	−0.72 (−1.32 to −0.13), standardised mean difference (Hedges g)	−2.6 to 1.16	76%	HKSJ −1.42 to −0.03; leave-one-out −0.94 to −0.53; small-study test not performed (fewer than 10 studies)	pooled; 9 included studies reported the outcome for both arms; 7 against a comparator charted as a conventional implant and measured as deviation of the delivered result from the plan
complications any	8 (RCT 1, comparative 7)	1.05 (0.8 to 1.36), risk ratio (pooled on the log scale)	0.75 to 1.45	0%	HKSJ 0.8 to 1.37; leave-one-out 0.63 to 1.11; small-study test not performed (fewer than 10 studies)	pooled; 9 included studies reported the outcome for both arms; 8 against a comparator charted as a conventional implant
infection	6 (RCT 2, comparative 4)	0.94 (0.3 to 2.99), risk ratio (pooled on the log scale)	0.18 to 4.85	0%	HKSJ 0.51 to 1.74; leave-one-out 0.77 to 1.39; small-study test not performed (fewer than 10 studies)	pooled; 8 included studies reported the outcome for both arms; 6 against a comparator charted as a conventional implant
operative time	11 (RCT 1, comparative 10)	−11.8 (−30.5 to 6.9), mean difference (minutes)	−74.6 to 51.0	93%	HKSJ −46.3 to 22.7; leave-one-out −24.0 to −7.3; Egger intercept −1.26, *p* = 0.601	pooled; 14 included studies reported the outcome for both arms; 13 against a comparator charted as a conventional implant
revision or removal	8 (RCT 0, comparative 8)	0.28 (0.11 to 0.72), risk ratio (pooled on the log scale)	0.09 to 0.91	0%	HKSJ 0.12 to 0.66; leave-one-out 0.22 to 0.33; small-study test not performed (fewer than 10 studies)	pooled; 9 included studies reported the outcome for both arms; 8 against a comparator charted as a conventional implant
blood loss	.	not pooled	.	.	.	5 included studies reported the outcome for both arms; 4 against a comparator charted as a conventional implant; 0 with extractable estimates against a conventional comparator, below the prespecified minimum of 3
function	.	not pooled	.	.	.	4 included studies reported the outcome for both arms; 4 against a comparator charted as a conventional implant; 0 with extractable estimates against a conventional comparator, below the prespecified minimum of 3

CI, confidence interval; HKSJ, Hartung–Knapp–Sidik–Jonkman; I-squared, percentage of variability attributable to between-study heterogeneity; RCT, randomised controlled trial. Mean differences and standardised mean differences (Hedges g) below 0, and risk ratios below 1, favour the patient-specific implant. The accuracy row is the prespecified seven-study pool; the primary estimate, restricted to the four studies that measure deviation from the virtual plan with smaller meaning better, is −1.21 (95% CI −1.69 to −0.74; I-squared 0%; Section 3.6 and Text S6). A full stop (.) indicates not applicable.

**Table 6:** Risk of bias of the clinical-tier studies.

Tool (Tier)	Studies Appraised	Overall Judgement Distribution
RoB 2 (randomised trials)	12	high: 2; some concerns: 10
ROBINS-I (comparative non-randomised)	75	low: 2; moderate: 4; serious: 69
JBI case series (non-comparative series)	179	items met, median 5 of 10; 5 or fewer items met: 100; 8 or more: 35; distribution 0/10: 3; 1/10: 18; 2/10: 32; 3/10: 18; 4/10: 17; 5/10: 12; 6/10: 19; 7/10: 25; 8/10: 20; 9/10: 9; 10/10: 6

**Table 7:** Certainty of evidence per clinical outcome (GRADE domains; the comparison is the delivered patient-specific implant against a conventional comparator).

Outcome	Studies (Participants)	Effect	Risk of Bias	Inconsistency	Indirectness	Imprecision	Publication Bias	Certainty
Deviation of the delivered implant from the preoperative plan (patient-specific versus conventional)	4 (81)	−1.21 (−1.69 to −0.74); prediction −2.26 to −0.17; standardised mean difference (Hedges g)	serious: the three comparative studies at serious risk under ROBINS-I; the trial at some concerns under RoB 2	not serious: I-squared 0% once the pool is restricted to commensurable measures; surface and landmark measures still differ across studies	serious: the comparison evaluates the implant as delivered, not a design method; accuracy measures differ across studies	serious: four small studies with 81 participants in total	not assessed (fewer than ten studies; small-study tests underpowered)	Very low
Operative time (patient-specific versus conventional)	11 (384)	−11.8 (−30.5 to 6.9); prediction −74.6 to 51.0; mean difference (minutes)	serious: confounding by period, surgeon and case complexity	very serious: I-squared about 90%, prediction interval spans large savings and large excesses; direction varies within and between regions	serious: implant as delivered, not design method	serious: confidence interval includes no difference	Egger test reported in Table 5 (ten or more studies); no downgrade applied because the test is weak with this heterogeneity	Very low
Any complication (patient-specific versus conventional)	8 (242)	1.05 (0.8 to 1.36); prediction 0.75 to 1.45; risk ratio (pooled on the log scale)	serious: confounding by period, surgeon and case complexity; selective reporting of events	not serious statistically (I-squared 0%) but event definitions differ	serious: implant as delivered, not design method	serious: interval includes both benefit and harm	not assessed (fewer than ten studies)	Very low
Infection (patient-specific versus conventional)	6 (133)	0.94 (0.3 to 2.99); prediction 0.18 to 4.85; risk ratio (pooled on the log scale)	serious: confounding by period, surgeon and case complexity; selective reporting of events	not serious statistically (I-squared 0%) but event definitions differ	serious: implant as delivered, not design method	very serious: few events, interval from a third to three times	not assessed (fewer than ten studies)	Very low
Revision or removal of the implant (patient-specific versus conventional)	8 (284)	0.28 (0.11 to 0.72); prediction 0.09 to 0.91; risk ratio (pooled on the log scale)	serious: confounding by period, surgeon and case complexity; selective reporting of events	not serious statistically (I-squared 0%) but event definitions differ	serious: implant as delivered, not design method	serious: few events; interval excludes no effect but rests on eight non-randomised studies	not assessed (fewer than ten studies)	Very low
Clinical outcomes of computationally designed implants outside the craniomaxillofacial region (cranial, pelvic, limb and spinal)	mostly non-comparative series; a minority of comparative studies and two randomised trials (pelvis, distal humerus), no pooled outcome	not pooled	serious: comparative studies at serious risk from confounding; series rarely report consecutive or complete inclusion	not estimable (no pooled outcome)	serious	very serious	not assessed	Very low

## Data Availability

The data presented in this study are available in the article and its Supplementary Materials. The search code, raw search responses, screening and charting outputs, instruction files, adjudicated corrections, analysis code, verification suite and the adversarial review reports are available on request from the corresponding author.

## Supplementary Materials

The following supporting information can be downloaded at https://www.mdpi.com/article/10.3390/bioengineering13101149/s1, Text S1: Extended methods: search, selection, retrieval, charting agreement and corrections; Text S2: Prospective protocol with dated amendments; Text S3: Full search strategies (PRISMA-S); Text S4: Instructions given to the AI screeners, charters and assessors; Text S5: Contemporaneous log of AI use; Text S6: Adjudication of the plan-deviation accuracy pool; Table S1: All included studies; Table S2: Studies excluded at assessment; Table S3: Technical credibility items; Table S4: Risk of bias per clinical-tier study; Table S5: Funding, conflict of interest and industry authorship; Table S6: Outcome-by-study disposition for the meta-analysis test; Table S7: Artificial intelligence studies: evaluation data, leakage risk and prediction-model reporting. References [13,15,16,17,19,20,21,22,23,24,25,71,89,96,103,108,111,119] are cited in the Supplementary Materials.

## Abbreviations

The following abbreviations are used in this manuscript:

AI	Artificial intelligence
API	Application programming interface
CAD/CAM	Computer-aided design/computer-aided manufacturing
CI	Confidence interval
CINAHL	Cumulative Index to Nursing and Allied Health Literature
CLAIM	Checklist for Artificial Intelligence in Medical Imaging
CLI	Command-line interface
CNN	Convolutional neural network
CSF	Cerebrospinal fluid
CT	Computed tomography
DOI	Digital object identifier
DSC	Dice similarity coefficient
GPT	Generative pre-trained transformer
GRADE	Grading of Recommendations Assessment, Development and Evaluation
HD95	95th-percentile Hausdorff distance
HHS	Harris Hip Score
HKSJ	Hartung–Knapp–Sidik–Jonkman (confidence-interval method)
HR	Hazard ratio
JBI	Joanna Briggs Institute
MSTS/MSTS-93	Musculoskeletal Tumor Society (rating system), 1993 revision
NCBI	National Center for Biotechnology Information
OSF	Open Science Framework
PEEK	Polyether ether ketone
PRISMA	Preferred Reporting Items for Systematic Reviews and Meta-Analyses
PRISMA-S	PRISMA extension for reporting literature searches
PROSPERO	International Prospective Register of Systematic Reviews
PSI	Patient-specific implant
R0	Clear (microscopically negative) resection margin
RCT	Randomised controlled trial
RMSE	Root mean square error
RoB 2	Cochrane Risk-of-Bias tool, version 2 (randomised trials)
ROBINS-I	Risk Of Bias In Non-randomised Studies—of Interventions
SD	Standard deviation
SSM	Statistical shape model(ling)
SWiM	Synthesis Without Meta-analysis
TMJ	Temporomandibular joint
TRIPOD + AI	Transparent Reporting of a multivariable prediction model for Individual Prognosis Or Diagnosis + Artificial Intelligence
VAS	Visual analogue scale
VSP	Virtual surgical planning
