# Supplementary material for: Patient-Specific Implant Design: Current Evidence for Artificial Intelligence, Imaging and Computational Planning—A Systematic Review of the Biomedical-Indexed Literature
Source: Bioengineering (Basel). 2026 Oct 1;13(10):1149. doi: 10.3390/bioengineering13101149 (PMC13701698; doi:10.3390/bioengineering13101149)
Supplement: Supplementary file 1 [file bioengineering-13-01149-s001.zip › bioengineering-4586457 - MDPI --prisma.pdf]

PRISMA 2020 flow and reporting checklists

PRISMA 2020 flow

Identification from databases

- Records identified: 7772
    - Crossref (bounded sweep): 2000
    - Europe PMC: 1687
    - MEDLINE via PubMed: 3207
    - OpenAlex (3 Sept run): 878
- Duplicate records removed: 2713
- Records screened (unique): 5059
- Excluded by explicit rule: 2682
    - i_no_individualisation_term: 1850
    - ii_no_device_term: 182
    - iii_non_human_signal: 40
    - iv_review_or_editorial_type: 410
    - v_case_report_type: 200
- Records entering dual AI title and abstract triage: 2377
- Excluded by both independent screeners: 1453
- Retained after triage: 924

Identification through other methods (citation chasing)

- Reviews chased: 32
- References seen: 836
- New records after deduplication and date restriction: 728
- Excluded by explicit rule: 584
    - i_no_individualisation_term: 234
    - ii_no_device_term: 57
    - iii_non_human_signal: 2
    - iv_review_or_editorial_type: 39
    - pre_2015: 238
    - v_case_report_type: 14
- Passed the rule layer (records screened by dual AI): 144
- Excluded by both independent screeners: 89
- Retained after triage: 55

Assessment

- Reports sought for retrieval: 979
    - Retained from databases: 924
    - Retained from citation chasing: 55
- Reports not retrieved as full text (assessed from the abstract): 494
- Full reports retrieved: 425
- Reports assessed for eligibility: 979
    - Full-report route: 425
    - Abstract-only route: 494
    - Neither abstract nor retrievable full text (title only): 60
        - held unresolved: 47

        - excluded as duplicate copies of an assessed report: 13
- Reports excluded after assessment: 273
    - case_report: 30
    - duplicate_preprint: 25
    - duplicate report (same article under a second identifier): 35
    - no_evaluation: 17
    - no_method: 31
    - non_human: 10
    - not_psi: 107
    - not_skeletal: 3
    - source_type: 15
- Unresolved after assessment: 67
    - of which had no abstract and no retrievable text: 47
    - of which had text that did not meet eligibility: 20
- Studies included: 639

PRISMA 2020 checklist

Locators cite manuscript sections (the workspace convention uses no margin line numbers). Departures are named, not hidden. Item wording is abbreviated; confirm the current checklist at equator-network.org at submission.

#	Item	Where / status
1	Title identifies the report as a systematic review	Title
2	Abstract per PRISMA 2020 for Abstracts	Abstract (search date, sources, screening method, limitations and registration status stated there and in 2.1, 2.3, 2.4)
3	Rationale	Introduction
4	Objectives	Introduction, final paragraph; protocol section 2 (Text S2)
5	Eligibility criteria	2.2; protocol section 3
6	Information sources with dates	2.3 (PubMed, Europe PMC, Crossref searched 2 September 2026; OpenAlex 3 September 2026 as a supplementary run, amendments A1 and A9; registries and citation chasing 2 September)
7	Full search strategies	Text S3 (verbatim strings, hit and retrieval counts, per PRISMA-S); summarised in 2.3
8	Selection process	2.4 (rule layer with published reasons; dual independent AI screeners from different vendors, recall-union; sampled rule-layer audit; full-record assessment) **Departure:** PRISMA expects two independent human reviewers; screening is AI-performed with published per-record reasons
9	Data collection process	2.5 (AI charter from full text where retrievable, otherwise abstract, text source recorded; independent second charter on a seeded sample with agreement reported); same departure as item 8
10	Data items	2.5; charting instructions in Text S4
11	Risk of bias assessment	2.6 (RoB 2, ROBINS-I, JBI case series for clinical tiers; six-item credibility set for technical tiers)
12	Effect measures	2.7 (mean difference in minutes for operative time; standardised mean difference for plan deviation; risk ratios for event outcomes; headline results otherwise in their own units)
13	Synthesis methods	2.7 (grouping, ordering, result summary rules, heterogeneity exploration; prespecified meta-analysis test, DerSimonian-Laird model, prediction interval; amendment A8); results in 3.6, Table 6, Figure 3
14	Reporting bias assessment	2.7 and 3.6 (Egger test where ten or more studies were pooled; not assessed for the smaller pool; stated in Table 6, Table 7 and 4.3)
15	Certainty assessment	2.6 (GRADE per outcome, starting level and named downgrades); Table 7
16	Study selection results and flow	3.1; Figure 1; prisma/prisma-flow.md (all counts machine-derived)
17	Excluded studies with reasons	Table S2 (full-record exclusions with codes and quotes); searches/screen-rule-excluded.tsv, searches/triage-reconciled.tsv and searches/chase-reconciled.tsv in the repository
18	Study characteristics	Tables 2, 3, 4 and S1 (Table 3 lists the randomised and comparative studies; Table S1 lists every included study)
19	Risk of bias in studies	3.7; Table 5 (summary), Table S4 (per study), Table S3 (technical credibility items)
20	Results of syntheses	3.2 to 3.6; Table 6, Table S6 and Figure 3 for the pooled outcomes
21	Reporting biases	3.6 (Egger result) and 4.3 (publication bias not assessable for the accuracy pool; coverage limits)
22	Certainty of evidence	3.7; Table 7
23	Discussion	4.1 to 4.4
24	Registration and protocol	2.1 and Abstract (protocol committed before retrieval; PROSPERO record prepared, submission pending, status to be stated at submission); amendments A1 to A9 in Text S2
25	Support	Declarations (PENDING: author to supply)
26	Competing interests	Declarations (PENDING: author to supply)

#	Item	Where / status
27	Availability of data, code and materials	Declarations; full repository accompanies the manuscript

SWiM (Synthesis Without Meta-analysis) checklist

#	Item	Where / status
1	Grouping studies for synthesis	2.7 (by pillar, then anatomical region; tiers as the evidence axis); protocol section 8
2	Standardised metric and transformation	2.7 (results summarised in their own units without transformation; standardised mean difference used only for the pooled accuracy outcome because measures differ)
3	Synthesis methods	2.7 (counts per category and ranges; no vote counting on direction or significance; random-effects pooling where the prespecified condition held)
4	Criteria for prioritising results	2.7 (order of tier: randomised, comparative, series, technical; independently evaluated AI methods first)
5	Investigation of heterogeneity	2.7 (descriptive, by tier, region, comparator class and text source); 3.2 to 3.6, including the source-stratified credibility and outcome-reporting counts in 3.5 and 3.6; I-squared, prediction intervals, Hartung-Knapp and leave-one-out for the pooled outcomes
6	Certainty of evidence	2.6, 3.7
7	Data presentation	Tables 1 to 7, Figures 2 and 3, Tables S1 to S6
8	Reporting results	3.2 to 3.6 (each synthesis states the number and tier of contributing studies)
9	Limitations of the synthesis	4.3
