# Supplementary material for: Patient-Specific Implant Design: Current Evidence for Artificial Intelligence, Imaging and Computational Planning—A Systematic Review of the Biomedical-Indexed Literature
Source: Bioengineering (Basel). 2026 Oct 1;13(10):1149. doi: 10.3390/bioengineering13101149 (PMC13701698; doi:10.3390/bioengineering13101149)
Supplement: Supplementary file 1 [file bioengineering-13-01149-s001.zip › bioengineering-4586457-supplementary.pdf]

Supplementary materials

Text S1. Extended methods: search, selection, retrieval, charting agreement and corrections

S1.0 Final counts of the flow (all arms, after deduplication)

Stage	Count
Records identified (all sources)	7772
Unique records	5059
Duplicates removed at retrieval	2713
Excluded by rule	2682
To dual AI triage	2377
Excluded by both screeners	1453
Retained from databases	924
Added by citation chasing	55
Reports sought	979
Full reports retrieved	414
Assessed from the abstract	505
No abstract or text, held unresolved	60
Reports assessed	979
Excluded at assessment	273
Unresolved	67
Studies included	639

Records identified by source: Crossref (bounded sweep) 2000; Europe PMC 1687; MEDLINE via PubMed 3207; OpenAlex (3 Sept run) 878. Excluded at assessment by reason: case report 30; duplicate preprint 25; duplicate report 35; no evaluation 17; no method 31; non human 10; not psi 107; not skeletal 3; source type 15.

OpenAlex supplementary arm (amendment A9, run 3 September 2026)

Records retrieved 878; new unique after deduplication against the corpus 412; excluded by rule 116 (i no individualisation term 84; ii no device term 8; iv review or editorial type 24); to dual triage 296; both include 95; excluded by both 153; retained 143; three-class agreement 0.879; kappa on inclusion 0.874.

sol Claude	include	uncertain	exclude
include	95	8	0
uncertain	5	14	0
exclude	4	19	153

Rule-layer audit: 25 rule-excluded records of the main run re-screened, 0 wrongly excluded; one-sided 97.5% upper bound 13.7%, implying at most 353 wrongly excluded among the 2566 main-run exclusions.

The sections that follow describe the main run of 2 September 2026 (before the OpenAlex supplementary arm), whose counts therefore differ from the final flow above.

S1.1 Retrieval by source

Source and strategy	Hits	Retrieved
MEDLINE via PubMed / S1_primary	2457	2456
MEDLINE via PubMed / S2_psi_phrase	751	751
Europe PMC / E1_primary	1686	1687
OpenAlex / O1 (UNAVAILABLE: HTTP 429)	n/a	0
OpenAlex / O2 (UNAVAILABLE: HTTP 429)	n/a	0
OpenAlex / O3 (UNAVAILABLE: HTTP 429)	n/a	0
OpenAlex / O4 (UNAVAILABLE: HTTP 429)	n/a	0
OpenAlex / O5 (UNAVAILABLE: HTTP 429)	n/a	0
OpenAlex / O6 (UNAVAILABLE: HTTP 429)	n/a	0
OpenAlex / O7 (UNAVAILABLE: HTTP 429)	n/a	0
OpenAlex / O8 (UNAVAILABLE: HTTP 429)	n/a	0
OpenAlex / O9 (UNAVAILABLE: HTTP 429)	n/a	0
OpenAlex / O10 (UNAVAILABLE: HTTP 429)	n/a	0
OpenAlex / O11 (UNAVAILABLE: HTTP 429)	n/a	0
OpenAlex / O12 (UNAVAILABLE: HTTP 429)	n/a	0
OpenAlex / O13 (UNAVAILABLE: HTTP 429)	n/a	0
OpenAlex / O14 (UNAVAILABLE: HTTP 429)	n/a	0
Crossref (bounded sweep) / C1	5434738	1000
Crossref (bounded sweep) / C2	3229240	1000

Across all strategies, 6,894 records were retrieved. After deduplication, 4,647 unique records remained and 2,247 duplicates were removed.

S1.2 Rule layer

Rule	Records excluded
i. no individualisation term	1,766
ii. no device term	174
iii. non human signal	40
iv. review or editorial type	386
v. case report type	200

The rule layer excluded 2,566 records, set aside 386 reviews for citation chasing, and passed 2,081 records to dual AI triage.

In the seeded audit, 25 rule-excluded records were re-screened and 0 false excludes were found. The one-sided 97.5% Clopper-Pearson upper bound for the false-exclusion proportion was 13.7%. Applied to the 2,566 rule-excluded records, this corresponds to an upper bound of 353 wrongly excluded records.

S1.3 Dual AI triage

Database-search records

sol Claude Opus	Include	Uncertain	Exclude
Include	516	33	7
Uncertain	44	43	15
Exclude	27	96	1,300

Measure	Value

Measure	Value
Records triaged	2,081
Raw agreement, three classes	0.893
Cohen's kappa, three classes	0.781
Raw agreement, include versus not include	0.947
Cohen's kappa, include versus not include	0.866
Both include	516
Both exclude	1,300
Retained	781

Citation-chase increment

sol Claude Opus	Include	Uncertain	Exclude
Include	24	0	3
Uncertain	1	0	1
Exclude	18	8	89

Measure	Value
Records triaged	144
Raw agreement, three classes	0.785
Cohen's kappa, three classes	0.496
Raw agreement, include versus not include	0.847
Cohen's kappa, include versus not include	0.592
Both include	24
Both exclude	89
Retained	55

S1.4 Citation chasing

Measure	Count
Reviews chased	32
References seen	836
New candidates after deduplication and date restriction	728
Candidates passed to dual AI triage	144
Candidates retained	55

Rule-layer exclusion of candidates	Count
i. no individualisation term	234
ii. no device term	57
iii. non human signal	2
iv. review or editorial type	39
v. case report type	14
pre 2015	238

Reference lists were obtained through NCBI links, which index references only for PubMed Central deposits. Citation chasing was therefore partial.

S1.5 Full-record assessment inputs

Full-record assessment was conducted for 979 records.

Assessment input	Records
PubMed Central full text	322
Europe PMC full text	14
Unpaywall full text	78
Abstract only (no full text, abstract present)	505
Title or metadata only (no abstract, no full text; held unresolved)	60
of which: retrieval returned a landing page rather than the article (the other 3 of the 14 audited landing pages were texts retrieved only for the artificial intelligence extraction)	11

Abstract-only records are listed below.

ID	Title
pmid:42674940	Accuracy of β-tricalcium phosphate gap patient-specific implants in chin osteotomies: a p…
pmid:42608239	PEEK patient-specific implants for orbital reconstruction: accuracy and clinical outcomes.
pmid:42570496	Patient-Specific Sensitivity of Osteotomy Interface Modelling in Mandibular Reconstructio…
pmid:42551059	A novel 3D-printed implant system for mandibular reconstruction (IPS® MotionPlate) - a fi…
pmid:42448534	Three-dimensional virtual planning and custom polyetheretherketone reconstruction for sph…
pmid:42418929	Virtual surgical planning in frontofacial advancement using a customized RED II distracto…
pmid:42413458	Accuracy of maxillary positioning using patient-specific implants in a surgery-first appr…
pmid:42407172	A methodology for constructing and assessing a combined experimental and computational va…
pmid:42320193	Dual-CNN-based AI framework for patient-specific design of short femoral stems.
pmid:42308584	A TPMS-integrated paediatric proximal femoral osteotomy implant demonstrates structural f…
pmid:42302371	Finite element analysis of polycarbonate-urethane coatings for talus implant applications.
pmid:42290023	A Novel Alloplastic Patient-Specific Temporalis Tendon Enthesis for Mandibular Resection…
pmid:42233609	Mechanobiological modeling of bone healing in mandibular reconstruction: a comparative st…
pmid:42210774	Numerical Inverse Design of Patient-Specific Dental Implants: Accelerating FEA-Based Opti…
pmid:42183622	Assessment of Accuracy and Orbital Volume Using Patient-Specific Titanium Implant Versus…
pmid:42169235	Surface roughness optimisation in FFF tibial insert patterns for silicone mould.
pmid:42160799	Why detailed modeling matters in the FE-based pre-clinical evaluation of temporomandibula…
pmid:42090197	Design and Manufacturing Process of 3D-Printed Metamaterial Calcaneal Plates via Scalar F…
pmid:42020225	Osseointegration dynamics of mandibular patient-specific implants: A mechanoregulation-ba…
pmid:41861487	Conceptual design of growth-adaptive calcium phosphate cement implants for large paediatr…
pmid:41840235	Reverse-engineered stock plates as a “makeshift” patient-specific alternative: expanding…
pmid:41784751	Reconstruction of normal morphology for simulated femoral condyle lesions based on shape…
pmid:41759452	A patient-specific PEEK fixation system for simultaneous maxillary advancement and midfac…
pmid:41554658	Early-stage stability and degradation of patient-specific biodegradable 3D-printed implan…
pmid:41553224	Patient-Specific Lattice Cage Design for Cervical Spinal Fusion.
pmid:41544967	Virtual surgical planning and 3D custom-made implants in the management of orbital floor…
pmid:41524259	Patient-specific Subperiosteal Implant for Dental Rehabilitation of Severe Atrophic Maxil…
pmid:41506104	Orthognathic surgical accuracy and cost-efficiency of patient-specific implants using int…
pmid:41506030	3D tibial HU reconstruction from biplanar X-rays utilizing a hybrid PCA-CNN framework.

ID	Title
pmid:41499545	Correction of Mandibular Asymmetry With Bilateral Asymmetric Custom-Made 3D Implant.
pmid:41418444	Benchmarking PA12 and PA12CF35 for selective laser sintering of patient-specific implants…
pmid:41397797	[Early follow-up study on three-dimensional-printed customized porous acetabular componen…
pmid:41384766	Bioresorbable-bioactive auxetic “personalised” phalanx with a CT-guided AI-driven model t…
pmid:41344382	Orbital Floor Reconstruction With Patient-Specific Implants: Comparable Outcomes Between…
pmid:41336765	A Novel Approach for Shape Segmentation of Vertebrae: Decomposition into Anatomical Regio…
pmid:41285248	Biomechanical evaluation of fixation systems in different segmental Le Fort I osteotomies…
pmid:41274196	Biomechanical analysis of pedicle screw density and rod contouring in adolescent idiopath…
pmid:41248445	Virtual Surgical Planning in Pediatric Craniomaxillofacial Surgery: A 12-Year Institution…
pmid:41114876	Local mechanical stresses can explain the mechanoregulation of bone healing after mandibu…
pmid:41094110	Use of machine learning predictive models in sagittal alignment planning in adolescent id…
pmid:41090924	Customized Titanium Implants for Cranial Fibrous Dysplasia: A Personalized Surgical Appro…
pmid:40930860	Biomechanical impact of sagittal curvature radius in glenoid fossa on TMJ prosthesis perf…
pmid:40876815	Development of porous customized Ti-6Al-4V cranial implant manufactured by laser beam pow…
pmid:40845376	Virtual Surgical Planning for Facial Masculinization Surgery.
pmid:40779986	Two-piece versus single-piece patient-specific titanium subperiosteal implants in atrophi…
pmid:40753708	Prototype design of porous Ti6Al4V intraosseous implant for use in mandibular reconstruct…
pmid:40726307	Invited review article: Patient-specific 3D-Printed metal plates for osteotomy and fractu…
pmid:40694406	A Biomimetic Titanium Scaffold With and Without Magnesium Filled for Adjustable Patient-S…
pmid:40681392	Impact-resistance of bio-inspired functionalized polyether-ether-ketone implant for crani…
pmid:40614522	Influence of pre-bending on primary fixation stability in one-segmental mandibular recons…
pmid:40578009	A finite element biomechanical investigation of lumbar spine segments through novel inter…
pmid:40512299	Strategies for the Patient-Specific Implant Angle of Bone Scaffolds Using Optimization.
pmid:40503891	Development of Machine Learning Models to Predict Tumor Endoprosthesis Survival.
pmid:40494102	Advanced topology optimisation for porous hip Implants: Bridging in silico models and in…
pmid:40483854	SCAI-Net: An AI-driven framework for optimized, fast, and resource-efficient skull implan…
pmid:40483235	Automatic MRI segmentation of masticatory muscles using deep learning enables large-scale…
pmid:40480278	Patient-Specific Titanium Implants Reduce Hardware-Related Complications Compared to Stoc…
pmid:40399182	Long-term clinical results of additively manufactured subperiosteal implants for the trea…
pmid:40288205	Towards the development of reliable finite element models of Ti6Al4V trabecular structure…
pmid:40232250	Clinical outcomes of 3-dimensional printed custom porous polyethylene orbital implant for…
pmid:40209337	The utility of additively manufactured β-Ti latticed hip implants in reducing femoral str…
pmid:40199670	A semi-automated design workflow for patient-specific implants in orthognathic surgery us…
pmid:40183614	Computer-assisted planning and modeling for the surgical treatment of patients with fibro…
pmid:40180533	Blinded prediction of custom-made pelvic implant failure using patient-specific finite el…
pmid:40121172	Custom design of a temporomandibular joint prosthesis: A kinematic approach evaluated by…
pmid:40107082	Optimization of primary screw stability in Trabecular bone using neural network-based mod…
pmid:40047935	Utilizing 3d-printed patient-specific porous titanium cones in complex primary and revisi…

ID	Title
pmid:40040189	Automatic Cranial Defect Reconstruction with Self-Supervised Deep Deformable Masked Autoe…
pmid:40024182	Biomechanical assessment in virtual surgical planning for mandibular reconstruction.
pmid:40009971	Automatic skull reconstruction by deep learnable symmetry enforcement.
pmid:39992375	Oversized Total Talar Prosthesis Enhances the Ankle Stability After Total Talus Replaceme…
pmid:39947960	Finite element analysis of biomechanical effects of oversized total talar prosthesis and…
pmid:39933970	Single-jaw patient-specific implants in bimaxillary orthognathic surgery: Randomized cros…
pmid:39921749	Three dimensionalprinted titanium block to reconstruct severe acetabular bone defects in…
pmid:39892636	A statistical shape and density model can accurately predict bone morphology and regional…
pmid:39875225	Jawline improvement using patient-specific angle implants with virtual planning in orthog…
pmid:39757350	Finite Element Model of Patient-Specific Flanged Acetabular Components Highlights Biomech…
pmid:39700854	3d-printed sacral reconstruction prosthesis from multiscale topology optimization: A comp…
pmid:39603155	Towards improved functionality of mandibular reconstruction plates enabled by additively…
pmid:39602978	Design of patient-specific mandibular reconstruction plates and a hybrid scaffold.
pmid:39553819	Management of Atrophic Edentulous Mandible Fractures Utilizing Virtual Surgical Planning…
pmid:39489075	Enhancing biomechanical outcomes in proximal femoral osteotomy through optimised blade pl…
pmid:39266434	PSI: Planner-specific, physician-specific, or patient-specific implant for orbital recons…
pmid:39265478	Improving deep learning-based automatic cranial defect reconstruction by heavy data augme…
pmid:39246118	Inverse design of skull osteoinductive implants with multi-level pore structures through…
pmid:39244030	Feasibility of augmented reality combine patient-specific implants (PSI) applied to navig…
pmid:39186906	Anatomically and mechanically conforming patient-specific spinal fusion cages designed by…
pmid:39182846	Long-term enophthalmos after complex orbital bone loss successfully treated with patient-…
pmid:39160013	From imaging to personalized 3D printed molds in cranioplasty.
pmid:39090062	[The reconstruction techniques and mid-term clinical outcomes of hip revision for acetabu…
pmid:39084954	Design and optimization of a novel patient-specific subperiosteal implant additively manu…
pmid:39008077	Comparison of the use of biocompatible materials and titanium in the treatment of midshaf…
pmid:39004739	Automated design of bone-preserving, insertable, and shape-matching patient-specific acet…
pmid:38996963	Endoscopic Endonasal Occipitocervical Fixation with a Customized Three-Dimensional Printe…
pmid:38992884	Bone remodeling after revision total hip arthroplasty for large acetabular defects.
pmid:38983674	Biomechanical study of using patient-specific diaphyseal femoral cone in revision total k…
pmid:38981580	Patient-specific Implants Improve Volumetric Surgical Accuracy Compared to Stock Reconstr…
pmid:38906577	Cylindrical depth image based customized helical bone plate design.
pmid:38896405	Virtual reconstruction of orbital defects using Gaussian process morphable models.
pmid:38776646	Biomechanical investigation of a customized interspinous spacer system in the treatment o…
pmid:38762148	A scapular statistical shape model can reliably predict premorbid glenoid morphology in c…
pmid:38669091	Hip joint center lateralization minimally affects the biomechanics of patient-specific fl…
pmid:38492502	Dynamic topology optimization of 3D-Printed transtibial orthopedic implant using tunable…
pmid:38433178	Influence of build orientation and support structure on additive manufacturing of human k…
pmid:38430381	Thickness and design features of clinical cranial implants-what should automated methods…
pmid:38394650	The potential of proximal junctional kyphosis prevention using a novel tether pedicle scr…
pmid:38388230	Comparison of patient specific implant reconstruction vs conventional titanium mesh recon…
pmid:38368208	Customized orbital implant versus 3D preformed titanium mesh for orbital fracture repair:…
pmid:38368205	Clinical and radiographic assessment of patient-specific transantral reconstruction of or…
pmid:38361509	Artificial intelligence-based orthopaedic perpetual design.

ID	Title
pmid:38340477	Personalized lattice-structured prosthesis as a graftless solution for mandible reconstru…
pmid:38274145	Total Talar Replacement: Surgical Technique.
pmid:38246644	Method of computational design for additive manufacturing of hip endoprosthesis based on…
pmid:38237204	Performance of personalised prosthesis under static pressure: Numerical analysis and expe…
pmid:38182026	Finite element analysis of a customized coronoid prosthesis for traumatic coronoid defici…
pmid:38151940	[Three-dimensional reconstruction of femur based on Laplace operator and statistical shap…
pmid:38143713	The Application of a Multi-Material Flexible Chain Mail for the Design of an Artificial S…
pmid:38129183	Skeletal stability after mandible bilateral sagittal split osteotomy - comparison of pati…
pmid:38064852	Biomechanical evaluation of CAD/CAM magnesium miniplates as a fixation strategy for the t…
pmid:37985018	Stress-shielding resistant design of custom pelvic prostheses using lattice-based topolog…
pmid:37886407	A Novel Approach of Customized Pelvic Implant Design Based on Symmetrical Analysis and 3D…
pmid:37875041	Biomechanical feasibility of non-locking system in patient-specific mandibular reconstruc…
pmid:37797593	Design and analysis of multi-material structures of 3D printed implants of mandible.
pmid:37757617	Coupling biomechanical models of implants with biodegradation models: A case study for bi…
pmid:37713150	A Comparative Study of Titanium Cranioplasty for Extensive Calvarial Bone Defects: Three-…
pmid:37678107	Biomechanical evaluation of additively manufactured patient-specific mandibular cage impl…
pmid:37658855	Precontoured dynamic compression plate using patient-specific 3D-printed models in minima…
pmid:37643059	Combining Patient-specific Implant With Malar Reduction to Repair Mid-facial Asymmetry Ca…
pmid:37624362	Design and development of a patient-specific temporomandibular joint implant: Probing the…
pmid:37562162	Finite element analysis of a customized implant in PMMA coupled with the cranial bone.
pmid:37556925	Mechanical behaviour of reconstructed defected skull with custom PEEK implant and Titaniu…
pmid:37544200	Design of customized implants and 3D printing of symmetric and asymmetric cranial cavitie…
pmid:37534346	Comparison of Two Types of Patient Specific Implants (PSI) and Quad Zygoma Implant (QZI)…
pmid:37517940	Maxillomandibular advancement is a safe procedure in patients with obstructive sleep apno…
pmid:37460178	[Three-dimensional finite element model construction and biomechanical analysis of custom…
pmid:37351673	Toward global availability of low-cost, patient-specific cranial implants: creation and v…
pmid:37302708	Late Frontal Bone Reconstruction Using Three-Dimensional Printed Models for Titanium Mesh…
pmid:37244075	Design and manufacturing of patient-specific Ti6Al4V implants with inhomogeneous porosity.
pmid:37216926	Biomechanical analysis of a single-level customized cage screw fixation for anterior cerv…
pmid:37185164	Parameter-based patient-specific restoration of physiological knee morphology for optimiz…
pmid:37145143	Beyond type III Paprosky acetabular defects: are partial pelvic replacements with iliosac…
pmid:37041951	Scope of PSI in Maxillofacial Region: Our Experience.
pmid:36965999	Patient-specific femoral implant design using metamaterials for improving load transfer a…
pmid:36965217	Development of biodegradable customized tibial scaffold with advanced architected materia…
pmid:36920278	Finite element analysis of biplanar customized reconstruction plates for lateral and cent…
pmid:36898626	Interbody Fusion Cage Design Driven by Topology Optimization.
pmid:36825890	The optimal strategy for 3D-printed uncemented endoprosthesis for the bone defect reconst…
pmid:36801786	On the need of a scale-dependent material characterization to describe the mechanical beh…

ID	Title
pmid:36792242	Comparative evaluation of a patient-specific customised plate designs and screws for part…
pmid:36792235	Finite element analysis of screw fixation durability under multiple boundary and loading…
pmid:36639878	Patient-Specific Orbital Implants Vs. Pre-Formed Implants for Internal Orbital Reconstruc…
pmid:36563638	Early experience of wafer-free Le Fort I osteotomy with patient-specific implants in clef…
pmid:36541118	Submodelling approach to screw-to-bone interaction in additively manufactured subperioste…
pmid:36442275	Novel and accurate 3D-Printed surgical guide for mandibular reconstruction with integrate…
pmid:36401069	Accuracy and safety of in-house surgeon-designed three-dimensional-printed patient-specif…
pmid:36385681	Filling the gap: a series of 3D-printed titanium truss cages for the management of large,…
pmid:36384079	Accuracy and outcomes of virtual surgical planning and 3D-printed guides for osseous free…
pmid:36310474	[Rapid femur modeling method based on statistical shape model].
pmid:36302272	On interaction between fatigue of reconstruction plate and time-dependent bone remodeling.
pmid:36257198	Deep learning-based framework for automatic cranial defect reconstruction and implant mod…
pmid:36241591	Reconstruction of orbital wall fractures with superior orbital fissure syndrome using ind…
pmid:36202213	What is the Accuracy of PEEK Implants for Cranioplasty in Comparison to Their Patient Spe…
pmid:36184071	Accuracy of mandibular anterior subapical osteotomy by virtual planning in orthognathic s…
pmid:36152972	Finite element analysis of the biomechanical effects of titanium and Cfr-peek additively…
pmid:36085744	CranGAN: Adversarial Point Cloud Reconstruction for patient-specific Cranial Implant Desi…
pmid:36068031	Biomechanical analysis of a customized lumbar interspinous spacer based on transfacetoped…
pmid:36041093	Accuracy of Intentional Change of Frontal Ramal Inclination From Virtual to Actual Orthog…
pmid:35963834	Clinical application of automated virtual orbital reconstruction for orbital fracture man…
pmid:35950785	Biomechanical evaluation of customized root implants in alveolar bone: A comparative stud…
pmid:35880901	Design of patient specific basal dental implant using Finite Element method and Artificia…
pmid:35872386	Biomechanical validation of structural optimized patient-specific mandibular reconstructi…
pmid:35872223	Deep convolutional neural network-based automated segmentation of the maxillofacial compl…
pmid:35835610	Comprehensive virtual orthognathic planning concept in surgery-first patients.
pmid:35727116	Designing and in vitro testing of a novel patient-specific total knee prosthesis using th…
pmid:35660552	Semi-automated digital workflow to design and evaluate patient-specific mandibular recons…
pmid:35443132	Assessment of Customized Alveolar Bone Augmentation Using Titanium Scaffolds vs Polyether…
pmid:35297154	Custom design and biomechanical clinical trials of 3D-printed polyether ether ketone femo…
pmid:35275582	3D-printed porous condylar prosthesis for temporomandibular joint replacement: Design and…
pmid:35255279	Analysis of the effects of mandibular reconstruction based on microvascular free flaps af…
pmid:35245699	In silico evaluation of lattice designs for additively manufactured total hip implants.
pmid:35237925	The evaluation of artificial talus implant on ankle joint contact characteristics: a fini…
pmid:35183467	Accurate reconstruction of bone defects in orbital-maxillary-zygomatic (OMZ) complex with…
pmid:35139481	Marker-free surgical navigation of rod bending using a stereo neural network and augmente…
pmid:35119415	Reconstruction of Cranial Bone Defects Using Polyamide 12 Patient-Specific Implant: Long…
pmid:35061064	Augmented reality and three-dimensional plate library-assisted posterior minimally invasi…
pmid:35014088	Influence of different diameter reductions in the labial neck region on the stress distri…
pmid:35001211	The comparison of stress and strain between custom-designed bone plates (CDBP) and

ID	Title
	lockin…
pmid:34984904	Additively Manufactured Lattice-like Subperiosteal Implants for Rehabilitation of the Sev…
pmid:34741907	Preclinical study of additive manufactured plates with shortened lengths for complete man…
pmid:34715640	Development and validation of a parametric human mandible model to determine internal str…
pmid:34657530	Mandibular reconstruction system reliability analysis using probabilistic finite element…
pmid:34563812	Biomechanical comparison of locking and non-locking patient-specific mandibular reconstru…
pmid:34450324	Additive Manufacturing for Personalized Skull Base Reconstruction in Endoscopic Transcliv…
pmid:34423622	[3D printed personalized lumbar spinal fusion device design and biomechanical performance…
pmid:34340106	Automatic skull defect restoration and cranial implant generation for cranioplasty.
pmid:34298147	One size may not fit all: patient-specific computational optimization of locking plates f…
pmid:34275217	[Reconstruction of zygomatico-orbtial and maxillary bone defects using three-dimensional…
pmid:34260465	Virtual Surgical Planning Combined With Intraoperative Navigation in Mandibular Bilateral…
pmid:34227419	Design of patient specific bone stiffness mimicking scaffold.
pmid:34225889	Topological design and biomechanical evaluation for 3D printed multi-segment artificial v…
pmid:34224462	Virtual Surgical Planning for Intracranial Intraosseous Meningioma Reconstruction.
pmid:34102730	[Early clinical efficacy analysis of personalized three-dimensional printing talus prosth…
pmid:33864971	A personalized 3D-printed plate for tibiotalocalcaneal arthrodesis: Design, fabrication,…
pmid:33784838	The three-dimensional shape symmetry of the lunate and its implications.
pmid:33707038	Intraoperative real-time navigation and intraoperative three-dimensional imaging for pati…
pmid:33683380	Virtual planning on contralateral hemipelvis for posteriorly fixed acetabular fractures.
pmid:33652236	Design Optimization and Manufacturing of Bio-fixed tibial implants using 3D printing tech…
pmid:33647729	On the limits of finite element models created from (micro)CT datasets and used in studie…
pmid:33529765	Evaluation of Patient-Specific Cranial Implant Design Using Finite Element Analysis.
pmid:33524959	3D printing-assisted fabrication of a patient-specific antibacterial radial head prosthes…
pmid:33494021	3D-printed PEEK implant for mandibular defects repair - a new method.
pmid:33452834	Accuracy of Fibula Reconstruction Using Patient-Specific Cad/Cam Plates: A Multicenter St…
pmid:33452611	Quantitative analysis of regional specific pelvic symmetry.
pmid:33449664	Effect of Porous Microstructures on the Biomechanical Characteristics of a Root Analogue…
pmid:33370061	Low-Cost, Three-Dimensionally-Printed, Anatomical Models for Optimization of Orbital Wall…
pmid:33359737	Patient-Specific Three-Dimensional Printing Guide for Single-Stage Skull Bone Tumor Surge…
pmid:33349103	Biomechanical effects of reconstruction of the posterior structures after laminectomy wit…
pmid:33317937	In silico patient-specific optimization of correction strategies for thoracic adolescent…
pmid:33310682	Porous structure design and mechanical behavior analysis based on TPMS for customized roo…
pmid:33237435	Accuracy of custom temporomandibular joint replacement surgery using a virtual surgical p…
pmid:33187871	Topology optimization of a mandibular reconstruction plate and biomechanical validation.
pmid:33170352	Use of porous custom-made cones for meta-diaphyseal bone defects reconstruction in knee r…
pmid:33080431	Implicit and explicit finite element models predict the mechanical response of calcium pho…
pmid:33010215	Customized Titanium Reconstruction of Orbital Fractures Using a Mirroring Technique for V…
pmid:32840135	Influence of tibial bearing curvatures of a customised total knee implant on squatting mo…
pmid:32660275	Biomechanical performance design of joint prosthesis for medical rehabilitation via gener…
pmid:32590512	The Latest Evolution in Virtual Surgical Planning: Customized Reconstruction Plates in Fr…

ID	Title
pmid:32518971	Computer-assisted surgery for replacement of the temporomandibular joint with customized…
pmid:32506737	Computer-aided porous implant design for cranio-maxillofacial defect restoration.
pmid:32505528	Accuracy of laser-melted patient-specific implants in genioplasty - A three-dimensional r…
pmid:32427351	Can custom 3D printed implants successfully reconstruct massive acetabular defects? A 3D-…
pmid:32410539	Design of Patient Specific Spinal Implant (Pedicle Screw Fixation) using FE Analysis and…
pmid:32320044	Biomechanical Analysis to Probe Role of Bone Condition and Subject Weight in Stiffness Cu…
pmid:32279847	Design of a patient-specific mandible reconstruction implant with dental prosthesis for m…
pmid:32279058	Topological optimization of 3D printed bone analog with PEKK for surgical mandibular reco…
pmid:32242531	Additive manufacturing of reconstruction devices for maxillofacial surgery: design and ac…
pmid:32103625	Assessment of 3-dimensional bone augmentation of severely atrophied maxillary alveolar ri…
pmid:31870519	Virtual planning, simultaneous dental implantation and CAD/CAM plate fixation: a paradigm…
pmid:31789791	Precision in Oculofacial Surgery: Made-To-Specification Cast-Molded Implants in Orbital R…
pmid:31783282	Biomechanics of alloplastic mandible reconstruction using biomaterials: The effect of imp…
pmid:31741290	Subject-specific finite element analysis of a lumbar cage produced by electron beam melti…
pmid:31675463	Development of customized implant and customized surgical osteotomy guide in ablative tum…
pmid:31655451	Biomechanical comparison of a 3D-printed sacrum prosthesis versus rod-screw systems for r…
pmid:31482715	Automated CT bone segmentation using statistical shape modelling and local template match…
pmid:31367783	Custom made onlay implants in peek in maxillofacial surgery: a volumetric study.
pmid:31353173	The use of patient-specific implants in genioplasty and its clinical accuracy: a prelimin…
pmid:31319772	Preliminary study of a customised total knee implant with musculoskeletal and dynamic squ…
pmid:31304779	Structure and properties of a personalized bio-fixed implant prepared with selective lase…
pmid:31277543	[Design and finite element analysis of customized pelvic prosthesis].
pmid:31261343	Patient-Specific Reconstruction Utilizing Computer Assisted Three-Dimensional Modelling f…
pmid:31229384	A general multi-objective topology optimization methodology developed for customized desi…
pmid:31122108	Modeling three-dimensional-printed trabecular metal structures with a homogenization appr…
pmid:31082835	Clinical Application of a Specific Simulation Software for 3-Dimensional Orbital Volume M…
pmid:30928205	Development of a statistical shape-function model of the implanted knee for real-time pre…
pmid:30894302	Accuracy of patient-specific implants and additive-manufactured surgical splints in ortho…
pmid:30801978	Computational intelligence based design of implant for varying bone conditions.
pmid:30777714	Accuracy of custom-fitted temporomandibular joint alloplastic reconstruction and virtual…
pmid:30629776	Comparison of biomechanical characteristics in treatment of distal humerus fracture using…
pmid:30562250	Symmetry Matching of the Medial Acetabular Surface-A Quantitative Analysis in View of Pat…
pmid:30537876	Patient-specific design process and evaluation of a hip prosthesis femoral stem.
pmid:30497901	Combined Application of Modified Three-Dimensional Printed Anatomic Templates and Customi…
pmid:30375662	Computational analysis of customized cruciate retaining total knee arthroplasty restorati…
pmid:30366309	CT image segmentation of bone for medical additive manufacturing using a convolutional ne…

ID	Title
pmid:30337077	Surgery of complex craniofacial defects: A single-step AM-based methodology.
pmid:30306217	Customized implants for acetabular Paprosky III defects may be positioned with high accur…
pmid:30286374	Nondestructive characterization of bone tissue scaffolds for clinical scenarios.
pmid:30220611	Primary reconstruction of orbital fractures using patient-specific titanium milled implan…
pmid:30131272	Evolutionary steps in the design and biofunctionalization of the additively manufactured…
pmid:30025603	Biomechanical evaluation of Chinese customized three-dimensionally printed total temporom…
pmid:29903488	Stand-alone lumbar cage subsidence: A biomechanical sensitivity study of cage design and…
pmid:29859818	A new three-dimensional, print-on-demand temporomandibular prosthetic total joint replace…
pmid:29807784	3D printing utility for surgical treatment of acetabular fractures.
pmid:29408349	Vertebral body replacement using patient-specific three-dimensional-printed polymer impla…
pmid:29408000	Design of complex bone internal structure using topology optimization with perimeter cont…
pmid:29368178	Evaluation of accuracy of virtual surgical planning for patient-specific pre-contoured pl…
pmid:29260271	Reconstruction of Mandibular Contour Using Individualized High-Density Porous Polyethylen…
pmid:29233510	Patient-specific puzzle implant preformed with 3D-printed rapid prototype model for combi…
pmid:29216793	Parametric study of patient-specific femoral locking plates based on a combined musculosk…
pmid:29191076	Customized surface-guided knee implant: Contact analysis and experimental test.
pmid:29088629	Overcorrected glenoid implants to prevent recurrent glenohumeral subluxation after total…
pmid:28888931	Biomechanical testing of zirconium dioxide osteosynthesis system for Le Fort I advancemen…
pmid:28868631	Finite element analysis of customized implant in mandibular reconstruction after tumor re…
pmid:28828975	Customized Titanium Mesh Based on the 3D Printed Model vs. Manual Intraoperative Bending…
pmid:28793965	Clinical accuracy of waferless maxillary positioning using customized surgical guides and…
pmid:28786025	Clinical effects of 3-D printing-assisted personalized reconstructive surgery for blowout…
pmid:28747263	The validity and reliability of computed tomography orbital volume measurements.
pmid:28709790	Corrective Osteotomy for Malunited Diaphyseal Forearm Fractures Using Preoperative 3-Dime…
pmid:28639517	Development of finite element model for customized prostheses design for patient with pel…
pmid:28629601	A custom-made temporomandibular joint prosthesis for fabrication by selective laser melti…
pmid:28585457	Effect of geometric parameters in the design of customized hip implants.
pmid:28552926	Modelling and structural analysis of skull/cranial implant: beyond mid-line deformities.
pmid:28552201	Increasing the accuracy of mandibular reconstruction with free fibula flaps using functio…
pmid:28474182	Homogenous scaffold-based cranial/skull implant modelling and structural analysis-unit ce…
pmid:28463685	Reconstruction of Maxillary and Orbital Floor Defect With Free Fibula Flap and Whole Indi…
pmid:28434826	The accuracy of patient specific implant prebented with 3D-printed rapid prototype model…
pmid:28427318	Load application for the contact mechanics analysis and wear prediction of total knee rep…
pmid:28199931	A personalized 3D-printed prosthetic joint replacement for the human temporomandibular jo…
pmid:28106226	Applied Taguchi method for fatigue testing of customized hip implant.
pmid:28056711	Optimization of custom cementless stem using finite element analysis and elastic modulus…
pmid:28005746	Customized Orbital Wall Reconstruction Using Three-Dimensionally Printed Rapid Prototype…
pmid:27981068	Structure-enhanced local phase filtering using L0 gradient minimization for efficient sem…
pmid:29786331	[APPLICATION OF THREE-DIMENSIONAL PRINTING CUSTOMIZED BONE CEMENT MODELS ON REPAIR OF BON…
pmid:27627721	Can an Endplate-conformed Cervical Cage Provide a Better Biomechanical Environment than a…

ID	Title
pmid:27501991	Computer-assisted versus traditional freehand technique in fibular free flap mandibular r…
pmid:27431399	Design and fabrication of 3D-printed anatomically shaped lumbar cage for intervertebral d…
pmid:27167515	Skull reconstruction after resection of bone tumors in a single surgical time by the asso…
pmid:26835729	A comparison study on the design of mirror and anatomy reconstruction technique in maxill…
pmid:26803563	An automatic segmentation system of acetabulum in sequential CT images for the personaliz…
pmid:26660897	Designing patient-specific 3D printed craniofacial implants using a novel topology optimi…
pmid:26608137	Late Reconstruction of the Orbit With Patient-Specific Implants Using Computer-Aided Plan…
pmid:26598243	Computer-aided design and computer-aided modeling (CAD/CAM) generated surgical splints, c…
pmid:26596361	The feasibility of a custom-made endoprosthesis in mandibular reconstruction: Implant des…
pmid:26566203	The feasibility of producing patient-specific acrylic cranioplasty implants with a low-co…
pmid:26405891	Automatic extraction of 3D anatomical feature curves of hip bone models reconstructed fro…
pmid:26363532	Exploring inter-subject anatomic variability using a population of patient-specific femur…
pmid:26281256	[Reconstruction of the orbital fracture with enophthalmos using customized titanium mesh…
pmid:26174791	Design and optimization of the fixing plate for customized mandible implants.
pmid:26000081	Finite element analysis of patient-specific condyle fracture plates: a preliminary study.
pmid:25974770	Craniofacial reconstruction using patient-specific implants polyether ether ketone with c…
pmid:25911122	Accuracy of fibula reconstruction using patient-specific CAD/CAM reconstruction plates an…
pmid:25703687	Finite element analysis of a condylar support prosthesis to replace the temporomandibular…
pmid:25588747	Spontaneous fractures in custom-made porous hydroxyapatite cranioplasty implants: is frag…
pmid:25556524	Computer simulation and image guidance for individualised dynamic spinal stabilization.
pmid:25465486	Patient specific implants (PSI) in reconstruction of orbital floor and wall fractures.
pmid:41344151	Automated high-fidelity 3D reconstruction of middle-ear ossicles from low-resolution clin…
pmid:41266230	Comparing the importance of implant customization vs. usage of a fibular graft using a no…
pmid:41218231	Biomechanical impact of tooth root morphology to inform dental implant design.
pmid:40326366	On design, fabrication, and pre-clinical validation of customized 3D-printed dental impla…
pmid:39731307	Long-Term Complications in Patient-Specific Plates for Maxillary Reconstruction.
pmid:38409347	Endoscopic-Assisted Forehead Augmentation with Polyetheretherketone (PEEK) Patient-Specif…
pmid:38408764	The role of bone remodeling in measuring migration of custom implants for large acetabula…
pmid:37536840	A biomechanical study of osseointegrated patient-matched additively manufactured implant…
pmid:37023601	HyM3D: A hybrid method for the automatic 3D reconstruction of a defective cranial vault.
pmid:33914952	Bilateral asymmetry of bone density adjacent to pelvic sarcomas: A retrospective study us…
pmid:27957883	Virtual anatomical reconstruction of large acetabular bone defects using a statistical sh…
doi:10.20944/preprints202507.1208.v1	3D Printing and Virtual Surgical Planning in Craniofacial and Thoracic Surgery: Success C…
doi:10.20944/preprints202501.2283.v1	Simulation of a Custom-Made Temporomandibular Joint –an Academic View on an Industrial Wo…
doi:10.20944/preprints202307.1391.v1	Clinical Application of a Patient-Specific 3D Printed Medical Device: Surgical Planning a…
doi:10.20944/preprints202307.1681.v1	Delayed Orbital Floor Reconstruction Using a Mirroring Technique and patient-specific imp…
doi:10.20944/preprints202407.0500.v1	Computer Simulation-Based Multi-objective Optimisation of Additively Manufactured Cranial…
doi:10.20944/preprints202107.0110.v1	A Multi-Criteria Assessment Strategy for 3d Printed Porous Polyetheretherketone (Peek) Pa…
doi:10.21203/rs.3.pex-1384/v1	A Comprehensive 3D-Molded Bone Flap Protocol for Patient-Specific Cranioplasty
title:1ae5734e965933fa	Surgical planning and finite element analysis for the neurocraneal protection in craniopl…
doi:10.20944/preprints202307.0273.v1	Pre-planning of Individualized Talus Implants Based on Computed Tomography-Automated Segm…

ID	Title
doi:10.20944/preprints202207.0017.v1	Evaluation of Intraorbital Soft Tissue Modifications after Traumatic Injuries Management
title:d5a82fbc298ba79c	Novel bone fixation implants minimising stress shielding
doi:10.1016/j.asoc.2018.01.025	Design of patient specific dental implant using FE analysis and computational intelligenc…
doi:10.1016/j.ijom.2023.10.293	ORBITAL PATIENT-SPECIFIC IMPLANT: PATIENT-SPECIFIC, PHYSICIAN-SPECIFIC OR PLANNER-SPECIFI…
doi:10.18502/keg.v2i2.602	Customised design of a patient specific 3D printed whole mandible implant
doi:10.1016/j.jmbbm.2020.103700	Design of a patient-specific mandible reconstruction implant with dental prosthesis for m…
doi:10.37896/jxu15.4/045	Finite Element Analysis of Patient Specific Maxillofacial Implant
doi:10.3390/app15073522	Development of Surrogate Model for Patient-Specific Lattice-Structured Hip Implant Design…
doi:10.1016/j.wneu.2021.01.102	Evaluation of Patient-Specific Cranial Implant Design Using Finite Element Analysis
doi:10.2139/ssrn.4968323	Biomechanical Evaluation of Auxein's Patient Specific Implant for Temporomandibular Joint…
doi:10.3390/technologies11020058	Computational Investigation of a Tibial Implant Using Topology Optimization and Finite El…
doi:10.1016/j.bprint.2025.e00412	Topology optimization and manufacturing of maxillofacial patient specific implant using F…
doi:10.1016/j.finel.2017.07.010	Direct medical image-based Finite Element modelling for patient-specific simulation of fu…
doi:10.14445/22315381/ijett-v70i9p235	Finite Element Analysis of a Patient Specific Maxillofacial Implant
doi:10.3390/app10144738	Design of a Metal 3D Printing Patient-Specific Repairing Thin Implant for Zygomaticomaxil…
doi:10.1109/icbme61513.2023.10488647	The Role of Lingual Frame on Stability of Mandibular Subperiosteal Implant: A Patient-Spe…
doi:10.5137/1019-5149.jtn.49659-25.3	Design of patient-specific lattice cages and finite element analysis in spinal fusion sur…
doi:10.3390/app12063003	Biomechanical Analysis of Patient-Specific Temporomandibular Joint Implant and Comparison…
doi:10.1093/jcde/qwab047	Design process of patient-specific osteosynthesis plates using topology optimization
doi:10.3390/designs8020031	Finite Element Analysis of Patient-Specific Cranial Implants under Different Design Param…
doi:10.1177/09544119221114729	Design of patient specific basal dental implant using Finite Element method and Artificia…
doi:10.1007/978-981-97-0900-7_18	Comparative Study of a Patient-Specific TMJ Implant-Finite Element Analysis Approach
doi:10.4015/s1016237223500175	DESIGN OF PATIENT SPECIFIC HIP PROSTHESIS BASED ON FINITE ELEMENT ANALYSIS: A COMPARATIVE…
doi:10.21495/em2026-97	Finite Element Modeling of Patient-Specific Mandibular Plates for Condylar Fracture
doi:10.1007/s40033-023-00447-0	Homogeneous and Heterogeneous Modeling of Patient-Specific Hip Implant Under Static and D…
doi:10.1016/j.medengphy.2025.104321	Blinded prediction of custom-made pelvic implant failure using patient-specific finite el…
doi:10.1109/icbme68496.2025.11392468	Design and Biomechanical Comparison of a Patient-Specific Anatomical Plate Versus Convent…
doi:10.3390/app10124098	Patient-Specific 3-Dimensional Printing Titanium Implant Biomechanical Evaluation for Com…
doi:10.1115/1.4062509	Design and Development of Patient-Specific Miniplate for the Treatment of Maxillofacial F…
doi:10.1097/scs.0000000000013117	Finite Element Analysis of Conventional and Patient-Specific Fixation Systems in Large Ma…
doi:10.3390/app122010569	Topology Optimization of Patient-Specific Custom-Fit Distal Tibia Plate: A Spiral Distal…
doi:10.2139/ssrn.6079453	Deterministic and probabilistic finite element analysis framework for patient-specific ma…
doi:10.1016/j.dental.2024.07.008	Design and optimization of a novel patient-specific subperiosteal implant additively manu…
doi:10.3390/cryst15070650	Development of Patient-Specific Lattice Structured Femoral Stems Based on Finite Element…
doi:10.1515/cdbme-2016-0025	Patient specific root-analogue dental implants – additive manufacturing and finite elemen…
doi:10.3390/app152212099	Topology Optimization of a Patient-Specific Femoral Component for Total Knee Endoprosthes…
doi:10.1007/s12663-023-01950-3	Comparison of Two Types of Patient Specific Implants (PSI) and Quad Zygoma Implant (QZI)…
doi:10.1109/cict67193.2025.11399135	AI-Assisted Analysis of Biomaterials for Patient-Specific Implant Design and Fabrication

ID	Title
doi:10.1016/j.jcms.2015.10.004	The feasibility of a custom-made endoprosthesis in mandibular reconstruction: Implant des…
doi:10.20535/2521-1943.2025.9.2(105).336223	Finite element analysis methodology of patient-specific revision knee endoprostheses base…
doi:10.4172/2577-0268-c1-002	Patient specific implant workflow
doi:10.1109/icbme68496.2025.11392524	Patient-Specific TMJ Implants: A Finite Element Study on Placement and Material Effects
doi:10.1007/s13770-025-00730-z	Strategies for the Patient-Specific Implant Angle of Bone Scaffolds Using Optimization
doi:10.1097/scs.0000000000012936	Assessment of Accuracy and Orbital Volume Using Patient-Specific Titanium Implant Versus…
doi:10.1109/icbme64381.2024.10894810	Patient-Specific Anatomical Plate Outperforms Commercial Plate in Finite Element Analysis…
doi:10.3390/app15105384	Biomechanical Optimization of Lumbar Fusion Cages with a Porous Design: A Finite Element…
doi:10.1016/j.procir.2016.01.078	Optimization of Patient-Specific Design of Medical Implants for Manufacturing
doi:10.1055/s-0034-1395385	Finite Element Analysis of Patient-Specific Condyle Fracture Plates: A Preliminary Study
doi:10.1016/j.prosdent.2026.02.038	Osseointegration dynamics of mandibular patient-specific implants: A mechanoregulation-ba…
doi:10.46298/mbj.16190	From Anatomical To Biomechanical Personalization: Comparing Patient-Specific Implants And…
doi:10.1016/j.ijom.2019.03.488	Topology optimization of patient-specific mandibular reconstruction plates and physical v…
doi:10.1109/embc48229.2022.9871069	CranGAN: Adversarial Point Cloud Reconstruction for patient-specific Cranial Implant Desi…
doi:10.1007/s00366-020-01090-z	Symbolic regression metamodel-based optimal design of patient-specific spinal implant (pe…
doi:10.1016/j.jmbbm.2023.106173	3D printed patient-specific fixation plates for the treatment of slipped capital femoral…
doi:10.1016/j.promfg.2021.06.090	Image Processing-based Method for Automatic Design of Patient-Specific Cranial Implant fo…
doi:10.1142/s0219519415400527	PRESURGERY DESIGN AND BIOMECHANICAL EVALUATION OF PATIENT-SPECIFIC KNEE IMPLANT
doi:10.1515/bmt-2023-0017	Parameter-based patient-specific restoration of physiological knee morphology for optimiz…
doi:10.1109/etcm.2018.8580310	Pre-operative patient-specific alloplastic implant design and manufacturing: cranioplasty…
doi:10.1115/1.4071170	Topology Optimization of a Hip Implant for Additive Manufacturing
pmid:32919821	Accuracy of maxillary repositioning surgery using CAD/CAM customized surgical guides and…
pmid:29496055	Three-dimensional printing of patient-specific surgical plates in head and neck reconstru…
pmid:27193477	Is a computer-assisted design and computer-assisted manufacturing method for mandibular r…
pmid:27107476	Morphological results of customized microvascular mandibular reconstruction: A comparativ…
pmid:25843949	Multicenter study on the use of patient-specific CAD/CAM reconstruction plates for mandib…
pmid:25434288	Accuracy of fibular sectioning and insertion into a rapid-prototyped bone plate, for mand…
pmid:31843280	In-House Surgeon-Led Virtual Surgical Planning for Maxillofacial Reconstruction.
pmid:34328575	Polyetheretherketone patient-specific implants (PPSI) for the reconstruction of two diffe…
pmid:32858611	Mandibular Angle Contouring Using Porous Polyethylene Stock or PEEK-based Patient Specifi…
pmid:37863724	Accuracy and influencing factors of maxillary and mandibular repositioning using pre-bent…
pmid:34907951	Surgical Accuracy of Positioning the Maxilla in Patients With Skeletal Class II Malocclus…
pmid:33941394	Accuracy of mandibular proximal segment position using virtual surgical planning and cust…
pmid:30443778	Customized virtual surgical planning in bimaxillary orthognathic surgery: a prospective r…
pmid:27769722	The use of patient-specific implants in orthognathic surgery: A series of 32 maxillary os…
pmid:26432286	A technique for developing CAD geometry of long bones using clinical CT data.
pmid:26700554	A patient-specific guide for optimizing custom-made glenoid implantation in cases of seve…
pmid:35007501	Do Patient-Specific Implants Decrease Complications and Increase Orbital Volume Reconstru…
pmid:27519662	A prospective multicenter study to compare the precision of posttraumatic internal orbita…

ID	Title
pmid:36202719	Postoperative skeletal stability at the one-year follow-up after splintless Le Fort I ost…
pmid:36116544	What Is the Accuracy of Bimaxillary Orthognathic Surgery Using Occlusally-Based Guides an…
pmid:35760659	Custom made cutting guides and osteosynthesis plates versus CAD/CAM occlusal splints in p…
pmid:35151639	Comparison of the Accuracy of Maxillary Positioning With Interim Splints Versus Patient-S…
pmid:33277160	Accuracy of splint vs splintless technique for virtually planned orthognathic surgery: A…
pmid:32156446	Do patient-specific cutting guides and plates improve the accuracy of maxillary repositio…
pmid:38423069	Comprehensive functional outcome analysis and importance of bone remodelling on personali…
pmid:35107617	Customized cost-effective polymethylmethacrylate cranioplasty: a cosmetic comparison with…
pmid:33581784	Evaluation of implant properties, safety profile and clinical efficacy of patient-specifi…
pmid:33418118	3D-Printer-Assisted Patient-Specific Polymethyl Methacrylate Cranioplasty: A Case Series…
pmid:30950944	Cost-Effective Technique of Fabrication of Polymethyl Methacrylate Based Cranial Implant…
pmid:30732480	Polymethylmethacrylate cranioplasty using low-cost customised 3D printed moulds for crani…
pmid:30676441	Polymethyl Methacrylate in Patient-Specific Implants: Description of a New Three-Dimensio…
pmid:30264941	Evaluation of locally manufactured patient-specific custom made implants for cranial defe…
pmid:29905512	Cranioplasty with a low-cost customized polymethylmethacrylate implant using a desktop 3D…
pmid:28011181	Patient-specific polymethylmethacrylate prostheses for secondary reconstruction of large…
pmid:26594999	Customized Titanium Mesh for Repairing Cranial Defects: A Method With Comprehensive Evalu…
pmid:24909393	Comparison of polyetheretherketone and titanium cranioplasty after decompressive craniect…
doi:10.1016/j.eng.2020.02.019	A Systematic Approach for Making 3D-Printed Patient-Specific Implants for Craniomaxillofa…
doi:10.1016/j.addma.2020.101137	Additive manufacturing of fracture fixation implants: Design, material characterization,…
title:0ea15fab4c8b9f62	COMPARISON OF ONE– AND TWO–PIECE PATIENT SPECIFIC IMPLANTS FOR VARIOUS MOVEMENTS OF MAXIL…
doi:10.1016/j.engfailanal.2021.105353	Failure analysis of an in-vivo fractured patient-specific Ti6Al4V mandible reconstruction…
doi:10.33612/diss.1426903730	Patient-specific implants in trauma surgery
doi:10.1177/14644207221104875	Optimizing cranial implant and fixture design using different materials in cranioplasty
doi:10.2139/ssrn.5936726	Evaluate the Feasibility of Generative Designed Patient-Specific Implants for Orthognathi…
doi:10.65204/djms-mfba-p	pdf Multi-Factor Biomechanical Assessment of Patient Specific Cranial Implants Using Fini…
doi:10.1007/s11665-024-09633-x	Patient-Specific Cranial Implant Fabrication: Evaluating Single-Point Incremental Forming…
doi:10.1177/14644207231155761	Designing cranial fixture shapes and topologies for optimizing PEEK implant thickness in…
doi:10.1016/j.stlm.2023.100107	Development of a topologically optimized patient-specific mandibular reconstruction impla…
doi:10.1007/978-3-030-88465-9_50	Developing a Methodology for Design of Patient-Specific Plate Type Implants and Defining…
doi:10.48550/arxiv.2406.17487	Anatomically and mechanically conforming patient-specific spinal fusion cages designed by…
doi:10.1515/cdbme-2025-0137	Enhanced Clinical CT Image Resolution through Multi-Planar Reconstruction for Improved Bo…
doi:10.55981/metalurgi.2023.738	Comparative Studies Simulation Software for Bone Plate Compression
doi:10.5604/01.3001.0054.8694	Advancements in Maxillofacial Reconstruction: Design and Manufacturing using Electron Bea…
doi:10.1080/17452759.2026.2653335	From virtual design to physical prototype: topology-optimised patient-specific mandibular…
doi:10.1109/raics.2018.8635093	Bio-Modelling, Design, Analyses and Prototyping of Patient-Specific Medical Implants

ID	Title
	Usin…
doi:10.62791/20368	Computational modeling of material and structures for biomedical applications
doi:10.1177/14644207241312947	Optimising surgical efficiency by designing lightweight skull-PSI assemblies and curved f…
doi:10.5281/zenodo.19542555	Biomechanics of Spinal Implants
doi:10.5281/zenodo.19542554	Biomechanics of Spinal Implants
title:f8e0ddb52098db61	Design and optimisation of an additively manufactured patient-specific partial mandible r…
doi:10.1016/j.ijsolstr.2025.113776	Integrated nonlinear biomechanical modeling, topology optimization, and LPBF process simu…
title:bef7a4d6ad704fa2	Biomechanics of alloplastic mandible reconstruction using biomaterials: The effect of imp…
doi:10.1302/1358-992x.2025.8.050	IN SILICO ANALYSIS OF PROXIMAL HUMERUS FRACTURE FIXATIONS TOWARDS IMPROVED TREATMENTS
title:a84f348e3c0fa2f7	Numerical and Experimental Investigation of a New Load-sharing Implant for Early-onset Kn…
title:7df7bc11148b9050	DESIGN OPTIMISATION OF PATIENT-SPECIFIC DISTAL HUMERAL HEMIARTHROPLASTY IMPLANTS FOR NATU…
doi:10.26226/morressier.5c8f90a0b5d368000a26b6c7	1884 - Can the Pain Catastrophizing Scale Predict Patient Pain and Discharge Disposition…
title:521c675cfacec9e5	Design, optimization, and virtual validation of personalized mandibular prostheses using…
doi:10.25904/1912/5981	Designing and manufacturing TPMS structures for temporary paediatric internal fixation de…
doi:10.1016/j.ijom.2025.04.703	Deep Learning Based Automatic 3D Segmentation of The Jaws from CBCT for Patient-Specific…
doi:10.1117/12.2580719	An online platform for automatic skull defect restoration and cranial implant design
doi:10.1109/access.2024.3415173	CraNeXt: Automatic Reconstruction of Skull Implants With Skull Categorization Technique
doi:10.1145/3774521.3774531	RibCageImp: A Deep Learning Framework for 3D Ribcage Implant Generation
doi:10.48550/arxiv.2006.00980	An Online Platform for Automatic Skull Defect Restoration and Cranial Implant Design
doi:10.31661/jbpe.v0i0.2504-1912	Automated CT Image Reconstruction of Zygomatic Fractures for Preoperative Planning and Na…
doi:10.3390/app16104763	Optimizing 3D UNet Parameters for Cranial Defect Reconstruction
doi:10.7939/83976	Data-driven Design of Customized Biomimetic Cranial Implants: Integrating Microstructural…
doi:10.1055/s-0043-1767095	In-silico and ex-vivo validation of semi-automatic segmentation and patient specific impl…
title:973472df08a69edc	Complications, associated risk factors, and stability of the outcome of orthognathic surg…
doi:10.1371/journal.pone.0325587.t001	Segmentation accuracy.
title:62dd0a1a93fe84af	Segmentation Accuracy of the Knee Joint Using MRI Technology
doi:10.1007/s42242-023-00259-x	Customized scaffolds for large bone defects using 3D-printed modular blocks from 2D-medic…
doi:10.1109/isda.2015.7489206	Multi-agent 3D reconstruction of human femur from MR images
title:42023028a9de6872	Development of a Patient-Specific Plate for Hoffa Fracture:: CT-Based Segmentation and Ad…
title:837ea219c36fe570	AUTOMATIC RECONSTRUCTION OF LARGE ACETABULAR BONE DEFECTS USING STATISTICAL SHAPE MODELS
doi:10.1371/journal.pone.0325587.t003	Full bone reconstruction accuracy.
title:f9899d4ceab458c1	Supporting Technologies in the Assessment and Reconstruction of Bony Orbit
doi:10.64951/jmdnt.2026.2.16	Two-Center Prospective Clinical Feasibility Study Evaluating AI-Guided 3D-Printed Surgica…
doi:10.1055/s-0037-1600868	Improved Technology for Navigation-Guided Orbital Surgery and Reconstruction
doi:10.1302/1358-992x.2024.18.082	PATIENT-SPECIFIC TIBIAL IMPLANTS: ALGORITHMIC DESIGN PROCESSES FOR CLINICAL CASES
doi:10.1055/s-0045-1803877	Implementing Augmenting Reality in Craniomaxillofacial Reconstructive Surgery: An Early I…
doi:10.1142/s0219519418500240	DESIGN OF A MAXILLOFACIAL PROSTHESIS BASED ON TOPOLOGY OPTIMIZATION
doi:10.1115/1.4049193	Shape Accuracy and Surface Quality of Additively Manufactured, Optimized, Patient-

ID	Title
	Specifi…
title:64d0cb470bccee10	General deformable 3D hip prosthesis model creation for patient-specific implant design a…
doi:10.17918/00010717	Design envelope for future acetabular implant reconstructions and statistical shape model…
title:cd08f82d341247aa	Retrospective study on mandible morphology towards improving implant design
doi:10.1302/1358-992x.2024.18.002	DOES SHAPE SYMMETRY EXIST IN THE LUNATE BONE? TOWARDS DEVELOPMENT OF A PATIENT-SPECIFIC L…
doi:10.1088/1757-899x/747/1/012076	Comparative analysis of the revision acetabular customized implant position by finite ele…
doi:10.2139/ssrn.4484792	Finite Element Analysis of a Customized Implant in Pmma Coupled with the Cranial Bone
doi:10.1016/j.matdes.2023.111605	Patient-specific Ti-6Al-4V lattice implants for critical-sized load-bearing bone defects…
doi:10.1007/s40430-018-1425-9	Design and additive manufacturing of patient-specific cranial and pelvic bone implants fr…
doi:10.15593/rjbiomech/2021.4.07	Finite element analysis of the stress state of the hip joint endoprothesis while walking
doi:10.2139/ssrn.4499911	Design of Customized Implants and 3d Printing of Symmetric and Asymmetric Cranial Cavities
doi:10.1109/icbme51989.2020.9319325	Design and fabrication of mandibular customized implants by CAD/CAM method and 3-D printi…
doi:10.17615/s5er-hk90	Custom-designed orthopedic implants evaluated using finite element analysis of patient-sp…
doi:10.1007/978-981-15-0199-9_44	Design and Analysis of Customized Hip Implant Using Finite Element Method
doi:10.1142/s021951942340016x	FINITE ELEMENT ANALYSIS OF CUSTOMIZED KNEE IMPLANTS BY VARYING LOADS DURING FLEXION-EXTEN…
doi:10.3390/app142210396	Inclusion of Muscle Forces Affects Finite Element Prediction of Compression Screw Pullout…
doi:10.7939/r36q1sn87	The Development and Analysis of a Generic Talus Bone Prosthetic
doi:10.1016/j.irbm.2017.01.003	Computer Assisted Design and Analysis of Customized Porous Plate for Mandibular Reconstru…
doi:10.1016/j.procir.2022.06.065	Mechanical simulation considering anisotropy of trabecular scaffolds for 3D-printed biomi…
doi:10.1007/s40846-018-0370-5	Implementation of Computer-Assisted Design, Analysis, and Additive Manufactured Customize…
doi:10.3390/app9122513	Fabrication and Analysis of a Ti6Al4V Implant for Cranial Restoration
doi:10.1088/1757-899x/986/1/012035	Some aspects of custom-made 3d-printed hip joint implant structural simulation
doi:10.3390/technologies12080125	Computer Simulation-Based Multi-Objective Optimisation of Additively Manufactured Cranial…
doi:10.1088/1757-899x/1306/1/012017	Biomechanical evaluation of a custom-made mandibular plate
title:7ca24a48131b4fb5	Computational Framework for Designing Patient-specific, 3D-printable, and Stress-shieldin…
doi:10.1007/s40964-024-00866-9	Development of customized tibial implant with advanced architected materials utilizing se…
doi:10.1016/j.procs.2024.04.312	Automatic Skull Shape Completion of Defective Skulls Using Transformers for Cranial Impla…
doi:10.1016/j.procir.2024.08.047	Optimizing Orthopaedic Bone Plates for Directed Energy Deposition with Generative Design…
doi:10.1016/j.identj.2026.111095	Feasibility of Generatively Designed Patient-Specific Implants for Orthognathic Surgery:…
doi:10.1007/978-3-031-81673-4_65	Refining Bone Plate Design for Proximal Tibia Fractures: A Comparative Evaluation of Meth…
doi:10.5204/thesis.eprints.251499	Design and Fabrication of Multi-Structural Scaffolds for the Treatment of Critical-Sized…
doi:10.1016/j.adoms.2021.100229	Reconstruction of large mandibular bone defects extended to the condyle using patient-spe…
pmid:33705044	Accuracy of Patient-Specific Meshes as a Reconstruction of Orbital Floor Blow-Out Fractur…
doi:10.1016/j.joms.2021.08.040	Clinical Accuracy of Bimaxillary Surgery Completed with Custom Prefabricated Splints vs.…
doi:10.33612/diss.1135250381	Virtual surgical planning and patient specific osteosynthesis in Orthognathic Surgery
pmid:34215417	Clear Aligner Orthognathic Splints (CAOS) and Custom Maxillary Fixation Plates for Surger…
doi:10.1016/j.jdent.2026.106453	Virtual surgical planning for midface reconstruction using osseous free flaps
pmid:42055482	Patient-specific implants in minimally invasive orthognathic surgery: A case series

ID	Title
doi:10.5281/zenodo.19017940	PATIENT-SPECIFIC IMPLANTS VERSUS CONVENTIONAL PLATING SYSTEMS IN GENIOPLASTY: A COMPREHEN…
doi:10.5281/zenodo.19017939	PATIENT-SPECIFIC IMPLANTS VERSUS CONVENTIONAL PLATING SYSTEMS IN GENIOPLASTY: A COMPREHEN…
doi:10.33612/diss.835211514	Design optimisation of patient-specific implants and total joint replacements in oral and…
doi:10.5281/zenodo.19562531	CLINICAL AND FUNCTIONAL OUTCOMES OF PATIENT-SPECIFIC 3D-PRINTED TITANIUM IMPLANTS IN RECO…
doi:10.5281/zenodo.19562530	CLINICAL AND FUNCTIONAL OUTCOMES OF PATIENT-SPECIFIC 3D-PRINTED TITANIUM IMPLANTS IN RECO…
doi:10.26041/fhnw-14094	Fronto-orbital advancement with patient-specific 3D-printed implants and robot-guided las…
pmid:40769801	Custom-made fibular cradle plate to optimise bone positioning for implant placement, rest…
doi:10.1177/27325016251352171	Zygomatic Osteotomy and Repositioning: Current Thinking and Use of Technological Aids
doi:10.33612/diss.231382708	Unlocking the third dimension of acetabular fracture surgery
doi:10.58837/chula.the.2024.1000	End-to-end development of mandible reconstruction using machine learning
doi:10.64951/jmdnt.2026.1.04	Does Surgical Sequencing Still Mater in Fully Digital Bimaxillary Orthognathic Surgery? A…
doi:10.5281/zenodo.18811308	QUANTITATIVE EVALUATION OF SURGICAL FIDELITY IN VSP-GUIDED MANDIBULAR RECONSTRUCTION: A R…
doi:10.5281/zenodo.18811309	QUANTITATIVE EVALUATION OF SURGICAL FIDELITY IN VSP-GUIDED MANDIBULAR RECONSTRUCTION: A R…
doi:10.53846/goediss-10683	Vergleichende Analyse zweier Operationsplanungsverfahren für die Unterkieferrekonstruktio…
doi:10.6084/m9.figshare.c.8613367	Assessment of plan–execution fidelity in a fully digital workflow using custom-made plate…
doi:10.6084/m9.figshare.c.8613367.v1	Assessment of plan–execution fidelity in a fully digital workflow using custom-made plate…
doi:10.1016/j.matdes.2025.113668	Establishment of design guidelines for patient-specific 3D-printed reconstruction implant…
title:9053f855561730d1	Automated computer-aided design of cranial implants: a deep learning approach
doi:10.1109/access.2026.3663618	Tailoring Patient-Specific Cranial Implants for Bone Reconstruction via End-to-End Deep L…
doi:10.48550/arxiv.2204.06310	Deep Learning-based Framework for Automatic Cranial Defect Reconstruction and Implant Mod…
doi:10.48550/arxiv.2406.06372	Improving Deep Learning-based Automatic Cranial Defect Reconstruction by Heavy Data Augme…
doi:10.47749/t/unicamp.2025.1489775	Automatic generation of customized cranial implants through deep learning
doi:10.1007/s40964-026-01767-9	Rapid design and additive manufacturing of precise cranial implants using deep learning a…
doi:10.5445/ir/1000190624	Tailoring Patient-Specific Cranial Implants for Bone Reconstruction via End-to-End Deep L…
doi:10.1109/iccvw60793.2023.00114	Deep Generative Networks for Heterogeneous Augmentation of Cranial Defects
doi:10.48550/arxiv.2108.05269	Learning to Rearrange Voxels in Binary Segmentation Masks for Smooth Manifold Triangulati…
doi:10.1109/tiptekno68206.2025.11270164	Patch-Based Deep Learning Approaches for Automated 3D Skull Segmentation in CT Imaging
doi:10.48550/arxiv.1911.08805	Segmentation of Defective Skulls from CT Data for Tissue Modelling
doi:10.48550/arxiv.2308.04883	Deep Generative Networks for Heterogeneous Augmentation of Cranial Defects
doi:10.18502/jcr.v10i4.15313	Numerical Study of Mechanical Development of Patient-Specific Mandibular Reconstruction I…
title:7c999d4b2b066c35	Next-Generation Scaffold-Based Implants for Mandibular Reconstructions: Patient-Specific…
doi:10.2139/ssrn.7324371	Patient-specific biomechanical analysis of titanium plate failure in mandibular reconstru…
doi:10.1016/j.rineng.2026.110026	Biomechanical evaluation of mesh-based mandibular reconstruction plates and their implica…
title:791924899ea2aa08	Sacral anchoring of the LUMiC prosthesis: Development of a protocol to in vitro evaluate…

S1.6 Charting agreement on the seeded sample and adjudicated corrections

Charting agreement on the seeded 60-record sample

Records compared: 60 (sample of 60; missing from either charter: 0)

Field	Raw agreement	Kappa
eligible	0.87	0.70
excl_code	0.85	0.47
region	0.90	0.86
pillars	0.83	0.73
tier	0.87	0.84
comparator	0.85	0.71
ai_independent_eval	1.00	1.00
ai_clinical_eval	1.00	1.00
code_or_data	0.70	0.48

ID	Field	Charted value	Corrected value	Reason
pmid:34441806	mech_result	(missing field, 29-field row)	.	format repair: charter omitted the mech_result field, shifting the time and clinical results one column left; all other values unchanged
pmid:29408349	eligible	yes (pillars comp, comp_method .)	unclear	abstract-only record; the abstract names no design or planning method, so no pillar is established; charted unclear per the unclear-never-no rule pending full text
pmid:33647729	excl_code	not_psi	no_method	the stated method generated bone–screw FE models at different image resolutions without designing the implant
pmid:30629776	eligible	no	yes	the personalized plate and traditional Y-shaped plate were built and quantitatively compared by finite-element analysis
pmid:30629776	excl_code	case_report	.	the single clinical case accompanies an independently evaluable finite-element comparison of the personalized implant
pmid:30629776	region	.	upper_limb_other	the implant was designed for a distal humerus fracture
pmid:30629776	pillars	.	comp	the personalized and traditional plates were analyzed by the finite-element method
pmid:30629776	tier	.	in_silico	the qualifying evaluation comprised finite-element models of the humerus and two plates
pmid:30629776	comparator	.	yes	the personalized plate was compared with a traditional Y-shaped compression plate
pmid:30629776	code_or_data	.	unclear	the abstract reports the finite-element study but gives no code or data availability information
doi:10.1177/09544119221114729	eligible	yes	unclear	the abstract varies thread geometry and categorical bone conditions but does not state that implant geometry derives from one individual's anatomy
pmid:33170352	eligible	no	yes	eleven patients received custom-made cones and three patient-specific surgical configurations were evaluated by FEA
pmid:33170352	excl_code	no_method	.	the study reports both treatment of eleven patients and quantitative biomechanical evaluation of their custom-made cones
pmid:33170352	region	.	knee	the cones reconstructed femoral and tibial defects

ID	Field	Charted value	Corrected value	Reason
				during revision total knee arthroplasty
pmid:33170352	pillars	.	comp	bone geometries and implanted configurations from three clinical cases were replicated and analyzed by FEA
pmid:33170352	tier	.	clinical_series	eleven patients underwent knee revision using sixteen custom-made cones
pmid:33170352	compara tor	.	yes	custom-made cones were compared by FEA with cemented and cementless stems
pmid:33170352	code_or _data	.	unclear	the supplied abstract and repository metadata contain no code or data availability statement
pmid:42317473	excl_co de	no_method	not_psi	The study constructed a population SSM, while implant design was described only as a potential application.
pmid:26363532	excl_co de	no_method	not_psi	The study generated virtual femurs, with implant design mentioned only as a possible use.
pmid:35995899	excl_co de	no_method	not_psi	The evaluated CNN generated improved CT slices but did not design or evaluate a patient-specific implant.
doi:10.1016/j.finel.2017.07.010	pillars	imaging+comp	comp	The title establishes finite-element modelling, but does not establish evaluation of a distinct imaging pipeline.
doi:10.14445/22315381/ijett-v70i9p235	tier	.	in_silico	the title explicitly identifies the study as a patient-specific finite element analysis
pmid:25911122	tier	clinical_compar ative	clinical_series	seven consecutive patients formed one cohort whose outcomes were compared with the virtual plan
pmid:36401069	compara tor	no	yes	the postoperative CBCT model was superimposed on and compared with the virtual surgical plan
pmid:29088629	compara tor	no	.	the title alone does not state whether implant configurations were compared
pmid:39182846	code_or _data	none	unclear	the abstract provides no code or data availability statement
pmid:40985989	code_or _data	none	unclear	The only identified supporting file is a photograph, and no code or data availability statement is given.
pmid:28299273	code_or _data	none	unclear	The provided full text contains no code or data availability statement.
pmid:38793041	code_or _data	unclear	none	the only supplementary materials identified are “Figures S1 and S2,” with no code or study data reported as available
pmid:36401069	compara tor_type	.	alternative_method	adjudication set comparator yes (design compared against an alternative method per the adjudicator’s reason) without a type; type assigned from the same reason
pmid:33170352	credibili ty	(malformed string from adjudicator)	u for each unsettled item	format repair only
pmid:36401069	compara tor_type	alternative_met hod	ground_truth	comparison is postoperative CBCT against the virtual plan as reference
pmid:33170352	comp_m ethod,n_ patients, n_units, compara tor_type ,implant _type,ye ar	.	fea; 11; 3; alternative_method; custom-made cones; year from record	completion of the row after the adjudicator flipped eligibility, values taken from the adjudicator’s quoted text (eleven patients, three configurations evaluated by FEA against stems)
doi:10.20944/preprints202501.2 283.v1	eligible	yes	no (duplicate_preprint)	preprint version of pmid:40428165; journal version retained (amendment A3)
doi:10.21203/rs.3.rs-4145453/v1	eligible	yes	no	preprint version of pmid:39693002; journal version

ID	Field	Charted value	Corrected value	Reason
			(duplicate_preprint)	retained (amendment A3)
doi:10.21203/rs.3.rs-4241140/v1	eligible	yes	no (duplicate_preprint)	preprint version of pmid:39242632; journal version retained (amendment A3)
doi:10.21203/rs.3.rs-4160020/v1	eligible	yes	no (duplicate_preprint)	preprint version of pmid:39242632; journal version retained (amendment A3)
doi:10.20944/preprints202107.0110.v1	eligible	yes	no (duplicate_preprint)	preprint version of pmid:34441859; journal version retained (amendment A3)
doi:10.21203/rs.3.rs-1290007/v1	eligible	yes	no (duplicate_preprint)	preprint version of pmid:35264178; journal version retained (amendment A3)
doi:10.21203/rs.3.rs-1590857/v1	eligible	yes	no (duplicate_preprint)	preprint version of pmid:35869276; journal version retained (amendment A3)
doi:10.21203/rs.3.rs-858174/v1	eligible	yes	no (duplicate_preprint)	preprint version of pmid:36401069; journal version retained (amendment A3)
doi:10.21203/rs.3.rs-145748/v1	eligible	yes	no (duplicate_preprint)	preprint version of pmid:34834588; journal version retained (amendment A3)
doi:10.21203/rs.3.rs-94887/v2	eligible	yes	no (duplicate_preprint)	preprint version of pmid:33761991; journal version retained (amendment A3)
doi:10.21203/rs.3.rs-262939/v1	eligible	yes	no (duplicate_preprint)	preprint version of pmid:34521397; journal version retained (amendment A3)
doi:10.1101/2021.07.22.21260825	eligible	yes	no (duplicate_preprint)	preprint version of pmid:35037088; journal version retained (amendment A3)
doi:10.21203/rs.3.rs-9337194/v1	eligible	yes	no (duplicate_preprint)	preprint version of pmid:42479267; identical four-author list (amendment A3)
doi:10.20944/preprints202307.1681.v1	eligible	yes	no (duplicate_preprint)	preprint version of pmid:38793041; identical four-author list (amendment A3)
doi:10.21203/rs.3.rs-8848013/v1	eligible	yes	no (duplicate_preprint)	preprint version of pmid:42032041; same first author, same design question and implant (title similarity 0.76); judged the journal version (amendment A3)
pmid:34441806	(full row)	(partial correction)	(re-charted row)	complete re-charting by sol after adjudication flipped eligibility; eligible=yes
pmid:30629776	(full row)	(partial correction)	(re-charted row)	complete re-charting by sol after adjudication flipped eligibility; eligible=yes
pmid:33170352	(full row)	(partial correction)	(re-charted row)	complete re-charting by sol after adjudication flipped eligibility; eligible=yes
pmid:42290023	(full row)	eligible unclear	eligible unclear	re-assessment of an unclear record by a second sol pass from the same instructions
pmid:42090197	(full row)	eligible unclear	eligible unclear	re-assessment of an unclear record by a second sol pass from the same instructions
pmid:40288205	(full row)	eligible unclear	eligible unclear	re-assessment of an unclear record by a second sol pass from the same instructions
pmid:40209337	(full row)	eligible unclear	eligible unclear	re-assessment of an unclear record by a second sol pass from the same instructions
pmid:39603155	(full row)	eligible unclear	eligible no	re-assessment of an unclear record by a second sol pass from the same instructions
pmid:39487496	(full row)	eligible unclear	eligible yes	re-assessment of an unclear record by a second sol pass from the same instructions
pmid:38776646	(full row)	eligible unclear	eligible unclear	re-assessment of an unclear record by a second sol pass from the same instructions
pmid:38246644	(full row)	eligible unclear	eligible unclear	re-assessment of an unclear record by a second sol pass from the same instructions
pmid:38143713	(full row)	eligible unclear	eligible no	re-assessment of an unclear record by a second sol pass from the same instructions
pmid:37797593	(full row)	eligible unclear	eligible unclear	re-assessment of an unclear record by a second sol pass from the same instructions
pmid:37757617	(full row)	eligible unclear	eligible unclear	re-assessment of an unclear record by a second sol pass from the same instructions

ID	Field	Charted value	Corrected value	Reason
pmid:37643059	(full row)	eligible unclear	eligible unclear	re-assessment of an unclear record by a second sol pass from the same instructions
pmid:37216926	(full row)	eligible unclear	eligible unclear	re-assessment of an unclear record by a second sol pass from the same instructions
pmid:36965217	(full row)	eligible unclear	eligible yes	re-assessment of an unclear record by a second sol pass from the same instructions
pmid:36541118	(full row)	eligible unclear	eligible yes	re-assessment of an unclear record by a second sol pass from the same instructions
pmid:36152972	(full row)	eligible unclear	eligible yes	re-assessment of an unclear record by a second sol pass from the same instructions
pmid:35835610	(full row)	eligible unclear	eligible no	re-assessment of an unclear record by a second sol pass from the same instructions
pmid:33524959	(full row)	eligible unclear	eligible yes	re-assessment of an unclear record by a second sol pass from the same instructions
pmid:33494021	(full row)	eligible unclear	eligible no	re-assessment of an unclear record by a second sol pass from the same instructions
pmid:32518971	(full row)	eligible unclear	eligible yes	re-assessment of an unclear record by a second sol pass from the same instructions
pmid:32505528	(full row)	eligible unclear	eligible yes	re-assessment of an unclear record by a second sol pass from the same instructions
pmid:32320044	(full row)	eligible unclear	eligible yes	re-assessment of an unclear record by a second sol pass from the same instructions
pmid:32242531	(full row)	eligible unclear	eligible no	re-assessment of an unclear record by a second sol pass from the same instructions
pmid:31655451	(full row)	eligible unclear	eligible no	re-assessment of an unclear record by a second sol pass from the same instructions
pmid:29191076	(full row)	eligible unclear	eligible yes	re-assessment of an unclear record by a second sol pass from the same instructions
pmid:29088629	(full row)	eligible unclear	eligible no	re-assessment of an unclear record by a second sol pass from the same instructions
pmid:28888931	(full row)	eligible unclear	eligible yes	re-assessment of an unclear record by a second sol pass from the same instructions
pmid:28585457	(full row)	eligible unclear	eligible no	re-assessment of an unclear record by a second sol pass from the same instructions
pmid:27167515	(full row)	eligible unclear	eligible no	re-assessment of an unclear record by a second sol pass from the same instructions
pmid:25465486	(full row)	eligible unclear	eligible yes	re-assessment of an unclear record by a second sol pass from the same instructions
pmid:41266230	(full row)	eligible unclear	eligible yes	re-assessment of an unclear record by a second sol pass from the same instructions
pmid:41218231	(full row)	eligible unclear	eligible yes	re-assessment of an unclear record by a second sol pass from the same instructions
doi:10.20944/preprints202507.1208.v1	(full row)	eligible unclear	eligible yes	re-assessment of an unclear record by a second sol pass from the same instructions
doi:10.1016/j.ijom.2023.10.293	(full row)	eligible unclear	eligible yes	re-assessment of an unclear record by a second sol pass from the same instructions
doi:10.18502/keg.v2i2.602	(full row)	eligible unclear	eligible no	re-assessment of an unclear record by a second sol pass from the same instructions
doi:10.2139/ssrn.4968323	(full row)	eligible unclear	eligible yes	re-assessment of an unclear record by a second sol pass from the same instructions
doi:10.1016/j.bprint.2025.e00412	(full row)	eligible unclear	eligible yes	re-assessment of an unclear record by a second sol pass from the same instructions
doi:10.1016/j.finel.2017.07.010	(full row)	eligible unclear	eligible no	re-assessment of an unclear record by a second sol pass from the same instructions
doi:10.14445/22315381/ijett-v70i9p235	(full row)	eligible unclear	eligible yes	re-assessment of an unclear record by a second sol pass from the same instructions
doi:10.1109/icbme61513.2023.1	(full	eligible unclear	eligible yes	re-assessment of an unclear record by a second sol

ID	Field	Charted value	Corrected value	Reason
0488647	row)			pass from the same instructions
doi:10.5137/1019-5149.jtn.49659-25.3	(full row)	eligible unclear	eligible no	re-assessment of an unclear record by a second sol pass from the same instructions
doi:10.1007/978-981-97-0900-7_18	(full row)	eligible unclear	eligible yes	re-assessment of an unclear record by a second sol pass from the same instructions
doi:10.21495/em2026-97	(full row)	eligible unclear	eligible yes	re-assessment of an unclear record by a second sol pass from the same instructions
doi:10.1007/s40033-023-00447-0	(full row)	eligible unclear	eligible yes	re-assessment of an unclear record by a second sol pass from the same instructions
doi:10.1016/j.medengphy.2025.104321	(full row)	eligible unclear	eligible no	re-assessment of an unclear record by a second sol pass from the same instructions
doi:10.2139/ssrn.6079453	(full row)	eligible unclear	eligible yes	re-assessment of an unclear record by a second sol pass from the same instructions
doi:10.1016/j.dental.2024.07.008	(full row)	eligible unclear	eligible yes	re-assessment of an unclear record by a second sol pass from the same instructions
doi:10.3390/cryst15070650	(full row)	eligible unclear	eligible no	re-assessment of an unclear record by a second sol pass from the same instructions
doi:10.1007/s12663-023-01950-3	(full row)	eligible unclear	eligible yes	re-assessment of an unclear record by a second sol pass from the same instructions
doi:10.1109/cict67193.2025.11399135	(full row)	eligible unclear	eligible no	re-assessment of an unclear record by a second sol pass from the same instructions
doi:10.1016/j.jcms.2015.10.004	(full row)	eligible unclear	eligible yes	re-assessment of an unclear record by a second sol pass from the same instructions
doi:10.4172/2577-0268-c1-002	(full row)	eligible unclear	eligible no	re-assessment of an unclear record by a second sol pass from the same instructions
doi:10.1109/icbme68496.2025.11392524	(full row)	eligible unclear	eligible yes	re-assessment of an unclear record by a second sol pass from the same instructions
doi:10.1007/s13770-025-00730-z	(full row)	eligible unclear	eligible no	re-assessment of an unclear record by a second sol pass from the same instructions
doi:10.1109/icbme64381.2024.10894810	(full row)	eligible unclear	eligible yes	re-assessment of an unclear record by a second sol pass from the same instructions
doi:10.1016/j.procir.2016.01.078	(full row)	eligible unclear	eligible no	re-assessment of an unclear record by a second sol pass from the same instructions
doi:10.1016/j.prosdent.2026.02.038	(full row)	eligible unclear	eligible no	re-assessment of an unclear record by a second sol pass from the same instructions
doi:10.46298/mbj.16190	(full row)	eligible unclear	eligible yes	re-assessment of an unclear record by a second sol pass from the same instructions
doi:10.1109/embc48229.2022.9871069	(full row)	eligible unclear	eligible yes	re-assessment of an unclear record by a second sol pass from the same instructions
doi:10.1007/s00366-020-01090-z	(full row)	eligible unclear	eligible yes	re-assessment of an unclear record by a second sol pass from the same instructions
doi:10.1016/j.jmbbm.2023.106173	(full row)	eligible unclear	eligible yes	re-assessment of an unclear record by a second sol pass from the same instructions
doi:10.1016/j.promfg.2021.06.090	(full row)	eligible unclear	eligible yes	re-assessment of an unclear record by a second sol pass from the same instructions
doi:10.1109/etcm.2018.8580310	(full row)	eligible unclear	eligible yes	re-assessment of an unclear record by a second sol pass from the same instructions
doi:10.1115/1.4071170	(full row)	eligible unclear	eligible yes	re-assessment of an unclear record by a second sol pass from the same instructions
pmid:27769722	(full row)	eligible unclear	eligible yes	re-assessment of an unclear record by a second sol pass from the same instructions
doi:10.1097/scs.0000000000012936	eligible	yes	no (duplicate_report)	same article as pmid:42183622 (DOI or title identical); the copy with full text, then the PubMed copy, was kept
doi:10.1016/j.prosdent.2026.02.038	eligible	no	no (duplicate_report)	same article as pmid:42020225 (DOI or title identical); the copy with full text, then the PubMed copy, was kept

ID	Field	Charted value	Corrected value	Reason
doi:10.4055/cios25232	eligible	no	no (duplicate_report)	same article as pmid:41938871 (DOI or title identical); the copy with full text, then the PubMed copy, was kept
doi:10.5137/1019-5149.jtn.49659-25.3	eligible	no	no (duplicate_report)	same article as pmid:41553224 (DOI or title identical); the copy with full text, then the PubMed copy, was kept
doi:10.3390/ma18214822	eligible	yes	no (duplicate_report)	same article as pmid:41227783 (DOI or title identical); the copy with full text, then the PubMed copy, was kept
doi:10.1007/s13770-025-00730-z	eligible	no	no (duplicate_report)	same article as pmid:40512299 (DOI or title identical); the copy with full text, then the PubMed copy, was kept
doi:10.1016/j.medengphy.2025.104321	eligible	no	no (duplicate_report)	same article as pmid:40180533 (DOI or title identical); the copy with full text, then the PubMed copy, was kept
doi:10.4317/medoral.27065	eligible	yes	no (duplicate_report)	same article as pmid:40121675 (DOI or title identical); the copy with full text, then the PubMed copy, was kept
doi:10.4103/njms.njms_155_23	eligible	yes	no (duplicate_report)	same article as pmid:39234141 (DOI or title identical); the copy with full text, then the PubMed copy, was kept
doi:10.1016/j.dental.2024.07.008	eligible	yes	no (duplicate_report)	same article as pmid:39084954 (DOI or title identical); the copy with full text, then the PubMed copy, was kept
doi:10.3390/polym15173620	eligible	no	no (duplicate_report)	same article as pmid:37688247 (DOI or title identical); the copy with full text, then the PubMed copy, was kept
doi:10.1007/s12663-023-01950-3	eligible	yes	no (duplicate_report)	same article as pmid:37534346 (DOI or title identical); the copy with full text, then the PubMed copy, was kept
doi:10.1515/bmt-2023-0017	eligible	no	no (duplicate_report)	same article as pmid:37185164 (DOI or title identical); the copy with full text, then the PubMed copy, was kept
doi:10.3390/ma16010398	eligible	yes	no (duplicate_report)	same article as pmid:36614737 (DOI or title identical); the copy with full text, then the PubMed copy, was kept
doi:10.1109/embc48229.2022.9871069	eligible	yes	no (duplicate_report)	same article as pmid:36085744 (DOI or title identical); the copy with full text, then the PubMed copy, was kept
doi:10.1177/09544119221114729	eligible	unclear	no (duplicate_report)	same article as pmid:35880901 (DOI or title identical); the copy with full text, then the PubMed copy, was kept
doi:10.3390/ma15124342	eligible	yes	no (duplicate_report)	same article as pmid:35744401 (DOI or title identical); the copy with full text, then the PubMed copy, was kept
doi:10.18063/ijb.v8i1.437	eligible	yes	no (duplicate_report)	same article as pmid:35187275 (DOI or title identical); the copy with full text, then the PubMed copy, was kept
doi:10.1097/gox.0000000000004081	eligible	yes	no (duplicate_report)	same article as pmid:35186634 (DOI or title identical); the copy with full text, then the PubMed copy, was kept
doi:10.1038/s41598-021-94980-1	eligible	yes	no (duplicate_report)	same article as pmid:34321582 (DOI or title identical); the copy with full text, then the PubMed copy, was kept
doi:10.1016/j.ijom.2021.05.011	eligible	yes	no (duplicate_report)	same article as pmid:34059405 (DOI or title identical); the copy with full text, then the PubMed copy, was kept
doi:10.1016/j.wneu.2021.01.102	eligible	yes	no (duplicate_report)	same article as pmid:33529765 (DOI or title identical); the copy with full text, then the PubMed copy, was kept

ID	Field	Charted value	Corrected value	Reason
doi:10.1016/j.jmbbm.2020.103700	eligible	yes	no (duplicate_report)	same article as pmid:32279847 (DOI or title identical); the copy with full text, then the PubMed copy, was kept
doi:10.1302/2046-3758.83.bjr-2018-0193.r1	eligible	yes	no (duplicate_report)	same article as pmid:30997041 (DOI or title identical); the copy with full text, then the PubMed copy, was kept
doi:10.1155/2016/8242535	eligible	yes	no (duplicate_report)	same article as pmid:27274727 (DOI or title identical); the copy with full text, then the PubMed copy, was kept
doi:10.1016/j.jcms.2015.10.004	eligible	yes	no (duplicate_report)	same article as pmid:26596361 (DOI or title identical); the copy with full text, then the PubMed copy, was kept
doi:10.1055/s-0034-1395385	eligible	yes	no (duplicate_report)	same article as pmid:26000081 (DOI or title identical); the copy with full text, then the PubMed copy, was kept
doi:10.1186/s41205-025-00260-3	eligible	yes	no (duplicate_report)	same article as pmid:40067641 (DOI or title identical); the copy with full text, then the PubMed copy, was kept
doi:10.21203/rs.3.rs-3280673/v1	eligible	no	no (duplicate_report)	same article as pmid:38233785 (DOI or title identical); the copy with full text, then the PubMed copy, was kept
pmid:36401069	eligible	yes	no (duplicate_report)	same article as doi:10.21203/rs.3.rs-858174/v1 (DOI or title identical); the copy with full text, then the PubMed copy, was kept
title:1ae5734e965933fa	eligible	yes	no (duplicate_report)	same article as pmid:36185133 (DOI or title identical); the copy with full text, then the PubMed copy, was kept
pmid:36152972	eligible	yes	unclear	dental boundary re-adjudication under amendment A2: The text describes additively manufactured subperiosteal implant models but does not state that their geometry was derived from an individual patient’s anatomy
pmid:42210774	eligible	yes	no (not_psi)	dental boundary re-adjudication under amendment A2: The synthetic cohort varied bone quality and loading, with optimisation prescribing parameters such as wider diameters for low-density bone
pmid:39487496	eligible	yes	no (not_psi)	dental boundary re-adjudication under amendment A2: Manufacturer CAD implant models with fixed dimensions were placed into a patient-derived maxillary model rather than anatomy-derived implant geometry
doi:10.1016/j.asoc.2018.01.025	eligible	yes	unclear	dental boundary re-adjudication under amendment A2: The title calls the implant patient-specific but provides no text establishing anatomy-derived geometry rather than parameter optimisation
pmid:35880901	eligible	yes	no (not_psi)	dental boundary re-adjudication under amendment A2: Implants varied only in thread length, diameter, and pitch across strong, weak, and normal basal-bone conditions
pmid:42169041	eligible	yes	no (not_psi)	dental boundary re-adjudication under amendment A2: The six alternative implant configurations were modeled on Visible Human Project tomography rather than an individual patient’s anatomy
pmid:30131272	eligible	yes	unclear	dental boundary re-adjudication under amendment A2: The abstract describes a generic FEA-derived design but does not state whether each implant’s geometry was derived from its recipient’s anatomy
doi:10.1109/icbme61513.2023.10488647	eligible	yes	unclear	dental boundary re-adjudication under amendment A2: The title identifies a patient-specific finite-element study but does not establish that the implant geometry was derived from that patient’s anatomy.

ID	Field	Charted value	Corrected value	Reason
pmid:32383353	eligible	yes	no (not_psi)	dental boundary re-adjudication under amendment A2: The prosthetic stems were sized from the medullary-cavity diameter, while the tooth-mechanism geometry was otherwise a common design.
pmid:36541118	eligible	yes	unclear	dental boundary re-adjudication under amendment A2: The text describes an additively manufactured subperiosteal implant structure but does not establish that its geometry was derived from an individual maxilla.
pmid:42351864	ai_independent_eval	yes	internal_only	AI validation re-adjudication (internal split of one source = internal_only): Development and test sets were patient-level splits of 11,973 augmented pairs from one PGIMER dataset; the additional patient case came from the same centre and
pmid:42320193	ai_independent_eval	yes	unclear	AI validation re-adjudication (internal split of one source = internal_only): Models used designs based on two femurs and were evaluated on a third unseen femur, but the third femur's institutional or dataset origin was not reported.
pmid:41436650	ai_independent_eval	unclear	internal_only	AI validation re-adjudication (internal split of one source = internal_only): The AI models were trained and evaluated using FEM-generated mechanical-response data; exact development and evaluation counts were not reported.
pmid:41094110	ai_independent_eval	yes	unclear	AI validation re-adjudication (internal split of one source = internal_only): A previously validated model was assessed in 34 retrospectively identified surgical patients, but its development source and whether predictions prospectively g
pmid:41094110	ai_clinical_eval	yes	unclear	AI validation re-adjudication (internal split of one source = internal_only): A previously validated model was assessed in 34 retrospectively identified surgical patients, but its development source and whether predictions prospectively g
pmid:40483854	ai_clinical_eval	unclear	no	AI validation re-adjudication (internal split of one source = internal_only): The augmented development set combined MUG500+ and SkullFix, and evaluation used SkullFix test cases from a source already represented in development; the test
pmid:40107082	ai_independent_eval	unclear	internal_only	AI validation re-adjudication (internal split of one source = internal_only): Surrogates were developed from 2,880 screw pull-out simulations and computationally evaluated through optimization for 80 trabecular-bone specimens.
pmid:40040189	ai_independent_eval	unclear	internal_only	AI validation re-adjudication (internal split of one source = internal_only): Evaluation used SkullBreak and SkullFix; no distinct external development-independent source or prospective clinical use was described.
pmid:40009971	ai_independent_eval	unclear	yes	AI validation re-adjudication (internal split of one source = internal_only): Quantitative testing used open benchmarks and additional qualitative evaluation used real clinical cases outside those benchmark data; no prospective workflow u
pmid:37185164	ai_independent_eval	unclear	internal_only	AI validation re-adjudication (internal split of one source = internal_only): All modelling and prediction-error assessment were based on one 673-knee database, with no external dataset or prospective patient use described; allocation cou
pmid:36257198	ai_independent_	yes	internal_only	AI validation re-adjudication (internal split of one source = internal_only): Development and testing used the AutoImplant 2021 challenge datasets; 3-D

ID	Field	Charted value	Corrected value	Reason
	eval			printing and mixed-reality assessment were bench evaluations, and counts were not repo
pmid:34340106	ai_independent_eval	yes	internal_only	AI validation re-adjudication (internal split of one source = internal_only): Training and evaluation pairs were generated by injecting synthetic defects into the same database of 167 healthy skulls; the number and provenance of real-defe
doi:10.21203/rs.3.rs-2252495/v1	ai_independent_eval	internal_only	unclear	AI validation re-adjudication (internal split of one source = internal_only): Twelve CT cases were prepared for U-Net training, but no held-out evaluation set was described; membrane fabrication and coating tests did not constitute prospe
doi:10.20944/preprints202307.0273.v1	ai_independent_eval	unclear	internal_only	AI validation re-adjudication (internal split of one source = internal_only): The configurations were explicitly tested on the same samples from one CT dataset; clinical trials were described only as future work.
doi:10.1007/s00366-020-01090-z	ai_clinical_eval	no	unclear	AI validation re-adjudication (internal split of one source = internal_only): The title identifies a symbolic-regression metamodel, but provides no development, evaluation-source, or prospective clinical-use details.
pmid:36257198	tier	bench	imaging_dataset	independent reviews: printing and mixed-reality viewing without a measured physical quantity does not reach the bench tier
title:0ea15fab4c8b9f62	(full row)	eligible unclear	eligible yes	re-assessment of an unclear record by a second sol pass (OpenAlex arm)
doi:10.1016/j.engfailanal.2021.105353	(full row)	eligible unclear	eligible yes	re-assessment of an unclear record by a second sol pass (OpenAlex arm)
doi:10.2139/ssrn.5936726	(full row)	eligible unclear	eligible yes	re-assessment of an unclear record by a second sol pass (OpenAlex arm)
doi:10.1007/s11665-024-09633-x	(full row)	eligible unclear	eligible yes	re-assessment of an unclear record by a second sol pass (OpenAlex arm)
doi:10.1007/978-3-030-88465-9_50	(full row)	eligible unclear	eligible yes	re-assessment of an unclear record by a second sol pass (OpenAlex arm)
doi:10.1016/j.ijsolstr.2025.113776	(full row)	eligible unclear	eligible unclear	re-assessment of an unclear record by a second sol pass (OpenAlex arm)
doi:10.1016/j.ijom.2025.04.703	(full row)	eligible unclear	eligible yes	re-assessment of an unclear record by a second sol pass (OpenAlex arm)
doi:10.1145/3774521.3774531	(full row)	eligible unclear	eligible yes	re-assessment of an unclear record by a second sol pass (OpenAlex arm)
title:62dd0a1a93fe84af	(full row)	eligible unclear	eligible no	re-assessment of an unclear record by a second sol pass (OpenAlex arm)
doi:10.1007/s42242-023-00259-x	(full row)	eligible unclear	eligible no	re-assessment of an unclear record by a second sol pass (OpenAlex arm)
doi:10.1109/isda.2015.7489206	(full row)	eligible unclear	eligible unclear	re-assessment of an unclear record by a second sol pass (OpenAlex arm)
doi:10.1055/s-0045-1803877	(full row)	eligible unclear	eligible yes	re-assessment of an unclear record by a second sol pass (OpenAlex arm)
doi:10.2139/ssrn.4484792	(full row)	eligible unclear	eligible yes	re-assessment of an unclear record by a second sol pass (OpenAlex arm)
doi:10.1007/s40430-018-1425-9	(full row)	eligible unclear	eligible yes	re-assessment of an unclear record by a second sol pass (OpenAlex arm)
doi:10.2139/ssrn.4499911	(full row)	eligible unclear	eligible yes	re-assessment of an unclear record by a second sol pass (OpenAlex arm)
doi:10.1007/978-981-15-0199-9_44	(full row)	eligible unclear	eligible yes	re-assessment of an unclear record by a second sol pass (OpenAlex arm)
doi:10.1016/j.irbm.2017.01.003	(full row)	eligible unclear	eligible yes	re-assessment of an unclear record by a second sol pass (OpenAlex arm)

ID	Field	Charted value	Corrected value	Reason
doi:10.1007/s40846-018-0370-5	(full row)	eligible unclear	eligible yes	re-assessment of an unclear record by a second sol pass (OpenAlex arm)
doi:10.1007/s40964-024-00866-9	(full row)	eligible unclear	eligible yes	re-assessment of an unclear record by a second sol pass (OpenAlex arm)
doi:10.1016/j.adoms.2021.100229	(full row)	eligible unclear	eligible unclear	re-assessment of an unclear record by a second sol pass (OpenAlex arm)
doi:10.1016/j.joms.2021.08.040	(full row)	eligible unclear	eligible no	re-assessment of an unclear record by a second sol pass (OpenAlex arm)
pmid:34215417	(full row)	eligible unclear	eligible no	re-assessment of an unclear record by a second sol pass (OpenAlex arm)
pmid:42055482	(full row)	eligible unclear	eligible yes	re-assessment of an unclear record by a second sol pass (OpenAlex arm)
pmid:40769801	(full row)	eligible unclear	eligible unclear	re-assessment of an unclear record by a second sol pass (OpenAlex arm)
doi:10.47749/t/unicamp.2025.1489775	(full row)	eligible unclear	eligible no	re-assessment of an unclear record by a second sol pass (OpenAlex arm)
doi:10.1007/s40964-026-01767-9	(full row)	eligible unclear	eligible yes	re-assessment of an unclear record by a second sol pass (OpenAlex arm)
doi:10.2139/ssrn.7324371	(full row)	eligible unclear	eligible unclear	re-assessment of an unclear record by a second sol pass (OpenAlex arm)
title:521c675cfacec9e5	ai_independent_eval	internal_only	unclear	AI validation re-adjudication (OpenAlex arm): Unseen volumes are mentioned, but neither dataset size nor their relationship to the training source is described; evaluation was computational and biomechanica
doi:10.1016/j.procs.2024.04.312	ai_independent_eval	internal_only	unclear	AI validation re-adjudication (OpenAlex arm): Performance on test images is reported without identifying their number, origin, or relationship to the training data; no prospective clinical use is described.
doi:10.1007/s40964-022-00342-2	ai_independent_eval	internal_only	unclear	AI validation re-adjudication (OpenAlex arm): The generative-design system had no reported development dataset; two designs for one patient case underwent prototype manufacturing and bench inspection only.
doi:10.48550/arxiv.2406.06372	ai_independent_eval	yes	unclear	AI validation re-adjudication (OpenAlex arm): Quantitative evaluation used SkullBreak and SkullFix, but their relationship to the development data and all sample counts are unstated; real clinical defects w
doi:10.5281/zenodo.18811309	eligible	yes	no (duplicate_report)	Zenodo version DOI of doi:10.5281/zenodo.18811308 (identical title, deposit and year); found at reference resolution
(all duplicate groups)	eligible	(various)	no (duplicate_report or duplicate_preprint)	report-level deduplication rerun over all 979 assessed records: 58 groups, 60 copies excluded, earlier duplicate corrections (47) replaced; keep rule journal > full text > PubMed copy; audit in searches/duplicate-groups.json
pmid:41003421	n_units	15	.	independent review reports four mechanically tested coupons in the full text against the charter's 15; the denominator is left unstated rather than adjudicated from either side
pmid:41003421	(full row)	(previous)	tier bench, n_patients 0	targeted re-charting after independent review (patient denominator, tier, quote)
pmid:32320044	eligible	yes	unclear	parameter-only customisation without anatomy-derived geometry established (A2 applied beyond the dental set; independent review)
pmid:32320044	(full row)	(previous)	tier ., n_patients .	targeted re-charting after independent review (patient denominator, tier, quote)
pmid:41248445	eligible	yes	unclear	clinical tier without a verified patient denominator (protocol minimum five); held unresolved

ID	Field	Charted value	Corrected value	Reason
				(independent review)
pmid:34984904	(full row)	(previous)	tier bench, n_patients 1	targeted re-charting after independent review (patient denominator, tier, quote)
pmid:34225889	(full row)	(previous)	tier bench, n_patients .	targeted re-charting after independent review (patient denominator, tier, quote)
pmid:30777714	eligible	yes	unclear	clinical tier without a verified patient denominator (protocol minimum five); held unresolved (independent review)
pmid:26174791	(full row)	(previous)	tier in_silico, n_patients .	targeted re-charting after independent review (patient denominator, tier, quote)
pmid:27107476	(full row)	(previous)	tier clinical_comparative, n_patients 30	targeted re-charting after independent review (patient denominator, tier, quote)
pmid:34984904	eligible	yes (bench)	unclear	animal evaluation with a single human application (round-3 review)
pmid:41003421	quote	(abstract sentence on 15 scaffolds)	(full-text coupon sentence)	the abstract states fifteen candidate scaffolds while the full text reports four mechanically tested lattice coupons; the tier quote now cites the full text and the discrepancy is recorded here

S1.7 Unclear fractions per charted field among included studies

Charted field	Unclear (%)
id	0.0
eligible	0.0
excl_code	100.0
year	0.0
country	67.6
region	0.0
implant_type	0.0
material	36.5
manufacturing	28.0
pillars	0.0
ai_task	94.4
ai_model	94.8
img_modality	30.8
img_method	65.7
comp_method	5.9
tier	0.0
n_patients	0.5
n_units	9.7
comparator	0.0
comparator_type	16.6
acc_result	55.4
mech_result	50.5
time_result	79.3
clin_result	63.1
followup	74.3
ai_independent_eval	96.9
ai_clinical_eval	94.5
code_or_data	64.3
credibility	0.0
quote	0.0

Text S2. Prospective protocol with dated amendments

Version 1.0, 2 September 2026. Committed to version control before any record was retrieved or screened. The commit history of this repository fixes the sequence; every later change is recorded in the amendments table (section 9) with its date and reason.

Design: systematic review reported per PRISMA 2020 (Page et al., BMJ 2021;372:n71; PMID 33782057) with the search reported per PRISMA-S; synthesis without meta-analysis reported per SWiM (Campbell et al., BMJ 2020;368:l6890; PMID 31948937). **Registration:** a PROSPERO record is prepared from this protocol (`docs/registration/`) for the author to submit; the manuscript states the true submission status. **Target journal (default, author's decision):** Journal of Clinical Medicine (MDPI); MDPI numbered citations; British spelling; no em dashes.

0. What had already happened when this protocol was committed

Eight PubMed hit-count queries were run on 2 September 2026 to size candidate concept blocks (counts only, retrieved with `retmax=0`; no record identifier, title or abstract was retrieved or read). Their strings and counts are archived in `searches/scoping-counts.md`. No record had been screened, and no finding had been drawn from any search, when this protocol was committed. The workspace's two earlier orthopaedic reviews (AI decision support; machine-learning complication prediction) were consulted for pipeline design only; neither covers implant design and neither contributed any eligibility judgement.

1. Rationale

Patient-specific implants, whose geometry is derived from the individual patient's imaging, are now routine in cranial, craniomaxillofacial, pelvic, spinal and limb-salvage reconstruction and are spreading into arthroplasty. Their design pipeline has three technical pillars: imaging, from acquisition through segmentation to a surface model; computational planning, including virtual surgical planning, statistical shape modelling, parametric and automated design, topology and lattice optimisation and finite element verification; and, most recently, artificial intelligence, applied to segmentation, defect and shape completion, design generation, parameter prediction and outcome prediction. Each pillar carries its own validation literature, much of it in engineering venues, and each is invoked in clinical papers as though its accuracy were established. No review, to our knowledge, has set out what evidence actually exists for each pillar in the specific setting of implant design, at which tier of evidence (in silico, bench, cadaver or phantom, clinical series, comparative clinical), with what comparators and with what risk of bias. This review maps that evidence.

2. Objectives and review questions

Primary objective. To identify and appraise primary studies published from 2015 onward that apply artificial intelligence, an evaluated imaging pipeline, or computational planning to the design of patient-specific skeletal implants, and to map the evidence by pillar, anatomical region, evidence tier and outcome.

Review questions. 1. What methods within each pillar have been applied to patient-specific implant design, for which anatomical regions and implant types? 2. At what evidence tier has each method been evaluated, against what comparator, with what sample size and follow-up? 3. What outcomes are reported (geometric accuracy and fit, mechanical performance, design and production time, clinical outcomes) and what do they show? 4. For artificial intelligence methods specifically, is there any evaluation on data independent of development, any prospective or clinical evaluation, and any public model or code? 5.

What is the risk of bias of the clinical studies and the technical credibility of the computational studies? 6. Where are the gaps: which pillar, region or outcome has no evidence above the in-silico tier?

3. Eligibility criteria

Population and setting. Human patients of any age, or human anatomical data (clinical imaging datasets, cadaveric specimens, anatomy-derived phantoms), for a skeletal implant: a device implanted in or onto bone in orthopaedic, spinal, cranial, craniomaxillofacial or jaw reconstruction, including joint replacement components, tumour endoprostheses, cranioplasty plates, orbital and midface plates, mandibular reconstruction implants, spinal cages and vertebral body replacements, and patient-specific subperiosteal or reconstruction jaw implants.

Concept, both required. (a) The implant is patient-specific: its geometry, or the geometry of a load-bearing part of it, is derived from the individual patient's imaging or anatomy. Standard off-the-shelf implants, including 3D-printed but non-individualised implants, do not qualify. (b) At least one of the three pillars is applied to the implant's design or planning and is evaluated: - **Artificial intelligence:** machine learning or deep learning at any step of the pipeline (segmentation for implant design, defect or shape completion, design generation, design parameter prediction, prediction of fit or outcome for a patient-specific implant). - **Imaging:** an evaluation of the image-to-implant pipeline itself (segmentation accuracy for the design, the effect of imaging protocol or modality on the implant, registration or mirroring accuracy, statistical or template shape modelling for the design). - **Computational planning:** finite element or other biomechanical simulation of the specific implant, topology, lattice or shape optimisation, parametric or automated design, or virtual surgical planning where the implant itself is planned (guide-only planning does not qualify).

Evaluation requirement. The study reports at least one measured quantity about the method or the implant: geometric deviation or fit, mechanical response, design or production time, or a clinical outcome. Workflow descriptions without any measured quantity are excluded.

Study types. Primary research at any tier: in-silico studies, bench or mechanical testing, cadaveric or phantom studies, retrospective imaging-dataset studies, clinical case series of at least five patients, comparative non-randomised studies and randomised trials. Excluded: case reports and series of fewer than five patients (counted at screening, reported in the flow, not charted); reviews, editorials, letters, protocols, conference abstracts without full papers; non-human studies; studies of patient-specific instrumentation or guides without a patient-specific implant; standard endosseous dental implants and prosthodontic restorations; external prostheses and orthoses; non-skeletal implants (cardiovascular, breast, cochlear, soft tissue); tissue-engineering studies without an implant designed for a specific patient.

Dates and languages. Publication date 1 January 2015 to the search date, chosen because "current evidence" is the review's remit and the deep learning literature in medical imaging begins in this window. No language restriction at screening; non-English records are charted from the abstract and any machine-readable full text, and flagged.

4. Information sources and search

Sources searched by script with archived raw responses: MEDLINE via PubMed (E-utilities), Europe PMC (title and abstract fields), OpenAlex (title and abstract search, fully retrievable), Crossref (bounded relevance sweep, disclosed as supplementary). PROSPERO is searched for competing reviews. Reference lists of systematic and scoping reviews identified during screening are chased backward for primary studies. Embase, Scopus, Web of Science, IEEE Xplore and CINAHL are unavailable to the project and this is declared as a limitation in advance. The search is built as the intersection of four concept blocks, individualisation (patient-specific, custom, personalised, 3D-printed and synonyms), device (implant,

prosthesis and specific device terms), skeletal region, and method (the union of the three pillars' vocabularies), with a publication-date limit and no incidence, outcome or study-type filter, to protect recall. Full strings are in `scripts/search.py` and are printed verbatim to `searches/search-log.md` by the run.

5. Selection

1. **Rule layer.** Explicit, published rules exclude records that (i) lack any individualisation term, (ii) lack any device term, (iii) carry a non-human indexing signal, (iv) carry a review, editorial, letter, comment or protocol publication type (reviews are retained in a separate list for citation chasing), or (v) carry the Case Reports publication type. Each exclusion is published with its rule. A seeded random sample of 25 rule-excluded records is re-read against the criteria to bound the false-exclusion rate.
2. **Dual independent AI title-abstract screening.** Two screeners from different vendors (a GPT-based agent via the Codex CLI and a Claude model), each blind to the other, classify every rule-passing record as include, exclude or uncertain against the criteria, from a single written instruction file that is a deliverable. A record is retained unless both exclude it (recall-union). Outputs are reconciled against the input identifier set; any record dropped by either screener is re-classified. Raw agreement, the full cross-tabulation and chance-corrected agreement are reported.
3. **Full-record assessment.** Retained records are assessed against the criteria by an AI assessor from full text where openly available (PMC, Europe PMC, Unpaywall) and otherwise from the abstract, with per-record reasons published; the source of the text used is recorded per record.
4. **Disclosure.** All screening in the manuscript's first version is AI-performed and provisional; the independent human screen required by this protocol is an author action before submission, and the manuscript says so.

6. Data items and charting

One row per report, charted by AI agents from a written instruction file that is a deliverable, with the sentence supporting the evidence-tier assignment quoted verbatim. Items: identifiers; year; country; anatomical region; implant type and material; manufacturing route; pillar(s) applied (artificial intelligence, imaging, computational planning; more than one allowed); method detail per pillar (AI task and model family; imaging modality and segmentation approach; computational method); evidence tier (in silico; bench or mechanical; cadaver or phantom; retrospective imaging dataset; clinical non-comparative; clinical comparative non-randomised; randomised); sample size (patients, cases, specimens or models); comparator; outcomes reported by domain (geometric accuracy and fit; mechanical; design or production time; clinical: complications, revision, function, operative time) with the headline value and its unit; follow-up; for AI studies, whether evaluation used data independent of development, whether any prospective or clinical evaluation exists, and whether code or model is public; data and code availability; funding and conflicts where stated. Where a value is not settled by the text, the field is `unclear`, never `no`.

7. Appraisal

- Randomised trials: RoB 2. Comparative non-randomised clinical studies: ROBINS-I. Non-comparative clinical series: the JBI checklist for case series.
- Technical studies (in silico, bench, cadaver or phantom, imaging dataset, AI development): a six-item credibility set derived from ASME V&V 40 and TRIPOD+AI/CLAIM, reported as item counts rather than a score: (1) validation data or specimens independent of those used to develop or tune the method; (2) quantitative comparison against a physical experiment or a ground truth; (3) uncertainty or sensitivity analysis reported; (4) inputs specified reproducibly (imaging protocol, material properties, loads and boundary conditions, or training data description); (5) code, model or design files available; (6) more than one anatomy or specimen evaluated.
- Certainty of evidence for clinical outcome domains is summarised per GRADE principles narratively (no formal Summary of Findings table is expected because no meta-analysis is expected).

8. Synthesis

Structured narrative synthesis per SWiM, grouped by pillar and then by anatomical region, with an evidence map (tier by pillar by region) and tables of included studies. A meta-analysis is run only if at least three comparative clinical studies report the same outcome against the same class of comparator with extractable estimates; then a random-effects model with prediction interval. Otherwise no pooled

estimate is produced, and the manuscript says so rather than pooling heterogeneous designs. Counts in the manuscript are computed by script from the charting file and checked by a verification script; no count is typed by hand.

9. Amendments

Date	Change	Reason
2026-09-02	A1. OpenAlex could not be searched: the API now meters a free daily budget per client and every request returned HTTP 429 (budget exhausted, reset at midnight UTC) during the search run. The run proceeded on MEDLINE via PubMed, Europe PMC and the Crossref bounded sweep; the OpenAlex strings remain in `scripts/search.py` and its unavailability is logged per query in `searches/search-log.md`. If OpenAlex becomes available before the manuscript is finalised, the queries will be run as a dated supplementary search and any new records screened under the same rules and reported as a separate row in the flow	Third-party service limit discovered during retrieval, before any record was screened
2026-09-02	A2. Clarification of the dental boundary raised by the second charter: root-analogue or otherwise anatomy-derived endosseous jaw implants (geometry taken from the individual socket or jaw) are eligible as patient-specific skeletal implants; implants customised only by parameter choice (length, diameter, lattice parameter, patient weight) without anatomy-derived geometry are not patient-specific (`not_psi`); standard root-form dental implants and prosthodontic restorations remain excluded	Boundary case not settled by the protocol wording; decided before any adjudication of the affected records and applied uniformly at adjudication
2026-09-02	A3. Preprints (Research Square, medRxiv, bioRxiv, arXiv, SSRN) are eligible as primary research when they carry a full report; they are identified by their venue in Table S1 and their number is stated in the Results, and the absence of peer review is named in the limitations. Preprints later published as journal articles present in the corpus are treated as duplicates of the journal version	The protocol excluded conference abstracts without full papers but was silent on preprints; the boundary was raised by the second charter and fixed before adjudication
2026-09-03	A4. Rule (iii) as implemented excludes a record with a non-human indexing signal only when no human signal (patient, human, clinical, cadaver, volunteer, surgery) is also present; 138 records carried both signals and were retained for triage rather than excluded. The committed wording did not state the exception	Implementation detail disclosed after independent review; the exception favours recall and is reported with its count
2026-09-03	A5. Retrieval-count discrepancies explained: PubMed S1 reported 2457 hits and 2456 records were fetched (one identifier, PMID 36469653, was not returned by efetch and is absent from the corpus); Europe PMC reported 1686 hits and 1687 records were returned because one record (PMID 34984904) appeared on two cursor pages and was removed at deduplication	Reconciliation of the archived raw responses after independent review
2026-09-03	A6. Residual duplicate reports: assessed records that were the same article as another assessed record under a second identifier (a DOI-keyed copy from Europe PMC or Crossref beside the PubMed copy, or two identical titles), in addition to the 15 preprint copies of amendment A3. They are excluded at assessment as duplicate reports (the journal version over a preprint, repository or version copy, then the copy with full text, then the PubMed copy, is retained, by a report-level grouping over all assessed records on normalised title, DOI stem and preprint-journal pairs, rerun after independent review found the first pass incomplete and one circular pair; final counts are those in the flow; where the copies disagreed on eligibility or tier the retained copy's charting stands and the disagreement is listed in the corrections file), counted in the flow, and the deduplication rule of scripts/search.py (identifier-based) is recorded as the cause	Found by both independent reviews; corrected before the revised draft

Date	Change	Reason
2026-09-03	A7. Funding and conflict-of-interest items (section 6) were omitted from the charting form and are charted in a separate pass from full text only (funding source, conflict declaration, industry authorship); abstract-only records carry unclear	Omission found by independent review; corrected as a supplementary charting pass
2026-09-03	A8. Meta-analysis: the prespecified condition (at least three comparative clinical studies with the same outcome against the same comparator class and extractable estimates) is tested on a dedicated extraction of arm-level estimates from the comparative studies and trials with full text (operative time, blood loss, geometric accuracy, complications, revision, infection, function); where the condition holds a random-effects pooled estimate with a prediction interval is reported for the delivered implant against its comparator, labelled as evidence about the implant rather than the design method; where it fails, the disposition is stated. The first draft's decision not to pool despite the condition being met was a post hoc deviation and is withdrawn	Independent review of the first draft
2026-09-03	A9. OpenAlex supplementary search run on 3 September 2026 once the API budget reset (amendment A1): 412 new unique records (414 at first count, two of which duplicated PubMed records already in the corpus and were removed before assessment), screened by the same rule layer and dual triage, charted and appraised in the same way, and reported as a separate arm of the flow	Fulfilment of A1

Text S3. Full search strategies (PRISMA-S)

Run date: 2026-09-02 Retrieval performed by `scripts/search.py` (protocol section 4); raw responses cached in `searches/raw/`. Crossref is a bounded relevance sweep (first pages only) and is logged as supplementary, not as a full-retrieval source.

Source / strategy	Hits reported	Records retrieved
MEDLINE via PubMed / S1_primary	2457	2456
MEDLINE via PubMed / S2_psi_phrase	751	751
Europe PMC / E1_primary	1686	1687
OpenAlex / O1 (UNAVAILABLE: HTTP 429)	n/a	0
OpenAlex / O2 (UNAVAILABLE: HTTP 429)	n/a	0
OpenAlex / O3 (UNAVAILABLE: HTTP 429)	n/a	0
OpenAlex / O4 (UNAVAILABLE: HTTP 429)	n/a	0
OpenAlex / O5 (UNAVAILABLE: HTTP 429)	n/a	0
OpenAlex / O6 (UNAVAILABLE: HTTP 429)	n/a	0
OpenAlex / O7 (UNAVAILABLE: HTTP 429)	n/a	0
OpenAlex / O8 (UNAVAILABLE: HTTP 429)	n/a	0
OpenAlex / O9 (UNAVAILABLE: HTTP 429)	n/a	0
OpenAlex / O10 (UNAVAILABLE: HTTP 429)	n/a	0
OpenAlex / O11 (UNAVAILABLE: HTTP 429)	n/a	0
OpenAlex / O12 (UNAVAILABLE: HTTP 429)	n/a	0
OpenAlex / O13 (UNAVAILABLE: HTTP 429)	n/a	0
OpenAlex / O14 (UNAVAILABLE: HTTP 429)	n/a	0
Crossref (bounded sweep) / C1	5434738	1000
Crossref (bounded sweep) / C2	3229240	1000

Records retrieved across all strategies: 6894 **Unique records after deduplication:** 4647

Full search strings

MEDLINE via PubMed / S1_primary

```
("patient-specific"[tiab] OR "patient specific"[tiab] OR "patient-matched"[ti
ab] OR "custom-made"[tiab] OR "custom made"[tiab] OR customized[tiab] OR cust
omised[tiab] OR personalized[tiab] OR personalised[tiab] OR individualized[ti
ab] OR individualised[tiab] OR "3D-printed"[tiab] OR "3D printed"[tiab] OR "t
hree-dimensionally printed"[tiab] OR "additively manufactured"[tiab] OR "addi
tive manufacturing"[tiab]) AND (implant*[tiab] OR prosthes*[tiab] OR endopros
thes*[tiab] OR cranioplasty[tiab] OR "reconstruction plate*"[tiab] OR "interb
ody cage*"[tiab] OR "vertebral body replacement"[tiab] OR "cranial plate*"[ti
ab] OR "orbital plate*"[tiab] OR megaprosthes*[tiab]) AND (bone*[tiab] OR ort
hop*[tiab] OR skull[tiab] OR cranial[tiab] OR cranio*[tiab] OR maxillofacial
[tiab] OR mandib*[tiab] OR maxill*[tiab] OR spine[tiab] OR spinal[tiab] OR ve
rtebra*[tiab] OR pelvi*[tiab] OR acetabul*[tiab] OR hip[tiab] OR knee[tiab] O
R femur[tiab] OR femoral[tiab] OR tibia*[tiab] OR humer*[tiab] OR shoulder[ti
ab] OR glenoid[tiab] OR scapul*[tiab] OR radius[tiab] OR ulna*[tiab] OR wrist
[tiab] OR ankle[tiab] OR talus[tiab] OR calcane*[tiab] OR sternum[tiab] OR ri
b[tiab] OR ribs[tiab] OR "chest wall"[tiab] OR sacr*[tiab] OR orbit*[tiab] OR
 zygoma*[tiab] OR temporomandibular[tiab] OR arthroplast*[tiab] OR osteotomy
[tiab] OR "limb salvage"[tiab] OR tumour[tiab] OR tumor[tiab] OR sarcoma[tiab]
```

OR "Bone and Bones"[Mesh] OR "Orthopedic Procedures"[Mesh] OR "Skull"[Mesh] OR "Spine"[Mesh] OR "Facial Bones"[Mesh] OR "Jaw"[Mesh]) AND (("artificial intelligence"[tiab] OR "machine learning"[tiab] OR "deep learning"[tiab] OR "neural network*"[tiab] OR convolutional[tiab] OR "generative design"[tiab] OR "generative model*"[tiab] OR "Artificial Intelligence"[Mesh] OR "Machine Learning"[Mesh] OR "Deep Learning"[Mesh]) OR ("finite element"[tiab] OR "topology optimi*"[tiab] OR "topological optimi*"[tiab] OR "shape optimi*"[tiab] OR "statistical shape"[tiab] OR "biomechanical simulation"[tiab] OR "computational model*"[tiab] OR "computational design"[tiab] OR "automated design"[tiab] OR "automatic design"[tiab] OR "design automation"[tiab] OR "virtual surgical planning"[tiab] OR "virtual planning"[tiab] OR "computer-assisted planning"[tiab] OR "computer assisted planning"[tiab] OR "Finite Element Analysis"[Mesh] OR "Computer Simulation"[Mesh] OR lattice[tiab] OR "parametric design"[tiab] OR "parametric model*"[tiab]) OR (segmentation[tiab] OR "image-based"[tiab] OR "image based"[tiab] OR "imaging protocol*"[tiab] OR mirror*[tiab] OR registration[tiab] OR "point cloud"[tiab] OR "surface reconstruction"[tiab] OR "shape completion"[tiab] OR "defect reconstruction"[tiab])) AND 2015:2026[dp]

MEDLINE via PubMed / S2_psi_phrase

("patient-specific implant*"[tiab] OR "patient specific implant*"[tiab] OR "patient-specific prosthes*"[tiab] OR "patient specific prosthes*"[tiab] OR "custom implant*"[tiab] OR "customized implant*"[tiab] OR "customised implant*"[tiab] OR "custom-made implant*"[tiab] OR "custom made implant*"[tiab] OR "personalized implant*"[tiab] OR "personalised implant*"[tiab] OR "individualized implant*"[tiab] OR "individualised implant*"[tiab] OR "patient-matched implant*"[tiab] OR "3D-printed implant*"[tiab] OR "3D printed implant*"[tiab] OR "additively manufactured implant*"[tiab] OR "custom prosthes*"[tiab] OR "customized prosthes*"[tiab] OR "customised prosthes*"[tiab] OR "patient-specific cranioplasty"[tiab] OR "patient-specific cranial implant*"[tiab] OR "customized cranioplasty"[tiab] OR "patient-specific reconstruction"[tiab]) AND (("artificial intelligence"[tiab] OR "machine learning"[tiab] OR "deep learning"[tiab] OR "neural network*"[tiab] OR convolutional[tiab] OR "generative design"[tiab] OR "generative model*"[tiab] OR "Artificial Intelligence"[Mesh] OR "Machine Learning"[Mesh] OR "Deep Learning"[Mesh]) OR ("finite element"[tiab] OR "topology optimi*"[tiab] OR "topological optimi*"[tiab] OR "shape optimi*"[tiab] OR "statistical shape"[tiab] OR "biomechanical simulation"[tiab] OR "computational model*"[tiab] OR "computational design"[tiab] OR "automated design"[tiab] OR "automatic design"[tiab] OR "design automation"[tiab] OR "virtual surgical planning"[tiab] OR "virtual planning"[tiab] OR "computer-assisted planning"[tiab] OR "computer assisted planning"[tiab] OR "Finite Element Analysis"[Mesh] OR "Computer Simulation"[Mesh] OR lattice[tiab] OR "parametric design"[tiab] OR "parametric model*"[tiab]) OR (segmentation[tiab] OR "image-based"[tiab] OR "image based"[tiab] OR "imaging protocol*"[tiab] OR mirror*[tiab] OR registration[tiab] OR "point cloud"[tiab] OR "surface reconstruction"[tiab] OR "shape completion"[tiab] OR "defect reconstruction"[tiab])) AND 2015:2026[dp]

Europe PMC / E1_primary

```
(TITLE_ABS:"patient-specific" OR TITLE_ABS:"patient specific" OR TITLE_ABS:"p
atient-matched" OR TITLE_ABS:"custom-made" OR TITLE_ABS:"custom made" OR TITL
E_ABS:customized OR TITLE_ABS:customised OR TITLE_ABS:personalized OR TITLE_A
BS:personalised OR TITLE_ABS:individualized OR TITLE_ABS:individualised OR TI
TLE_ABS:"3D-printed" OR TITLE_ABS:"3D printed" OR TITLE_ABS:"additively manuf
actured" OR TITLE_ABS:"additive manufacturing") AND (TITLE_ABS:implant OR TIT
LE_ABS:implants OR TITLE_ABS:prosthesis OR TITLE_ABS:prostheses OR TITLE_ABS:
endoprosthesis OR TITLE_ABS:cranioplasty OR TITLE_ABS:"reconstruction plate"
 OR TITLE_ABS:"interbody cage" OR TITLE_ABS:"vertebral body replacement" OR TI
TLE_ABS:megaprosthesis) AND (TITLE_ABS:bone OR TITLE_ABS:bones OR TITLE_ABS:o
rthopaedic OR TITLE_ABS:orthopedic OR TITLE_ABS:skull OR TITLE_ABS:cranial OR
 TITLE_ABS:craniofacial OR TITLE_ABS:maxillofacial OR TITLE_ABS:mandible OR T
ITLE_ABS:mandibular OR TITLE_ABS:maxilla OR TITLE_ABS:spine OR TITLE_ABS:spin
al OR TITLE_ABS:vertebral OR TITLE_ABS:pelvic OR TITLE_ABS:pelvis OR TITLE_AB
S:acetabular OR TITLE_ABS:hip OR TITLE_ABS:knee OR TITLE_ABS:femur OR TITLE_A
BS:femoral OR TITLE_ABS:tibia OR TITLE_ABS:tibial OR TITLE_ABS:humerus OR TIT
LE_ABS:shoulder OR TITLE_ABS:glenoid OR TITLE_ABS:scapula OR TITLE_ABS:orbita
l OR TITLE_ABS:zygoma OR TITLE_ABS:temporomandibular OR TITLE_ABS:arthroplast
y OR TITLE_ABS:sarcoma OR TITLE_ABS:tumour OR TITLE_ABS:tumor OR TITLE_ABS:"c
hest wall" OR TITLE_ABS:sternum OR TITLE_ABS:sacrum OR TITLE_ABS:ankle OR TIT
LE_ABS:wrist) AND (TITLE_ABS:"artificial intelligence" OR TITLE_ABS:"machine 
learning" OR TITLE_ABS:"deep learning" OR TITLE_ABS:"neural network" OR TITLE
_ABS:"neural networks" OR TITLE_ABS:convolutional OR TITLE_ABS:"generative de
sign" OR TITLE_ABS:"finite element" OR TITLE_ABS:"topology optimization" OR T
ITLE_ABS:"topology optimisation" OR TITLE_ABS:"shape optimization" OR TITLE_A
BS:"shape optimisation" OR TITLE_ABS:"statistical shape" OR TITLE_ABS:"biomec
hanical simulation" OR TITLE_ABS:"computational model" OR TITLE_ABS:"computat
ional design" OR TITLE_ABS:"automated design" OR TITLE_ABS:"design automation
" OR TITLE_ABS:"virtual surgical planning" OR TITLE_ABS:"virtual planning" OR
 TITLE_ABS:lattice OR TITLE_ABS:"parametric design" OR TITLE_ABS:segmentation
 OR TITLE_ABS:"image-based" OR TITLE_ABS:"point cloud" OR TITLE_ABS:"shape co
mpletion" OR TITLE_ABS:mirroring OR TITLE_ABS:registration) AND (PUB_YEAR:[20
15 TO 2026])
```

OpenAlex / O1 (UNAVAILABLE: HTTP 429)

```
"patient-specific implant" "finite element"
```

OpenAlex / O2 (UNAVAILABLE: HTTP 429)

```
"patient-specific implant" "deep learning"
```

OpenAlex / O3 (UNAVAILABLE: HTTP 429)

```
"patient-specific implant" "machine learning"
```

OpenAlex / O4 (UNAVAILABLE: HTTP 429)

```
"patient-specific implant" segmentation
```

OpenAlex / O5 (UNAVAILABLE: HTTP 429)

`"patient-specific implant" "topology optimization"`

OpenAlex / O6 (UNAVAILABLE: HTTP 429)

`"patient-specific implant" "statistical shape"`

OpenAlex / O7 (UNAVAILABLE: HTTP 429)

`"custom implant" "finite element" bone`

OpenAlex / O8 (UNAVAILABLE: HTTP 429)

`"patient-specific" cranioplasty "deep learning"`

OpenAlex / O9 (UNAVAILABLE: HTTP 429)

`"patient-specific" implant "generative design"`

OpenAlex / O10 (UNAVAILABLE: HTTP 429)

`"patient-specific" implant "virtual surgical planning"`

OpenAlex / O11 (UNAVAILABLE: HTTP 429)

`"3D-printed implant" "finite element" "patient-specific"`

OpenAlex / O12 (UNAVAILABLE: HTTP 429)

`"cranial implant" "deep learning"`

OpenAlex / O13 (UNAVAILABLE: HTTP 429)

`"patient-specific" "mandibular reconstruction" implant "finite element"`

OpenAlex / O14 (UNAVAILABLE: HTTP 429)

`"patient-specific" "pelvic" implant "finite element"`

Crossref (bounded sweep) / C1

`patient-specific implant design artificial intelligence deep learning`

Crossref (bounded sweep) / C2

`patient-specific implant finite element topology optimization design`

Supplementary OpenAlex search (amendment A9, run 3 September 2026 after the API budget reset)

Query	Hits	Retrieved	New (not in the 2 September corpus)
O1: `"patient-specific implant" "finite element"`	165	165	84
O2: `"patient-specific implant" "deep learning"`	30	30	20
O3: `"patient-specific implant"`	35	35	17

Query	Hits	Retrieved	New (not in the 2 September corpus)
"machine learning"			
O4: "patient-specific implant" segmentation	80	80	36
O5: "patient-specific implant" "topology optimization"	18	18	3
O6: "patient-specific implant" "statistical shape"	18	18	4
O7: "custom implant" "finite element" bone	71	71	42
O8: "patient-specific" cranioplasty "deep learning"	10	10	3
O9: "patient-specific" implant "generative design"	19	19	10
O10: "patient-specific" implant "virtual surgical planning"	294	294	132
O11: "3D-printed implant" "finite element" "patient-specific"	15	15	3
O12: "cranial implant" "deep learning"	60	60	44
O13: "patient-specific" "mandibular reconstruction" implant "finite element"	39	39	12
O14: "patient-specific" "pelvic" implant "finite element"	24	24	4

New unique records: 414

Text S4. Instructions given to the AI screeners, charters and assessors

S4.1 Screening instructions

Both AI screeners work from this file and nothing else, so that the only difference between them is the model reading it. It is a deliverable.

You are screening records for a systematic review of **patient-specific skeletal implant design: primary studies that apply artificial intelligence, an evaluated imaging pipeline, or computational planning to the design of an implant whose geometry is derived from the individual patient's anatomy**. You decide eligibility only. Do not judge study quality.

Output format, exactly

One tab-separated line per record, in input order. No preamble, no commentary, no markdown, no blank lines.

`ID<TAB>decision<TAB>reason`

- `decision`: `include`, `exclude`, or `uncertain`
- `reason`: for `exclude`, exactly one code from the table. For `include`, a short phrase naming the pillar(s) and the implant (e.g. `AI segmentation; cranioplasty`). For `uncertain`, what is missing.

Every record must appear exactly once, with the ID copied verbatim. If unsure, use `uncertain`; a wrong `exclude` is invisible and permanent, an `uncertain` costs nothing.

Exclusion codes

Code	Meaning
`not_psi`	No patient-specific implant: implant is off-the-shelf or generic (including generic 3D-printed designs), or only guides, templates, instrumentation, models or simulators are patient-specific
`not_skeletal`	Implant is not in or on bone (cardiovascular, breast, cochlear, soft tissue), or is a standard endosseous dental implant or prosthodontic restoration, or an external prosthesis or orthosis
`no_method`	None of the three pillars is applied and evaluated: no AI or machine learning; no evaluation of the image-to-implant pipeline (segmentation, registration, mirroring, shape modelling, imaging protocol); no finite element or biomechanical simulation, topology, lattice or shape optimisation, parametric or automated design, or virtual planning of the implant itself
`no_evaluation`	Workflow or technique description with no measured quantity (no accuracy, fit, mechanical, time or clinical result)
`case_report`	Human clinical report of fewer than five patients (a single case, a two-case note)
`source_type`	Review, editorial, letter, protocol, conference abstract without full paper, book chapter
`non_human`	Animal or non-human-anatomy study
`pre_2015`	Published before 2015
`other`	Anything else; state it

Include if ALL hold

1. **Patient-specific implant.** The implant, or a load-bearing part of it, is designed from the individual patient's imaging or anatomy. Cranioplasty plates, orbital or midface plates, mandibular reconstruction implants and plates designed on the patient's model, patient-specific subperiosteal or reconstruction jaw implants, custom acetabular or pelvic implants, tumour endoprostheses, custom joint replacement components, custom spinal cages and vertebral body replacements, custom osteotomy plates that are implanted. **Include**

in-silico, bench, cadaver, phantom and imaging-dataset studies as long as the design concept is patient-specific, even without any patient treated.

2. **At least one pillar applied to the design or planning and evaluated:**
    - AI: machine or deep learning for segmentation intended for implant design, defect or skull/bone shape completion, implant design generation, design parameter prediction, or fit/outcome prediction for a patient-specific implant.
    - Imaging: an evaluation of segmentation accuracy, imaging modality or protocol effects, registration or mirroring accuracy, or statistical shape modelling used for the implant design.
    - Computational planning: finite element or other biomechanical simulation of the specific implant, topology, lattice or shape optimisation, parametric or automated design, or virtual surgical planning where the implant itself is planned.
3. **Some measured quantity is reported** (geometric deviation, fit, stress, stiffness, fatigue, time, clinical outcome).
4. **Primary research, 2015 or later.** Clinical studies need at least five patients; technical studies have no minimum.

Hard cases

- Patient-specific *instrumentation* (cutting guides) for a standard implant: `not_psi`, unless a patient-specific implant is also designed.
- Patient-specific *anatomical models* for planning only: `not_psi`.
- Generic lattice or porous implant optimised with FEA, not for a specific patient: `not_psi`.
- Deep learning segmentation of bone with no stated implant-design purpose: `uncertain` if implant design is plausibly the purpose, otherwise `no_method`. Skull defect reconstruction or shape completion (AutoImplant-style) counts as implant design: include.
- Dental: standard root-form implants and crowns are `not_skeletal`; patient-specific subperiosteal implants, custom jaw reconstruction implants and custom TMJ prostheses are skeletal: include if the rest holds.
- A clinical series that used a patient-specific implant designed with FEA or virtual planning and reports outcomes: include (the pillar was applied and the outcome is the evaluation).
- A clinical series of patient-specific implants with no pillar named beyond "designed by CAD": `no_method`; if the abstract hints at simulation or planning but is unclear, `uncertain`.

S4.2 Charting instructions

You are charting records for a systematic review of **patient-specific skeletal implant design: primary studies (2015 onward) applying artificial intelligence, an evaluated imaging pipeline, or computational planning to the design of an implant whose geometry derives from the individual patient's anatomy**. First confirm eligibility from the fuller text, then extract. Read what is given (abstract, and full text where marked); never infer from recall of the paper.

Output: one line per record, 30 tab-separated fields, in this exact order

```
id	eligible	excl_code	year	country	region	implant_type	material	manufacturing	pillars	ai_task	ai_model	img_modality	img_method	comp_method	tier	n_patients	n_units	comparator	comparator_type	acc_result	mech_result	time_result	clin_result	followup	ai_independent_eval	ai_clinical_eval	code_or_data	credibility	quote
```

No header, no commentary, no markdown, no blank lines. Every record exactly once, ID copied verbatim. Use a literal `.` for any field with nothing; never leave a field empty; strip tabs and newlines from free text.

Field	Allowed values
`eligible`	`yes` `no` `unclear`
`excl_code`	if `no`: `not_psi` `not_skeletal` `no_method` `no_evaluation` `case_report` `source_type` `non_human` `pre_2015` `other`; else `.`
`year`	four digits
`country`	country of the first or corresponding author's institution, or `.`

Field	Allowed values
region	cranial cmf (orbit, midface, zygoma, mandible, maxilla, TMJ, jaw) spine pelvis_acetabulum hip knee shoulder upper_limb_other lower_limb_other chest_wall multi other
implant_type	short free text, e.g. cranioplasty plate mandibular reconstruction plate acetabular tri-flange tumour endoprosthesis interbody cage subperiosteal jaw implant
material	titanium peek pmma ceramic cocr polymer_other multiple unclear
manufacturing	am_metal (SLM, EBM, DMLS) am_polymer machined cast multiple not_manufactured (in silico only) unclear
pillars	one or more of ai imaging comp, joined by + in that order, e.g. ai+comp
ai_task	if ai: segmentation shape_completion design_generation parameter_prediction outcome_prediction registration other; joined by +; else .
ai_model	if ai: short family, e.g. 3D U-Net GAN diffusion random forest point-cloud network unclear; else .
img_modality	ct cbct mri ct+mri other unclear
img_method	if imaging pillar: segmentation_accuracy protocol_effect registration mirroring ssm template other; joined by +; else .
comp_method	if comp: fea topology_opt lattice_opt shape_opt ssm_design parametric_design automated_design vsp musculoskeletal_model; joined by +; else .
tier	the HIGHEST evidence tier in the study: in_silico bench cadaver_phantom imaging_dataset (retrospective images, no implant made or tested) clinical_series clinical_comparative rct
n_patients	integer patients treated clinically, or 0 if none, or . if unclear
n_units	integer of cases, images, specimens, models or designs evaluated (the analysed unit), or .
comparator	yes no
comparator_type	if yes: conventional_implant manual_design alternative_method ground_truth no_treatment other; else .
acc_result	headline geometric accuracy or fit result with unit, e.g. mean surface deviation 0.6 mm Dice 0.94 .
mech_result	headline mechanical result, e.g. peak von Mises 210 MPa under 2.3 kN .
time_result	headline design or production time result, e.g. design time 9 min vs 3 h manual .
clin_result	headline clinical result, e.g. complications 3/24; no revision at 18 mo .
followup	e.g. 18 mo mean .
ai_independent_eval	if ai: yes (evaluated on data not used for development or tuning, e.g. external centre, held-out test set from different source) internal_only unclear; else .
ai_clinical_eval	if ai: yes (used prospectively or in patient care) no unclear; else .
code_or_data	code data both none unclear
credibility	six characters, one per item, y n or u: (1) validation data or specimens independent of development or tuning; (2) quantitative comparison against physical experiment or ground truth; (3) uncertainty or sensitivity analysis; (4) inputs reproducibly specified (imaging protocol; material properties, loads and boundary conditions; or

Field	Allowed values
	training data description); (5) code, model or design files available; (6) more than one anatomy or specimen evaluated. For clinical-tier studies without a technical component write `......`
`quote`	one verbatim sentence from the source that establishes the tier assignment (e.g. the sentence saying patients were treated, or that testing was in silico only)

Rules

- `unclear`, never `no`, when the text does not settle it. Records marked FULL TEXT NOT AVAILABLE will have many `unclear` fields; that is correct.
- Eligibility again: the implant must be patient-specific (not guides, models or generic designs); at least one pillar applied to the design and evaluated; some measured quantity; primary research; 2015 or later; clinical studies need at least five patients. A study eligible on the abstract but ineligible on fuller reading is `eligible: no` with the code, every other field `.`, and the quote prefixed `EXCLUDE:`.
- The tier is the highest reached. A paper that designs with FEA and then treats 12 patients is `clinical_series` (its FEA is recorded in `comp_method`, its mechanical result in `mech_result`).
- Random split of one dataset is `internal_only` for `ai_independent_eval`; an external centre or an independent public dataset is `yes`.
- Numbers go in the result fields with their units; do not round to a narrative.

S4.3 Risk-of-bias appraisal instructions

You are appraising clinical studies included in a systematic review of patient-specific skeletal implant design. Each record states its tier. Apply the tool for its tier, from the text supplied (abstract and, where present, verified full text). Never infer beyond the text: where the text gives no information, the judgement is `no_information` (ROBINS-I), `unclear` (JBI) or `some_concerns` with the note "no information" (RoB 2 has no NI category; use `some_concerns` and say so).

Tools

- `rct` (randomised trials): **RoB 2**, five domains: D1 randomisation process; D2 deviations from intended interventions; D3 missing outcome data; D4 measurement of the outcome; D5 selection of the reported result. Judgements `low`, `some_concerns`, `high`. Overall per the RoB 2 algorithm (high if any domain high; some concerns if any domain some concerns; else low).
- `clinical_comparative` (non-randomised comparative studies): **ROBINS-I**, seven domains: D1 confounding; D2 selection of participants; D3 classification of interventions; D4 deviations from intended interventions; D5 missing data; D6 measurement of outcomes; D7 selection of the reported result. Judgements `low`, `moderate`, `serious`, `critical`, `no_information`. Overall = the worst domain judgement (no_information overall only if no domain can be judged).
- `clinical_series` (non-comparative series): **JBI checklist for case series**, ten items: Q1 clear inclusion criteria; Q2 condition measured in a standard, reliable way; Q3 valid methods for identification of the condition; Q4 consecutive inclusion; Q5 complete inclusion; Q6 clear reporting of demographics; Q7 clear reporting of clinical information; Q8 outcomes or follow-up results clearly reported; Q9 clear reporting of site or clinic demographics; Q10 appropriate statistical analysis. Judgements `yes`, `no`, `unclear`, `na`. Overall = the count of `yes` items out of 10, written as `k/10`.

Output, exactly

One tab-separated line per record, in input order, no header, no commentary, no markdown:

```
ID<TAB>tool<TAB>judgements<TAB>overall<TAB>support
```

- `tool`: `rob2`, `robins_i` or `jbi_case_series`, matching the record's tier.
- `judgements`: the domain or item judgements in order, joined by `;` (five for rob2, seven for robins_i, ten for jbi).
- `overall`: as defined above.

- `support`: one short clause per domain that drove the overall judgement, citing the text; strip tabs and newlines. For abstract-only records say "abstract only" first.

Every record exactly once, ID verbatim.

S4.4 Dental boundary re-adjudication (amendment A2)

Each record below is an included study that concerns a jaw or dental implant. Decide, from the record's own text only, whether the implant's geometry (or the geometry of a load-bearing part) is derived from the INDIVIDUAL patient's anatomy (for example a root-analogue implant shaped from the extracted tooth or socket, a subperiosteal frame fitted to the patient's jaw surface, a custom reconstruction implant designed on the patient's CT). Customisation only by choosing parameters (length, diameter, thread pitch, thickness, lattice parameter) across categories such as bone quality, patient weight or loading class, without anatomy-derived geometry, is NOT patient-specific. Standard root-form dental implants and prosthodontic restorations are not skeletal implants.

Output one tab-separated line per record, IDs verbatim, nothing else:

IDeligibleexcl_codereason

- eligible: `yes` (anatomy-derived geometry established by the text), `no` (parameter-only customisation: excl_code `not_psi`; or standard dental implant or restoration: excl_code `not_skeletal`), `unclear` (the text does not settle it; excl_code `.`).
- reason: one clause quoting or closely paraphrasing the decisive text.

S4.5 Artificial intelligence validation re-adjudication

For each record (an included study applying machine or deep learning to implant design), decide from the record's own text:

1. `ai_independent_eval`: `yes` only if the model was evaluated on data NOT drawn from the development source: a different centre or institution, a public benchmark dataset different from the training source, or a prospectively collected external set. A random or patient-level split of ONE dataset from ONE source into training, validation and test sets is `internal_only`, however large the test set and however it was augmented. If the text does not describe the evaluation data's origin, `unclear`.
2. `ai_clinical_eval`: `yes` only if the model's output was used prospectively in the care or design workflow for at least one patient, with something measured; `no` if the text describes computational or bench evaluation only; `unclear` if not stated.
3. `dev_n` and `eval_n`: the number of scans, cases or subjects used for development and for the decisive evaluation, as integers, or `.`; and `eval_source`: `same_source_split`, `external_centre`, `public_benchmark`, `prospective`, `synthetic`, `unclear`.

Output one tab-separated line per record, IDs verbatim, nothing else:

IDai_independent_evalai_clinical_evaldev_neval_neval_sourcereason

S4.6 Funding and conflict charting (amendment A7)

For each record (an included study with its full text supplied), record from the text only:

- `funding`: `industry` (any manufacturer, implant company or commercial sponsor funding or provision of implants or software), `public` (government, university, foundation or charity funding only), `none_stated` (the paper states it received no funding), `mixed` (industry and public), `unclear` (no funding statement found).
- `coi`: `declared` (at least one author declares a relevant financial interest or employment, consultancy, royalties or shares with a manufacturer), `none_declared` (the paper states no conflicts), `unclear` (no statement found).
- `industry_author`: `yes` if any author affiliation is a manufacturer or commercial design service, `no`, `unclear`.

Output one tab-separated line per record, IDs verbatim, nothing else:

IDfundingcoiindustry_authorevidence (one short clause quoting the statement, or “no statement found”)

S4.7 Arm-level outcome extraction (amendment A8)

Each record is a randomised trial or comparative non-randomised study of a patient-specific skeletal implant (designed with an evaluated method) against a comparator, with full text supplied. Extract, from the text only, every outcome in the following list that is reported for BOTH arms with numbers:

- `operative_time` (minutes; mean and SD, or median and IQR/range, per arm)
- `blood_loss` (mL)
- `accuracy` (geometric deviation of the achieved result from the plan or from the intended position, in mm or degrees; mean and SD per arm; name the measure)
- `complications_any` (patients with any complication / patients per arm)
- `revision_or_removal` (implants revised or removed / patients per arm)
- `infection` (events / patients per arm)
- `function` (name the scale; mean and SD per arm at the final follow-up)

Output one tab-separated line per outcome per record, IDs verbatim, nothing else:

IDtieroutcomemeasure_namepsi_npsi_valuepsi_dispersioncomp_ncomp_valuecomp_dispersioncomparator_descriptiontimepointdispersion_typequote

- `dispersion_type`: `sd`, `iqr`, `range`, `ci95`, `none`, or `events` for count outcomes (then value = events and dispersion = `.`).
- Use `.` for anything not reported. Do not convert or impute; report exactly what the text gives. If a record reports none of the listed outcomes for both arms, output a single line with outcome `none_reported` and the other fields `.`.

Text S5. Contemporaneous log of AI use

Date	Stage	System	What it did
2026-09-02	Scoping counts	Claude Fable 5.1 (Anthropic), Claude Code	Eight PubMed hit-count queries (counts only) to size concept blocks; archived in searches/scoping-counts.md
2026-09-02	Protocol v1.0	Claude Fable 5.1	Drafted the protocol before any retrieval
2026-09-02	Search	scripts/search.py (Claude Fable 5.1 adapted from the workspace's prior review script)	PubMed and Europe PMC retrieved in full and cached; OpenAlex unavailable (HTTP 429 daily budget); Crossref bounded sweep
2026-09-02	Registry search	gpt-5.6-sol (OpenAI), Codex CLI 0.152.1, read-only, web search	PROSPERO, OSF Registries and prior systematic/scoping reviews on the topic
2026-09-02	PROSPERO record draft	gpt-5.6-sol, Codex CLI 0.152.1, read-only	Drafted the registration record from protocol.md for the author to submit
2026-09-02	Rule-layer audit	gpt-5.6-sol, Codex CLI 0.152.1, read-only	Re-screened a seeded random sample of 25 rule-excluded records: 0 false excludes
2026-09-02	Title-abstract screening, pass 1	gpt-5.6-sol (OpenAI), Codex CLI 0.152.1, reasoning high, read-only, 18 chunks of up to 120 records	Screened all 2,081 rule-passing records from screening-instructions.md
2026-09-02	Title-abstract screening, pass 2 (SUPERSEDED)	Claude Sonnet 5 (Anthropic), Claude Code subagents, 11 chunks of up to 190 records	Independently screened the same 2,081 records from the same instructions, blind to pass 1. Superseded the same day by owner ruling (Sonnet not to be used); outputs archived in searches/claude-chunks/superseded-sonnet/ and not used in the reported flow
2026-09-02	Title-abstract screening, pass 2	Claude Opus 5 (Anthropic; model id claude-opus-5, 1M context, as reported by the first agent from its system context), Claude Code subagents, same 11 chunks and the 144 chase candidates	Re-ran pass 2 from the same instructions, blind to pass 1 and to the superseded Sonnet pass
2026-09-02	Reconciliation	scripts/triage_reconcile.py	Recall-union; cross-tab, raw agreement 0.890 (3-class), kappa 0.758; include-vs-not kappa 0.816; 739 retained
2026-09-02	Citation chasing	scripts/chase.py (Claude Fable 5.1)	Reference lists of 32 systematic/scoping reviews via NCBI elink (Europe PMC references API under maintenance); 144 new candidates to dual triage
2026-09-02	Output pipeline code	gpt-5.6-sol, Codex CLI 0.152.1, write access limited to scripts/, data/, tables/, figures/, prisma/	Wrote ingest_charting.py, build_outputs.py, figures.py, references.py, resolve_citations.py, verify.py, self_test.py; self-test re-run by the orchestrator: pass
2026-09-02	Full-text retrieval	scripts/fulltext.py	PMC via E-utilities, Europe PMC, Unpaywall; abstract-only records logged
2026-09-02	Reconciliation (final)	scripts/triage_reconcile.py	Main set: sol vs Opus raw agreement 0.893 (3-class), kappa 0.781; include-vs-not kappa 0.866; 516 both-include; 781 retained. Chase increment (144): sol vs Opus kappa 0.592 on inclusion; 55 retained
2026-09-02	Full-text retrieval, defect and rerun	scripts/fulltext.py; defect caught by Claude Opus 5 second charters	The first retrieval took the wrong NCBI link set (articles citing the record) for the PMC route; 619 of 692 texts were other papers. Ten sol charting chunks and four Opus sample chunks run on those inputs were quarantined (searches/sol-jobs/charting-superseded-badfulltext, searches/claude-chunks/charting-sample-superseded-badfulltext) and not used. The fetcher was corrected (own-PMC link only; title-match guard on every route), the cache wiped and every record re-retrieved: 400 of 836 with verified full text
2026-	Charting	gpt-5.6-sol, Codex CLI 0.152.1, read-only, 70	Full-record assessment and charting of all 836

Date	Stage	System	What it did
09-02		chunks of up to 12 records	records from charting-instructions.md
2026-09-02	Second charter (sample)	Claude Opus 5, four subagents, 15 records each	Independent charting of a seeded 60-record sample for agreement
2026-09-03	Adjudication of charting disagreements	gpt-5.6-sol, Codex CLI 0.152.1, read-only, 5 chunks	Decided each of the 68 disagreeing fields (33 records) from the record text; 28 field changes applied as corrections; three records whose eligibility flipped were re-charted in full by a further sol run
2026-09-03	Re-assessment of unclear records	gpt-5.6-sol, 5 chunks of up to 13	Re-charted the 65 records first charted with unclear eligibility: 36 eligible, 19 excluded, 10 still unclear
2026-09-03	Preprint duplicates	scripts (Claude Fable 5.1) with Crossref and PubMed author lists	15 preprint versions of charted journal articles excluded as duplicates (amendment A3)
2026-09-03	Risk-of-bias appraisal	gpt-5.6-sol, 22 chunks of up to 12 plus one supplementary chunk	RoB 2, ROBINS-I and JBI case-series appraisal of the clinical-tier studies from appraisal-instructions.md
2026-09-03	Results, Discussion, Conclusions	Claude Fable 5.1	Drafted from data/canonical.json; every numeric literal checked by scripts/verify.py; references resolved by scripts and gated by scripts/verify_references.py
2026-09-03	Adversarial review, round 1	gpt-5.6-sol, Codex CLI 0.152.1, two independent runs (sol-a, sol-b), full repository read access, no write access	23 and 20 findings; converging majors: residual duplicate reports (same article under two identifiers), dental boundary applied unevenly, undisclosed meta-analysis deviation, funding and conflicts never charted, overstated descriptions of three AI studies, one-arm flow hiding the abstract-only route, blanket certainty ratings. Reports in docs/review-sol-a-r1-2026-09-03.md and docs/review-sol-b-r1-2026-09-03.md
2026-09-03	Corrections after round 1	Claude Fable 5.1 (orchestration and text); gpt-5.6-sol (dental re-adjudication, AI validation re-adjudication, funding and conflict charting from 296 full texts, arm-level outcome extraction from 41 comparative studies and trials with full text, two-arm PRISMA flow implementation); scripts	Duplicate reports excluded (amendment A6); dental and AI fields re-adjudicated; prespecified meta-analysis test run (amendment A8); outcome-level GRADE table; OpenAlex supplementary search run (amendment A9) and its increment screened, charted and appraised like the main corpus
2026-09-03	Revision v0.2	Claude Fable 5.1	Results, Discussion, Conclusions and Abstract rewritten from the rebuilt canonical data after the round-1 corrections and the OpenAlex arm; every numeric literal re-verified
2026-09-03	Adversarial review, round 2	gpt-5.6-sol, Codex CLI 0.152.1, two independent runs (sol-c, sol-d), read access to the repository, no write access	Checks resolution of the round-1 findings, regressions introduced by the corrections, and the added meta-analysis against the extracted values
2026-09-03	Corrections after round 2 (v0.3)	Claude Fable 5.1 (orchestration, text); gpt-5.6-sol (targeted re-charting of eight records; funding for three records); scripts	Report-level deduplication rerun over all assessed records (searches/duplicate-groups.json); title-only records held unresolved; meta-analysis membership taken from the charted comparator class and the plan-deviation measure, with Hartung-Knapp, leave-one-out and Egger sensitivity analyses; GRADE table with publication-bias column; AI attributions, regional claims, Text S1 and checklist locators corrected
2026-09-03	Adversarial review, round 3 (confirmation)	gpt-5.6-sol, Codex CLI 0.152.1, one run (sol-e), read-only	Checks resolution of the round-2 findings and regressions
2026-09-03	Corrections after round 3 (v0.4)	Claude Fable 5.1 (orchestration, text); gpt-5.6-sol (per-outcome re-extraction of arm-level estimates from the 40 comparative studies and trials with full text; funding for two records); scripts	The first outcome extraction had returned one outcome per study because the batch runner's footer demanded one line per record; repeated per outcome, which changed the pooled estimates (deviation from plan 7 studies; operative time 11; revision or removal, any

Date	Stage	System	What it did
			complication and infection newly poolable). One row per study per outcome with a stated measure preference; outcome-by-study disposition in Table S6. Title-only composition, the rule-audit rounding, the Bai and Beisekenov rows and the source-stratified counts corrected

Text S6. Adjudication of the plan-deviation accuracy pool

The prespecified accuracy pool combined one measure per study from the seven comparative studies and trials that reported deviation of the delivered result from the plan against a conventional implant. At revision, two adjudicators from different vendors (A: GPT-based; B: Claude-based) each read every study's methods independently, from the same written instruction, and recorded what the pooled measure was compared against (reference), the geometry measured (construct), whether a smaller value means a better result (direction), and the sentence from the paper that establishes it. A study entered the primary estimate only where both adjudicators found a virtual-plan reference and a smaller-is-better direction. Prespecified pool, 7 studies: -0.72 (95% CI -1.32 to -0.13; I-squared 76%). Restricted to a virtual-plan reference, 5 studies: -0.85 (95% CI -1.69 to -0.01; I-squared 78%). Primary, 4 studies: -1.21 (95% CI -1.69 to -0.74; I-squared 0%). Standardised mean differences are Hedges g; negative values favour the patient-specific implant.

Study	Measure pooled	Effect as submitted	Reference (A / B)	Construct (A / B)	Smaller is better (A / B)	Disposition
Abdellatif et al. 2026, PMID 42498926	overall bony landmark deviation across all planes (mm)	-1.70	virtual plan / virtual plan	landmark / landmark	yes / yes	primary estimate
Le et al. 2025, PMID 39819879	mediolateral displacement of affected condyle (mm)	-1.16	virtual plan / preoperative anatomy	landmark / positional	yes / yes	held out: adjudicators disagree on the reference
Kuehle et al. 2023, PMID 37762977	anteroposterior deviation from planned position (mm)	+0.32	virtual plan / virtual plan	positional / positional	no / no	held out: signed deviation, smaller is not better
Cassoni et al. 2022, PMID 36142107	RMSE 3D error between planned and postoperative maxillary position (mm)	-0.95	virtual plan / virtual plan	surface / surface	yes / yes	primary estimate
Möllmann et al. 2021, PMID 34900674	D1 change in bilateral lateralmost condylar-point distance (mm)	-0.02	preoperative anatomy / preoperative anatomy	landmark / landmark	no / no	held out: compares with preoperative anatomy, not the plan
Yang et al. 2021, PMID 32572853	Absolute distance deviation of reconstructed maxilla or mandible (mm)	-0.97	virtual plan / virtual plan	surface / surface	yes / yes	primary estimate
Kim et al. 2023, PMID 37138237	Mean linear distance discrepancy across 11 anatomical reference points (mm)	-1.24	virtual plan / virtual plan	landmark / landmark	yes / yes	primary estimate

Quoted evidence per study

Abdellatif et al. 2026, PMID 42498926.

Adjudicator A: This model was later used for comparison to the actual skull model derived from the postoperative CT scan for the radiographic statistical analysis.

Adjudicator B: Methods, Outcomes: "The primary outcome was the precision of reduction, by measuring linear deviations of the actual postoperative model compared to the VSP model for both study and control groups." Formula: "Deviation = absolute distance between one landmark to one plane in the actual model derived from the postoperative CT − absolute distance between one landmark to one plane in the VSP reduced model." Landmarks: "Linear deviations between predefined dental and bony landmarks to established anatomical planes were measured and underwent statistical analysis." Reported group means are positive magnitudes (0.044 vs 0.69 mm), so smaller is better.

Le et al. 2025, PMID 39819879.

Adjudicator A: The gray model represents the preoperatively planned neo-mandible. The green models depict reconstructed models from postoperative computed tomography (CT) scans.

Adjudicator B: Methods, Procedures: “Postoperative computed tomography (CT) data were compared with preoperative data.” “Superimpositions of preoperative and postoperative mandibular models were conducted using the unaffected side as a reference.” Outcome definition for the pooled row: “Displacement of the affected condyle was measured by tracking the uppermost point of the condylar head across 3D: superior-inferior, anterior-posterior, and medial-lateral (Fig. 2 A).” No plan is named for this row; the registration is preoperative model onto postoperative model. Internal conflict noted, because the figure legend says “The gray model represents the preoperatively planned neo-mandible.” and the separate condylar-axis row does name the plan: “Deviations from the preoperative plan and postoperative CT were assessed in 3D: roll (coronal), pitch (sagittal), and yaw (transverse).” That axis row is therefore the closer textual match to deviation from the virtual plan.

Adjudicator B named a closer-matching measure in the same study: coronal roll deviation of condylar axis (degrees).

Kuehle et al. 2023, PMID 37762977.

Adjudicator A: This deviation equals the translational movement that the automatic matching process (Iterative Closest Point (ICP) algorithm) determined while matching the postoperative position to the planned position of each segment.

Adjudicator B: Methods, Data Analysis: “This deviation equals the translational movement that the automatic matching process (Iterative Closest Point (ICP) algorithm) determined while matching the postoperative position to the planned position of each segment.” Direction fails because the reported anteroposterior values are signed, not absolute, with zero as target: “The segments are positioned by an average of −2.69 mm too low, with a standard deviation of over 3 mm, and too far back by 1.3 mm, with a standard deviation of 2.54 mm in the conventional group.” “The PSI segments are positioned by an average of 1.29 mm too low, with a standard deviation of 1.59 mm ($p < 0.001$), and 0.63 mm too far back, with a standard deviation of 1.62 mm ($p < 0.001$).” The better group has the larger (less negative) mean, so pooling the signed means as smaller-is-better inverts the sign.

Cassoni et al. 2022, PMID 36142107.

Adjudicator A: The mesh of the model reconstructed by postoperative CBCT (T1) and the mesh derived from VSP (T0) were imported into the same three-dimensional space (Figure 3).

Adjudicator B: Methods, Processing of Data: “A 3D virtual model was created and converted into an STL file related to the virtual surgical programming (T0) and postoperative CBCT (T1) (Figure 2).” “The two STL files were imported into Geomagic ® Control X™ 2018 software to evaluate, with an ICP algorithm, the differences in terms of the maxillary position between T0 and T1 (ΔT).” “The RMS, used as an accuracy parameter (3D error, ΔT), was considered clinically acceptable in a range of values between ±2 mm, as described in the literature by many authors [10].” Whole-surface ICP point-to-point deviation of the maxilla (ROI2) after registration on unoperated cranial anatomy (ROI1); RMS magnitude, so smaller is better.

Möllmann et al. 2021, PMID 34900674.

Adjudicator A: For evaluation, the distances of the corresponding points were measured pre- and postoperatively and tabulated. In addition, the differences of the distances were determined by subtracting the postoperative value from the value determined preoperatively.

Adjudicator B: Methods, Measuring Points and Distances: “To evaluate the accuracy of the reconstruction, the distances measured pre- and postoperatively were compared.” Selection of CT Data Sets: “For evaluation, the distances of the corresponding points were measured pre- and postoperatively and tabulated. In addition, the differences of the distances were determined by subtracting the postoperative value from the value determined preoperatively.” No virtual plan enters the measurement; D1 is the bilateral capitulum mandibulae lateralmost point distance measured on preoperative and postoperative CT. Direction also fails because the values are signed changes with zero as target: “A deviation into the negative range means a narrowing of the section in question, while a deviation into the positive range is to be interpreted as a widening in this area.”

Yang et al. 2021, PMID 32572853.

Adjudicator A: The primary end point of the study was the accuracy of reconstruction, defined as the distance or angulation deviations of anatomic landmarks between the virtual plan and the actual surgical outcome.

Adjudicator B: Methods, Outcomes: “The primary end point of the study was the accuracy of reconstruction, defined as the distance or angulation deviations of anatomic landmarks between the virtual plan and the actual surgical outcome.” “The postoperative skull model was repositioned to coincide with the preoperative virtually reconstructed skull based on the sides of maxilla or mandible not treated by surgery.” “The absolute distance deviation of the maxilla or mandible was measured by calculating the distance between pre- and postoperative models based on points, which was automatically calculated by the built-in function of the software.” Results confirm the reference: “The primary end point, accuracy of reconstruction, was derived by comparing the postoperative maxilla or mandible with the preoperative virtual models.” Here “pre-” denotes the preoperative virtually reconstructed model, i.e. the plan; absolute distance, so smaller is better.

Kim et al. 2023, PMID 37138237.

Adjudicator A: The distance of each landmark set in the simulated predictive image (T0) and the CBCT image at 7 days post-surgery (T1) was measured based on the X, Y, and Z axes (Fig. 3).

Adjudicator B: Methods, Outcome evaluation: “Accuracy was evaluated by superimposing the preoperative simulation (T0) and the CT data on the 7 th day after the operation (T1).” “A preplanned preoperative simulation STL file was used as the reference data.” “Further, the linear differences between the planned and actual movements were evaluated.” “The distance of each landmark set in the simulated predictive image (T0) and the CBCT image at 7 days post-surgery (T1) was measured based on the X, Y, and Z axes (Fig. 3).” Table 2 reports positive magnitudes per landmark (total 0.485 vs 1.213 mm), so smaller is better.

Table S1. All included studies

Study	Region	Implant	Material	Manufacturing	Pillars	Tier	Patients	Analysed units	Headline result
RONG et al. 2015. Journal of Mechanics in Medicine and Biology. doi:10.1142/s0219519415400527	knee	patient-specific knee implant	unclear	not_manufactured	comp	in_silico	0	1	stress distribution much better than hinged knee implant
Ciocca et al. 2015. Journal of cranio-maxillo-facial surgery : official publication of the European Association for Cranio-Maxillo-Facial Surgery. PMID 25434288	cmf	mandibular reconstruction plate	unclear	unclear	comp	clinical_comparative	10	10	lateral shift difference between groups not significant; operating room time and related costs reduced during fibular sectioning
Gander et al. 2015. Journal of cranio-maxillo-facial surgery : official publication of the European Association for Cranio-Maxillo-Facial Surgery. PMID 25465486	cmf	orbital floor and wall implant	titanium	am_metal	imaging+comp	clinical_series	12	12	no reoperation for implant or globe malposition; no visual impairment
Zanotti et al. 2015. Acta neurochirurgica. PMID 25588747	cranial	cranioplasty implant	ceramic	not_manufactured	comp	in_silico	0	3	implant load increased 80% with local discontinuities and 50% with general discontinuities
Abel et al. 2015. The British journal of oral & maxillofacial surgery. PMID 25703687	cmf	condylar support TMJ prosthesis	unclear	not_manufactured	comp	in_silico	0	3	CSP stresses were much lower than in the other two prostheses; bone strains were at physiological levels
Wilde et al. 2015. International journal of computer assisted radiology and surgery. PMID 25843949	cmf	mandibular reconstruction plate	titanium	machined	comp	clinical_series	30	30	regular condylar position 28/30; six plans adapted intraoperatively; median online planning time 35 min; complications included plate exposure, infection, graft or flap necrosis, and guide or plate positioning difficulties
Schepers et al. 2015. Journal of cranio-maxillo-facial surgery : official publication of the European Association for Cranio-Maxillo-Facial Surgery. PMID 25911122	cmf	mandibular reconstruction plate	unclear	unclear	comp	clinical_series	7	7	fibula mean deviation 3.0 (SD 1.8) mm and angulation 4.2° (SD 3.2°); dental implant mean deviation 3.3 (SD 1.3) mm and angulation 13.0° (SD 6.7°); resection-plane deviation 1.8 (SD 0.9) mm; flap survival 6/7
Manrique et al. 2015. The Journal of craniofacial surgery. PMID 25974770	cranial	cranioplasty implant	peek	unclear	comp	clinical_series	6	6	implant manufacturing time 2 weeks; no major or minor complications during 4-year follow-up
Aquilina et al. 2015. Craniomaxillofacial trauma & reconstruction. PMID 26000081	cmf	condylar fracture fixation plate	unclear	not_manufactured	comp	in_silico	0	3	PSI 1 had stability comparable to a single 2.0 mm straight four-hole plate
Qin et al. 2015. Journal of cranio-maxillo-facial surgery : official publication of the European Association for Cranio-Maxillo-Facial Surgery. PMID 26174791	cmf	mandibular reconstruction plate	unclear	unclear	comp	in_silico	.	.	fillet radius, cross-section, and countersink distribution affected fixing-plate strength and anti-bending capacity; an optimized structure was identified; several clinical applications successfully implemented
Sun et al. 2015. Hua xi kou qiang yi xue za zhi = Huaxi	cmf	orbital reconstruction mesh	mult	unclear	imaging+	clinical_series	18	18	no serious complications; eye and facial appearance

Study	Region	Implant	Material	Manufacturing	Pillars	Tier	Patients	Analysed units	Headline result
kouqiang yixue zazhi = West China journal of stomatology. PMID 26281256		with Medpor	iple		comp				and function recovered significantly
Zhang et al. 2015. The Journal of craniofacial surgery. PMID 26594999	cranial	cranioplasty plate	titanium	unclear	imaging+comp	clinical_series	8	8	all 8 patients satisfied
Pinheiro et al. 2015. Journal of cranio-maxillo-facial surgery : official publication of the European Association for Cranio-Maxillo-Facial Surgery. PMID 26596361	cmf	mandibular endoprosthesis	unclear	not_manufactured	comp	in_silico	0	1	displacement fields and stress distributions very similar to intact mandible
Baumann et al. 2015. Journal of oral and maxillofacial surgery : official journal of the American Association of Oral and Maxillofacial Surgeons. PMID 26608137	cmf	orbital implant	multiple	unclear	imaging+comp	clinical_series	6	6	planned and postoperative PSI positions showed good correlation; ophthalmic parameters improved in 6/6; enophthalmos adequately corrected
Capelli et al. 2016. The Spine Journal. doi:10.1016/j.spinee.2016.01.049	spine	interbody cage	multiple	not_manufactured	comp	in_silico	0	13	stress peaks below material failure thresholds under 400 N compression and 7.5 Nm bending
Gattinger et al. 2016. Current Directions in Biomedical Engineering. doi:10.1515/cdbme-2016-0025	cmf	root-analogue dental implant	unclear	am_metal	imaging+comp	bench	0	3	.
Tan et al. 2016. Journal of neurosurgery. PMID 26566203	cranial	cranioplasty plate	pmma	cast	imaging+comp	cadaver_phantom	0	1	cranial index of symmetry 96.2%
Sutradhar et al. 2016. Medical & biological engineering & computing. PMID 26660897	cmf	craniofacial bone replacement	unclear	unclear	comp	bench	0	4	survived maximum mastication load 120 lb
Eraly et al. 2016. Journal of shoulder and elbow surgery. PMID 26700554	shoulder	custom glenoid component	unclear	unclear	comp	cadaver_phantom	0	10	total intraosseous screw length 89% vs 52% of planned; angular implant deviation lower with guide ($P < .05$)
Shuang et al. 2016. Medicine. PMID 26817880	upper_limb_other	custom osteosynthesis plate	unclear	unclear	imaging+comp	rct	13	13	operative time 70.6 ± 12.1 min vs 92.3 ± 17.4 min; bone union 13/13; good or excellent elbow function 83.1% vs 71.4%; no wound infections
Moiduddin et al. 2016. Technology and health care : official journal of the European Society for Engineering and Medicine. PMID 26835729	cmf	mandibular reconstruction implant	unclear	unclear	imaging	bench	0	6	mirroring more accurate for medium and large defects; anatomical reconstruction perfect fitting for small defects
Tarsitano et al. 2016. Journal of cranio-maxillo-facial surgery : official publication of the European Association	cmf	customized mandibular reconstruction plate	unclear	unclear	imaging+comp	clinical_comparative	30	30	mean preoperative-to-postoperative differences significantly better for mandibular angle (p =

Study	Region	Implant	Material	Manufacturing	Pillars	Tier	Patients	Analysed units	Headline result
for Cranio-Maxillo-Facial Surgery. PMID 27107476									0.034), bi-gonial diameter (p = 0.041), and chin protrusion (p = 0.05); midline deviation p = 0.092
Tarsitano et al. 2016. Journal of cranio-maxillo-facial surgery : official publication of the European Association for Cranio-Maxillo-Facial Surgery. PMID 27193477	cmf	mandibular reconstruction plate	unclear	unclear	comp	clinical_comparative	40	40	mean operating time 435 min vs 550.5 min; gain 115.5 min; microvascular complications 0/20 vs 2/20; mean hospital stay 13.8 vs 17 days
Anssari Moin et al. 2016. International journal of dentistry. PMID 27274727	cmf	root analogue dental implant	titanium	not_manufactured	comp	in_silico	0	5	Plug design von Mises stress 82 MPa vertical and 168 MPa oblique; Fins micromotion 5.45 µm vertical and 6.25 µm oblique
Zimmerer et al. 2016. Journal of cranio-maxillo-facial surgery : official publication of the European Association for Cranio-Maxillo-Facial Surgery. PMID 27519662	cmf	orbital reconstruction implant	titanium	unclear	comp	clinical_comparative	195	195	variance in orbital volume difference significantly lower with individualized implants; individualization and navigation provided clinical benefit
Zhang et al. 2016. Orthopaedic surgery. PMID 27627721	spine	endplate-conformed cervical cage	unclear	unclear	imaging+comp	cadaver_phantom	0	12	lower interface stress in all directions (all $P < 0.05$); smaller FLE-EXT ROM ($P = 0.001$) but larger AR ROM ($P = 0.017$)
Suojanen et al. 2016. Journal of cranio-maxillo-facial surgery : official publication of the European Association for Cranio-Maxillo-Facial Surgery. PMID 27769722	cmf	maxillary osteosynthesis plate	titanium	am_metal	comp	clinical_series	32	32	fit excellent 23/32, good 4/32, acceptable 4/32, unusable 1/32
Oh et al. 2016. The Journal of craniofacial surgery. PMID 28005746	cmf	orbital wall mesh	multiple	unclear	imaging+comp	clinical_series	104	104	postoperative orbital volume 20.74 ± 2.07 cm³ vs uninjured side 21.39 ± 1.93 cm³; all 104 reconstructions successful; no postoperative complications
Luongo et al. 2016. BioMed research international. PMID 28070512	cmf	alveolar ridge scaffold	ceramic	machined	imaging+comp	clinical_series	15	15	pain/swelling 2/15 (13.3%); scaffold exposure 3/15 (20.0%); complete graft loss 1/15 (6.6%); implant survival 14/14 (100%)
Mommaerts et al. 2016. Annals of maxillofacial surgery. PMID 28299273	cmf	mandibular angle and border augmentation implant	titanium	am_metal	imaging+comp	clinical_series	12	17	complications in 3/12; implant removal in 1/12; all 12 patients were satisfied
Jahadakbar et al. 2016. Bioengineering (Basel, Switzerland). PMID 28952598	cmf	mandibular fixation plate	multiple	am_metal	comp	cadaver_phantom	0	1	maximum actual von Mises stress 594 MPa; safety factor 1.7; contact pressure 177% higher with porous NiTi
Alike et al. 2016. Zhongguo xiu fu chong jian wai ke za zhi = Zhongguo xiufu chongjian waike zazhi = Chinese journal of reparative and reconstructive surgery. PMID 29786331	multi	customized bone cement prosthesis	unclear	cast	imaging+comp	clinical_series	13	13	bone cement prosthesis-bone interface match index 91.2%-94.7% (mean 93.2%); all incisions healed; no prosthesis fracture or peripheral fracture at follow-up
Díaz et al. 2017. IFMBE Proceedings.	crania	cranioplasty plate	mult	not_manufactu	comp	in_silico	0	2	implant 2 maximum stress/displacement: PEEK

Study	Region	Implant	Material	Manufacturing	Pillars	Tier	Patients	Analysed units	Headline result
doi:10.1007/978-981-10-4086-3_102	l		iple	red					32.575 MPa/0.1448 mm; Ti6Al4V 47.034 MPa/0.0050 mm
Wilk et al. 2017. American Journal of Engineering and Applied Sciences. doi:10.3844/ajeassp.2017.13.19	lower_limb_other	segmental femoral implant	titanium	am_metal	comp	bench	0	2	mean printed pore diameter 16% smaller than computational model; effective elastic modulus 20.8 and 10.5 GPa versus predicted 17.6 and 13.6 GPa; compression loads 600 and 1200 N
De Maesschalck et al. 2017. European archives of oto-rhino-laryngology : official journal of the European Federation of Oto-Rhino-Laryngological Societies (EUFOS) : affiliated with the German Society for Oto-Rhino-Laryngology - Head and Neck Surgery. PMID 27501991	cmf	mandibular reconstruction plate	unclear	unclear	imaging+comp	clinical_comparative	18	18	mandibular osteotomy mean distance deviation 2.3 mm; fibular osteotomy mean distance deviation 1.9 mm; CAS and freehand reconstruction were morphologically comparable except nonaffected-side axial angle (1.0° vs 2.9°; P = 0.03)
Gutwald et al. 2017. Computer methods in biomechanics and biomedical engineering. PMID 27887036	cmf	mandibular reconstruction plate	titanium	unclear	comp	bench	0	13	maximum stress 573 MPa standard vs 393 MPa customized; median fatigue limit 475 ± 27 N vs 650 ± 27 N
Vanden Berghe et al. 2017. Computer methods in biomechanics and biomedical engineering. PMID 27957883	pelvis_acetabulum	acetabular custom implant	unclear	not_manufactured	imaging+comp	imaging_dataset	0	.	hip joint centre reconstruction error 2.6 mm; acetabular radius reconstruction error 0.7 mm
Moser et al. 2017. Journal of cranio-maxillo-facial surgery : official publication of the European Association for Cranio-Maxillo-Facial Surgery. PMID 28011181	cranial	cranioplasty plate	pmma	cast	comp	clinical_series	17	17	mean implant volume 66.5 vs 72 cm3, p = 0.513; mean thickness difference 0.4-1.8 mm; complications 3/17 (17.6%); surgical-site infections 2/17 (11.8%); implant removal 1/17
Saravana Kumar et al. 2017. Proceedings of the Institution of Mechanical Engineers. Part H, Journal of engineering in medicine. PMID 28056711	hip	cementless hip stem	unclear	not_manufactured	comp	in_silico	0	.	stress-shielding decreased as elastic modulus distribution was optimized
Dharme et al. 2017. The International journal of artificial organs. PMID 28106226	hip	customized hip stem	unclear	unclear	comp	bench	0	147	HO, VO, and NSA accounted for 54.38%, 16.33%, and 29.16% of variation in fatigue safety factors
Kim et al. 2017. Journal of cranio-maxillo-facial surgery : official publication of the European Association for Cranio-Maxillo-Facial Surgery. PMID 28434826	cmf	prebent orbital-wall mesh	titanium	unclear	imaging	clinical_comparative	82	82	postoperative defect area 8.03 ± 3.5% vs 18.7 ± 15.41% for medial-wall fractures and 7.14 ± 5.74% vs 12.8 ± 4.92% for inferior-wall fractures; p < 0.01
Fu et al. 2017. Journal of oral and maxillofacial surgery : official journal of	cmf	individualized maxillary and orbital-floor mesh	titaniu	unclear	comp	clinical_series	6	6	all 6 flaps survived; symmetrical facial contours, normal lower-jaw

Study	Region	Implant	Material	Manufacturing	Pillars	Tier	Patients	Analysed units	Headline result
the American Association of Oral and Maxillofacial Surgeons. PMID 28463685			m						movement, and proper vertical distance for dental implants in all patients
Phanindra Bogu et al. 2017. Medical & biological engineering & computing. PMID 28474182	cranial	porous cranioplasty plate	multiple	not_manufactured	comp	in_silico	0	6	titanium solid implant performed best overall; PEEK Type 5 with 45.73% porosity was preferred considering weight and osseointegration
Rana et al. 2017. Journal of cranio-maxillo-facial surgery : official publication of the European Association for Cranio-Maxillo-Facial Surgery. PMID 28552201	cmf	mandibular reconstruction plate	unclear	am_metal	comp	clinical_series	22	22	mean pre- to postoperative intercondylar-distance difference 1.2 ± 1.4 mm; fibula graft shaping time 41.6 ± 4.9 min; wound-healing disturbance or plate exposure in 8/22; all PSFIs remained in situ during follow-up
Li et al. 2017. International journal of oral and maxillofacial surgery. PMID 28552440	cmf	personalized orthognathic fixation plates	titanium	am_metal	imaging+comp	clinical_series	10	10	largest RMSD 1.10 mm positional and 2.82° orientational; CASS planning median 40 min; POSG design median 80 min; 10/10 operations completed; no plate breakage; uneventful healing
Bogu et al. 2017. Acta of bioengineering and biomechanics. PMID 28552926	cranial	cranioplasty plate	multiple	not_manufactured	comp	in_silico	0	6	Ti6Al4V showed low deformation and PEEK showed less equivalent stress
Xu et al. 2017. Medical engineering & physics. PMID 28629601	cmf	total TMJ replacement prosthesis	multiple	am_metal	comp	in_silico	0	1	compressive strain at the condyle neck was lower than in the intact mandible except near screws; strain was relatively high at the scaffold-bone interface
Iqbal et al. 2017. Proceedings of the Institution of Mechanical Engineers. Part H, Journal of engineering in medicine. PMID 28639517	pelvis_acetabulum	hemi-pelvic prosthesis	unclear	not_manufactured	comp	in_silico	0	1	peak stresses 20%-40% larger in affected-side single-leg standing; prosthesis peak stresses two- to threefold those of normal pelvis but below allowable fatigue limitation
Chen et al. 2017. Scientific reports. PMID 28646207	cranial	cranioplasty implant	unclear	unclear	imaging+comp	in_silico	0	20	intra-rater overlap 87.07 ± 1.6%; inter-rater overlap 87.73 ± 1.4%; design time 26.7 ± 2 min vs 137.8 ± 5 min traditional software
Byrne et al. 2017. The Journal of hand surgery. PMID 28709790	upper_limb_other	forearm fixation plate	titanium	am_metal	imaging+comp	clinical_series	5	5	mean correction error 1.8° ulna and 1.4° radius; supination improved 47° to 89°; pronation 68° to 87°; pain and grip strength improved significantly
Fan et al. 2017. Graefe's archive for clinical and experimental ophthalmology = Albrecht von Graefes Archiv fur klinische und	cmf	orbital Medpor-titanium implant	multiple	unclear	imaging	clinical_comparative	56	56	maximum width, depth, area and medial-inferior wall angle errors significantly lower than control; average surgical duration

Study	Region	Implant	Material	Manufacturing	Pillars	Tier	Patients	Analysed units	Headline result
experimentelle Ophthalmologie. PMID 28786025									substantially shorter than control; postoperative clinical evaluation superior to control
Heufelder et al. 2017. Journal of cranio-maxillo-facial surgery : official publication of the European Association for Cranio-Maxillo-Facial Surgery. PMID 28793965	cmf	maxillary osteosynthesis implant	unclear	unclear	imaging+comp	clinical_series	22	22	median 3D maxillary position deviation 0.39 mm; left-right 0.30 mm, up-down 0.33 mm, anterior-posterior 0.7 mm
Raisian et al. 2017. Reviews on recent clinical trials. PMID 28828975	cmf	orbital titanium mesh	titanium	unclear	imaging	rct	10	10	mean enophthalmos 0.35 ± 0.4 mm vs 2.4 ± 0.8 mm at 4 mo; preoperative vertical dystopia or diplopia resolved in 2/5 intervention patients
Koh et al. 2017. Bone & joint research. PMID 28947604	knee	bicruciate-retaining total knee prosthesis	multiple	not_manufactured	comp	in_silico	0	1	femoral rollback difference from normal 1.4 mm patient-specific vs 4.9 mm bicruciate-retaining and 9.8 mm cruciate-retaining; tibial rotation difference 1.4° vs 3.4° and 5.8°
Kang et al. 2017. Journal of the American Academy of Orthopaedic Surgeons. Global research & reviews. PMID 30211354	knee	patient-specific total knee arthroplasty	multiple	not_manufactured	comp	in_silico	0	1	medial insert contact stress increased 27% OTS vs 13% PS with 5° varus malalignment and 11% OTS vs 6% PS with 5° internal malrotation
George et al. 2018. . doi:10.1109/raics.2018.8635093	multi	knee and cranial implants	multiple	am_polymer	comp	bench	0	2	knee implant life about 108 cycles; cranial implant withstands deceleration 5 g
Dai et al. 2018. Journal of Mechanics in Medicine and Biology. doi:10.1142/s0219519418500240	cmf	maxillofacial prosthesis	unclear	unclear	comp	bench	0	.	.
Dahake et al. 2018. The international journal of medical robotics + computer assisted surgery : MRCAS. PMID 28868631	cmf	mandibular reconstruction implant	unclear	not_manufactured	comp	in_silico	0	12	maximum-gap case had 37% higher screw strain and 28% higher implant stress than zero-gap case
Hingsammer et al. 2018. Journal of the mechanical behavior of biomedical materials. PMID 28888931	cmf	Le Fort I osteosynthesis miniplates and screws	multiple	unclear	comp	in_silico	0	2	safety factor 2.13 for zirconium dioxide vs 4.51 for titanium at 500 N
Khosravipour et al. 2018. Proceedings of the Institution of Mechanical Engineers. Part H, Journal of engineering in medicine. PMID 29191076	knee	surface-guided knee implant	polymer_other	unclear	comp	bench	0	1	peak von Mises stresses 16.28 MPa at 90° and 29.55 MPa at 120°; average contact pressure 5.51 and 5.46 MPa FEA vs 5.67 and 8.14 MPa film
Fan et al. 2018. Proceedings of the Institution of Mechanical Engineers. Part H, Journal of engineering in medicine. PMID 29216793	lower_limb_othe	femoral locking plate	multiple	not_manufactured	comp	in_silico	0	.	optimal design used bicortical locking screws, Ti-6Al-4V, 4.75-mm plate thickness, 2 mm fracture gap, and 1 mm interface gap

Study	Region	Implant	Material	Manufacturing	Pillars	Tier	Patients	Analysed units	Headline result
Kim et al. 2018. Journal of plastic, reconstructive & aesthetic surgery : JPRAS. PMID 29233510	cmf	orbital floor and medial wall implant	multiple	unclear	imaging	clinical_comparative	28	28	orbital volume ratio decreased from 127.6% to 99.79% in comminuted group and from 117.03% to 101.3% in intact group (p < 0.05); diplopia improved in 9/10 patients; moderate-to-severe enophthalmos improved in 18/18 patients
Wang et al. 2018. Aesthetic plastic surgery. PMID 29260271	cmf	mandibular contour implant	polymer_other	unclear	comp	clinical_series	12	12	delayed infection and implant removal in 1/12; satisfactory contour reconstruction in 11/12
Kang et al. 2018. Bone & joint research. PMID 29305427	knee	medial unicompartmental knee prosthesis	multiple	not_manufactured	comp	in_silico	0	3	cortical rim coverage >95%; swing-phase lateral cartilage stress 7% above healthy for PS vs 32% for standard; lateral meniscus 5% vs 28%
Maini et al. 2018. Archives of orthopaedic and trauma surgery. PMID 29368178	pelvis_acetabulum	acetabular fixation plate	unclear	unclear	comp	rct	25	25	satisfactory/anatomical reduction in a higher percentage than conventional contouring; surgical duration lower than conventional contouring; total blood loss lower than conventional contouring
Wang et al. 2018. Proceedings of the Institution of Mechanical Engineers. Part H, Journal of engineering in medicine. PMID 29383959	spine	vertebral body replacement	unclear	not_manufactured	comp	in_silico	0	16	maximum von Mises stress 71 MPa flat vs 34 MPa anatomical design under flexion/extension
Yang et al. 2018. Oral oncology. PMID 29496055	cmf	maxillary or mandibular reconstruction plate	unclear	unclear	comp	clinical_series	10	10	mean absolute distance deviation 1.40 ± 0.63 mm; intraoperative success 100%; no major adverse events
Day et al. 2018. Plastic and reconstructive surgery. Global open. PMID 29707444	multi	craniofacial augmentation implant	multiple	am_polymer	imaging+comp	clinical_series	13	13	no implant extrusion, implant-associated abscess, seroma, hematoma, or implant removal
Kang et al. 2018. Biomechanics and modeling in mechanobiology. PMID 29730771	chest_wall	rib prosthesis	peek	am_polymer	comp	bench	1	3	maximum von Mises stress 39.88 MPa for C-design; deformation difference up to 20% between FEA and experiment; discharged 10 days after surgery; postoperative CT showed stable reconstruction
Chana Rodríguez et al. 2018. Revista espanola de cirugia ortopedica y traumatologia (English ed.). PMID 29807784	pelvis_acetabulum	acetabular fixation plate	unclear	unclear	imaging+comp	clinical_series	20	20	complete implant contact in 19/20 cases; partial contact in 1/20; model printing 385 min mean (range 322-539 min); osteosynthesis 16.9 min mean (range 10-24 min); anatomic reduction in 20/20 cases; no intra-operative contouring

Study	Region	Implant	Material	Manufacturing	Pillars	Tier	Patients	Analysed units	Headline result
									changes
Dimitroulis et al. 2018. Journal of cranio-maxillo-facial surgery : official publication of the European Association for Cranio-Maxillo-Facial Surgery. PMID 29859818	cmf	TMJ total joint prosthesis	unclear	unclear	comp	clinical_series	38	50	mean 74.4% reduction in joint pain; no device failures
Calvo-Echenique et al. 2018. Computer methods and programs in biomedicine. PMID 29903488	spine	interbody cage	unclear	not_manufactured	comp	in_silico	0	1	wider and anterior cages increased failed-bone volume under 500 N preload and 7.5 Nm multidirectional rotation
Morales-Gómez et al. 2018. Journal of neurosurgery. PMID 29905512	cranial	cranioplasty plate	pmma	cast	comp	clinical_series	22	22	infection 0/22; implant rejection 0/22; wound dehiscence 0/22; implant removal 0/22
Chen et al. 2018. Journal of cranio-maxillo-facial surgery : official publication of the European Association for Cranio-Maxillo-Facial Surgery. PMID 30025603	cmf	customized total temporomandibular joint prosthesis	multiple	unclear	comp	in_silico	0	2	maximum stress 19.61 MPa in UHMWPE, 170.01 MPa in mandibular component, and peak von Mises stress 236.08 MPa in topside screw
Kärkkäinen et al. 2018. The British journal of oral & maxillofacial surgery. PMID 30220611	cmf	patient-specific orbital implant	titanium	machined	comp	clinical_series	15	15	mean planned-to-postoperative implant-position difference 1.9 mm (range 0.5-5.6 mm); fractured-orbit volume 1.34 ml less than contralateral side; no reoperations; no implant-related complications
Vlok et al. 2018. South African journal of surgery. Suid-Afrikaanse tydskrif vir chirurgie. PMID 30264941	cranial	cranioplasty plate	pmma	cast	comp	clinical_series	60	60	100% accurate implant-to-defect ratio; sepsis 5/60 (8.3%); graft removal 2/60
Koh et al. 2018. Journal of experimental orthopaedics. PMID 30578465	knee	patient-specific posterior-stabilized total knee arthroplasty	multiple	not_manufactured	comp	in_silico	0	10	APSV-TKA femoral rollback 11.9 mm at 120° flexion; maximum tibial internal rotation approximately 12.5°
Willing et al. 2018. Orthopaedic Proceedings. DESIGN OPTIMISATION OF PATIENT-SPECIFIC DISTAL HUMERAL HEMIARTHROPLASTY IMPLANTS	upper_limb_other	distal humeral hemiarthroplasty implant	unclear	not_manufactured	comp	in_silico	0	6	population-optimal offset 0.72 mm; specimen-optimal offsets 0.52–1.04 mm; optimized designs increased contact area vs BRE but not to intact joint
Morais et al. 2018. Portuguese National Funding Agency for Science, Research and Technology (RCAAP Project by FCT). Automated computer-aided design of cranial implants: a deep learning approach	cranial	cranial implant	unclear	not_manufactured	ai	imaging_dataset	0	.	.
Hosni et al. 2019. UNC Libraries. doi:10.17615/s5er-hk90	knee	custom femoral knee component	unclear	not_manufactured	comp	in_silico	0	1	about 40% less bone removal vs conventional design; more even bone-implant interface stress

Study	Region	Implant	Material	Manufacturing	Pillars	Tier	Patients	Analysed units	Headline result
									distribution
Леонид et al. 2019. Vibroengineering PROCEDIA. doi:10.21595/vp.2019.20961	pelvis_acetabulum	custom revision hip implant	titanium	not_manufactured	comp	in_silico	0	1	implant maximum stress 326.5 MPa under 880 N double-supported load
Moiduddin et al. 2019. Applied Sciences. doi:10.3390/app9122513	cranial	cranioplasty plate	titanium	am_metal	imaging+comp	bench	0	1	maximum von Mises stress 2.5 MPa; strain 1.49 × 10−4; deformation 3.26 × 10−6 mm
Oldřich et al. 2019. arXiv (Cornell University). doi:10.48550/arxiv.1911.08805	cranial	cranioplasty implant	unclear	not_manufactured	ai+imaging	imaging_dataset	0	.	.
Omidi et al. 2019. Engineering Solid Mechanics. doi:10.5267/j.esm.2018.10.001	cmf	TMJ prosthesis	titanium	not_manufactured	comp	in_silico	0	3	anatomical TMJ prosthesis generated lower stress than the commercial TMJ prosthesis
Weber et al. 2019. International orthopaedics. PMID 30306217	pelvis_acetabulum	custom-made acetabular implant	unclear	unclear	comp	clinical_series	11	11	mean planned-to-final deviation 3.6° ± 2.8° inclination, -1.2° ± 7.0° anteversion; centre-of-rotation x 0.3 ± 3.9 mm, y -1.1 ± 3.8 mm, z 0.4 ± 3.2 mm
Koh et al. 2019. Artificial organs. PMID 30375662	knee	customized cruciate-retaining total knee arthroplasty	unclear	not_manufactured	comp	in_silico	0	3	contact stress 18%/8% lower during gait and 24%/9% lower during deep knee bend, medial/lateral
Schneider et al. 2019. Clinical oral investigations. PMID 30443778	cmf	pre-bent orthognathic fixation plate	unclear	unclear	comp	rct	21	21	SNA P<0.001; SNB P=0.002; ANB P<0.001 favoring VSP; VSP reduced operation duration; P=0.041
Liu et al. 2019. The Journal of arthroplasty. PMID 30497901	pelvis_acetabulum	pelvic reconstruction implant	unclear	unclear	comp	clinical_comparative	38	38	greater tumor resection accuracy than common 3D-printed template; shorter operating time than common 3D-printed template; lower tumor recurrence but higher implant loosening than common 3D-printed template
Abdelaal et al. 2019. The International journal of artificial organs. PMID 30537876	hip	patient-specific femoral stem	unclear	not_manufactured	comp	in_silico	0	1	close geometrical fit and fill between femoral canal and stem surfaces; peak micromotion 21 µm custom vs 34 µm conventional; conventional stem up to 89% higher load transfer than custom stem
Caiti et al. 2019. Medical & biological engineering & computing. PMID 30588576	upper_limb_othe	patient-specific distal radius fixation plate	titanium	not_manufactured	comp	in_silico	0	5	maximum stress reduced 31% under torsion; 1.9-mm plate median interfragmentary strain 2.14% (IQR 0.2) under bending; maximum stress

Study	Region	Implant	Material	Manufacturing	Pillars	Tier	Patients	Analysed units	Headline result
	r								below 82% of yield stress
Zhong et al. 2019. Microscopy research and technique. PMID 30629776	upper_limb_other	personalized anatomical distal humerus locking plate	unclear	unclear	comp	in_silico	1	1	peak humerus stress and displacement lower with DPALP than YCP; peak screw stress higher with DPALP; less operation time with DPALP (no value reported); Broberg and Morrey Score 83 in 1 patient
Ridwan-Pramana et al. 2019. The Journal of craniofacial surgery. PMID 30676441	cranial	cranioplasty plate	pmma	cast	comp	clinical_series	16	16	no implant removal in 16 patients
Đurić et al. 2019. British journal of neurosurgery. PMID 30732480	cranial	cranioplasty plate	pmma	cast	comp	clinical_series	29	29	satisfactory aesthetic outcome 28/29 (96.5%); no infections
Maricevich et al. 2019. Surgical neurology international. PMID 30775055	cranial	cranioplasty plate	pmma	cast	comp	clinical_series	63	63	intraoperative molding within a few minutes; infection 2/63 (3.2%); extrusion 3/63 (4.8%); reoperation 11/63 (17.4%); implant removal 3/63 (4.8%)
Luenam et al. 2019. Journal of orthopaedic surgery (Hong Kong). PMID 30798711	upper_limb_other	individualized radial head prosthesis	unclear	not_manufactured	imaging	cadaver_phantom	0	11	no statistically significant difference in head diameter, dish diameter, articular depth, head thickness, end-plane angle, offset, or head volume
Rückschloß et al. 2019. Journal of cranio-maxillo-facial surgery : official publication of the European Association for Cranio-Maxillo-Facial Surgery. PMID 30894302	cmf	maxillary patient-specific fixation implant	unclear	unclear	comp	clinical_comparative	18	18	left/right 0.51 mm ± 0.48 vs 1.11 mm ± 1.32; anterior/posterior 0.39 mm ± 0.26 vs 1.42 mm ± 0.87; up/down 0.44 mm ± 0.31 vs 0.62 mm ± 0.47; rotations <2°
Desai et al. 2019. The Journal of craniofacial surgery. PMID 30950944	cranial	cranioplasty implant	pmma	cast	comp	clinical_comparative	30	30	craniofacial symmetry improvement satisfactory in 92.3%; infection 13.3%; patient satisfaction excellent in 69%; 1 resurgery in alginate-impression group
Koh et al. 2019. Bone & joint research. PMID 30997041	knee	patient-specific unicompartmental knee arthroplasty	multiple	not_manufactured	comp	in_silico	0	3	wear rates 9.2, 6.8, and 4.3 mm3/million and volumetric wear 42.7, 31.6, and 19.9 mg for nonconforming, anatomy-mimetic, and conforming designs after 5 million cycles
Kim et al. 2019. Annals of plastic surgery. PMID 31082835	cmf	orbital wall implant	unclear	unclear	imaging+comp	clinical_comparative	56	56	preoperative injured-site orbital volume ICC 0.906 (95% CI 0.840-0.945); postoperative ICC 0.867 (95% CI 0.773-0.922)
Barbera et al. 2019. The International journal of artificial organs. PMID 31122108	pelvis_acetabulu	trabecular hemipelvis implant	titanium	am_metal	comp	bench	0	2	model-test differences below 5.5%; higher-density cell unit 14% stiffer; cell-unit orientation changed stiffness up to 10%

Study	Region	Implant	Material	Manufacturing	Pillars	Tier	Patients	Analysed units	Headline result
Koh et al. 2019. Journal of experimental orthopaedics. PMID 31161463	knee	cruciate-retaining total knee implant	multiple	not_manufactured	comp	in_silico	0	4	APS TKA differed from normal by 1.6 mm anterior translation and 1.5° internal rotation during gait swing; rollback difference 4.1 mm and internal rotation 2.3° smaller during deep knee bend
Iqbal et al. 2019. Medical engineering & physics. PMID 31229384	pelvis_acetabulum	hemi-pelvic tumour prosthesis	unclear	unclear	comp	in_silico	1	.	optimized implants withstood static loading for six activities with fewer stress-concentration areas; implantation successful in 1 patient
Low et al. 2019. The Journal of craniofacial surgery. PMID 31261343	cranial	hybrid cranioplasty implant	multiple	cast	comp	clinical_series	11	11	mean intra-operative reconstruction time 30 min; mean cosmesis VAS 91; SF-36 quality of life improved in 11/11
Guo et al. 2019. Zhongguo gu shang = China journal of orthopaedics and traumatology. PMID 31277543	pelvis_acetabulum	pelvic tumour prosthesis	titanium	not_manufactured	comp	in_silico	0	1	pelvis maximum von Mises stress 39.0, 202.8, and 42.4 MPa and displacement 0.199, 0.766, and 0.847 mm; prosthesis stress 62.3, 318, and 468 MPa under three load conditions
Zhang et al. 2019. Computer methods in biomechanics and biomedical engineering. PMID 31304779	lower_limb_other	bio-fixed tibial implant	unclear	am_metal	comp	bench	0	.	strain performance equivalent to traditionally modelled material; more even stress distribution
Wang et al. 2019. Proceedings of the Institution of Mechanical Engineers. Part H, Journal of engineering in medicine. PMID 31319772	knee	total knee implant	unclear	not_manufactured	comp	in_silico	0	4	custom implant produced larger femoral external rotation and posterior translation than symmetric implant; compressive force was larger after 50° flexion
Saeidi et al. 2019. Tuwhera (Auckland University of Technology). Numerical and Experimental Investigation of a New Load-sharing Implant for Early	knee	load-sharing knee implant	unclear	unclear	comp	cadaver_phantom	0	.	maximum femoral cartilage von Mises stress reduced 40%; contact pressure reduced 35%; medial load reduced approximately 18%
Gillingham et al. 2019. SUNScholar (Stellenbosch University). Retrospective study on mandible morphology towards improving implant design	cmf	mandibular reconstruction implant	unclear	not_manufactured	imaging+comp	imaging_dataset	0	.	SSM accuracy did not differ significantly from mirroring (reported $p<0.05$)
Oldhoff et al. 2019. Research Repository (Delft University of Technology). Design and optimisation of an additively manufactured patient-specific partial m	cmf	mandibular reconstruction implant	titanium	not_manufactured	imaging+comp	imaging_dataset	0	35	SSM and mirroring gave closest estimates; no significant difference; stress concentrations and displacements maintained vs healthy intact mandible

Study	Region	Implant	Material	Manufacturing	Pillars	Tier	Patients	Analysed units	Headline result
Tilton et al. 2020. Additive manufacturing. doi:10.1016/j.addma.2020.101137	shoulder	proximal humerus fracture fixation plate	unclear	am_metal	comp	bench	0	.	average medial fracture gap displacement reduced 47.2%; screw cut-out risk reduced 14.6% versus conventional plate
Du et al. 2020. Engineering. doi:10.1016/j.eng.2020.02.019	cmf	craniomaxillofacial reconstruction implant	unclear	am_metal	comp	clinical_series	41	41	surgery time and recovery time reduced (no values reported); 41 surgical cases successfully conducted
Suchyta et al. 2020. Journal of Clinical and Translational Science. doi:10.1017/cts.2020.325	cmf	orbital floor implant	unclear	unclear	imaging+comp	cadaver_phantom	0	10	average postoperative deviation from plan was not significantly different for Black Bone MRI versus CT
Solovev et al. 2020. IOP Conference Series Materials Science and Engineering. doi:10.1088/1757-899x/747/1/012076	pelvis_acetabulum	revision acetabular implant	unclear	unclear	comp	in_silico	0	1	stress distributions compared for projected versus actual implant positions under two-leg standing
Леонид et al. 2020. IOP Conference Series Materials Science and Engineering. doi:10.1088/1757-899x/986/1/012035	hip	tumour endoprosthesis	unclear	not_manufactured	comp	in_silico	0	1	.
Kargarnejad et al. 2020. . doi:10.1109/icbme51989.2020.9319325	cmf	mandibular reconstruction implant	unclear	unclear	comp	in_silico	0	1	plate maximum von Mises stress 106 MPa and deformation 54.42 µm; cortical bone maximum stress 44 MPa and strain 1940 µm/m
Seebach et al. 2020. Journal of Medical Devices. doi:10.1115/1.4049193	cmf	mandibular osteosynthesis plate	titanium	am_metal	comp	bench	0	.	.
Zhu et al. 2020. . doi:10.21203/rs.2.23817/v1	lower_limb_other	custom talar component	cocr	not_manufactured	comp	in_silico	0	2	maximum von Mises stress 120.48 vs 68.11 MPa under load 1 and 166.81 vs 117.92 MPa under load 2, Type-1 vs Type-2
Wong et al. 2020. Applied Sciences. doi:10.3390/app10124098	lower_limb_other	distal femoral segmental-defect implant	titanium	not_manufactured	comp	in_silico	0	1	maximum implant von Mises stress 1318 MPa; overall construct deformation 4.8 mm postoperative, 2.5 mm after fracture healing, and 2.1 mm after plate removal
Wang et al. 2020. Applied Sciences. doi:10.3390/app10144738	cmf	zygomaticomaxillary fracture repair implant	unclear	not_manufactured	comp	in_silico	0	1	maximum bone-stress variation 2.75–26.78% for PSRT versus intact model and 30.67–96.26% for mini-plates under 250 N
Li et al. 2020. International journal of oral and maxillofacial surgery. PMID 31353173	cmf	genioplasty fixation plate	titanium	am_metal	comp	clinical_series	15	15	largest RMS difference 0.69 mm mediolateral translation and 2.01° yaw; 15/15 operations completed

Study	Region	Implant	Material	Manufacturing	Pillars	Tier	Patients	Analysed units	Headline result
									successfully; no difficulty using patient-specific plates
Dahake et al. 2020. The international journal of medical robotics + computer assisted surgery : MRCAS. PMID 31675463	cmf	mandibular reconstruction implant	unclear	unclear	imaging+comp	cadaver_phantom	0	.	statistically significant improvement with the customized surgical osteotomy guide versus without; minimal bone-implant junction gap
Chen et al. 2020. Ophthalmic plastic and reconstructive surgery. PMID 31789791	cmf	orbital reconstruction implant	polymer_other	cast	imaging	clinical_series	9	9	improved ductions, facial appearance and symmetry; orbital hematoma 1/9 resolved within weeks
Seier et al. 2020. International journal of oral and maxillofacial surgery. PMID 31870519	cmf	mandibular reconstruction plate	unclear	unclear	comp	clinical_series	21	21	dental implant survival 97.4% (74/76); wound infection requiring secondary intervention 23.8%
Mangano et al. 2020. 3D printing in medicine. PMID 31915946	cmf	subperiosteal jaw implant	titanium	am_metal	comp	clinical_series	10	10	mean surgeon-rated fit 7/10 (SD 1.6); 2/10 insufficient fit; mean intervention duration 44.3 min (SD 19.4); implant survival 100%; complications 3/10
El Morsy et al. 2020. Clinical implant dentistry and related research. PMID 32103625	cmf	alveolar ridge augmentation sheet	peek	unclear	comp	clinical_series	14	34	mean vertical bone gain 3.47 mm (SD 1.46); mean horizontal bone gain 3.42 mm (SD 1.1); wound breakdown 1/14; dental implants successfully placed after sheet removal
Liu et al. 2020. The British journal of oral & maxillofacial surgery. PMID 32156446	cmf	maxillary osteosynthesis plate	unclear	unclear	comp	clinical_comparative	18	18	largest Le Fort I discrepancies 0.50 (0.18) mm anteroposterior and 0.82 (0.60)° yaw vs 1.32 (1.40) mm superioinferior and 8.48 (7.73)° yaw
Merema et al. 2020. Oral diseases. PMID 32176821	cmf	mandibular reconstruction plate	titanium	machined	imaging+comp	clinical_series	8	8	mean screw entry point deviation 1.54 mm (SD 0.85, range 0.10-3.19); mean angular deviation 5.76° (SD 3.27, range 1.26-16.62); conventional plate yield-strength exceedance 42%-153% and ultimate-strength exceedance 13%-100%; patient-specific screw forces reduced 20%-66%; no mechanical failure among 7 surviving patients; flange dehiscence 2/8
Soni et al. 2020. Indian journal of orthopaedics. PMID 32257031	lower_limb_other	femoral shaft fixation plate	multiple	not_manufactured	comp	in_silico	0	3	maximum implant and screw von Mises stress 18.62 MPa titanium, 19.40 MPa stainless steel and 27.40 MPa cobalt-chromium-molybdenum under 500 N
Koh et al. 2020. BioMed	kn	patient-specific	m	not_ma	com	in_silic	0	3	AM-PS UKA showed 0.39

Study	Region	Implant	Material	Manufacturing	Pillars	Tier	Patients	Analysed units	Headline result
research international. PMID 32258105	ee	medial unicompartmental knee prosthesis	multiple	nufactured	p	o			mm translation and 0.36° rotation decreases versus the natural knee during swing phase
Cheng et al. 2020. Journal of the mechanical behavior of biomedical materials. PMID 32279058	cmf	mandibular bone analog implant	polymer_other	am_polymer	comp	in_silico	0	2	maximum stress decreased 67.9%, 71.9% and 68.6% vs fibular graft model under the three loading locations
Li et al. 2020. Journal of the mechanical behavior of biomedical materials. PMID 32279847	cmf	mandibular reconstruction implant with dental prosthesis	unclear	am_metal	comp	bench	0	2	axial/oblique maximum stress 764/720 MPa vs 778/925 MPa; resistant force 2163 N vs 1678 N
Biswas et al. 2020. Current medical imaging. PMID 32410539	spine	patient-specific pedicle screw fixation	unclear	not_manufactured	ai+comp	in_silico	0	.	microstrain at six peri-implant positions optimized toward intact-bone strain
Durand-Hill et al. 2020. Journal of orthopaedic research : official publication of the Orthopaedic Research Society. PMID 32427351	pelvis_acetabulum	custom acetabular component	titanium	am_metal	imaging+comp	clinical_series	20	20	100% within 10 mm of planned center of rotation; 95% within 10° rotation; 58% within 5° cup inclination and version; 1 periprosthetic fracture
Sigron et al. 2020. Journal of clinical medicine. PMID 32455967	cmf	hybrid orbital floor mesh	titanium	unclear	imaging+comp	clinical_comparative	22	22	mean absolute orbital-volume difference 1.0 (0.7) mL vs 1.6 (1.2) mL; surgery time 57.3 (23.4) min vs 99.8 (28.9) min; p = 0.001; postoperative stay 4.6 (3.9) d vs 3.8 (3.0) d; no significant difference
Rückschloß et al. 2020. Journal of cranio-maxillo-facial surgery : official publication of the European Association for Cranio-Maxillo-Facial Surgery. PMID 32505528	cmf	genioplasty patient-specific implant	titanium	am_metal	imaging+comp	clinical_series	29	29	left/right 0.25 ± 0.28 mm; anterior/posterior 0.70 ± 0.64 mm; up/down 0.45 ± 0.38 mm; rotations <2 deg
Boccalatte et al. 2020. Oral and maxillofacial surgery. PMID 32518971	cmf	temporomandibular joint prosthesis	multiple	unclear	imaging+comp	clinical_series	5	6	mean overall planned-to-postoperative prosthesis difference 2.21 mm (range 1.16-3.56 mm)
Lee et al. 2020. Plastic and reconstructive surgery. PMID 32590512	cmf	mandibular reconstruction plate	unclear	unclear	comp	clinical_comparative	126	126	total operative time 643.0 min vs 741.7 min; p = 0.001; hardware complications 5.5% vs 15.5%; p = 0.091; customized plates independently predicted fewer overall complications p = 0.03
Meynen et al. 2020. Computer methods in biomechanics and biomedical engineering. PMID 32619099	pelvis_acetabulum	acetabular custom implant	unclear	not_manufactured	imaging+comp	imaging_dataset	0	90	median hip joint centre error 3.54 mm two-sided vs 4.29 mm one-sided for pelvic discontinuities

Study	Region	Implant	Material	Manufacturing	Pillars	Tier	Patients	Analysed units	Headline result
Xu et al. 2020. Computer methods in biomechanics and biomedical engineering. PMID 32660275	other	joint prosthesis	unclear	unclear	imaging+comp	bench	0	.	relative density, surface topography and wear-resisting performance improved by GSO
Wang et al. 2020. Proceedings of the Institution of Mechanical Engineers. Part H, Journal of engineering in medicine. PMID 32723134	knee	customised total knee replacement patellar component	multiple	not_manufactured	comp	in_silico	0	4	bone-component contact at 15° flexion for small and 38° for medium implant; none for large
Katschnig et al. 2020. Polymers. PMID 32764496	cmf	maxillofacial defect implant	multiple	am_polymer	imaging	cadaver_phantom	0	2	bone-margin gaps approximately 1–2 mm; PETG print 27 and 30 min; PETG/TPU print 2 h 50 min and 2 h 59 min
Badiali et al. 2020. Scientific reports. PMID 32770002	cmf	mandibular osteosynthesis plate	titanium	am_metal	comp	clinical_comparative	22	44	median ramus total angular error 3.77° and translational error 1.41 mm; plate errors 3.14° and 1.08 mm; system successful in 22/22 patients; outcome clinically satisfactory in 22/22
Rahimov et al. 2020. Annals of maxillofacial surgery. PMID 32855907	cmf	customized orbital plate	titanium	unclear	imaging+comp	clinical_comparative	77	77	mean operation time 2.23/1.98/3.07 h virtual vs 3.47/2.05/3.31 h traditional by fracture group; residual diplopia 4/42 (9.5%) vs 12/35 (34.3%) at 6 mo
Burastero et al. 2020. Archives of orthopaedic and trauma surgery. PMID 33170352	knee	custom-made metaphyseal cone	titanium	unclear	comp	clinical_series	11	16	no mismatches between preoperative planning and intraoperative findings; custom-made cones produced more homogeneous bone stress; press-fit configurations increased fracture risk by more than 35%; no loosening or component migration
Freigang et al. 2020. BMC musculoskeletal disorders. PMID 33234122	knee	custom unicondylar knee arthroplasty	unclear	unclear	comp	clinical_series	29	31	target alignment achieved in 22/31 knees; malalignment rate 29%; preoperative planning and manufacturing approximately 6 wk; FJS 76.8 (SD 17.9); KSS knee +22.8 points; KSS function +34.8 points; VAS pain 5.4 to 1.1; no component loosening
Peng et al. 2020. BioMed research international. PMID 33335928	pelvis_acetabulum	hemipelvic tumour prosthesis	titanium	am_metal	imaging+comp	clinical_series	6	6	data processing 3 d; prosthesis fabrication and postprocessing 1 wk; VAS 7.167 to 2.83 (p=0.004); Harris hip score 32.33 to 58.17 (p=0.001); no loosening or infection
Liu et al. 2020. ACS biomaterials science & engineering. PMID 33449664	cmf	root analogue dental implant	titanium	am_metal	comp	bench	0	.	porous custom RAIs increased bone ingrowth and decreased bone resorption versus conventional implants and produced more

Study	Region	Implant	Material	Manufacturing	Pillars	Tier	Patients	Analysed units	Headline result
									uniform peri-implant stress under 100 N
Kormi et al. 2020. Journal of Craniofacial Surgery. PMID 33705044	cmf	orbital floor mesh	unclear	unclear	comp	clinical_series	8	8	mean 3D position error 1.3 to 1.8 mm (range 0.4 to 4.8 mm); postoperative orbital volume restored in 8/8 patients
Marta et al. 2020. Repositori digital de la UPF (Universitat Pompeu Fabra). General deformable 3D hip prosthesis model creation for patient-specific implant	hip	hip prosthesis	unclear	not_manufactured	imaging+comp	imaging_dataset	0	.	accurate shape registration; deformation required refinement to fit all anatomical parameters
Rijn et al. 2020. Research Repository (Delft University of Technology). Sacral anchoring of the LUMiC prosthesis: Development of a protocol to in vitro	pelvis_acetabulum	sacral anchoring tumour endoprosthesis	unclear	unclear	comp	bench	0	4	lower implant-bone micromotions than old LUMiC stem in FE model; 1 mm shell/20% infill bone model most comparable to FE model
Kilian et al. 2021. Bio-Design and Manufacturing. doi:10.1007/s42242-021-00153-4	knee	biphasic osteochondral implant	multiple	am_polymer	imaging+comp	cadaver_phantom	0	1	perfect geometric compatibility with SLA-fabricated defect model; implant printing ca. 30 min
Park et al. 2021. Journal of Computational Design and Engineering. doi:10.1093/jcde/qwab047	cmf	mandibular condyle osteosynthesis plate	unclear	not_manufactured	comp	in_silico	0	1	approximately 2× endurance capability against fractured bone separation versus conventional plates
Леонид et al. 2021. Russian journal of biomechanics. doi:10.15593/rjbiomech/2021.4.07	pelvis_acetabulum	custom acetabular component	unclear	not_manufactured	comp	in_silico	0	1	screws and endoprosthesis had sufficient safety margin; local bone micro-destruction expected around screw contacts
Yj et al. 2021. . doi:10.21203/rs.3.rs-141473/v1	pelvis_acetabulum	hemipelvic endoprosthesis	titanium	am_metal	comp	clinical_series	6	6	mean operative duration 333 min; MSTS 18.7 (range 13–27) at 12 wk; local recurrence 1/6; no aseptic loosening or implant breakage
György et al. 2021. . doi:10.21203/rs.3.rs-798715/v1	shoulder	total shoulder implant	multiple	not_manufactured	imaging+comp	in_silico	0	2	displacement ≤0.1 mm under 750 N compression; stress shielding quantified by SED difference
Cholkar et al. 2021. ESAFORM 2021. doi:10.25518/esaform21.756	cmf	patient-specific zygoma implant	multiple	not_manufactured	imaging+comp	in_silico	0	12	optimal Ti6Al4V design reduced mass 25%
Asmaria et al. 2021. Jurnal Penelitian Fisika dan Aplikasinya (JPFA). doi:10.26740/jpfa.v11n1.p83-93	pelvis_acetabulum	iliac fixation plate	titanium	not_manufactured	comp	in_silico	0	1	peak von Mises stress 3.616 MPa in Inventor and 664 MPa in ABAQUS under 307 N

Study	Region	Implant	Material	Manufacturing	Pillars	Tier	Patients	Analysed units	Headline result
Li et al. 2021. arXiv (Cornell University). doi:10.48550/arxiv.2108.05269	cranial	cranioplasty implant	unclear	not_manufactured	ai+imaging	imaging_dataset	0	.	.
Yang et al. 2021. Annals of surgical oncology. PMID 32572853	cmf	maxillary or mandibular reconstruction plate	titanium	am_metal	comp	clinical_comparative	33	33	absolute distance deviation 1.5 ± 0.5 mm vs 2.1 ± 0.7 mm; mean difference −0.7 mm, 95% CI −1.1 to −0.3; median operative time 510 min vs 496 min; plate removal 0/17 vs 2/16; no major postoperative adverse events in either group
Wang et al. 2021. Computer methods in biomechanics and biomedical engineering. PMID 32840135	knee	customised total knee implant	unclear	not_manufactured	comp	in_silico	0	.	transverse curvature influenced mediolateral translation; longitudinal curvature influenced femoral adduction
Olate et al. 2021. The Journal of craniofacial surgery. PMID 32858611	cmf	mandibular angle contour implant	peek	am_polymer	comp	clinical_comparative	21	21	greater facial symmetry than stock implants; P>0.05; surgical time 15 min shorter with PSI; P=0.021; infections in both groups; P>0.05
Wong et al. 2021. International journal of oral and maxillofacial surgery. PMID 32919821	cmf	maxillary fixation plate	titanium	unclear	comp	clinical_series	30	30	RMSE 0.38 mm transverse; 0.64 mm anteroposterior; 0.55 mm vertical; all surgical movements within 2 mm and 4° of planned
Blumer et al. 2021. Journal of oral and maxillofacial surgery : official journal of the American Association of Oral and Maxillofacial Surgeons. PMID 33010215	cmf	customized orbital titanium mesh	titanium	unclear	imaging+comp	clinical_series	34	34	long-term sequelae: diplopia 8.8%; mild enophthalmos 11.8%
Van Doremalen et al. 2021. Archives of orthopaedic and trauma surgery. PMID 33128609	upper_limb_other	contoured clavicle fixation plate	unclear	unclear	imaging+comp	clinical_comparative	14	14	plate fixation 15:52 vs 08:55; P = 0.032; discomfort-related implant removal 0/6 vs 5/7; P = 0.012; infection 1/7
Mian et al. 2021. Oral and maxillofacial surgery. PMID 33237435	cmf	custom TMJ prosthesis	unclear	unclear	comp	clinical_series	40	40	Lin concordance 0.97; 95% limits of agreement -5.9 to 5.4 mm; final position 0.4 mm anterior, 0.4 mm superior, and 2.4° posteriorly rotated
Karanxha et al. 2021. Journal of cranio-maxillo-facial surgery : official publication of the European Association for Cranio-Maxillo-Facial Surgery. PMID 33277160	cmf	maxillary and mandibular fixation plates	unclear	unclear	comp	clinical_comparative	16	16	A-point x-axis error 0.137 ± 0.067 mm vs 0.527 ± 0.387 mm; B-point x-axis error 0.697 ± 0.519 mm vs 1.728 ± 1.181 mm
Song et al. 2021. Journal of the mechanical behavior of biomedical materials. PMID 33310682	cmf	root analogue dental implant	titanium	unclear	comp	bench	0	8	yield strengths of all eight P and G configurations exceeded cortical bone; 30% and 40% porosity eliminated stress shielding in FEA

Study	Region	Implant	Material	Manufacturing	Pillars	Tier	Patients	Analysed units	Headline result
La Barbera et al. 2021. Clinical biomechanics (Bristol, Avon). PMID 33317937	spine	scoliosis rod-and-screw construct	unclear	not_manufactured	comp	in_silico	0	1080	3D coronal correction 67% versus 55%
Liu et al. 2021. Journal of biomaterials applications. PMID 33349103	spine	artificial lamina	peek	not_manufactured	comp	in_silico	0	4	ROM, intradiscal pressure, and annulus stresses decreased versus laminectomy across all motion patterns
Wu et al. 2021. World neurosurgery. PMID 33359737	cranial	cranioplasty plate	pmma	unclear	imaging+comp	clinical_series	14	14	implants were exact fits for the defects; cranial vault asymmetry index significantly improved; no intraoperative or postoperative complications in 14 patients
Dvoracek et al. 2021. Plastic and reconstructive surgery. PMID 33370061	cmf	orbital reconstruction plate	titanium	unclear	imaging	clinical_series	9	9	postoperative fracture-side orbital volumes were not significantly different from unfractured-side volumes; total model preparation approximately 10 h; plate bending approximately 60 s; enophthalmos resolved in 6/7 patients
De Santis et al. 2021. Materials (Basel, Switzerland). PMID 33401673	cranial	hybrid cranioplasty device	multiple	multiple	imaging+comp	bench	0	30	impact caused external cement-layer element failure without significantly altering underlying PCL structure stability
Schön et al. 2021. World neurosurgery. PMID 33418118	cranial	cranioplasty implant	pmma	cast	comp	clinical_series	16	16	postoperative CT scans showed well-fitting implants; median operation time 121 min (range 89–206); complications 7/16; reoperation 6/16
Zavattero et al. 2021. The Laryngoscope. PMID 33452834	cmf	mandibular reconstruction plate	unclear	unclear	comp	clinical_series	47	54	0.5-3 mm postoperative reconstruction accuracy
Maydanshahi et al. 2021. Biomedical materials (Bristol, England). PMID 33524959	upper_limb_other	radial head prosthesis	pmma	cast	imaging+comp	clinical_series	8	8	maximum von Mises stress 3.3 MPa under 200 N; intraoperative fabrication 16 min (1 min mixing; 15 min hardening); mean grip 32 kg vs 37 kg uninjured; MEPI 81/100; DASH 7/100; 1/8 secondary surgery; no infection or prosthesis removal
Huys et al. 2021. World neurosurgery. PMID 33529765	cranial	cranial implant	multiple	not_manufactured	comp	in_silico	0	3	PEEK showed high flexibility and local peak stress at the bone-screw interface; CeTi distributed stress evenly with limited deflection
Da Silva Júnior et al. 2021. 3D printing in medicine. PMID 33548008	cranial	cranioplasty implant	pmma	cast	imaging+comp	clinical_series	16	16	harmonious symmetry on preoperative and postoperative 3D CT; planning and mould production average 10 days; no infection, bleeding, or reoperation; 16/16 patients

Study	Region	Implant	Material	Manufacturing	Pillars	Tier	Patients	Analysed units	Headline result
									or relatives satisfied
Kozakiewicz et al. 2021. Materials (Basel, Switzerland). PMID 33578685	multi	custom craniofacial reconstruction implant	ceramic	machined	imaging+comp	clinical_series	14	14	soft tissue shift 3.79 ± 2.02 mm; implant position stable 14/14 for at least 6 y; no long-term clinical complications
Basu et al. 2021. Journal of clinical neuroscience : official journal of the Neurosurgical Society of Australasia. PMID 33581784	cranial	acrylic cranioplasty prosthesis	pmma	cast	comp	clinical_series	10	10	cranial index of symmetry 94.5 ± 3.9%; hardness 15.8 ± 0.24 Hv; tensile strength 30.7 ± 3.9 MPa; elastic modulus 1.5 ± 0.1 GPa; mean postoperative GOS 4.2 ± 0.9; acceptable cosmetic outcomes
IJpma et al. 2021. JAMA network open. PMID 33599774	pelvis_acetabulum	acetabular osteosynthesis plate	titanium	machined	imaging+comp	clinical_series	10	10	postoperative gap 3 (IQR 2-5) mm; step-off 0 (IQR 0-2) mm; screw-direction deviation 7.1° (IQR 7°-8°); plates and guides designed, fabricated, and sterilized within 4 days; native hip retained 10/10; deep wound infection 1/10
Brouwer de Koning et al. 2021. Scientific reports. PMID 33633247	cmf	mandibular reconstruction plate	unclear	unclear	imaging+comp	clinical_series	11	11	TRE 3.2 ± 1.1 mm at anatomical landmarks and 2.6 ± 1.5 mm at cutting-guide landmarks; navigation workflow added 32 min mean
Guoqing et al. 2021. Journal of the mechanical behavior of biomedical materials. PMID 33652236	lower_limb_other	bio-fixed tibial implant	unclear	am_metal	comp	bench	0	.	weight reduced; stress and displacement increased with more uniform distribution
Mu et al. 2021. International orthopaedics. PMID 33666766	lower_limb_other	total talar prosthesis	multiple	am_metal	imaging+comp	clinical_series	9	9	talar height 27.59 ± 5.99 mm preop vs 34.56 ± 3.54 mm postop; Meary's angle 11.73 ± 4.79° vs 4.45 ± 1.82°; AOFAS 26.33 ± 6.62 to 79.67 ± 3.14; VAS 6.33 ± 1.03 to 0.83 ± 0.75; postoperative complications 0/9
Neuhaus et al. 2021. International journal of oral and maxillofacial surgery. PMID 33707038	cmf	patient-specific total TMJ prosthesis	unclear	unclear	imaging+comp	clinical_comparative	21	26	mouth opening 21.69 ± 2.80 mm preop to 36.40 ± 1.25 mm; pain 6.18 ± 0.74 to 1.06 ± 0.52
Guo et al. 2021. Experimental and therapeutic medicine. PMID 33732321	cmf	TMJ condylar prosthesis	peek	am_polymer	comp	bench	0	5	maximum force 3814.7 ± 442.6 N Model A and 4245.7 ± 348.3 N Model B
Corona-Castuera et al. 2021. ACS omega. PMID 33748604	hip	partial hip prosthesis	unclear	am_metal	imaging+comp	bench	0	.	target-density lattice elastic modulus 0.25 GPa simple and 0.95 GPa double; compressive strength 14 MPa
Wang et al. 2021. Journal of orthopaedic surgery and	spine	vertebral body replacement	uncle	not_manufactu	comp	in_silico	0	2	rotational stiffness 32.8%-36.2% higher and posterior rod stress 24.8%-28.1%

Study	Region	Implant	Material	Manufacturing	Pillars	Tier	Patients	Analysed units	Headline result
research. PMID 33761991			ar	red					lower with artificial pedicle fixation
Sharma et al. 2021. Frontiers in physiology. PMID 33897455	cranial	cranioplasty plate	titanium	am_metal	imaging+comp	bench	0	2	overall RMSE 0.55 mm
Zhang et al. 2021. Bioactive materials. PMID 33898870	multi	large bone defect porous implant	titanium	am_metal	comp	clinical_series	26	26	6-month bending strength 2370 ± 141 N vs blank 2163 ± 156 N; implant-bone complex instability 0/26 at 28.44 ± 8.23 mo
Wong et al. 2021. Journal of orthopaedic translation. PMID 33981572	lower_limb_other	sinus tarsi implant	unclear	not_manufactured	comp	in_silico	0	1	sulcus tali and sinus tarsi stress 15.29 MPa at 75% stance; navicular height increased 4.2% at 25% stance
Kobayashi et al. 2021. Neurologia medico-chirurgica. PMID 34092749	cranial	cranioplasty plate	polymer_other	unclear	comp	clinical_series	18	18	UHMWPE Wing had the highest withstand load and lowest deformation; 17/18 had no postoperative adverse events for at least 18 mo; 1 screw loosening and graft uplift
Lang et al. 2021. PloS one. PMID 34101755	cmf	mandibular reconstruction osteosynthesis plate	titanium	am_metal	comp	cadaver_phantom	0	21	TOPOS implants 500000 cycles without plate failure vs miniplates 132000 ± 146000 cycles
Yang et al. 2021. Zhonghua wai ke za zhi [Chinese journal of surgery]. PMID 34102730	lower_limb_other	total talus prosthesis	multiple	multiple	imaging+comp	clinical_series	8	8	prosthesis measurement completely consistent with contralateral healthy talus; AOFAS 26.3 ± 6.6 preoperative to 79.3 ± 4.2 at 12 mo; no revision
Knitschke et al. 2021. Cancers. PMID 34208555	cmf	custom osteosynthesis plate	titanium	am_metal	comp	clinical_comparative	104	104	median time to therapy initiation 42 d with VSP vs 31.0 d with non-VSP; operating time 508.2 ± 88.8 min vs 562.5 ± 98.5 min; bone R0 margins 95.1% VSP vs 96.8% non-VSP (p = 0.516); soft-tissue R0 margins 63.4% vs 73.0% (p = 0.470)
Shah et al. 2021. The Journal of craniofacial surgery. PMID 34224462	multi	intracranial and orbital reconstruction implant	unclear	unclear	comp	clinical_series	6	6	planned-vs-actual orbital volume discrepancy 1.5% ± 1.6%; postoperative orbital volume within 1.3% ± 2.0% of unaffected orbit; temporal implant accuracy 81.0%
Kang et al. 2021. Materials science & engineering. C, Materials for biological applications. PMID 34225889	spine	multi-segment artificial vertebral implant	titanium	am_metal	comp	bench	.	2	optimized implant maximum Mises stress was about 41.5% of trussed implant; maximum compression load and compression stiffness per unit mass were about 2 times trussed implant; good short-term clinical outcomes

Study	Region	Implant	Material	Manufacturing	Pillars	Tier	Patients	Analysed units	Headline result
Rana et al. 2021. Proceedings of the Institution of Mechanical Engineers. Part H, Journal of engineering in medicine. PMID 34227419	multi	bone graft scaffold	multiple	not_manufactured	comp	in_silico	0	6	maximum von Mises stress difference vs natural bone -6% to 10.7% for porous implants and 26% to 50% for solid implants; average strain difference -7% to -17.9% and -36% to -59%, respectively
Pinheiro et al. 2021. Scientific reports. PMID 34234245	cmf	TMJ replacement	multiple	not_manufactured	comp	in_silico	0	1	excessive mediolateral and anteroposterior implant displacement vs intact joint in INC, LML, and RML biting; peri-screw principal strains about -8400 με and 9530 με
Järvinen et al. 2021. The Journal of craniofacial surgery. PMID 34260465	cmf	orthognathic fixation implant	unclear	unclear	imaging+comp	clinical_series	10	10	all 10 patient-specific implants fitted precisely onto the mandible and screws entered the predrilled holes; predetermined occlusion obtained in 10/10 patients
Li et al. 2021. Zhonghua kou qiang yi xue za zhi = Zhonghua kouqiang yixue zazhi = Chinese journal of stomatology. PMID 34275217	cmf	zygomatico-orbital and maxillary prosthesis	unclear	unclear	comp	clinical_series	5	5	deviation 2-3 mm at fixed structure during clinical evaluation and about 1 mm after prosthesis scanning; satisfactory reconstruction contour in 5/5 patients
Carnicero et al. 2021. Scientific reports. PMID 34321582	cmf	subperiosteal jaw implant	titanium	not_manufactured	comp	in_silico	1	1	Opt2 maximum von Mises stress 199.51 MPa under standard bite loading and 395.03 MPa under critical bite loading; volume reduced 27.68%
Li et al. 2021. Medical image analysis. PMID 34340106	cranial	cranioplasty implant	unclear	not_manufactured	ai+comp	imaging_dataset	0	167	.
Wang et al. 2021. World journal of surgical oncology. PMID 34365976	knee	tumour endoprosthesis	titanium	am_metal	comp	clinical_series	15	15	design, fabrication, and delivery about 10 d; all 15 implants osseointegrated; early aseptic loosening 1/15; intraoperative periprosthetic fracture 2/15; median MSTS-93 26 (range 23-30)
Liu et al. 2021. IEEE robotics and automation letters. PMID 34395869	cranial	cranioplasty plate	unclear	am_polymer	imaging+comp	cadaver_phantom	0	4	bone gap distance reduced >60% on plastic skulls and >30% on cadavers versus manual resizing; automated resizing 10–15 min vs manual 10–30 min
Ren et al. 2021. Zhongguo gu shang = China journal of orthopaedics and traumatology. PMID 34423622	spine	interbody fusion cage	unclear	not_manufactured	comp	in_silico	0	3	standard cage model extension stress 45.81 MPa in vertebral body and 97.07 MPa in cage; individualized model displacement generally lower than standard model
Sigron et al. 2021. Journal of clinical medicine. PMID 34441806	cmf	hybrid patient-specific orbital floor mesh	titanium	unclear	imaging+comp	clinical_comparative	30	30	surgery duration 58.9 (SD 20.1) min vs 94.8 (SD 33.0) min; difference 35.9 min; P = 0.003; complete diplopia

Study	Region	Implant	Material	Manufacturing	Pillars	Tier	Patients	Analysed units	Headline result
									resolution 9/12 vs 5/7; no major complications
Sharma et al. 2021. Journal of clinical medicine. PMID 34441859	cmf	orbital mesh implant	peek	am_polymer	imaging+comp	bench	0	15	morphological fit good 3/15, satisfactory 8/15, poor 4/15; von Mises stress 1.519–5.31 MPa; deformation 0.026–0.107 mm under 0.3 N
Meesters et al. 2021. Journal of personalized medicine. PMID 34442407	pelvis_acetabulum	pre-contoured acetabular fixation plate	unclear	unclear	imaging+comp	cadaver_phantom	0	111	median screw-direction difference 6.1° (IQR 4–8°); median entry-point difference 3.4 mm (IQR 2–4 mm); CT-to-guide production time 6 d
Sharma et al. 2021. International journal of molecular sciences. PMID 34445228	cranial	cranioplasty plate	peek	am_polymer	imaging+comp	bench	0	10	dimensional RMSE 0.731 ± 0.013 mm; reconstruction conformance RMSE 0.499 ± 0.032 mm; mean peak load 798.38 ± 211.45 N at displacement 2.54 ± 0.56 mm
Mattavelli et al. 2021. World neurosurgery. PMID 34450324	cranial	clival reconstruction scaffold	polymer_other	am_polymer	imaging+comp	cadaver_phantom	0	11	maximal positioning accuracy with real-defect scaffold; satisfactory accuracy with predicted clivectomy; CSF leak pressure significantly higher with screw fixation than gasket technique; production process approximately 30 h
Lin et al. 2021. BMC oral health. PMID 34521397	cmf	root-analogue dental implant	titanium	not_manufactured	comp	in_silico	0	6	maximum von Mises stress <25 MPa in cortical bone and <6 MPa in cancellous bone; threaded implants had lower stress than smooth implants
Chepurnyi et al. 2021. Annals of maxillofacial surgery. PMID 34522661	cmf	zygomatic complex patient-specific implant	unclear	unclear	imaging+comp	clinical_series	11	11	planned-to-actual PSI deviation 0.72 ± 0.41 mm; reconstructed-to-mirrored-side deviation 1.45 ± 0.7 mm; orbital volume difference 1.7 ± 0.8 mm3; fistula 1/11 requiring sequestrectomy with implant preservation; no implant exposure or maxillary sinusitis
Kang et al. 2021. Frontiers in oncology. PMID 34540688	cmf	pre-bent midface reconstruction plate	titanium	unclear	imaging+comp	clinical_comparative	34	34	BCR 59.4% ± 27.9% vs 37.0% ± 35.5%; DAR 87.5% ± 18.9% vs 64.9% ± 23.4%; dental implantation 50.0% vs 11.1%; all patients satisfied with facial symmetry
Zhong et al. 2021. Journal of the mechanical behavior of biomedical materials. PMID 34563812	cmf	mandibular reconstruction plate	unclear	not_manufactured	comp	in_silico	0	2	locking plate had lower maximum stress, strain, and displacement than non-locking plate
Montemurro et al. 2021. International journal of environmental research and	cranial	custom-made cranial bone flap	polyme	am_polymer	imaging+com	cadaver_phantom	0	5	task execution error <±1 mm; setup <1 min

Study	Region	Implant	Material	Manufacturing	Pillars	Tier	Patients	Analysed units	Headline result
public health. PMID 34639256			r_other		p				
Kargarnejad et al. 2021. Computer methods in biomechanics and biomedical engineering. PMID 34657530	cmf	mandibular reconstruction plate	unclear	not_manufactured	comp	in_silico	0	3	cortical bone stress 110 MPa; conventional plate stress 580 MPa; fibula model plate reliability 99.99% and screw pull-out reliability 96.71%; customized prosthesis 99.99% and 94.17%
Antúnez-Conde et al. 2021. Frontiers in oncology. PMID 34676161	cmf	mandibular reconstruction plate and mesh	titanium	unclear	comp	clinical_series	14	14	dynamic-navigation position deviation <1 mm and angular deviation <5°; 60 dental implants; osseointegration rate 91.49%; normal swallowing 12/14
Shaikh et al. 2021. Dentistry journal. PMID 34677171	cmf	maxillofacial framework implant	titanium	am_metal	imaging+comp	bench	0	3	green-part shrinkage 1.37% experiment vs 1.96% simulation; maximum residual stress 2.6–3.1 MPa; relative density 81 ± 4%; hardness 6.5 ± 0.8 HRC
Balato et al. 2021. Journal of personalized medicine. PMID 34683181	knee	antibiotic-loaded knee spacer	pmma	not_manufactured	comp	imaging_dataset	0	19	.
Li et al. 2021. Plastic and reconstructive surgery. PMID 34705785	cmf	maxillary osteosynthesis plate	titanium	am_metal	comp	rct	58	58	maxilla position discrepancy 1.41 ± 0.58 mm vs 2.20 ± 0.94 mm (P < 0.001); infection 1/27 vs 1/31; no significant difference in serious adverse events
Shi et al. 2021. Computers in biology and medicine. PMID 34741907	cmf	mandibular reconstruction plate	titanium	am_metal	comp	in_silico	0	4	maximum plate tensile stress reduced 6.3%; relative total displacement reduced 41.4%; bone segment dislocation below 42.1 µm
Yoon et al. 2021. Korean journal of neurotrauma. PMID 34760819	cranial	titanium mesh cranioplasty implant	titanium	am_metal	imaging+comp	clinical_series	40	40	mean DSC 0.75; operation time 152.5 ± 51.7 min; no complications requiring reoperation; 30/40 reported very satisfactory cosmetic outcomes at 6 mo
Maslov et al. 2021. Materials (Basel, Switzerland). PMID 34832464	pelvis_acetabulum	custom acetabular endoprosthesis	titanium	am_metal	comp	in_silico	0	1	optimal screw pretension 500–1000 N; highest screw stress 263 MPa
Badiali et al. 2021. Journal of personalized medicine. PMID 34834588	cmf	orthognathic fixation plate	titanium	am_metal	comp	clinical_series	22	22	mandibular median total angular error 2.34° and total translational error 2.01 mm; plate infections 1/22 (4.5%)
Möllmann et al. 2021. Frontiers in oncology. PMID 34900674	cmf	mandibular reconstruction plate	unclear	unclear	comp	clinical_comparative	94	94	D6 mean postoperative difference -1.04 mm PSI vs 1.52 mm CR; operative time 467 min PSI vs 397 min CR; complications 33/46 PSI vs

Study	Region	Implant	Material	Manufacturing	Pillars	Tier	Patients	Analysed units	Headline result
									31/48 CR; no significant difference
Wu et al. 2021. Biosensors. PMID 35049632	lower_limb_other	distal femoral defect scaffold	titanium	am_metal	comp	bench	0	6	69.6% lower implant weight; 12% lower displacement, 34% lower bone-plate von Mises stress, 33% lower bone first-principal stress and 32% lower bone first-principal strain than bone cement
Pu et al. 2021. Frontiers in oncology. PMID 35059310	pelvis_acetabulum	sacroiliac reconstruction prosthesis	titanium	am_metal	comp	clinical_comparative	24	24	operation time 120.30 ± 14.50 min vs 165.25 ± 15.00 min; MSTS-93 24.1 ± 2.8 vs 18.9 ± 2.6; complications 1/12 vs 1/12; disease-free survival 9/12 vs 9/12
MacLeod et al. 2021. Communications medicine. PMID 36737534	knee	high tibial osteotomy plate	titanium	am_metal	comp	in_silico	0	28	12-week fatigue-failure odds ratio 0.14 (95% CI 0.01 to 2.73)
Kanagalingam et al. 2022. Progress in Additive Manufacturing. doi:10.1007/s40964-022-00342-2	knee	high tibial osteotomy fixation plate	titanium	multiple	ai+comp	bench	0	2	mean surface deviation −0.10 mm for plate A and −0.11 mm for plate B; build time 10 h 8 min; machining cycle 1 h 37 min
Helguero et al. 2022. Procedia CIRP. doi:10.1016/j.procir.2022.06.065	other	trabecular biomimetic bone implant	unclear	unclear	comp	in_silico	0	.	compression modulus increased by 49.74% for trabecular vs compact structures
Jindal et al. 2022. Proceedings of the Institution of Mechanical Engineers Part L Journal of Materials Design and Applications. doi:10.1177/14644207221104875	cranial	cranioplasty plate	multiple	not_manufactured	comp	in_silico	0	.	PEEK implant von Mises stress 13.5–35% lower than autologous bone; square X fixtures reduced stress 18.4% with titanium and 10.9% with PEEK versus linear fixtures under 1780 N and 15 mmHg
Xiang et al. 2022. . doi:10.21203/rs.3.rs-1599305/v1	knee	patient-specific tibial plateau locking plate	titanium	not_manufactured	comp	in_silico	0	4	group D deformation index reduced 3.23%; stress index increased 58.89% vs group A
Karlagina et al. 2022. . doi:10.21203/rs.3.rs-2252495/v1	cmf	titanium barrier membrane	titanium	machined	ai+imaging	bench	0	7	CT processing 1 min vs 4.5–6.5 h manual
Tiwari et al. 2022. Applied Sciences. doi:10.3390/app12063003	cmf	total TMJ prosthesis	multiple	not_manufactured	comp	in_silico	0	1	natural TMJ deformation exceeded patient-specific implant deformation for Ti-6Al-4V and Co-Cr models
Al-Tamimi et al. 2022. Applied Sciences. doi:10.3390/app122010569	lower_limb_other	distal tibia fixation plate	titanium	not_manufactured	comp	in_silico	0	4	higher retained volume increased load-transfer reduction in the fractured area and reduced stress-shielding risk
Agarwal et al. 2022. European journal of trauma	pelvis	acetabular reconstruction plate	uncle	unclear	imaging+	clinical_series	30	30	virtual planning time 15.67 (SD 6.12) min contralateral

Study	Region	Implant	Material	Manufacturing	Pillars	Tier	Patients	Analysed units	Headline result
and emergency surgery : official publication of the European Trauma Society. PMID 33683380	_acetabulum		ar		comp				vs 81.83 (SD 28.02) min fractured side
Harding et al. 2022. International journal of oral and maxillofacial surgery. PMID 33941394	cmf	custom mandibular osteosynthesis plate	unclear	unclear	comp	clinical_comparative	55	55	root mean square error within 2 mm and 4° in both groups
De Moor et al. 2022. International journal of oral and maxillofacial surgery. PMID 34059405	cmf	subperiosteal jaw implant	titanium	am_metal	comp	in_silico	0	2	average peak von Mises stress 178.8 MPa under 200 N occlusion
Schader et al. 2022. Journal of shoulder and elbow surgery. PMID 34298147	shoulder	proximal humerus locking plate	unclear	not_manufactured	comp	in_silico	0	19	peri-screw bone strain reduced 6.8% ± 4.0% vs cohort-specific orientations and 25.24% ± 7.93% vs original PHILOS orientations
Atef et al. 2022. Oral and maxillofacial surgery. PMID 34328575	cmf	mandibular onlay implant	peek	unclear	comp	clinical_series	6	7	soft-tissue gain 6.8 ± 0.98 mm chin vs 4.42 ± 0.41 mm angle; P=0.0001
Gass et al. 2022. Journal of anatomy. PMID 34658032	cmf	orbital floor implant	unclear	not_manufactured	imaging+comp	imaging_dataset	0	19	median error 0.26 mm small and 0.34 mm large with SSM vs 0.70 mm and 0.73 mm with mirroring
Vancleef et al. 2022. Journal of orthopaedic research : official publication of the Orthopaedic Research Society. PMID 34668224	upper_limb_other	clavicle fracture fixation plate	titanium	unclear	comp	bench	0	1	mean plate-bone distance 0.47 mm PSP vs 0.63 mm commercial; peak von Mises 1080 MPa PSP vs 1870 MPa commercial with 5 mm gap; maximum interfragmentary motion 1.039 mm vs 2.317 mm
Pöppe et al. 2022. Acta neurochirurgica. PMID 34873659	cranial	cranioplasty implant	pmma	cast	imaging+comp	clinical_series	14	16	all 16 implants fitted well; postoperative CT showed excellent reconstruction; 3D-print preparation 30 min; printing 3–8 h; revision for hematoma 3/14; no mortality or new permanent neurological deficits
Alqussair et al. 2022. The Journal of craniofacial surgery. PMID 34907951	cmf	custom maxillary fixation plate	titanium	unclear	comp	clinical_series	10	10	mean absolute deviation 0.98 mm sagittal, 0.67 mm vertical, and 0.62 mm transverse
Timoshchuk et al. 2022. Journal of oral and maxillofacial surgery : official journal of the American Association of Oral and Maxillofacial Surgeons. PMID 35007501	cmf	orbital reconstruction implant	titanium	unclear	comp	clinical_comparative	85	85	orbital volume difference 4.2% vs 6.8% (P = .03); complications 8.3% vs 26.2% (P = .08)
Lin et al. 2022. European journal of oral sciences. PMID 35014088	cmf	root-analogue dental implant	unclear	not_manufactured	comp	in_silico	0	5	maximum implant/bone stress 206/111 MPa with 3 mm reduction; bone stress up to 65.1 MPa with 0-2 mm reduction vs 130.4 MPa for traditional implant
Vanaclocha-Saiz et al. 2022.	spi	lumbar disc nucleus	po	not_ma	com	in_silic	0	6	maximum implant

Study	Region	Implant	Material	Manufacturing	Pillars	Tier	Patients	Analysed units	Headline result
ACS applied bio materials. PMID 35014829	ne	prosthesis	lymer_other	nufactured	p	o			compression stress 16-34 MPa and tensile stress 5-16 MPa under 1000 N axial compression with or without 8 Nm flexion and 8 Nm axial rotation
Dot et al. 2022. European radiology. PMID 35037088	cmf	orthognathic fixation implant	unclear	unclear	ai+imaging	imaging_dataset	0	163	test vDSC/sDSC: upper skull 96.22%/96.92%, mandible 94.19%/97.92%, upper teeth 94.83%/98.87%, lower teeth 94.38%/98.53%, mandibular canal 81.59%/97.90%; automatic segmentation approximately 10 min per CT scan
Solórzano-Requejo et al. 2022. Materials (Basel, Switzerland). PMID 35057160	hip	short femoral stem	titanium	am_polymer	comp	bench	0	2	R1-R3 implantable in both geometric cases; R4 not implantable; V3 Ti21S selected for both geometric cases based on strain shielding and adjusted R^2
Shen et al. 2022. Materials (Basel, Switzerland). PMID 35057294	cmf	mandibular segmental defect implant	titanium	am_metal	comp	bench	0	5	peak implant stress 299 MPa under RMOL and 332 MPa under RGF for 1 mm hexagonal pores; all values below Ti6Al4V yield strength 800 MPa
Guo et al. 2022. International orthopaedics. PMID 35061064	shoulder	scapular fixation plate	unclear	unclear	comp	clinical_comparative	21	21	preoperative virtual simulation 44.42 ± 15.54 min; preoperative plate contouring 16.08 ± 5.09 min; operation 28.75 min shorter; no complications in 21/21; follow-up functional outcome similar between groups
Baldia et al. 2022. Acta neurochirurgica. PMID 35107617	cranial	cranioplasty prosthesis	pmma	am_polymer	imaging	clinical_comparative	70	70	CT anthropometric asymmetry correlated with cosmetic outcome, p=0.01; 3D-printed prostheses had better cosmetic scores than hand-moulded and bone-impression prostheses, p<0.001
Arafat et al. 2022. The Journal of craniofacial surgery. PMID 35119415	cranial	cranioplasty plate	polymer_other	am_polymer	comp	clinical_series	10	10	preoperative-to-postoperative mean difference 0.3197 ± 0.1649 (unit not reported); Glasgow Outcome Score 1 in 10/10; patient satisfaction 10 ± 0; surgeon satisfaction 9 ± 1; cranial symmetry index 98% ± 1%
Von Atzigen et al. 2022. Medical image analysis. PMID 35139481	spine	spinal fusion rod	unclear	unclear	ai+comp	cadaver_phantom	0	.	average bending time 231 s with 0.6 rebending maneuvers per rod vs 476 s with 3.5 freehand and 348 s with 1.1 marker-based
Jones et al. 2022. Journal of oral and maxillofacial surgery : official journal of the American Association of	cmf	maxillary osteosynthesis plate	unclear	unclear	comp	clinical_comparative	82	82	mean planned-to-actual maxillary discrepancy smaller with PSI than interim splint

Study	Region	Implant	Material	Manufacturing	Pillars	Tier	Patients	Analysed units	Headline result
Oral and Maxillofacial Surgeons. PMID 35151639									
Lv et al. 2022. Journal of plastic, reconstructive & aesthetic surgery : JPRAS. PMID 35183467	cmf	orbital-maxillary-zygomatic implant	peek	unclear	comp	clinical_series	12	12	postoperative exophthalmos 15.91 ± 1.80 mm; vertical eyeball-position difference 15.91 ± 1.80 mm; orbital volume 15.91 ± 1.80 mm; no obvious complications
Kittichokechai et al. 2022. Plastic and reconstructive surgery. Global open. PMID 35186634	cmf	orbital mesh	titanium	am_metal	imaging+comp	clinical_series	16	16	mean between-eye orbital-volume difference reduced 2904.40 mm3; exophthalmometry difference reduced 2.89 mm; infected wound requiring implant removal 1/16; unresolved vertical diplopia 1/16
Lin et al. 2022. International journal of bioprinting. PMID 35187275	cmf	mandibular continuity-defect reconstruction implant	titanium	am_metal	imaging+comp	bench	0	30	all AM implants passed 250,000 cycles; bone plates had condyle fracture, plate fracture, or screw loosening
Jeong et al. 2022. Polymers. PMID 35215652	cmf	zygomatico-maxillary scaffold	multiple	am_polymer	imaging+comp	clinical_series	8	8	mean volume conformity 79.71% (range 70.89%-86.31%); Young’s modulus 162.7 ± 12.8 MPa; wound dehiscence 1/8; no bony instability or occlusal complication
Liu et al. 2022. Medical & biological engineering & computing. PMID 35237925	lower_limb_other	total talus implant	cocr	not_manufactured	comp	in_silico	0	4	customized-scale models had larger total contact area than SSM-scale and universal-scale models
Dowgierd et al. 2022. Oral oncology. PMID 35255279	cmf	mandibular reconstruction implant	unclear	unclear	comp	clinical_series	21	21	average maximum deviation 7.7 mm; average minimum deviation -6.09 mm
Park et al. 2022. The Journal of craniofacial surgery. PMID 35261367	cranial	PCL/beta-TCP cranioplasty implant	multiple	am_polymer	imaging+comp	clinical_series	7	7	complete cranial symmetry 6/7; smooth edge interface 6/7; seroma 1/7; no implant fracture, mobilization, or dislocation at 6 mo
Hu et al. 2022. Journal of orthopaedic surgery and research. PMID 35264178	knee	strut-type bone-defect prosthesis	titanium	am_metal	comp	clinical_comparative	10	10	all 5 prosthesis patients had no interfacial gap at 2 y; peak implant displacement 0.07 mm versus 0.81 mm for cement-plate-screw system; MSTS 28.4 ± 1.8 versus 26.2 ± 2.8, P = 0.176; all 5 prosthesis patients achieved bone graft fusion
Msallem et al. 2022. Materials (Basel, Switzerland). PMID 35269201	cranial	cranioplasty plate	pmma	cast	comp	bench	0	10	mean fracture force 1484.6 ± 167.7 N
Cheng et al. 2022. Technology and health care : official journal of the European Society for Engineering and Medicine.	cmf	condylar prosthesis	titanium	am_metal	comp	in_silico	0	2	maximum stresses 6.6%, 36.4%, and 47.8% lower for porous versus solid model under LCI, LRM, and LBM

Study	Region	Implant	Material	Manufacturing	Pillars	Tier	Patients	Analysed units	Headline result
PMID 35275582									loading
Wu et al. 2022. Journal of biomedical materials research. Part B, Applied biomaterials. PMID 35297154	lower_limb_other	femoral shaft prosthesis	peek	am_polymer	comp	bench	1	2	prosthesis and femoral osteotomy end closely matched; maximum loading force and yield strength increased 3% and 6%; stress concentration reduced 5.4%–10.9%; no early complications
Lizio et al. 2022. Clinical oral implants research. PMID 35305283	cmf	customized guided-bone-regeneration mesh	titanium	am_metal	imaging+comp	clinical_series	17	19	mean planned-to-actual mesh fitting 82 ± 13.4%; mesh exposures 10/19; infection 3/19; failures 5/19; implant survival 97.7%
Mian et al. 2022. Polymers. PMID 35335596	cranial	cranioplasty implant	peek	am_polymer	imaging+comp	bench	0	1	overall deviation 0.5919 mm; gaps 0.91 mm X and 0.71 mm Y; maximum von Mises stress 8.15 MPa; strain 0.002; deformation 0.18 mm under 50 N at each of 3 regions; implant modeling around 1 h; FEA 450 s; support removal 30–40 min
Chen et al. 2022. BioMed research international. PMID 35342763	cmf	mandibular prosthesis	titanium	am_metal	imaging+comp	bench	0	5	maximum static load 189 N; fatigue 5,000,000 cycles at 10–100 N; animal-model P4 von Mises stress 508.53 MPa
Moellmann et al. 2022. Journal of clinical medicine. PMID 35407653	cranial	cranioplasty implant	peek	unclear	imaging+comp	clinical_series	39	39	95.17% of measurements within ±1.5 mm tolerance; postoperative complications 25/39; explantation 4/39
Liokatis et al. 2022. Laryngoscope investigative otolaryngology. PMID 35434332	cmf	midfacial osteosynthesis implant	titanium	am_metal	imaging+comp	clinical_comparative	8	18	median PSI deviation 2.01 mm; bone-segment deviation 2.77 mm PSI vs 3.28 mm prebent plates; no major complications; plate removal 2/8
Steiert et al. 2022. Neurosurgical review. PMID 35441994	cmf	craniofacial PMMA implant	pmma	cast	imaging+comp	cadaver_phantom	0	36	median total error volume 6.40 cm3 AR vs 13.48 cm3 freehand; registration 5–10 min; fabrication 10–20 min
Li et al. 2022. ACS biomaterials science & engineering. PMID 35443132	cmf	alveolar augmentation scaffold	multiple	multiple	comp	bench	0	.	PEEK bending strength about 1/3 titanium; both scaffold deformations <0.05 mm
Ali et al. 2022. Maxillofacial plastic and reconstructive surgery. PMID 35524015	cranial	temporal fossa implant	peek	machined	imaging+comp	clinical_series	8	8	seroma 1/8; patient satisfaction 4–5/5; surgeon grade I in 8/8
Van Kootwijk et al. 2022. Journal of the mechanical behavior of biomedical materials. PMID 35660552	cmf	mandibular reconstruction implant	titanium	am_metal	comp	bench	0	2	FE principal strains agreed with DIC measurements; no failures under >1000 N; no significant differences in stiffness, ultimate load, or ultimate displacement; LA-implant design time 633.3% shorter
Liu et al. 2022. Frontiers in bioengineering and	mult	customized metaphyseal bone-	titani	am_metal	imaging+	clinical_series	16	16	maximum von Mises stress 184.7 MPa in IM nail;

Study	Region	Implant	Material	Manufacturing	Pillars	Tier	Patients	Analysed units	Headline result
biotechnology. PMID 35721863	i	defect prosthesis	um		comp				proximal/distal femur 76.1/38.8 MPa in Mode I; no obvious loosening, subsidence, or breakage in 16/16; continuous bone regeneration in Mode II
Korkmaz et al. 2022. Biomedizinische Technik. Biomedical engineering. PMID 35727116	knee	total knee prosthesis	unclear	unclear	comp	bench	0	2	femoral component/cement von Mises stress 103.10/21.67 MPa anatomical vs 175.86/25.76 MPa conventional; cement deformation 15% lower
Rodríguez-Arias et al. 2022. Journal of personalized medicine. PMID 35743715	cmf	mandibular reconstruction plate	unclear	unclear	comp	clinical_comparative	37	37	average operating-room time gain 102 min; 5% fewer complications with CAD/CAM; flap survival 100%
Ingawale et al. 2022. Materials (Basel, Switzerland). PMID 35744401	cmf	total TMJ prosthesis	multiple	not_manufactured	comp	in_silico	0	2	peak von Mises stress 108.3 MPa in condylar screws under 400 N overload
Sánchez-Jáuregui et al. 2022. Journal of cranio-maxillo-facial surgery : official publication of the European Association for Cranio-Maxillo-Facial Surgery. PMID 35760659	cmf	maxillary osteosynthesis plate	unclear	unclear	comp	rct	30	30	mean error 0.8 mm anteroposterior, 0.4 mm vertical, 0.2 mm horizontal with customized devices; operative time reduced by mean 36.5 min
Ashraf et al. 2022. Surgical neurology international. PMID 35855120	cranial	cranioplasty implant	pmma	cast	imaging+comp	clinical_series	10	10	radiological outcome excellent (aligned within 1 mm) in 8/10; 1 implant inaccurate; PLA print time 4–8 h; no postoperative complications; revision planned for 1/10; cosmetic score 10/10 in 8/10
Fernandes et al. 2022. Scientific reports. PMID 35869276	spine	interbody cage	polymer_other	am_polymer	imaging+comp	cadaver_phantom	0	18	contact area 100 ± 23.6 mm2 vs 57.5 ± 13.7 mm2; 104.8 ± 39.6 mm2 vs 55.2 ± 35.1 mm2; contact stress 1.06 vs 1.84 MPa and 1.10 vs 2.44 MPa under 100 N
Zhong et al. 2022. Computer methods and programs in biomedicine. PMID 35872386	cmf	mandibular reconstruction plate	unclear	not_manufactured	comp	in_silico	0	3	peak von Mises stress 264 MPa and 296 MPa vs 393 MPa during left group clenching
Chepurnyi et al. 2022. Journal of cranio-maxillo-facial surgery : official publication of the European Association for Cranio-Maxillo-Facial Surgery. PMID 35963834	cmf	orbital wall patient-specific implant	unclear	unclear	imaging+comp	clinical_comparative	50	50	mean orbital volume difference 0.65 ± 0.26 cm3 vs 0.57 ± 0.23 cm3; shape difference -3.3 ± 3.5% vs 3.25 ± 2.5%; CAD time 36.7 ± 6.9 min vs 72.9 ± 7.7 min; no implant malposition or enophthalmos; diplopia 13.0% vs 11.1% at 3 mo
Woo et al. 2022. The Journal of craniofacial surgery. PMID 36041093	cmf	customized orthognathic fixation plate	unclear	machined	imaging+comp	clinical_series	16	32	mean virtual FRI correction 3.0° vs actual 2.9°; achievement 102.8%
Zhao et al. 2022. Medical engineering & physics.	spi	interspinous spacer	mult	not_manufactu	com	in_silic	0	3	PEEK CISP had lower maximum implant stress

Study	Region	Implant	Material	Manufacturing	Pillars	Tier	Patients	Analysed units	Headline result
PMID 36068031	ne		iple	red	p	o			than titanium CISP and Coflex
Hu et al. 2022. BMC musculoskeletal disorders. PMID 36076197	lower_limb_other	customized intercalary femoral prosthesis	titanium	am_metal	comp	clinical_series	12	12	stem tip displacement 0.63 ± 0.8 mm Stem A, 2.0 ± 1.4 mm Stem B, and 1.0 ± 0.8 mm Stem C; peak stem von Mises stress 35.49–107.38 MPa across models; MSTS 25.5 ± 1.3 vs 18.67 ± 0.9; VAS 0.5 ± 0.9 vs 5.4 ± 1.0; aseptic loosening 1/12
Pontiki et al. 2022. Annual International Conference of the IEEE Engineering in Medicine and Biology Society. IEEE Engineering in Medicine and Biology Society. Annual International Conference. PMID 36085707	chest_wall	rib prosthesis	pmma	multiple	comp	cadaver_phantom	0	288	mean mesh error 1.37 ± 0.66 mm right and 1.68 ± 0.77 mm left vs segmented ribs; model generation time 0.027 ± 0.009 min vs segmentation 11.56 ± 1.60 min
Sulakhe et al. 2022. Annual International Conference of the IEEE Engineering in Medicine and Biology Society. IEEE Engineering in Medicine and Biology Society. Annual International Conference. PMID 36085744	cranial	cranioplasty implant	unclear	not_manufactured	ai+comp	in_silico	0	.	.
Liu et al. 2022. Frontiers in surgery. PMID 36090321	lower_limb_other	diaphyseal defect prosthesis	titanium	not_manufactured	comp	in_silico	0	5	peak stresses 47.1 MPa tibia, 51.8 MPa prosthesis, 87.2 MPa screws, and 101.8 MPa IM nail under 700 N
Kang et al. 2022. Journal of orthopaedics. PMID 36090783	pelvis_acetabulum	customized acetabular cup	titanium	am_metal	comp	bench	0	6	peak von Mises 136.99 MPa; 6/6 specimens unbroken at 5340.74–5345.13 N
Abel et al. 2022. Journal of oral and maxillofacial surgery : official journal of the American Association of Oral and Maxillofacial Surgeons. PMID 36116544	cmf	maxillary and mandibular fixation plates	unclear	unclear	comp	clinical_series	49	49	A-point absolute discrepancies 0.57 mm horizontal, 0.37 mm transverse, 0.45 mm vertical; B-point 1.15, 0.62, 1.14 mm; pogonion 1.29, 0.85, 1.24 mm
Cassoni et al. 2022. International journal of environmental research and public health. PMID 36142107	cmf	maxillary fixation plate	titanium	am_metal	imaging+comp	clinical_comparative	10	10	3D error 1.22 mm (SD 0.46) vs 1.63 mm (SD 0.3); planning time 60–120 min
Tran et al. 2022. Journal of clinical medicine. PMID 36142953	cmf	mandibular reconstruction plate	polymer_ot	am_polymer	comp	cadaver_phantom	0	20	plate-mandible distance 0.39 ± 0.21 vs 0.75 ± 0.31 mm; reconstruction deviation 1.11 ± 0.32 vs 1.67 ± 0.47 mm; reconstruction time

Study	Region	Implant	Material	Manufacturing	Pillars	Tier	Patients	Analysed units	Headline result
			her						16.67 ± 4.18 vs 33.78 ± 8.45 min
Merema et al. 2022. Journal of personalized medicine. PMID 36143224	cmf	TMJ total joint replacement	unclear	not_manufactured	imaging+comp	cadaver_phantom	0	40	mean Euclidean rotation-axis error 0.81 mm
Nimmawitt et al. 2022. Materials (Basel, Switzerland). PMID 36143689	cmf	root-analog dental implant	titanium	am_metal	comp	bench	0	16	RMSE 0.09 mm; vertical discrepancy 0.05–0.10 mm; all cortical bone stresses <100 MPa and all implant stresses <900 MPa; design B reduced type IV cancellous-bone stress
Rahimov et al. 2022. Annals of maxillofacial surgery. PMID 36199454	cmf	orbital floor implant	unclear	unclear	imaging+comp	cadaver_phantom	1	10	mean absolute deviations 0.334–0.575 mm across three measurements; ocular mobility improved and diplopia reduced in one clinical case
Wang et al. 2022. BMC surgery. PMID 36203147	pelvis_acetabulum	3D-printed hemipelvic prosthesis with dual mobility bearing	multiple	am_metal	comp	clinical_series	11	11	peak affected cortical bone 73.3 MPa; hemipelvic prosthesis 25.5 MPa; screws 34.3 MPa; design 2 d; fabrication, post-processing, and delivery 7 d; MSTS-93 21.38/30 in 8 patients; deep infection 1/11; recurrent dislocation 1/11
Spalthoff et al. 2022. Frontiers in oncology. PMID 36212406	cmf	mandibular reconstruction plate	unclear	am_metal	imaging+comp	clinical_comparative	83	83	mean communication duration 14.05 ± 13.58 d; simple 17.25 ± 15.77 d vs complex 12.97 ± 12.73 d, P = .337; surgery postponed in 16/83; 15/16 postponed cases were complex, P = 0.001
Rios et al. 2022. Journal of clinical medicine. PMID 36233363	cmf	maxillary fixation plate	titanium	am_metal	comp	clinical_series	22	22	mean absolute discrepancies 0.59 mm x-axis, 0.74 mm y-axis, 0.56 mm z-axis; total error 0.62 mm; all occlusions visually similar to planned occlusions
Wodzinski et al. 2022. Computer methods and programs in biomedicine. PMID 36257198	cranial	cranial implant	unclear	unclear	ai+imaging+comp	imaging_dataset	0	.	Dice 0.91; boundary Dice 0.94; 95th-percentile Hausdorff distance 1.53 mm
Kerkfeld et al. 2022. Journal of personalized medicine. PMID 36294792	cmf	orthognathic plates and facial augmentation implants	multiple	unclear	imaging+comp	clinical_comparative	30	30	facial symmetry improvement 6.5%P (3.2-9.8%P), p=0.001; PEEK implant exposure 2/8; no other PEEK complications
Wan et al. 2022. Journal of the mechanical behavior of biomedical materials. PMID 36302272	cmf	mandibular reconstruction plate	unclear	not_manufactured	comp	in_silico	0	1	appropriate graft remodeling extended predicted plate fatigue life; rising occlusal load led to predicted fatigue fracture
Xu et al. 2022. BMC musculoskeletal disorders. PMID 36329424	spine	vertebral body replacement prosthesis	titanium	not_manufactured	comp	in_silico	0	1	fixation strength 26.73 N·m/° in flexion; mismatched cage maximum von Mises stress 418.7 MPa in rotation

Study	Region	Implant	Material	Manufacturing	Pillars	Tier	Patients	Analysed units	Headline result
Annino et al. 2022. Oral oncology. PMID 36384079	cmf	mandibular reconstruction plate	unclear	unclear	comp	clinical_series	26	26	intercondylar distance deviation 1.46 mm (SD 2.4); intergonial 1.82 mm (SD 2.0); anterior-posterior 2.14 mm (SD 1.9); gonial angle 3.33° (SD 2.4); return to operating room 2/26; infection/dehiscence 11/26; preoperative pain resolved 20/21
Gong et al. 2022. Frontiers in bioengineering and biotechnology. PMID 36466328	spine	artificial lamina	titanium	not_manufactured	comp	in_silico	0	1	peak screw stress 46.53 MPa; peak artificial lamina stress 53.84 MPa in axial rotation
Ruf et al. 2022. Frontiers in bioengineering and biotechnology. PMID 36466355	cmf	mandibular reconstruction plates	titanium	not_manufactured	comp	in_silico	0	1	interosseous gap strains 0.07%-0.61%; maximum plate stress 8.9% of yield
Knitschke et al. 2022. Cancers. PMID 36497255	cmf	jaw reconstruction plate	titanium	am_metal	comp	clinical_comparative	133	332	incomplete osseous union 26.6% PSI vs 8.7% non-PSI at patient level; adjusted OR 3.518 (95% CI 1.223-10.124), P = 0.02
Zheng et al. 2022. Frontiers in bioengineering and biotechnology. PMID 36507282	cmf	mandibular lattice implant	titanium	am_metal	imaging+comp	bench	0	4	optimized maximum von Mises stress 697.35 MPa at the granulation stage; 0-3000 με in 93.36% of bone-graft nodes
Wan et al. 2022. Frontiers in bioengineering and biotechnology. PMID 36532579	shoulder	customized reverse shoulder prosthesis	multiple	am_metal	comp	in_silico	1	1	liner and scapula impingement stress 100-200 MPa; maximum fixation-claw deformation 3.6%, 2.8%, and 3.5% for neutral, flexion, and extension; satisfactory shoulder function and daily-life activities after revision
Annino et al. 2022. Laryngoscope investigative otolaryngology. PMID 36544971	cmf	mandibular reconstruction plate	titanium	multiple	comp	clinical_series	59	60	intercondylar deviation 2.10 mm; intergonial 2.23 mm; anterior-posterior 1.76 mm; gonial angle 3.11 degrees; inpatient return to operating room 8/60; outpatient return to operating room 16/60; hardware removal 9/60
Chai et al. 2022. OTA international : the open access journal of orthopaedic trauma. PMID 36569106	pelvis_acetabulum	pelvic fracture fixation plate	unclear	not_manufactured	imaging+comp	imaging_dataset	0	13	3D model viewing and export 39% and 38% faster with Drishti than 3D Slicer
Babazadeh-Naseri et al. 2022. Rice Digital Scholarship Archive (Rice University). Computational Framework for Designing Patient-specific, 3D-printable, and Stress	pelvis_acetabulum	pelvic tumour implant	unclear	not_manufactured	imaging+comp	in_silico	0	.	predicted bone-resorption volume significantly reduced vs solid implant
Pisaneschi et al. 2023.	cra	cranioplasty plate	m	am_pol	com	bench	1	3	FEA deflection 0.82 mm for

Study	Region	Implant	Material	Manufacturing	Pillars	Tier	Patients	Analysed units	Headline result
Progress in Additive Manufacturing. doi:10.1007/s40964-023-00452-5	nial		ultiple	ymer	p				LPBF PEEK versus 1.32 mm for TDS PCU at 500 N; 3D-printed-TPB PCU reached 6 mm at 459 N
Benady et al. 2023. Materials & Design. doi:10.1016/j.matdes.2023.111605	other	lattice bone-defect implant	titanium	am_metal	comp	in_silico	.	.	.
Schottey et al. 2023. Annals of 3D Printed Medicine. doi:10.1016/j.stlm.2023.100107	cmf	mandibular reconstruction implant	titanium	not_manufactured	comp	in_silico	0	2	implant volume reduced 49%; stress remained below yield strength and fatigue limit with similar performance after optimization
Alshewaier et al. 2023. Journal of Mechanics in Medicine and Biology. doi:10.1142/s021951942340016x	knee	customized knee implant	unclear	not_manufactured	comp	in_silico	0	15	active stress and strain [Formula: see text]–20[Formula: see text] MPa were lower; flexion-extension scaling factors 0.785 and 1.0
Jindal et al. 2023. Proceedings of the Institution of Mechanical Engineers Part L Journal of Materials Design and Applications. doi:10.1177/14644207231155761	cranial	cranial implant	peek	not_manufactured	comp	in_silico	0	.	maximum von Mises stress 51.985 MPa at 1 mm; optimized design 18.477 MPa under 1780 N and 15 mmHg
He et al. 2023. . doi:10.20944/preprints202307.0273.v1	lower_limb_other	talus replacement	unclear	not_manufactured	ai+imaging	imaging_dataset	0	.	DSC >0.95; HD95 <0.6 mm
D’Alpaos et al. 2023. . doi:10.20944/preprints202307.1681.v1	cmf	orbital floor implant	multiple	multiple	imaging+comp	clinical_series	14	14	.
Abed Hashmi et al. 2023. International Journal of Science and Research (IJSR). doi:10.21275/sr23722105819	cmf	mandibular implant	multiple	am_polymer	comp	in_silico	0	1	max deformation 20.273 mm for PMMA and 5.5563 mm for Ti-alloy under fixed support; maximum von Mises stress 30.18 MPa; fabrication time 1 day 22 h
Kladovasilakis et al. 2023. Technologies. doi:10.3390/technologies11020058	knee	tibial knee implant	unclear	not_manufactured	comp	in_silico	0	1	mass reduction around 45% with discrete-based topology optimization while maintaining mechanical performance
Snekhalatha et al. 2023. Biomedical Engineering: Applications, Basis and Communications. doi:10.4015/s1016237223500175	hip	patient-specific hip prosthesis	unclear	not_manufactured	comp	in_silico	0	3	custom femur 1.32 MPa at 500 N, 5.3 MPa at 2000 N, and 7.96 MPa at 3000 N; pelvic stress 23% lower than conventional
Rossi et al. 2023. Zenodo (CERN European Organization for Nuclear Research). doi:10.5281/zenodo.195425	spine	patient-specific spinal implant	titanium	am_metal	comp	cadaver_phantom	0	24	IBPS 0.924; stress shielding reduced 62% vs solid titanium constructs

Study	Region	Implant	Material	Manufacturing	Pillars	Tier	Patients	Analysed units	Headline result
54									
Shams et al. 2023. European journal of orthopaedic surgery & traumatology : orthopedie traumatologie. PMID 35001211	lower_limb_other	distal femur fixation plate	unclear	not_manufactured	comp	in_silico	0	4	maximum principal stress with custom plates decreased by 39.79%, 12.54%, 9.49%, and 20.29% across four models vs locking compression plate
Mekcha et al. 2023. Journal of prosthodontic research. PMID 35858803	cmf	nanohydroxyapatite bone block graft	ceramic	unclear	imaging	clinical_series	12	12	all 12 grafts adapted and fit without adjustment; graft success 10/12; horizontal bone gain 3.06 ± 1.02 mm from DICOM and 3.56 ± 0.23 mm from STL
Sun et al. 2023. Computer methods in biomechanics and biomedical engineering. PMID 36036151	hip	gradient porous femoral stem	titanium	not_manufactured	comp	in_silico	0	1	minimum predicted bone loss 2.4%; global safety factor ≥10
Demes et al. 2023. Journal of stomatology, oral and maxillofacial surgery. PMID 36184071	cmf	mandibular anterior subapical fixation plate	titanium	unclear	comp	clinical_series	11	11	absolute linear difference 0.66 mm (SD 0.51); median discrepancies 0.56 mm x-axis, 0.77 mm y-axis, 0.65 mm z-axis
Wandell et al. 2023. Journal of oral and maxillofacial surgery : official journal of the American Association of Oral and Maxillofacial Surgeons. PMID 36202213	cranial	cranioplasty implant	peek	unclear	imaging+comp	clinical_series	12	12	RMSE 0.66–3.1 mm; 11/12 implants had RMSE <2 mm
Van der Wel et al. 2023. International journal of oral and maxillofacial surgery. PMID 36202719	cmf	maxillary osteosynthesis plate	unclear	unclear	comp	rct	58	27	median translations <1 mm and rotations <1° in both groups
He et al. 2023. Oral surgery, oral medicine, oral pathology and oral radiology. PMID 36241591	cmf	individualized orbital wall mesh	titanium	unclear	imaging	clinical_series	10	10	orbital structure, volume, and SOF size restored in 10/10; SOFS symptoms completely disappeared in 10/10; no severe postoperative complications
Chen et al. 2023. Journal of orthopaedic translation. PMID 36313976	lower_limb_other	total talus prosthesis	cocr	not_manufactured	comp	in_silico	0	1	joint-motion and segment-movement R2 0.721-0.993 vs 0.623-0.990 for TAA; midtarsal contact force 17.92% higher than intact
Gamieldien et al. 2023. European journal of orthopaedic surgery & traumatology : orthopedie traumatologie. PMID 36385681	lower_limb_other	segmental bone-defect truss cage	titanium	am_metal	comp	clinical_series	9	9	functional union 9/9; peri- or post-operative complications 0/9
Leung et al. 2023. Clinical oral investigations. PMID 36401069	cmf	Le Fort I fixation implant	unclear	unclear	comp	clinical_series	25	25	median linear deviations x 0.74 mm, y 0.75 mm, z 0.72 mm; pitch 1.40°, yaw 0.90°, roll 0.60°; PSI-related postoperative complications

Study	Region	Implant	Material	Manufacturing	Pillars	Tier	Patients	Analysed units	Headline result
									0/25
Merta et al. 2023. Journal of plastic, reconstructive & aesthetic surgery : JPRAS. PMID 36563638	cmf	maxillary osteosynthesis plate	titanium	machined	comp	clinical_series	12	12	implants fitted planned drill holes and bone contours in 10/12 cases; skeletal relationships corrected successfully in 11/12 cases
Soloviev et al. 2023. Materials (Basel, Switzerland). PMID 36614737	pelvis_acetabulum	acetabular endoprosthesis	titanium	not_manufactured	imaging+comp	in_silico	0	1	maximum endoprosthesis stress <500 MPa vs 950 MPa critical stress; maximum contact opening 0.22 mm
Landau Prat et al. 2023. Seminars in ophthalmology. PMID 36639878	cmf	orbital floor implant	polymer_other	unclear	imaging	clinical_comparative	27	27	relative enophthalmos -1.1 mm PSI vs -1.7 mm PFI postoperatively (P = 0.1); primary or vertical gaze diplopia improved in both groups (P = 0.03)
Shao et al. 2023. BMC musculoskeletal disorders. PMID 36698116	lower_limb_other	intercalary femoral endoprosthesis	titanium	am_metal	imaging+comp	clinical_series	9	9	0/9 prostheses failed after 10^6 cycles at 250% body weight; operative duration 325.00 ± 62.60 min; HHS 69.22 ± 14.64 vs 91.33 ± 2.24 and MSTS 16.89 ± 7.08 vs 27.67 ± 1.73, both P < 0.001; implant survival 100%
Sun et al. 2023. Heliyon. PMID 36747923	spine	interbody cage	titanium	not_manufactured	comp	in_silico	0	3	commercial cage endplate peaks 40.3 and 43.2 MPa vs customized cage 29.7 and 26.4 MPa under rotation
Patel et al. 2023. Journal of maxillofacial and oral surgery. PMID 36776348	cmf	subperiosteal zygoma implant	titanium	am_metal	comp	clinical_series	21	21	implant stresses under maximal loading were below yield and ultimate strength; 2 infections; 3 prosthesis loosenings; 2 occlusal discrepancies; no infection or dehiscence after 6 mo
Zhu et al. 2023. Medical engineering & physics. PMID 36792235	pelvis_acetabulum	custom pelvic implant	unclear	unclear	comp	in_silico	0	1	average weighted peak von Mises stress increased 22-fold with frictional versus tied bone-implant contact
Dutta et al. 2023. Medical engineering & physics. PMID 36792242	cmf	mandibular reconstruction plate	titanium	unclear	comp	in_silico	0	4	no plate or screw stresses exceeded the titanium yield limit under the mastication cycle
Danielli et al. 2023. Journal of the mechanical behavior of biomedical materials. PMID 36801786	pelvis_acetabulum	acetabular and hemipelvis prostheses	titanium	am_metal	comp	bench	0	2	scale-dependent elastic-modulus reduction improved prediction of prosthesis stiffness and local strain distribution

Study	Region	Implant	Material	Manufacturing	Pillars	Tier	Patients	Analysed units	Headline result
Tantisatirapong et al. 2023. Scientific reports. PMID 36801943	cranial	cranioplasty plate	unclear	not_manufactured	imaging+comp	imaging_dataset	0	4	BMG mean accuracy 93.4%; Dice 0.65; Hausdorff distance 1.46 mm
Gong et al. 2023. Journal of surgical oncology. PMID 36825890	upper_limb_other	distal radius tumour endoprosthesis	unclear	unclear	comp	clinical_comparative	22	22	0° palmar tilt showed better biomechanical performance; subluxation rate significantly lower with 0° than biological-angle design
Matin-Mann et al. 2023. Journal of imaging. PMID 36826970	cranial	round window niche implant	polymer_other	am_polymer	imaging+comp	cadaver_phantom	0	23	3/3 semi-automated implants achieved pressure-tight fit versus 0/3 manual implants fitting; 3-5 min semi-automated vs around 30 min manual
Yang et al. 2023. Frontiers in bioengineering and biotechnology. PMID 36845197	cmf	custom short dental implant with wing retention	titanium	machined	comp	in_silico	0	9	planar wing maximum von Mises stress 30.2 MPa at 3 mm RBH and below 40 MPa at 1 mm RBH under 150 N vertical load
Burge et al. 2023. Scientific reports. PMID 36849812	knee	total knee replacement components	unclear	not_manufactured	ai+imaging+comp	imaging_dataset	0	165	femur model RMSE 0.87 (SD 0.20) mm and component RMSE 0.85 (SD 0.21) mm, OUH ≥3 mm in 6/84; tibia model RMSE 0.80 (SD 0.15) mm and component RMSE 0.82 (SD 0.27) mm, OUH ≥3 mm in 1/81; less than 10 min per CT for femoral and tibial components
Moiduddin et al. 2023. Polymers. PMID 36850170	cmf	zygomatic implant	peek	am_polymer	imaging+comp	cadaver_phantom	0	3	fabrication deviation 0.12 mm; outer fit deviation 0.4810 mm; region-of-interest deviation 0.2729 mm; maximum stress 0.89 MPa; maximum strain 2.2 × 10-4; maximum deformation 14 µm under 50 N; printing time 2 h 45 min
Fuchs et al. 2023. Clinical oral investigations. PMID 36864278	multi	craniofacial composite bone graft	multiple	multiple	imaging	cadaver_phantom	0	3	all implants matched the defect situation well; smaller marginal gaps or overlaps occurred in complex geometries; maximum stress 0.28 ± 0.08 MPa reinforced versus 0.27 ± 0.08 MPa unreinforced (p = 0.757)
Bevini et al. 2023. Journal of clinical medicine. PMID 36902725	cmf	mandibular reconstruction plate	titanium	am_metal	imaging+comp	clinical_series	10	10	mean total angular discrepancy 4.6032°; mean translation 2.6555 mm; mean absolute FCS surface deviation 1.0431 mm; procedure successful in 10/10; late flap necrosis with PSI replacement in 1/10; dental rehabilitation completed in 5/10

Study	Region	Implant	Material	Manufacturing	Pillars	Tier	Patients	Analysed units	Headline result
Kladovasilakis et al. 2023. Journal of the mechanical behavior of biomedical materials. PMID 36965217	lower_limb_other	tibial bone-gap scaffold	multiple	am_polymer	imaging+comp	cadaver_phantom	0	.	PLA SD&FCC lattice at 20% relative density showed the best overall performance
Singh et al. 2023. Medical engineering & physics. PMID 36965999	hip	femoral meta-implant	unclear	am_metal	comp	bench	0	3	FE-experimental strain R2 = 0.9821 and R2 = 0.9977; proximal-lateral von Mises strain increased 7.1% and 44.1% versus solid implant
Marzola et al. 2023. Computer methods and programs in biomedicine. PMID 37023601	cranial	cranioplasty implant	unclear	not_manufactured	imaging+comp	imaging_dataset	0	.	.
Chowdhury et al. 2023. Journal of maxillofacial and oral surgery. PMID 37041951	cmf	maxillofacial patient-specific implant	unclear	unclear	comp	clinical_series	8	8	.
Sharma et al. 2023. Journal of clinical medicine. PMID 37048579	cranial	cranioplasty plate	peek	am_polymer	imaging	cadaver_phantom	0	15	mean RMSE 0.16 (SD 0.09) mm; mean maximum Hausdorff distance 0.96 (SD 0.54) mm
Kim et al. 2023. BMC oral health. PMID 37138237	cmf	orthognathic fixation plate	titanium	am_metal	comp	clinical_comparative	12	12	mean deviation 0.485 ± 0.280 mm customized vs 1.213 ± 0.716 mm conventional; P<0.01; operation time 5.76 ± 0.43 h customized vs 6.83 ± 0.72 h conventional; P<0.05; plate exposure requiring replacement 1/6 customized patients
Hanusrichter et al. 2023. International orthopaedics. PMID 37145143	pelvis_acetabulum	partial pelvic replacement	unclear	unclear	comp	clinical_series	10	10	COR reconstructed correctly in 9/10; implantation time 236 (SD 64) min; 4 further operations in 2/10; no implant revision or aseptic loosening; Harris Hip Score 27 to 67
Grothues et al. 2023. Biomedizinische Technik. Biomedical engineering. PMID 37185164	knee	total knee implant	unclear	not_manufactured	ai+comp	imaging_dataset	0	673	prediction error below each parameter's standard deviation; regression had lowest errors
Lv et al. 2023. Frontiers in bioengineering and biotechnology. PMID 37214294	spine	sacral prosthesis	unclear	unclear	comp	clinical_series	12	12	FEA predicted no fracture or stress shielding; design to surgery about 2 wk; deep infection with implant removal 1/12; osseointegration 12/12; no implant mechanical failure
Fortunato et al. 2023. Bioengineering (Basel, Switzerland). PMID 37237631	cranial	in situ bioprinted cranial scaffold	multiple	am_polymer	imaging+comp	cadaver_phantom	0	1	fiducial registration error 1.50 mm; deposition path error 1.38 ± 0.37 mm; printing procedure 1 h 40 min; whole workflow <2 h excluding imaging vs traditional surgery up to 3 h

Study	Region	Implant	Material	Manufacturing	Pillars	Tier	Patients	Analysed units	Headline result
Pietzka et al. 2023. Journal of personalized medicine. PMID 37240877	cmf	maxillary osteosynthesis plate	unclear	am_metal	comp	clinical_comparative	126	1424	dental root injuries 10/651 screws in conventional cohort vs 0/773 PSI screws (p < 0.001)
Pietzka et al. 2023. Journal of personalized medicine. PMID 37241016	cmf	orbital implant	titanium	am_metal	imaging+comp	clinical_comparative	75	75	implant inaccuracy 66.6% preformed vs 10% PSI; intraoperative revision 26.6% vs 13%; postoperative revision 11.1% vs 0% PSI
Rana et al. 2023. Journal of the mechanical behavior of biomedical materials. PMID 37244075	hip	patient-specific hip implant	titanium	am_metal	comp	bench	0	.	average stress shielding 56% solid vs 18% optimized; strain correlation coefficient 0.9633-0.9844
Abdelazeem et al. 2023. World neurosurgery. PMID 37302708	cranial	frontal cranioplasty mesh	titanium	unclear	imaging+comp	clinical_series	15	15	complications 4/15; favorable cosmetic outcomes 15/15
Xu et al. 2023. Acta neurochirurgica. PMID 37351673	cranial	cranioplasty implant	pmma	multiple	imaging+comp	cadaver_phantom	0	4	cosmesis 4/5 in 2 specimens and 3/5 in 2 specimens
Zhang et al. 2023. Materials (Basel, Switzerland). PMID 37445036	knee	tibial implant	titanium	am_metal	imaging+comp	bench	0	24	topology-optimized porous structures had compressive strength >400 MPa; target elastic modulus 3.031–10.528 GPa
Wang et al. 2023. Zhongguo xiu fu chong jian wai ke za zhi = Zhongguo xiufu chongjian waike zazhi = Chinese journal of reparative and reconstructive surgery. PMID 37460178	upper_limb_other	lunate prosthesis	titanium	not_manufactured	comp	cadaver_phantom	0	1	prosthesis stress significantly greater than normal lunate; wrist load-transfer mode unchanged
Csámer et al. 2023. Scientific reports. PMID 37491550	cranial	cranioplasty implant	pmma	cast	imaging+comp	clinical_series	52	54	no macroscopical gaps during validation; clinical fittings adequate; septic complications 2/54; implants removed and later replaced
Tel et al. 2023. Scientific reports. PMID 37495645	cmf	patient-specific maxillofacial osteosynthesis plate	titanium	not_manufactured	comp	imaging_dataset	0	20	implant von Mises stress 20–50 MPa; mean Le Fort I maxilla design time 22.5 min versus 102.5 min traditional; P < 0.0001
Ebker et al. 2023. The British journal of oral & maxillofacial surgery. PMID 37517940	cmf	maxillomandibular advancement fixation implant	unclear	unclear	comp	clinical_series	63	63	major complications 2/63; mean hospital stay 4 d
Manekar et al. 2023. Journal of maxillofacial and oral surgery. PMID 37534346	cmf	maxillary patient-specific implant	unclear	not_manufactured	comp	in_silico	0	3	stress and strain values low and acceptable; QZI showed more anterior-region stress
Bregoli et al. 2023. Medical engineering & physics. PMID 37536840	upper_limb_other	thumb osseointegrated fixture	unclear	unclear	comp	cadaver_phantom	0	.	safety factor 1.5 guaranteed

Study	Region	Implant	Material	Manufacturing	Pillars	Tier	Patients	Analysed units	Headline result
Singh et al. 2023. Journal of the mechanical behavior of biomedical materials. PMID 37544200	cranial	cranioplasty implant	multiple	am_polymer	imaging+comp	cadaver_phantom	0	2	titanium stresses comparable to PEEK; titanium strains and deformation much smaller than PEEK
Jindal et al. 2023. Journal of the mechanical behavior of biomedical materials. PMID 37556925	cranial	cranioplasty implant with fixation plates	multiple	not_manufactured	comp	in_silico	0	1	stresses reduced nearly 2.5 times versus the initial design and remained within yielding limits
Moncayo-Matute et al. 2023. Journal of the mechanical behavior of biomedical materials. PMID 37562162	cranial	cranioplasty implant	pmma	not_manufactured	comp	in_silico	0	1	no failure predicted in the implant-bone interface or anchoring system
Vitković et al. 2023. Materials (Basel, Switzerland). PMID 37570113	upper_limb_other	extra-articular distal humerus plate	polymer_other	am_polymer	comp	cadaver_phantom	0	1	maximum deviations reduced about 5% for both models; plate production time 1 h 10 min
Moiduddin et al. 2023. Journal of functional biomaterials. PMID 37623673	cranial	cranioplasty implant	peek	am_polymer	imaging+comp	cadaver_phantom	0	5	average outward deviation 0.4436 mm; maximum von Mises stress 61.92 MPa under 50 N; maximum deformation 6 microns; fabrication time 4 h 25 min
Banerjee et al. 2023. Journal of biomedical materials research. Part B, Applied biomaterials. PMID 37624362	cmf	total temporomandibular joint implant	unclear	unclear	comp	in_silico	0	.	.
Van Kootwijk et al. 2023. Journal of the mechanical behavior of biomedical materials. PMID 37678107	cmf	mandibular reconstruction cage	titanium	am_metal	comp	cadaver_phantom	3	5	FEA-DIC strain correlation r = 0.92 with SE = 29.32 με; simulated union reduced local implant peak stress 72.7 ± 1.5%
Kim et al. 2023. Annals of plastic surgery. PMID 37713150	cranial	cranioplasty implant	titanium	am_metal	imaging	clinical_comparative	35	35	root mean square deviation 2.58 ± 0.93 mm vs 4.82 ± 1.31 mm; complications 2/12 vs 6/23; reoperation 2/12 vs 5/23
Zhao et al. 2023. BMC oral health. PMID 37730602	cmf	mandibular reconstruction mesh	titanium	am_metal	imaging+comp	clinical_series	7	7	mean 3D deviation 0.78 ± 0.41 mm; bone-volume planning error 2.44% ± 2.10%; serious infections or complications 0/7; graft resorption 32.15% ± 6.95%
Wang et al. 2023. Frontiers in bioengineering and biotechnology. PMID 37744254	spine	individualized zero-profile interbody cage	titanium	not_manufactured	comp	in_silico	0	1	NIZP cage peak stress 310.0-440.0 MPa vs CPC 410.0-540.0 MPa across four motion conditions
Kuehle et al. 2023. Journal of clinical medicine. PMID 37762977	cmf	maxillary osteosynthesis plate	titanium	am_metal	imaging+comp	clinical_comparative	50	130	back/front deviation -0.63 ± 1.62 mm vs -1.3 ± 2.54 mm; down/up deviation -1.29 ± 1.59 mm vs -2.69 ± 3.1 mm
Liu et al. 2023. Frontiers in bioengineering and biotechnology. PMID 37790252	pelvis_acetabul	integrated acetabular prosthesis	unclear	am_metal	comp	clinical_series	5	5	IAP peak micromotion 1.5-11.2 μm vs MAP 9.7-45.2 μm under 210-4200 N; implant peak stress 25.8 MPa vs 83.1 MPa at 4200 N; Harris hip score 46.5 ± 4.8

Study	Region	Implant	Material	Manufacturing	Pillars	Tier	Patients	Analysed units	Headline result
	um								preoperative to 85.4 ± 5.5 final; complications 0/5
Imai et al. 2023. The British journal of oral & maxillofacial surgery. PMID 37863724	cmf	pre-bent orthognathic fixation plate	unclear	unclear	comp	clinical_series	34	34	median repositioning deviation 0.26 mm maxilla and 0.69 mm mandible; plate-hole deviation 0.23 mm maxilla and 0.36 mm mandible
Zhong et al. 2023. Journal of the mechanical behavior of biomedical materials. PMID 37875041	cmf	mandibular reconstruction plate	unclear	not_manufactured	comp	in_silico	0	4	non-locking system had higher plate stress, lower screw stress, and equivalent displacement versus locking
Chai et al. 2023. 3D printing and additive manufacturing. PMID 37886407	pelvis_acetabulum	pelvic fracture fixation plate	unclear	unclear	imaging+comp	imaging_dataset	0	13	lower geometrical deviation on re-stitched fracture side than mirrored nonfractured bone
Haque et al. 2023. Biomimetics (Basel, Switzerland). PMID 37887630	cranial	cranioplasty plate	multiple	not_manufactured	comp	in_silico	0	1	optimal fixation 4-5 points; screw spacing approximately 40 mm for smaller implants and 60 mm for larger implants
Lv et al. 2023. Journal of orthopaedic surgery and research. PMID 37936217	pelvis_acetabulum	acetabular fracture fixation plate	titanium	not_manufactured	comp	in_silico	0	1	maximum stress 420.75-592.90 MPa bespoke vs 3922.64-3946.58 MPa common plate under 2300 N
Sun et al. 2023. BMC oral health. PMID 37940900	cmf	pre-bent mandibular reconstruction plate	titanium	unclear	imaging+comp	clinical_series	15	15	osteotomy error 1.01 ± 0.90 mm; reconstruction angular error 1.70 ± 1.41°; operative time 487.25 ± 60.84 min with simultaneous implant placement and 397.18 ± 73.09 min otherwise; infections 2/15; plate and one bone segment removed 1/15
Babazadeh-Naseri et al. 2023. Medical engineering & physics. PMID 37985018	pelvis_acetabulum	pelvic tumour endoprosthesis	unclear	not_manufactured	comp	in_silico	0	1	peri-prosthetic bone volume predicted to undergo resorption 18% vs 44%
Zhao et al. 2023. Journal of clinical medicine. PMID 38137607	cmf	zygomatic titanium mesh and fixation plates	titanium	am_metal	imaging+comp	clinical_series	5	5	mean 3D deviation 1.07 ± 0.15 mm; maximum 3D deviation 3.11 ± 0.44 mm; severe complications 0/5; bone resorption 45.24% ± 3.13% at 6 mo
Wu et al. 2023. Frontiers in bioengineering and biotechnology. PMID 38149174	cranial	cranioplasty implant	pmma	cast	ai+comp	imaging_dataset	1	1930	ACVAI 2.22% vs 2.05% manual; PCVAI 2.14% vs 6.03% manual; implant design <10 min vs >1 h manual; postoperative

Study	Region	Implant	Material	Manufacturing	Pillars	Tier	Patients	Analysed units	Headline result
									complications 0/1 at >6 mo
S et al. 2024. Procedia Computer Science. doi:10.1016/j.procs.2024.04.312	cranial	cranioplasty plate	unclear	not_manufactured	ai+comp	imaging_dataset	0	.	higher Dice than 3D U-Net and attention 3D U-Net
Mineo et al. 2024. IOP Conference Series Materials Science and Engineering. doi:10.1088/1757-899x/1306/1/012017	cmf	mandibular reconstruction plate	unclear	unclear	comp	bench	0	.	.
Kesornsri et al. 2024. IEEE Access. doi:10.1109/access.2024.3415173	cranial	cranioplasty plate	unclear	not_manufactured	ai	imaging_dataset	0	.	Dice 0.7969 ± 0.13
Nazlı et al. 2024. Orthopaedic Proceedings. doi:10.1302/1358-992x.2024.18.002	upper_limb_other	lunate implant	unclear	unclear	imaging+comp	cadaver_phantom	0	2	contralateral lunate exceeded intra-subject variation in five shape modes; shape mode 3 significantly affected scapholunate and capitolunate angles ($p<0.05$)
Julie et al. 2024. Orthopaedic Proceedings. doi:10.1302/1358-992x.2024.18.082	lower_limb_other	tibial defect implant	polymer_other	am_polymer	comp	bench	0	2	Voronoi and Gyroid structures adapted more precisely to complex and irregular outer implant shapes than NaCl
Nourishirazi et al. 2024. Journal of Craniomaxillofacial Research. doi:10.18502/jcr.v10i4.15313	cmf	mandibular reconstruction implant	titanium	not_manufactured	comp	in_silico	0	3	maximum von Mises stress approximately 120 MPa; minimum jaw deviation
Honigmann et al. 2024. . doi:10.21203/rs.3.rs-4049296/v1	upper_limb_other	scaphoid prosthesis	titanium	not_manufactured	imaging	imaging_dataset	0	20	mean absolute surface distance 0.17 mm; maximum 0.64 mm
Zhang et al. 2024. . doi:10.21203/rs.3.rs-4729506/v1	knee	distal femoral segmental prosthesis	titanium	not_manufactured	imaging+comp	in_silico	0	3	maximum prosthesis stress 853.58 MPa single plate vs 254.62 MPa double plate vs 55.411 MPa intramedullary nail at 90°
Mejía Rodríguez et al. 2024. Designs. doi:10.3390/designs8020031	cranial	cranioplasty implant	multiple	not_manufactured	comp	in_silico	0	.	.
Moya et al. 2024. Technologies. doi:10.3390/technologies12080125	cranial	cranioplasty plate	unclear	unclear	comp	bench	0	.	.
G. et al. 2024. International Journal of Civil Environmental and Agricultural Engineering.	hip	hip implant	unclear	not_manufactured	comp	in_silico	0	.	customized implant outperformed the standard implant in FEA

Study	Region	Implant	Material	Manufacturing	Pillars	Tier	Patients	Analysed units	Headline result
doi:10.34256/ijceae2324									
Scherb et al. 2024. DS 133: Proceedings of the 35th Symposium Design for X (DFX2024). doi:10.35199/dfx2024.02	hip	revision femoral stem	unclear	not_manufactured	comp	in_silico	0	57	38° antetorsion: maximum stem-cement interface failure 8.46% shear and 3.92% tensile; 8° and 18°: no tensile or shear failure
Block et al. 2024. . doi:10.53846/goediss-10683	cmf	mandibular reconstruction plate	unclear	unclear	imaging+comp	clinical_comparative	29	29	commercial planning superior for whole-mandible deviation (p = 0.0217), intersegmental space volume (p = 0.0035), and length (p = 0.0007)
S. et al. 2024. . doi:10.62791/20368	knee	distal femur replacement	unclear	unclear	comp	in_silico	0	1	build orientation significantly affected shape distortion and residual stress
Demir et al. 2024. Computer methods in biomechanics and biomedical engineering. PMID 36920278	cmf	mandibular reconstruction plate	unclear	not_manufactured	comp	in_silico	0	9	central defect maximum von Mises stress 643.62 MPa conventional versus 101.83 MPa biplanar custom plate
Assink et al. 2024. European journal of trauma and emergency surgery : official publication of the European Trauma Society. PMID 37391531	knee	medial tibial plateau osteosynthesis plate	titanium	machined	imaging+comp	cadaver_phantom	0	6	median plate-position deviation 3.0 mm (IQR 2.8–3.7); median screw-direction deviation 3.3° (IQR 2.5–5.1)
Jirangkul et al. 2024. Archives of orthopaedic and trauma surgery. PMID 37658855	upper_limb_other	precontoured clavicle dynamic compression plate	unclear	unclear	imaging	clinical_series	40	40	plate fitted well in 40/40 cases; mean union time 12.88 weeks; Constant-Murley score 96.33 ± 3.66; ASES score 93.26 ± 5.15; implant removal 2/40
Ruf et al. 2024. Computers in biology and medicine. PMID 38064852	cmf	mandibular reconstruction miniplate	multiple	not_manufactured	comp	in_silico	0	1	mechanical strains increased locally under magnesium fixation
Merta et al. 2024. Journal of cranio-maxillo-facial surgery : official publication of the European Association for Cranio-Maxillo-Facial Surgery. PMID 38129183	cmf	mandibular osteotomy fixation plate	unclear	unclear	comp	clinical_comparative	53	53	no difference in skeletal stability between VSP-PSI and conventional mini-plate groups
Wang et al. 2024. Journal of shoulder and elbow surgery. PMID 38182026	upper_limb_other	coronoid prosthesis	unclear	not_manufactured	imaging+comp	in_silico	0	6	autograft articular stress 7.2-68 MPa; prosthesis reproduced intact contact mechanics
Xue et al. 2024. BMC oral health. PMID 38233785	cmf	mandibular reconstruction plate	titanium	am_metal	comp	in_silico	1	10	clinical plate maximum von Mises stress 475.81 MPa INC and 423.21 MPa L-MOL
Mohseni-Dargah et al. 2024. Journal of the mechanical behavior of biomedical materials. PMID 38237204	other	middle ear ossicular prosthesis	unclear	unclear	comp	cadaver_phantom	0	.	.
Oldhoff et al. 2024. European journal of trauma	uppe	distal radius osteosynthesis plate	titani	machined	imaging+	cadaver_phan	0	20	median rotation 2.6°, dorsal angulation 1.4°, radial

Study	Region	Implant	Material	Manufacturing	Pillars	Tier	Patients	Analysed units	Headline result
and emergency surgery : official publication of the European Trauma Society. PMID 38261077	r_limb_other		um		comp	tom			angulation 4.7°, and translation 2.4 mm; PSI design within 1 d; manufacture within 3 d
Li et al. 2024. Orthopaedic surgery. PMID 38296795	lower_limb_other	personalized lattice bone-defect implant	titanium	am_metal	imaging+comp	clinical_series	7	7	MSTS 27.3 (range 26-29); implant-related complications 0/7
Chen et al. 2024. Journal of dental sciences. PMID 38303793	cmf	customized mandibular body prosthesis	titanium	not_manufactured	comp	in_silico	0	25	whole-assembly von Mises stress 482.04-1076.26 MPa across 4 CMP designs
Zhang et al. 2024. Frontiers in bioengineering and biotechnology. PMID 38333079	lower_limb_other	tumour endoprosthesis	titanium	am_metal	comp	clinical_series	7	7	MSTS 24.9 ± 2.9; postoperative complications 0/7
Longhitano et al. 2024. Journal of the mechanical behavior of biomedical materials. PMID 38340477	cmf	mandibular reconstruction prosthesis	unclear	not_manufactured	imaging+comp	in_silico	0	1	maximum von Mises equivalent stress significantly lower than commercial plate; bone strains within maintenance and remodeling intervals
Khalaf et al. 2024. Journal of cranio-maxillo-facial surgery : official publication of the European Association for Cranio-Maxillo-Facial Surgery. PMID 38368205	cmf	orbital floor mesh	titanium	unclear	imaging+comp	clinical_series	7	7	orbital volume reduced from 29.59 cc to 27.21 cc; 0.6% smaller than intact orbit; diplopia resolved in 2/3 patients; postoperative enophthalmos mean 0.2229 mm vs 0.9914 mm preoperatively
Consorti et al. 2024. Journal of cranio-maxillo-facial surgery : official publication of the European Association for Cranio-Maxillo-Facial Surgery. PMID 38368208	cmf	customized orbital implant	unclear	unclear	imaging+comp	clinical_comparative	52	52	plate-position difference 0.62 mm (SD 0.235) vs 0.69 mm (SD 0.246); orbital-volume difference 0.95 ml vs 1.02 ml; clinical improvements and complications similar between groups
Krasovsky et al. 2024. Journal of cranio-maxillo-facial surgery : official publication of the European Association for Cranio-Maxillo-Facial Surgery. PMID 38388230	cmf	orbital reconstruction implant	titanium	unclear	imaging+comp	clinical_comparative	23	23	reconstruction inaccuracy 0.58 mm with PSI vs 1.54 mm with conventional mesh
Yan et al. 2024. Aesthetic plastic surgery. PMID 38409347	cranial	forehead augmentation implant	peek	unclear	comp	clinical_series	11	11	mean operative time 83.00 ± 29.44 min; FACE-Q satisfaction with forehead 47.64 ± 7.15 preoperatively to 78.81 ± 6.35 at 6 months; implant exposure, extrusion or removal 0/11

Study	Region	Implant	Material	Manufacturing	Pillars	Tier	Patients	Analysed units	Headline result
Iratwar et al. 2024. Journal of biomaterials applications. PMID 38423069	cranial	cranioplasty flap	pmma	unclear	imaging	clinical_series	10	10	mean surgery time 90 ± 20 min; no intraoperative or postoperative cerebrospinal fluid leakage, hematoma, or infection; excellent aesthetic outcomes except in a few cases
DeCarvalho et al. 2024. Medical & biological engineering & computing. PMID 38433178	knee	distal femur replacement	unclear	not_manufactured	imaging+comp	in_silico	0	1	maximum shape distortion 18.5 mm; build orientation significantly affected residual stresses
Müller et al. 2024. Scientific reports. PMID 38459092	hip	individualized hip endoprosthesis	unclear	am_metal	imaging+comp	bench	0	1	RMSE of stress deviations reduced 81% in GZ 6 and 66% in GZ 7; CCC > 0.97; optimization 23.5 h
García-Ávila et al. 2024. Journal of the mechanical behavior of biomedical materials. PMID 38492502	lower_limb_other	transtibial orthopedic implant	polymer_other	am_polymer	imaging+comp	bench	0	1	internal porosity reduced weight without compromising isotropic behavior
Li et al. 2024. Journal of orthopaedic surgery and research. PMID 38561755	pelvis_acetabulum	lattice hemipelvis prosthesis	titanium	am_metal	imaging+comp	clinical_series	12	12	MSTS 21 mean (range 18–24); complications 3/12; no revision, infection, screw breakage, or prosthesis loosening
Maintz et al. 2024. 3D printing in medicine. PMID 38639834	cmf	multiple cranio-maxillofacial implants	polymer_other	am_polymer	comp	cadaver_phantom	0	.	wall thickness down to 0.8 mm; minimal gap formation on bone models; fabrication 5–60 min
Hu et al. 2024. Journal of orthopaedic surgery and research. PMID 38654343	pelvis_acetabulum	hemipelvic endoprosthesis	titanium	am_metal	comp	clinical_comparative	32	32	acetabular eccentricity 93.7 ± 5.3 mm affected vs 94.7 ± 4.3 mm healthy mediolaterally; 55.6 ± 4.8 mm vs 54.3 ± 4.7 mm superior-inferior; Type I+II affected-leg stance peak stress 303.6 MPa vs 1074 MPa; surgery time 426.2 ± 67.0 min vs 301.7 ± 48.6 min; MSTS 24.3 ± 1.8 vs 21.8 ± 2.0; HHS 79.8 ± 5.2 vs 75.3 ± 3.5
Lee et al. 2024. Journal of orthopaedic research : official publication of the Orthopaedic Research Society. PMID 38669091	pelvis_acetabulum	flanged acetabular component	unclear	not_manufactured	comp	in_silico	0	2	additional lateralization slightly increased bone area at risk of failure and decreased areas compatible with bone ingrowth
Hu et al. 2024. BMC musculoskeletal disorders. PMID 38755628	pelvis_a	custom hemipelvic endoprosthesis	titaniu	am_metal	comp	clinical_series	60	60	design and fabrication 1–2 weeks; complications 13/60 (21.7%); bone integration

Study	Region	Implant	Material	Manufacturing	Pillars	Tier	Patients	Analysed units	Headline result
	cetabulum		m						128/130 (98.5%); VAS 5.5 ± 1.4 to 1.7 ± 1.3; MSTS-93 14.8 ± 2.5 to 23.0 ± 5.6
D'Alpaos et al. 2024. Journal of personalized medicine. PMID 38793041	cmf	orbital floor implant	multiple	multiple	imaging+comp	clinical_comparative	23	23	postoperative globe protrusion difference 1.14 mm mean (range 0.27–3.69 mm); mean surgical time 74 min vs 90 min; no implant-related complications or reoperations in 13 PSI patients
Zhao et al. 2024. Regenerative biomaterials. PMID 38854680	hip	custom hip joint prosthesis	unclear	am_metal	comp	clinical_comparative	33	33	maximum stress 332.73 MPa at 35% original weight; no damage after 5 million stem cycles or 10 million neck cycles; 33/33 had no infection, deep vein thrombosis, or wound nonunion; implants stable without loosening or osteolysis at 6 mo
Chen et al. 2024. BMC musculoskeletal disorders. PMID 38880911	lower_limb_other	custom femoral segmental prosthesis	titanium	am_metal	comp	clinical_series	11	11	union 10/11 (91%); deep infection 1/11; prosthetic dislocation 2/11
Seo et al. 2024. Medical engineering & physics. PMID 38906577	lower_limb_other	patient-specific helical femoral bone plate	unclear	am_metal	imaging+comp	bench	0	1	.
Kang et al. 2024. Frontiers in bioengineering and biotechnology. PMID 38966191	spine	patient-specific vertebral body replacement implant	titanium	not_manufactured	comp	in_silico	0	2	anisotropic model reduced maximum von Mises stress by 2-37 MPa in vertebra and 20-61 MPa in reconstruction system
Ettinger et al. 2024. Journal of oral and maxillofacial surgery : official journal of the American Association of Oral and Maxillofacial Surgeons. PMID 38981580	cmf	mandibular reconstruction plate	titanium	am_metal	comp	clinical_comparative	130	130	mean RMSE 1.15 mm PSI vs 1.46 mm stock; difference 0.31 mm (95% CI 0.16-0.47)
Nourishirazi et al. 2024. Journal of orthopaedics. PMID 38983674	knee	patient-specific femoral diaphyseal cone	unclear	not_manufactured	comp	in_silico	0	5	cone increased stress up to 30% and reduced femoral canal-cone relative motion nearly 60%
Kaye et al. 2024. World neurosurgery. PMID 38996963	spine	occipitocervical fixation plate	titanium	am_metal	imaging+comp	cadaver_phantom	0	5	.
Garner et al. 2024. Journal of orthopaedic research : official publication of the Orthopaedic Research	pelvis_acet	patient-specific acetabular component	unclear	not_manufactured	comp	in_silico	0	15	bone-implant contact +63%; reamed bone volume -97% vs hemispherical implants

Study	Region	Implant	Material	Manufacturing	Pillars	Tier	Patients	Analysed units	Headline result
Society. PMID 39004739	abulum								
Yurteri et al. 2024. Archives of orthopaedic and trauma surgery. PMID 39008077	upper_limb_other	patient-specific clavicle fracture plate	multiple	not_manufactured	comp	in_silico	0	4	plate peak von Mises stress 764.79 MPa titanium vs 234.74 MPa PEEK
Piovan et al. 2024. Journal of orthopaedic surgery and research. PMID 39068461	knee	patient-specific metaphyseal cone	titanium	am_metal	comp	in_silico	1	5	custom-made cones produced more homogeneous and lower bone stress than stem techniques under 2200 N and 1000 N loads
Oberoi et al. 2024. Dental materials : official publication of the Academy of Dental Materials. PMID 39084954	cmf	subperiosteal jaw implant	ceramic	unclear	comp	in_silico	1	2	bulk Young's modulus 205 GPa and maximum principal stress 61.14 MPa; lattice 83.3 GPa and 278.63 MPa under 125-250 N per abutment; further iteration successfully implanted in 1 patient
Zhang et al. 2024. Zhonghua wai ke za zhi [Chinese journal of surgery]. PMID 39090062	pelvis_acetabulum	acetabular augment/triflange cup	unclear	am_metal	comp	clinical_comparative	109	109	last rotation-center height 22.4±9.0 mm augment and 22.7±6.0 mm triflange; LLD 6.0±4.0 mm and 5.3±2.2 mm
Amin et al. 2024. Surgical neurology international. PMID 39108397	cranial	cranioplasty plate	peek	am_polymer	imaging+comp	clinical_series	11	11	fit and cosmetic outcome satisfactory by arbitrary assessment; seroma 5/11; mild fever 3/11; no infection, breakage, resorption, neurological deficit, or seizure
Geroski et al. 2024. Medical engineering & physics. PMID 39160013	cranial	cranioplasty implant	pmma	cast	imaging	clinical_series	5	5	good match without additional refinement in 5/5 surgeries
Smit et al. 2024. Journal of the mechanical behavior of biomedical materials. PMID 39186906	spine	interbody cage	unclear	unclear	comp	bench	0	2	subsidence risk reduced 91% for normal bone quality and 54% for osteopenia versus off-the-shelf cage
Mazzocchetti et al. 2024. Scientific reports. PMID 39191797	multi	craniofacial implant guide surface	unclear	not_manufactured	ai+comp	imaging_dataset	0	71	PointAttN accuracy 1.4468 mm; Chamfer distance 3.2731 mm; F-score 0.8929
Betti et al. 2024. Journal of orthopaedic surgery and research. PMID 39210457	lower_limb_other	osseointegrated transfemoral stem	unclear	not_manufactured	comp	imaging_dataset	0	140	cortical bone removed 3.15 vs 5.42 cm³ at level A; distal contact area 52.3% vs 32.2% at level B
Datarkar et al. 2024. National journal of maxillofacial surgery. PMID	cmf	maxillary PSI with strut abutments	uncle	unclear	comp	in_silico	0	1	maximum stress 2 MPa at the zygomaticomaxillary

Study	Region	Implant	Material	Manufacturing	Pillars	Tier	Patients	Analysed units	Headline result
39234141			ar						buttress
Zhu et al. 2024. Frontiers in bioengineering and biotechnology. PMID 39234264	pelvis_acetabulum	custom pelvic implant	titanium	am_metal	comp	in_silico	1	1	BL-CL, ISO, ORT and FOR predicted Cancellous 3 pullout; screw fatigue limit 542.2 MPa; total computation time 9.9 h BL-CL vs 40.8-71.9 h iterative methods; Cancellous 3 screw pullout required revision surgery
Parhiz et al. 2024. BioMed research international. PMID 39238768	cmf	modular mandibular implant	titanium	not_manufactured	comp	in_silico	0	2	horseshoe stress approximately 200 MPa; stress concentration approximately 350 MPa; reaction force under 200 N at 1 mm deflection
Sun et al. 2024. Scientific reports. PMID 39242632	pelvis_acetabulum	hemipelvic tumour prosthesis	titanium	am_metal	comp	in_silico	1	6	maximum iliac-screw stress 295.7 MPa original vs 209.7 MPa with pubic and iliac-back fixation (29.1% decrease); prosthesis loosening and broken screw at 6 mo
Zhang et al. 2024. Journal of materials chemistry. B. PMID 39246118	cranial	skull osteoinductive implant	unclear	unclear	ai+comp	bench	0	.	designs consistent with experimental and finite-element relationships between lattice parameters and elastic modulus
Wodzinski et al. 2024. Computers in biology and medicine. PMID 39265478	cranial	cranioplasty implant	unclear	not_manufactured	ai+imaging	imaging_dataset	0	.	Dice >0.94 on SkullBreak and >0.96 on SkullFix
Sabelis et al. 2024. Journal of cranio-maxillo-facial surgery : official publication of the European Association for Cranio-Maxillo-Facial Surgery. PMID 39266434	cmf	orbital reconstruction implant	unclear	not_manufactured	comp	imaging_dataset	0	60	median design difference 37% for proposals, 26% for final designs with different engineers, and 22% between surgeons; up to 40% design-time reduction
Wolf et al. 2024. Materials (Basel, Switzerland). PMID 39274630	lower_limb_other	total talus replacement implant	multiple	multiple	imaging+comp	bench	0	.	average and mean mesh distances <0.2 (unit not reported) below 60% mesh reduction; PEEK 50% infill maximum reaction-force difference 24% vs bone
Smit et al. 2024. Frontiers in bioengineering and biotechnology. PMID 39318669	spine	interbody cage	multiple	not_manufactured	comp	in_silico	0	7	median subsidence-risk reduction 89% for titanium and 94% for PEEK vs off-the-shelf cage; 75% for PEEK vs anatomically conforming cage
Zhao et al. 2024. Materials (Basel, Switzerland). PMID 39410466	hip	femoral stem	titanium	am_metal	comp	bench	0	9	TO-B compressive strength 258.88 MPa at 60% porosity
Caruso et al. 2024. Craniomaxillofacial trauma & reconstruction. PMID 39553819	cmf	mandibular fracture fixation plate	unclear	unclear	comp	clinical_series	5	5	high degree of accuracy in implant placement and alignment compared with preoperative virtual plans; successful fracture reduction

Study	Region	Implant	Material	Manufacturing	Pillars	Tier	Patients	Analysed units	Headline result
									in 5/5 patients
Shash et al. 2024. BMC musculoskeletal disorders. PMID 39563300	cranial	cranioplasty plate and mesh	multiple	not_manufactured	comp	in_silico	0	8	CFR-PEEK 60% plate produced skull tensile/compressive stresses of 21.64/24.24 MPa vs 24.99/25.88 MPa for titanium under 2000 N
Jakimiuk et al. 2024. 3D printing in medicine. PMID 39693002	upper_limb_other	distal radius osteotomy wedge	multiple	am_polymer	imaging+comp	cadaver_phantom	0	5	maximum compressive force 1211.01-2736.42 N for patient-specific wedges vs 2415.55 N for standard plate
Li et al. 2024. Biomaterials translational. PMID 39734704	pelvis_acetabulum	hemipelvic prosthesis	titanium	am_metal	imaging+comp	clinical_series	6	6	hip centre discrepancy 5.1 ± 1.4 mm horizontal; 4.2 ± 0.7 mm vertical; Young's modulus 1.68 ± 0.05 GPa; compressive strength 174 ± 8 MPa; MSTS 23.2 (range 20-25); delayed wound healing 1/6; no aseptic loosening or structural failure
Wodzinski et al. 2024. Annual International Conference of the IEEE Engineering in Medicine and Biology Society. IEEE Engineering in Medicine and Biology Society. Annual International Conference. PMID 40040189	cranial	cranioplasty implant	unclear	not_manufactured	ai	imaging_dataset	0	.	.
Pisaneschi et al. 2025. Progress in Additive Manufacturing. doi:10.1007/s40964-025-01098-1	cranial	cranial plate	polymer_other	am_polymer	comp	bench	0	1	FEA vs experiment displacement 1.07 mm vs 1.14 mm at 500 N; fracture at 1800 N and 6 mm displacement; von Mises stress 64.5 MPa at 500 N
Po-Kuei et al. 2025. Materials & Design. doi:10.1016/j.matdes.2025.113668	lower_limb_other	distal femoral defect reconstruction implant	titanium	am_metal	comp	bench	0	.	all implants withstood cyclic loads without damage
Palmisciano et al. 2025. Journal of Neurological Surgery Part B Skull Base. doi:10.1055/s-0045-1803877	cmf	craniomaxillofacial reconstruction implant	unclear	unclear	imaging+comp	clinical_series	10	10	registration accuracy qualitatively comparable to traditional navigation; intermittent virtual object drift; registration 10 min mean (range 8–15); AR use 20 min mean (range 15–30); excellent bony reduction and functional outcomes in all cases
Chaubey et al. 2025. . doi:10.1145/3774521.3774531	chest_w	ribcage implant	unclear	not_manufactured	ai	imaging_dataset	0	160	DSC 0.2524; Hausdorff distance 148.90 mm

Study	Region	Implant	Material	Manufacturing	Pillars	Tier	Patients	Analysed units	Headline result
	all								
Jindal et al. 2025. Proceedings of the Institution of Mechanical Engineers Part L Journal of Materials Design and Applications. doi:10.1177/14644207241312947	cranial	cranioplasty plate	multiple	not_manufactured	comp	in_silico	0	6	three curved fixture plates sustained 950 N without failure; PSI mass 7 g
Daum et al. 2025. FACE. doi:10.1177/27325016251352171	cmf	zygomatic implant	unclear	unclear	comp	clinical_series	8	8	high degree of fidelity to preoperative plan; complications 0/8; visual changes 0/8 at latest follow-up
П. et al. 2025. Orthopaedic Proceedings. doi:10.1302/1358-992x.2025.8.050	upper_limb_other	proximal humerus locking plate	unclear	unclear	comp	cadaver_phantom	0	19	significantly improved stability vs standard implant; patient-specific implants had moderate but significant benefit vs standardized optimized implants
Ovcharenko et al. 2025. Mechanics and Advanced Technologies. doi:10.20535/2521-1943.2025.9.2(105).336223	knee	revision femoral knee prosthesis	titanium	not_manufactured	comp	in_silico	0	4	.
Moncayo-Matute et al. 2025. . doi:10.20944/preprints202507.1208.v1	multi	cranial orbital and chest-wall implants	multiple	multiple	comp	clinical_series	7	7	surgery time 1.15-2.3 h; no significant intra- or postoperative complications; adequate postoperative functionality in all 7 cases
Honigmann et al. 2025. . doi:10.21203/rs.3.rs-5401931/v1	upper_limb_other	scaphoid prosthesis	unclear	not_manufactured	imaging	cadaver_phantom	0	20	frozen volume 1257.43 mm³ vs thawed 1259.22 mm³ (p > 0.5); tight-to-smooth mean absolute distance 0.17 mm, maximum 0.64 mm
Shang et al. 2025. . doi:10.21203/rs.3.rs-6931418/v1	knee	tumour knee prosthesis stem	titanium	am_metal	imaging+comp	bench	0	9	YG60 yield strength 192.2 ± 9.1 MPa; gait micromotion remained below 150 µm
Dehaghani et al. 2025. . doi:10.25904/1912/5981	hip	paediatric proximal femoral osteotomy fixation device	unclear	unclear	comp	bench	0	.	.
Javier et al. 2025. Journal of Orthopedic Research and Therapy. doi:10.29011/2575-8241.001385	pelvis_acetabulum	custom acetabular prosthesis	polymer_other	am_polymer	imaging+comp	cadaver_phantom	0	10	mean translational error 3.79 mm (SD 2.09 mm); mean angular error 3.73° (SD 2.59°); mean implant placement time 121.5 s (95% CI 59.8–183.2 s)
Alkentar et al. 2025. Applied Sciences. doi:10.3390/app15073522	hip	patient-specific lattice hip implant	titanium	not_manufactured	comp	in_silico	0	.	average deformation deviation from original model below 5%; simulation time reduced by more than 100 times
Zhu et al. 2025. Applied	spi	lumbar interbody	un	not_ma	com	in_silic	0	4	V-Cage reduced ROM 51%

Study	Region	Implant	Material	Manufacturing	Pillars	Tier	Patients	Analysed units	Headline result
Sciences. doi:10.3390/app15105384	ne	fusion cage	clear	nufactured	p	o			in extension, cage stress 41.7% in compression, and endplate stress 63.7% in right bending versus C-Cage
Tanveer et al. 2025. Applied Sciences. doi:10.3390/app152212099	knee	total knee femoral component	unclear	not_manufactured	comp	in_silico	0	2	reduced peak von Mises stress with more uniform stress distribution and improved mass efficiency
C.-H. et al. 2025. International Journal of Bioprinting. doi:10.36922/ijb025260250	cmf	mandibular reconstruction implant with drug reservoir	titanium	am_metal	comp	bench	0	9	implant stress <40% of Ti6Al4V yield strength; fatigue endurance >1 million loading cycles
Yildirim et al. 2025. Journal of Medicine and Dentistry. doi:10.64951/jmdnt.2026.1.04	cmf	bimaxillary orthognathic fixation implant	unclear	unclear	comp	clinical_comparative	46	46	mean translational and rotational deviations within predefined clinical limits (≤2 mm and ≤2°); no conversion to splint-based transfer 0/46
Rasheed et al. 2025. Dijlah Journal of Medical Sciences P-ISSN 3078-3178 E-ISSN 3078-8625. doi:10.65204/djms-multi-factor-biomechanical	cranial	cranial implant	multiple	not_manufactured	comp	in_silico	0	144	material effect: stress P=5.06E-15; deformation P=4.7E-35
Moris et al. 2025. Journal of stomatology, oral and maxillofacial surgery. PMID 39182846	cmf	orbital reconstruction implant	titanium	am_metal	imaging	clinical_series	12	12	enophthalmos 3.24 mm preoperative to 0.17 mm postoperative; orbital volume difference 3.38 mL to 0.37 mL; enophthalmos and diplopia resolved; Lancaster test normal in 10/12 (83.3%)
Liu et al. 2025. Orthopaedic surgery. PMID 39429061	lower_limb_other	partial bone-defect prosthesis	titanium	am_metal	comp	clinical_series	8	8	maximum residual-bone von Mises stress 22.1 to 1.2 MPa at distal femur and 37.2 to 22.1 MPa at distal tibia; infection recurrence 2/8; LEFS 31.5 ± 9.0 to 61.0 ± 4.1 in remaining 6 patients
Dogan et al. 2025. Computers in biology and medicine. PMID 39602978	cmf	mandibular reconstruction plate and hybrid scaffold	multiple	am_polymer	imaging+comp	in_silico	0	2	Voronoi pattern reduced mass by 37% without losing endurance
Maintz et al. 2025. International journal of computer assisted radiology and surgery. PMID 39671058	cranial	fronto-orbital fixation plates	multiple	am_polymer	imaging+comp	cadaver_phantom	0	3	osteotomy median deviation 0.44 mm; FOA median point-to-point distance 1.27 mm; osteotomy approximately 3 h; six implants printed in approximately 3.5 h
Jitkla et al. 2025. Computers in biology and medicine. PMID 39700854	spine	sacral reconstruction prosthesis	unclear	not_manufactured	comp	in_silico	0	.	maximum instrument von Mises stress reduced approximately 50-80% vs FRR and FRACR
Huang et al. 2025. The Journal of bone and joint surgery. American volume. PMID 39729975	pelvis_acetabulu	hemipelvic prosthesis	titanium	am_metal	imaging+comp	clinical_comparative	28	28	mean hip rotation centre displacement 14.12 ± 8.75 mm; peak sacral-screw stress 157.1 MPa with 3 screws vs 81.4 MPa with lumbosacral fixation under 700 N; prosthesis preparation approximately

Study	Region	Implant	Material	Manufacturing	Pillars	Tier	Patients	Analysed units	Headline result
	m								10 d; implant-related complications: breakage 4/28, aseptic loosening 4/28, dislocation 2/28; MSTS-93 18.2 ± 5.3
Trott et al. 2025. Head & neck. PMID 39731307	cmf	maxillary reconstruction plate	unclear	unclear	comp	clinical_series	39	39	plate-related complications 14/39 (35.8%); removal 10/39 (25.6%); exposure 12/39 (30.8%); loose screws 2/39 (5.1%); fracture 0/39
Merema et al. 2025. Scientific reports. PMID 39753636	cmf	mandibular continuity-defect reconstruction implant	peek	machined	comp	bench	0	7	mean failure load 1138 N PEEK-reconstructed vs 1268 N intact; no failure at 1.1 million cycles
Shash et al. 2025. Scientific reports. PMID 39788988	chest_wall	rib and costal-cartilage implants	multiple	not_manufactured	comp	in_silico	0	1	under impact, minimum fatigue safety factor 0.413 alumina, 0.388 PEEK, 0.213 PE; predicted failure
Dubron et al. 2025. Scientific reports. PMID 39814827	cmf	orbital reconstruction plate	unclear	not_manufactured	imaging+comp	imaging_data set	0	160	self-overestimation mean difference MR vs 3D -0.662 (95% CI -1.252 to -0.073), p=0.028; total workflow time 23 min 48 s MR vs 1 h 22 min conventional 3D
Le et al. 2025. The Journal of craniofacial surgery. PMID 39819879	cmf	mandibular reconstruction plate	titanium	am_metal	comp	clinical_comparative	18	18	medial-lateral condylar displacement 2.5 ± 1.42 mm PSP vs 4.92 ± 2.42 mm PBP; coronal axis deviation 1.46 ± 1.16° vs 5.45 ± 2.75°; total operation time 487.7 ± 90.1 min PSP vs 547.3 ± 110.1 min PBP
Saloniemi et al. 2025. Oral and maxillofacial surgery. PMID 39853440	cmf	subcondylar mandibular repositator plate	titanium	machined	comp	clinical_series	10	10	mean postoperative plate-position discrepancy at cranial screw hole 0.2 mm horizontally, 0.1 mm anteroposteriorly, and 0.6 mm vertically; planning and plate delivery within 24 h; no major complications or reoperation; satisfactory occlusion in 10/10
Oldhoff et al. 2025. European journal of trauma and emergency surgery : official publication of the European Trauma Society. PMID 39856352	lower_limb_other	corrective osteotomy plate	titanium	machined	comp	cadaver_phantom	0	12	median plan deviation with PSI 2.2° varus/valgus, 1.2° ante/recurvation, 3.5° rotation; osteotomy-plane error 1.4 mm; PSI designed, manufactured and applied within 5 days
Zhu et al. 2025. Journal of orthopaedic surgery and research. PMID 39856781	pelvis_acetabulum	pelvic reconstruction prosthesis	titanium	not_manufactured	comp	in_silico	0	3	Scheme C pelvis peak stress 56.414 MPa (Type 1), 98.31 MPa (Type 2), and 97.199 MPa (Type 3) under simulated walking
Rios et al. 2025. Journal of cranio-maxillo-facial surgery : official publication of the European Association	cmf	mandibular angle implant	titanium	unclear	comp	clinical_series	18	18	mean volumetric angle expansion 4.6 mm; jaw angle parameters concordant with ideal female and male

Study	Region	Implant	Material	Manufacturing	Pillars	Tier	Patients	Analysed units	Headline result
for Cranio-Maxillo-Facial Surgery. PMID 39875225									parameters
Alshewaier et al. 2025. PloS one. PMID 39899500	knee	total knee implant	multiple	not_manufactured	comp	in_silico	0	1	femoral bone stress 23.38 MPa with implant vs 24.53 MPa without implant under 1000 N and 1.5 N-m
Yang et al. 2025. Scientific reports. PMID 39900958	cmf	orbital wall implant	multiple	am_polymer	comp	clinical_series	40	40	median RMS conformance distance 1.073 mm at postoperative 6 months; mean implant insertion time 19.8 s (range 3-60); no surgery- or implant-associated complications; enophthalmos, EOM limitation and central diplopia 0/40 at 6 months
Sun et al. 2025. International orthopaedics. PMID 39921749	pelvis_acetabulum	acetabular titanium block	titanium	am_metal	comp	clinical_series	35	35	V-COR 50.1 ± 4.7 mm preoperatively to 19.7 ± 1.8 mm postoperatively; H-COR 33.1 ± 11.8 mm to 29.7 ± 1.7 mm; HHS 44.5 ± 10.0 preoperatively to 85.1 ± 7.4 at last follow-up; complications 3/35
Bevini et al. 2025. Journal of cranio-maxillo-facial surgery : official publication of the European Association for Cranio-Maxillo-Facial Surgery. PMID 39933970	cmf	orthognathic fixation implant	unclear	unclear	comp	rct	20	20	both approaches clinically equivalent in precision; maxilla-guided approach had lower discrepancies; no surgical complications
Li et al. 2025. Foot and ankle surgery : official journal of the European Society of Foot and Ankle Surgeons. PMID 39947960	lower_limb_other	total talar prosthesis	unclear	not_manufactured	comp	in_silico	0	5	TTP-FP1.5 caused significant biomechanical changes versus TTP0; ATFL reconstruction had a minor effect
Li et al. 2025. Journal of biomechanical engineering. PMID 39992375	lower_limb_other	total talar prosthesis	unclear	not_manufactured	comp	in_silico	0	5	TTP-FP1.5 and TTP-FP3 reduced talar tilt; TTP-VP1.5 and TTP-VP3 increased tibial contact force
Wodzinski et al. 2025. Computer methods and programs in biomedicine. PMID 40009971	cranial	cranioplasty implant	unclear	not_manufactured	ai	imaging_dataset	0	.	DSC 0.94; bDSC 0.94; HD95 1.31 versus 0.84/0.76/2.43 baseline
Vaziri et al. 2025. European journal of orthopaedic surgery & traumatology : orthopedie traumatologie. PMID 40047935	knee	patient-specific porous cone	titanium	am_metal	comp	clinical_series	10	19	preoperative-intraoperative alignment and precise defect reconstruction in 10/10 cases; no loosening or implant migration at early follow-up
Nayak et al. 2025. 3D printing in medicine. PMID 40067641	cmf	jaw reconstruction plate	titanium	am_metal	comp	in_silico	0	1	FEA stress levels within safe limits for titanium 3D-printed implants; design time significantly reduced vs traditional CAD

Study	Region	Implant	Material	Manufacturing	Pillars	Tier	Patients	Analysed units	Headline result
Zhou et al. 2025. Computer methods and programs in biomedicine. PMID 40107082	other	orthopaedic bone screw	unclear	not_manufactured	ai+comp	in_silico	0	80	prediction error 2-6% for nonlinear machine-learning models; optimized designs improved pull-out stiffness approximately 16% and strength approximately 14% versus average designs
Mejía Rodríguez et al. 2025. The Journal of prosthetic dentistry. PMID 40121172	cmf	TMJ prosthesis	unclear	not_manufactured	comp	in_silico	0	1	maximum von Mises stress 31 MPa; minimum principal stress -52.68 MPa
Akkoyun et al. 2025. Medicina oral, patologia oral y cirugia bucal. PMID 40121675	cmf	genioplasty fixation plate	titanium	not_manufactured	comp	in_silico	0	12	lowest bone displacement 0.92 mm narrowing and 0.79 mm widening; stable configurations peak PSI stress 805.946 MPa
Ceddia et al. 2025. Materials (Basel, Switzerland). PMID 40141579	pelvis_acetabulum	acetabular tri-flange	multiple	not_manufactured	comp	in_silico	0	2	maximum bone strain 1200.478 µm/m titanium vs 1632.887 µm/m PEEK
Benady et al. 2025. Medicina (Kaunas, Lithuania). PMID 40142287	knee	patient-specific intercalary cage	titanium	am_metal	comp	clinical_series	6	6	no complications observed after transition to printed Ti64 implants
Rakityansky et al. 2025. Zhurnal voprosy neirokhirurgii imeni N. N. Burdenko. PMID 40183614	cranial	cranioplasty implant	pmma	cast	imaging+comp	clinical_series	32	32	postoperative-to-virtual skull deviation and cranial asymmetry assessed; values not reported; craniometric asymmetry measurements showed effective skull correction
Cheng et al. 2025. International journal of oral and maxillofacial surgery. PMID 40199670	cmf	orthognathic fixation plate	unclear	not_manufactured	comp	in_silico	0	2	maximum von Mises stress 227.63 MPa for Le Fort I and 237.31 MPa for genioplasty; design time 94 min for Le Fort I and 45 min for genioplasty
Sarigul Sezenoz et al. 2025. European journal of ophthalmology. PMID 40232250	cmf	orbital implant	polymer_other	am_polymer	imaging	clinical_series	8	8	exophthalmometry asymmetry improved from 3.1 mm to 0.5 mm; enophthalmos improved in 7/8; hypoglobus in 3/5; diplopia in 3/4
Li et al. 2025. Orthopaedic surgery. PMID 40254773	knee	custom tibial plateau fixation plate	titanium	multiple	comp	clinical_series	10	10	peak VMS 260.98 MPa customized vs 170.79 MPa conventional; maximum displacement 0.26124 mm vs 0.61878 mm; fabrication 3–4 days; operative time 1.37 ± 0.58 h; Lysholm 88.6 ± 4.93; knee ROM 103.5° ± 11.56°; EQ-5D-3L 0.87 ± 0.47; no notable postoperative complications
Hu et al. 2025. Orthopaedic surgery. PMID 40310728	pelvis_acet	custom hemipelvic endoprosthesis	titanium	am_metal	comp	clinical_series	12	12	MSTS-93 15 to 26; VAS 5 to 1; poor wound healing 2/12; no mechanical failure, loosening, fracture, or

Study	Region	Implant	Material	Manufacturing	Pillars	Tier	Patients	Analysed units	Headline result
	abulum								dislocation
Sahni et al. 2025. Proceedings of the Institution of Mechanical Engineers. Part H, Journal of engineering in medicine. PMID 40326366	cmf	customized dental implant assembly	unclear	am_metal	comp	bench	0	2	static stress 38.89-77.81 MPa vs 75.47-158.09 MPa (case 1); 83.95-106.65 MPa vs 55.23-126.57 MPa (case 2); resonance 7763.2 Hz vs 7003.6 Hz (case 1) and 7910.1 Hz vs 7102.1 Hz (case 2)
Kasapoglu et al. 2025. International journal of medical sciences. PMID 40386052	cmf	subperiosteal maxillary implant	titanium	not_manufactured	comp	in_silico	0	2	maximum displacement 0.58 mm vs 4.50 mm; peak screw stress 295 MPa vs 510 MPa
Łoginoff et al. 2025. Journal of cranio-maxillo-facial surgery : official publication of the European Association for Cranio-Maxillo-Facial Surgery. PMID 40399182	cmf	subperiosteal maxillary implant	titanium	am_metal	comp	clinical_series	10	10	8/10 implants functional and stable; 2/10 removed for inadequate fit and recurrent infection; 10-year survival probability 80%
Andres et al. 2025. Bioengineering (Basel, Switzerland). PMID 40428165	cmf	custom temporomandibular joint prosthesis	multiple	unclear	imaging+comp	bench	1	5	CAD-to-segmentation distances mostly below 1 mm; static failure load 1540 N mean; fossa wear 0.624 mm3/10^6 cycles; condyle wear 0.0688 mm3/10^6 cycles
Mohamed et al. 2025. Journal of oral and maxillofacial surgery : official journal of the American Association of Oral and Maxillofacial Surgeons. PMID 40480278	cmf	mandibular reconstruction plate	titanium	am_metal	comp	clinical_comparative	265	265	hardware-related complications in 72/265; stock plates vs patient-specific implants HR 1.80, 95% CI 1.11–2.91, P = .0171
Juneja et al. 2025. Computers in biology and medicine. PMID 40483854	cranial	cranioplasty implant	unclear	unclear	ai+comp	imaging_dataset	0	.	DSC 0.889; Hausdorff distance 1.856 mm; SSIM 0.897; load-bearing capacity confirmed under 1780 N; reconstruction 0.1 s vs 2400 s CAD; end-to-end 93.5 s vs 2820 s CAD
Mao et al. 2025. Journal of the mechanical behavior of biomedical materials. PMID 40494102	hip	porous hip implant	unclear	unclear	comp	bench	0	.	lower stiffness and more natural load transmission than conventional design
Gupta et al. 2025. Journal of hand and microsurgery. PMID 40503244	upper_limb_other	patient-specific upper-limb fixation plate	titanium	am_metal	imaging+comp	clinical_series	7	7	planned corrections achieved on postoperative radiographs; no plate breakage in patient-specific plate cases; planning 2–3 d; plate fabrication 3–4 d; all fractures united within 6 wk; DASH scores improved in all 8 cases; complications in 3/7 patient-specific plate cases
Zeng et al. 2025. European journal of medical research. PMID 40517264	lower_lim	total talus prosthesis	unclear	not_manufactured	comp	in_silico	0	1	0.5 mm cartilage overlay most closely approximated normal ankle stress distribution; model

Study	Region	Implant	Material	Manufacturing	Pillars	Tier	Patients	Analysed units	Headline result
	b_other								validation peak contact pressure 2.7681 MPa vs 2.7–4.0 MPa experimental
Guezou-Philippe et al. 2025. PloS one. PMID 40561157	knee	total knee arthroplasty components	unclear	not_manufactured	ai+imaging+comp	imaging_dataset	0	40	segmentation Dice 98.5% (SD 0.8%); full-bone RMSE 1.0 mm (SD 0.3); implant-fit RMSE 0.9 mm (SD 0.5); whole workflow about 15 min
Ruf et al. 2025. Computers in biology and medicine. PMID 40614522	cmf	mandibular reconstruction plate	unclear	not_manufactured	comp	in_silico	0	3	higher stress in pre-stressed plate; healing-region strains unaffected by pre-bending
Ruan et al. 2025. Journal of orthopaedic surgery and research. PMID 40629372	lower_limb_other	total talar prosthesis	multiple	not_manufactured	comp	in_silico	0	720	1.5-mm buffer below 43.32 MPa or 3-mm buffer below 96.94 MPa produced peak cartilage stress comparable to native condition
Kang et al. 2025. Scientific reports. PMID 40702034	lower_limb_other	total talar prosthesis	cocr	am_metal	comp	clinical_series	6	3	PVMS 218.01 MPa rational scaffold vs 565.35 MPa topologically optimized under 5340 N; mean VAS 8.0 (SD 1.7) preoperatively to 3.3 (SD 0.6) at 3 mo; median FAOS 48.0 [IQR 17-76] to 61.0 [IQR 48-72]
Jarayabhand et al. 2025. Journal of orthopaedic surgery (Hong Kong). PMID 40726307	multi	patient-specific fixation plate	unclear	am_metal	comp	clinical_series	11	11	CT-to-manufacture time 3-43 days; mean 16 days; all 11 achieved bony union, good-to-excellent clinical outcomes, and no complications
Shi et al. 2025. 3D printing in medicine. PMID 40736597	spine	total sacral endoprosthesis	unclear	am_metal	imaging+comp	clinical_series	9	9	mean angular deviation 4.27° (IQR 4.15); mean position deviation 4.00 mm (IQR 2.90); R0 resection 9/9; perioperative complications 3/9; screw breakage 1/9
Hijazi et al. 2025. Journal of the mechanical behavior of biomedical materials. PMID 40753708	cmf	intraosseous mandibular implant	titanium	not_manufactured	comp	in_silico	0	2	predicted functional life 4–119 years under cyclic 50–100 N masticatory forces; no stress shielding
Roshdy et al. 2025. Computers in biology and medicine. PMID 40779986	cmf	subperiosteal jaw implant	titanium	unclear	comp	in_silico	0	2	framework von Mises stress 60.74 MPa single-piece vs 150.09 MPa two-piece under 50 N oblique loading
Chen et al. 2025. BMC ophthalmology. PMID 40826038	cmf	absorbable orbital reconstruction plate	polymer_other	unclear	imaging+comp	clinical_comparative	94	94	postoperative enophthalmos 0.000 (0.000–0.500) mm vs 0.500 (0.000–0.500) mm; orbital volume discrepancy 0.1100 (0.0525–0.3050) mm3 vs 0.2550 (0.0900–0.3600) mm3; operative duration 55.41 ± 19.76 min vs 98.00 ± 18.52 min; no infection, implant displacement, or vision deterioration; superior

Study	Region	Implant	Material	Manufacturing	Pillars	Tier	Patients	Analysed units	Headline result
									diplopia resolution P = 0.023 and ocular mobility restoration P = 0.049
Bao et al. 2025. The Journal of craniofacial surgery. PMID 40845376	cmf	mandibular angle and chin augmentation implants	peek	unclear	comp	clinical_series	6	12	chin abscess 1/6; no revision surgery; all patients very satisfied
Ekşi Altan et al. 2025. Proceedings of the Institution of Mechanical Engineers. Part H, Journal of engineering in medicine. PMID 40876815	cranial	cranioplasty plate	titanium	am_metal	comp	bench	0	1	isotruss implant sustained 18,000 N with approximately 50% weight reduction and von Mises stress 40 MPa
Chang et al. 2025. Frontiers in bioengineering and biotechnology. PMID 40909220	cmf	BSSO fixation plate	titanium	am_metal	imaging+comp	bench	0	30	TOPO proof load 326.90 N right and 389.85 N left vs 52.37 N commercial; mandibular displacement 0.45 ± 0.04 mm vs 0.66 ± 0.15 mm
Zhong et al. 2025. Journal of biomechanical engineering. PMID 40913392	cmf	mandibular reconstruction plate	titanium	am_metal	comp	in_silico	0	2	PSMRP plate stress 141 MPa vs 261 MPa during incisal biting and 139 MPa vs 305 MPa during molar clenching; plate displacement 0.47 mm vs 0.80 mm and 0.48 mm vs 0.74 mm
Moncayo-Matute et al. 2025. Journal of personalized medicine. PMID 41003100	multi	customized cranial and clavicular implants	multiple	multiple	imaging+comp	clinical_series	7	7	surgical duration reduced approximately 30%; no significant intra- or postoperative complications in 7 cases
Beisekenov et al. 2025. Journal of functional biomaterials. PMID 41003421	cmf	mandibular reconstruction plate	titanium	am_metal	ai+imaging+comp	bench	0	.	held-out test MAE 7.8 MPa for peak plate stress, 0.062 mm for displacement, and 6.4 MPa for screw stress; peak von Mises stress 225 MPa and peak deflection 2.46 mm under 600 N; design iteration <5 ms vs ~8 min FEM; full-cycle development time reduced ~25%
Yu et al. 2025. Journal of orthopaedic surgery and research. PMID 41023710	spine	interbody cage	titanium	not_manufactured	imaging+comp	in_silico	0	10	SSM leave-one-out RMS error 1.7 ± 0.3 mm with 17 PCs and 1.6 ± 0.3 mm with all PCs; ROM decreased 10.2%–20.7% after cage insertion; personalised models and customised cage generated in approximately 30 min
Ren et al. 2025. Head & face medicine. PMID 41039504	cmf	absorbable orbital wall plate	polymer_other	unclear	imaging+comp	clinical_comparative	106	106	6-month interocular exophthalmometry difference 0.144 ± 0.3805 mm vs 0.447 ± 0.5985 mm; residual diplopia 9.2% vs 31.6%; no implant-related infection or displacement in absorbable group
Orassi et al. 2025. Clinical oral investigations. PMID	cmf	mandibular reconstruction plate	titani	am_metal	comp	in_silico	0	1	predicted bone formation 39% in anterior gap and

Study	Region	Implant	Material	Manufacturing	Pillars	Tier	Patients	Analysed units	Headline result
41114876			um						69% in posterior gap after 90 days; ossification threshold 0.30 MPa improved agreement with clinical data
Shen et al. 2025. 3D printing in medicine. PMID 41128973	spine	expandable corpectomy cage	titanium	multiple	imaging+comp	bench	0	3	axial compression stiffness 2127 ± 146 N/mm and yield load 8547 ± 1213 N; PECS subsidence stiffness 546.71 ± 93.7 N/mm vs FECS 148.38 ± 4.02 N/mm
Seo et al. 2025. Scientific reports. PMID 41131218	multi	pelvic and tibial tumour endoprostheses	titanium	am_metal	comp	bench	0	60	Ti dissolution after 7 d in Hartmann's solution: solid 82.8 µg/L; hybrid 90.5 µg/L; lattice 128 µg/L
Sheta et al. 2025. BMC oral health. PMID 41176602	cmf	orbital wall implant	titanium	machined	imaging+comp	rct	28	28	reconstructed-to-intact orbital volume difference 0.6543 ± 0.2767 cm³ vs 1.666 ± 0.3884 cm³; PSIs delivered within 5–7 days; residual enophthalmos 1/14 vs 3/14; residual diplopia 0/14 vs 1/14
Banis et al. 2025. Journal of experimental orthopaedics. PMID 41180554	hip	cementless femoral stem	titanium	am_metal	ai+comp	cadaver_phantom	0	1300	FEA-DIC total displacement difference 0.17 ± 0.09 mm; optimal stem length 18.5% ± 2.7% of femur length; mean micromotion 28.5 ± 6.75 µm
Li et al. 2025. Bone & joint research. PMID 41186144	pelvis_acetabulum	custom acetabular component	titanium	not_manufactured	comp	in_silico	0	2	gait-cycle peak stress 126.90 MPa for enhanced triflange versus 140.70 MPa for quadri-flange; maximum micromotion 79 versus 163 µm
Wang et al. 2025. Frontiers in bioengineering and biotechnology. PMID 41194981	knee	integrated tibial prosthesis	cocr	am_metal	comp	clinical_series	6	6	reported inconsistently: at 2100 N cortical stress 16.69 versus 30.00 MPa in the abstract and 16.32 versus 20.03 MPa in Results; micromotion 7.1 versus 13.0 µm in the abstract and 45.7 versus 49.9 µm in Results; KSS 46.5 ± 4.8 preoperatively to 85.4 ± 5.5 at 24 mo; no radiolucency, loosening, osteolysis, or complications in 6 patients
Yüceer-Çetiner et al. 2025. Materials (Basel, Switzerland). PMID 41227783	cmf	total TMJ prosthesis	multiple	not_manufactured	comp	in_silico	0	2	mandibular component von Mises stress 192.18 MPa with Ti6Al4V versus 204.31 MPa with CoCrMo; fossa stress 92.004 versus 94.182 MPa
Safavi et al. 2025. Journal of orthopaedic surgery and research. PMID 41239450	hip	porous femoral stem	titanium	not_manufactured	comp	bench	0	8	linear proximal porous stem reduced stress shielding 9.5% during walking and 8.1% during stair climbing; proximal porous and solid shell stems predicted to pass ISO 7206-4

Study	Region	Implant	Material	Manufacturing	Pillars	Tier	Patients	Analysed units	Headline result
Sagar et al. 2025. Journal of cranio-maxillo-facial surgery : official publication of the European Association for Cranio-Maxillo-Facial Surgery. PMID 41266230	cmf	mandibular reconstruction implant	unclear	not_manufactured	comp	in_silico	0	6	without fibular graft SCI maximum stress was up to 77.7% higher than CI; with graft up to 56.3% higher
Canko et al. 2025. BMC oral health. PMID 41272638	cmf	subperiosteal jaw implant	multiple	not_manufactured	comp	in_silico	0	8	implant von Mises stress 444.538 MPa for I-shaped Co-Cr without cantilever versus 623.024 MPa for Y-shaped Co-Cr with cantilever under 200 N oblique load
Honigmann et al. 2025. Bioengineering (Basel, Switzerland). PMID 41301138	upper_limb_other	scaphoid prosthesis	unclear	not_manufactured	imaging	cadaver_phantom	0	20	frozen-thawed volume difference 0.14% (SD 0.55); smoothing surface deviations up to 0.64 mm
Lv et al. 2025. Journal of orthopaedic surgery and research. PMID 41361902	spine	artificial vertebral body	multiple	multiple	comp	clinical_comparative	21	21	mean subsidence 0.37 mm with tantalum AVB vs 4.1 mm with titanium mesh; peak implant stress 515.7 MPa with titanium mesh vs <50 MPa with titanium and tantalum AVBs; operation duration 323.3 ± 90.2 min vs 364.4 ± 60.9 min; 2-yr VAS 0.7 ± 0.6 vs 1.8 ± 0.6; fusion 100% vs 38.9%; rod fracture 0/3 vs 6/18
Guo et al. 2025. Frontiers in bioengineering and biotechnology. PMID 41383719	pelvis_acetabulum	split-type triflange acetabular cup	titanium	am_metal	imaging+comp	clinical_series	14	14	planned-achieved COR difference 1.4 mm AP, 0.1 mm ML, 0.3 mm SI; 9/14 within 5° for inclination and anteversion; at 4200 N, peak flange stress 70.744 MPa normal and 80.366 MPa osteoporotic; P1 micromotion 122.861 and 131.244 μm; design 2 d; manufacturing, post-processing and delivery 7 d; HHS 31.9 ± 8.5 to 82.9 ± 5.9; OHS 7.6 ± 2.3 to 35.4 ± 3.1; 2/14 complications; no implant failure
Liokatis et al. 2025. Maxillofacial plastic and reconstructive surgery. PMID 41385022	cmf	mandibular condylar fixation plate	titanium	unclear	imaging+comp	clinical_series	8	10	mean joint-surface deviation 2.27 mm; median point deviation 2.56 mm; surgery duration 250 min median; median mouth opening 37 mm at 1 yr; 7/8 satisfied; 1 screw loosening with ectopic bone
Tang et al. 2025. Zhongguo xiu fu chong jian wai ke za zhi = Zhongguo xiufu chongjian waike zazhi = Chinese journal of reparative and reconstructive surgery.	pelvis_acetabul	custom porous acetabular component	unclear	unclear	comp	clinical_series	8	8	hip centre shift 2.1 ± 0.5 mm horizontal and 2.0 ± 0.7 mm vertical relative to contralateral side; operation time 187 min mean; HHS 87.9 ± 3.7 and VAS 0.3 ±

Study	Region	Implant	Material	Manufacturing	Pillars	Tier	Patients	Analysed units	Headline result
PMID 41397797	um								0.5 at final follow-up; no aseptic loosening or screw breakage
Shash et al. 2025. Journal of materials science. Materials in medicine. PMID 41436650	chest_wall	rib reconstruction implant	multiple	not_manufactured	ai+comp	in_silico	0	8	AI model accuracy >99.9%; CFP60 cortical rib stress 40.68 MPa tensile and 58.5 MPa compressive under 1000 N lateral load
Elsharabasy et al. 2026. Journal of Dentistry. doi:10.1016/j.jdent.2026.106453	cmf	midface reconstruction plates	unclear	unclear	comp	clinical_series	78	78	identical superimposition between preoperative virtual planning and postoperative CT; dental rehabilitation 72/78 (92.3%); dental implants 68/78 (87.1%); removable prosthesis 4/78 (5.1%)
Ampaites et al. 2026. Results in Engineering. doi:10.1016/j.rineng.2026.110026	cmf	mandibular reconstruction plate with TMJ prosthesis	multiple	am_metal	comp	bench	0	.	FEA-experiment stiffness deviation <7%; peak plate and screw stresses reduced approximately 60%; fossa stress reduced up to 50%
Zhong et al. 2026. Virtual and Physical Prototyping. doi:10.1080/17452759.2026.2653335	cmf	mandibular reconstruction plate	titanium	am_metal	comp	cadaver_phantom	0	3	anatomical fit confirmed on 3 printed mandibular models; approximately 25% mass reduction with comparable structural safety and stability
Gürlek et al. 2026. Journal of Craniofacial Surgery. doi:10.1097/scs.0000000000013117	cmf	maxillary fixation plate	unclear	not_manufactured	comp	in_silico	0	1	single-piece PSP had the lowest displacement in all directions; screw stress 88.379 MPa and bone stress 14.835 MPa
Salem et al. 2026. IEEE Access. doi:10.1109/access.2026.3663618	cranial	cranial implant	unclear	unclear	ai	bench	0	40	mean Dice 0.901; Boundary Dice 0.908; HD95 1.52 mm
Fritz et al. 2026. Journal of Medical Devices. doi:10.1115/1.4071170	pelvis_acetabulum	acetabular revision implant	titanium	am_metal	comp	bench	0	.	contact-force/manufacturing-constrained implant had most native regional strain energy and low contact forces
Nuermaimaiti et al. 2026. . doi:10.21203/rs.3.rs-10388339/v1	knee	proximal tibial tumour endoprosthesis	multiple	multiple	imaging+comp	clinical_series	13	13	planned prosthesis implanted 13/13 without documented conversion; no progressive radiographic mechanical failure; implant retention 13/13 through 12–15 mo; manufacturing cycle approximately 1 week; MSTS-93 median 14 to 26; complications 2/13
Alshikh et al. 2026. . doi:10.21203/rs.3.rs-8841053/v1	cmf	maxillary ridge augmentation mesh	multiple	multiple	comp	rct	14	28	gained bone volume 499.47 ± 80.46 mm³ PEEK vs 370.82 ± 51.69 mm³ titanium (p = 0.004); graft loss 174.83 ± 40.78 mm³ vs 127.73 ± 35.55 mm³ (p = 0.040); mesh exposure 1/14 sites vs 2/14 sites

Study	Region	Implant	Material	Manufacturing	Pillars	Tier	Patients	Analysed units	Headline result
Etik et al. 2026. . doi:10.21203/rs.3.rs-8848013/v1	cmf	subperiosteal jaw implant	peek	not_manufactured	imaging+comp	in_silico	0	4	maximum implant von Mises stress 531.44 MPa at 1.0-mm implant/1.5-mm screw vs 451.81 MPa at 1.5-mm implant/2.0-mm screw under posterior oblique loading
Abdeldayem et al. 2026. . doi:10.21203/rs.3.rs-9337194/v1	cmf	mandibular reconstruction plate	unclear	unclear	imaging+comp	clinical_series	30	30	mean menton deviation 0.053 ± 0.266 (unit not reported); mean reconstruction time 59.13 ± 12.25 min; graft success 29/30; stable postoperative occlusion 28/30; plate loosening or removal 1/30
Haneef et al. 2026. International Journal of Applied Dental Sciences. doi:10.22271/oral.2026.v12.i6a.2425	cmf	maxillofacial defect implant	multiple	multiple	comp	clinical_series	10	10	satisfactory adaptation in 10/10; no significant bilateral differences (p>0.05); infection with implant exposure and removal 1/10; uncomplicated healing 9/10 at 12 mo
Nguyen et al. 2026. Applied Sciences. doi:10.3390/app16104763	cranial	cranioplasty plate	unclear	not_manufactured	ai	imaging_dataset	0	250	Dice 0.94; Hausdorff distance 3.8 mm; inference time 0.004 s per case
Ismailov et al. 2026. Zenodo (CERN European Organization for Nuclear Research). doi:10.5281/zenodo.18811308	cmf	pre-contoured mandibular reconstruction plate	titanium	unclear	comp	clinical_comparative	12	12	mean global 3D deviation 3.40 ± 1.02 mm; 3.55 ± 1.12 mm vs 3.29 ± 1.10 mm, P > 0.05; hardware destabilization 1/12
Axrorov et al. 2026. Zenodo (CERN European Organization for Nuclear Research). doi:10.5281/zenodo.19562530	cmf	mandibular reconstruction implant	titanium	am_metal	comp	clinical_series	32	32	implant survival 93.7%; improved mastication, speech, and facial symmetry
Gondra et al. 2026. . doi:10.64898/2026.04.02.26349771	cranial	cranioplasty implant	pmma	cast	imaging+comp	clinical_series	8	8	implant fitting satisfactory in 8/8 cases; design time ~1 h small defects to ~3 h large defects; printing time 2-3 h small defects to 8-10 h large defects; satisfactory aesthetic outcome 7/8 (87.5%); major implant-related complications 0/8; postoperative infections 0/8; implant removals 0/8
Guo et al. 2026. University of Alberta Library. doi:10.7939/83976	cranial	biomimetic cranial implant	unclear	am_polymer	ai+imaging+comp	bench	0	.	segmentation accuracy 94.7%; porosity error 3.2 ± 1.8%; elastic modulus error 4.1 ± 2.3%; compressive strength up to 950 MPa; elastic modulus 1.6–13.9 GPa
Mejía Rodríguez et al. 2026. The Journal of prosthetic dentistry. PMID 40930860	cmf	custom TMJ prosthesis	unclear	not_manufactured	comp	in_silico	0	2	curvature radii #3 and #4 reduced von Mises and minimum principal stresses by 50% and optimized translation by 45%
Oldhoff et al. 2026. Journal	pel	posterior wall	tit	machin	com	bench	0	10	fracture gapping 0.0–0.8

Study	Region	Implant	Material	Manufacturing	Pillars	Tier	Patients	Analysed units	Headline result
of orthopaedic research : official publication of the Orthopaedic Research Society. PMID 40985989	vis_acetabulum	acetabular fracture fixation plate	anium	ed	p				mm; median stiffness 1.75 kN/mm PSI vs 1.71 kN/mm conventional before cycling; peak implant stress 55 MPa vs 165 MPa at 3200 N
Tehli et al. 2026. Operative neurosurgery (Hagerstown, Md.). PMID 41090924	cranial	cranioplasty implant	titanium	am_metal	imaging+comp	clinical_series	33	33	complications 0/33; complete tumour removal and satisfactory cosmetic results verified on postoperative CT
Ngo et al. 2026. European spine journal : official publication of the European Spine Society, the European Spinal Deformity Society, and the European Section of the Cervical Spine Research Society. PMID 41094110	spine	patient-specific spinal rod	titanium	unclear	ai+comp	clinical_series	34	34	postoperative-minus-predicted lumbar lordosis -3.8° and pelvic tilt -0.1° at 2 years; median thoracic kyphosis gain 9.3° vs preoperative and 4.9° vs planned at 2 years
Zhong et al. 2026. Annals of biomedical engineering. PMID 41191275	cmf	mandibular reconstruction plate	titanium	multiple	comp	bench	0	2	PSMRP stiffness 300.8 N/mm versus 201.2 N/mm; PSMRP survived 2500 N and 860 cycles while manual-bent plate failed at 1800 N and 575 cycles
Wood-Bailey et al. 2026. Archives of oral biology. PMID 41218231	cmf	custom root-analogue dental implant	multiple	not_manufactured	comp	in_silico	0	6	custom implant tensile strain 7400 με and compressive strain 12000 με; bite reaction force 1268 N
Türk et al. 2026. Journal of stomatology, oral and maxillofacial surgery. PMID 41285248	cmf	Le Fort I patient-specific fixation plate	unclear	not_manufactured	comp	in_silico	0	4	PSI reduced stress magnitudes versus miniplates under 125 N loading
Zoabi et al. 2026. Journal of oral and maxillofacial surgery : official journal of the American Association of Oral and Maxillofacial Surgeons. PMID 41344382	cmf	orbital floor implant	unclear	unclear	imaging+comp	clinical_comparative	23	23	RMS planned-postoperative implant deviation 0.67 ± 0.22 mm mirroring vs 0.62 ± 0.24 mm virtual reduction; orbital volume 108.6% to 99.5% with mirroring and 107.3% to 101.1% with virtual reduction; diplopia resolved in most cases
Zouggar et al. 2026. Journal of the mechanical behavior of biomedical materials. PMID 41418444	cranial	cranial implant	multiple	am_polymer	comp	bench	0	2	dimensional deviation <5%; warpage prediction error <15%; PA12CF35 shrinkage 17.5% lower and residual stress 7.7% lower than PA12; solidification speed improved 26% with PA12CF35
Bayram et al. 2026. The Journal of craniofacial surgery. PMID 41499545	cmf	mandibular onlay implant	titanium	am_metal	imaging+comp	clinical_series	8	8	FAI significantly reduced at 12 mo; AES and RSE significantly improved at 12 mo; no early complications
Adell-Gómez et al. 2026. Journal of cranio-maxillo-facial surgery : official publication of the European Association for Cranio-Maxillo-Facial Surgery. PMID 41506104	cmf	orthognathic fixation plates	unclear	unclear	imaging+comp	clinical_comparative	90	90	significantly improved maxillary midline, mandibular midline, maxillary dental plane, and pogonion accuracy; $p < 0.05$; no significant difference in surgical time;

Study	Region	Implant	Material	Manufacturing	Pillars	Tier	Patients	Analysed units	Headline result
									mandibular plates replaced intraoperatively in 1/15 PSI patients
Chintalapudi et al. 2026. The Journal of craniofacial surgery. PMID 41524259	cmf	subperiosteal jaw implant	titanium	am_metal	comp	clinical_series	8	8	no infections, implant mobility, or inflammation; gingival retraction 1/8
Troise et al. 2026. Journal of stomatology, oral and maxillofacial surgery. PMID 41544967	cmf	orbital floor mesh	titanium	unclear	comp	clinical_comparative	52	52	implant malposition 0/24 vs 7/28 (25%); p = 0.011; median operative time 55 min [IQR 50–65] vs 110 min [IQR 98–125]; p < 0.001; persistent diplopia 1/24 (4%) vs 8/28 (28.6%); p = 0.028
Wang et al. 2026. Dental materials : official publication of the Academy of Dental Materials. PMID 41554658	cmf	biodegradable mandibular implant	multiple	am_polymer	comp	bench	0	2	RI-2 proof load 83.8 ± 5.3 N and bending strength 1466 ± 92 N·mm; RI-3 failed after 14.4 ± 1.2 d
Yang et al. 2026. Cancers. PMID 41595190	cmf	mandibular reconstruction plate	unclear	unclear	imaging+comp	clinical_comparative	111	111	mean resection-plane deviation 1.63 ± 1.42 mm; PSIs had less intergonial-distance and operated-side coronal-angle deviation; plate fracture 0% PSI vs 15.2% conventional with bone; median fabrication time 6.0 d; plate-related complications 43/110; plate exposure 24/110
Wüster et al. 2026. Oral and maxillofacial surgery. PMID 41634287	cmf	mandibular reconstruction plate	titanium	am_metal	imaging+comp	clinical_series	15	15	posterior segment deviation 6.78 ± 3.34 mm basal and 7.61 ± 3.40 mm crestal; anterior 3.54 ± 1.52 mm basal and 4.49 ± 2.51 mm crestal; plate exposure 3/15; non-union 3/10; no plate fracture or loosening
Kim et al. 2026. Korean journal of neurotrauma. PMID 41657642	cranial	cranioplasty flap	peek	machined	imaging	clinical_comparative	36	36	CSF collection 0/16 modified vs 2/20 control; hemorrhage 0/16 vs 2/20 control
Pulino et al. 2026. Craniomaxillofacial trauma & reconstruction. PMID 41725913	cmf	mandibular reconstruction plate	unclear	not_manufactured	comp	in_silico	0	3	peak plate stress 695.5 MPa conventional, 595.6 MPa grid-type, and 185.2 MPa customized
Thanh et al. 2026. Bioengineering (Basel, Switzerland). PMID 41749792	shoulder	proximal humerus endoprosthesis	peek	am_polymer	imaging+comp	clinical_series	7	7	maximum surface deviation <2 mm; mechanical implant survival 85.7%; fracture 1/7 at 28 mo; manufacturing time 7–14 d; MSTS 23.0 ± 1.6; complications 3/7; limb salvage 100%
Sun et al. 2026. Journal of cranio-maxillo-facial surgery : official publication of the European Association for Cranio-Maxillo-Facial Surgery. PMID 41759452	cmf	maxillary fixation plate	peek	not_manufactured	comp	in_silico	0	2	displacement 0.08416 mm PEEK vs 0.09285 mm titanium; screw stress 515.8 MPa vs 414.2 MPa
Ruf et al. 2026. Frontiers in bioengineering and biotechnology. PMID	cmf	mandibular fixation plates	titaniu	not_manufactu	comp	in_silico	0	4	pre-bent plates peak 305.44–374.38 MPa before biting; same-shaped patient-specific

Study	Region	Implant	Material	Manufacturing	Pillars	Tier	Patients	Analysed units	Headline result
41768128			m	red					plates below 60 MPa
Mäntynen et al. 2026. Maxillofacial plastic and reconstructive surgery. PMID 41779306	cmf	orthognathic fixation plate	unclear	unclear	imaging+comp	clinical_comparative	40	40	PSI fit 37/40 (92.5%); ULD vs standard-dose p = 0.238; reoperation for postoperative malocclusion 1/40
Do et al. 2026. World journal of surgical oncology. PMID 41795068	cmf	mandibular reconstruction plate	titanium	am_metal	comp	clinical_series	9	9	deformation 2.01 mm NFCP, 2.20 mm DFMP, and 1.45 mm modified DFMP; peak stress 683.3 MPa, 378.2 MPa, and 498.8 MPa; production time approximately 2–3 wk; plate fractures 2/9; screw loosening 1/9
De Cet et al. 2026. Medical & biological engineering & computing. PMID 41838281	spine	interbody fusion cage	ceramic	not_manufactured	comp	in_silico	0	7	microstructured HAp failed volume 0.28% ± 0.21%; system stiffness 10.35 ± 0.85 kN/mm
Ghorab et al. 2026. Oral and maxillofacial surgery. PMID 41840235	cmf	mandibular reconstruction plate	unclear	unclear	comp	clinical_series	10	10	mean overall linear deviation 1.92 mm; RMSE 2.11 mm; mean gonial angle deviation 1.66°
Xiao et al. 2026. Bone & joint research. PMID 41880204	upper_limb_other	total wrist prosthesis	multiple	am_metal	comp	in_silico	0	3	carpal platform and screws 65.27 MPa; radial shank 2.09 MPa; carpal bones 33.84 MPa
Oteiza et al. 2026. Craniomaxillofacial trauma & reconstruction. PMID 41892802	cmf	orbital reconstruction implant	multiple	multiple	imaging+comp	cadaver_phantom	0	18	fit score 2.00 ± 1.04 PEEK vs 2.18 ± 0.60 titanium on 1–5 Likert scale (1 = very good); mechanical stability score 1.67 ± 0.49 PEEK vs 1.91 ± 0.54 titanium; stable ratings 100% vs 91%; procedure time 9.5 ± 5.3 min PEEK vs 11.2 ± 5.1 min titanium
Liu et al. 2026. BMC musculoskeletal disorders. PMID 41943026	knee	peri-knee defect prosthesis	titanium	am_metal	comp	clinical_series	10	10	stress concentrated in intramedullary nails, prosthesis lateral flanks, and tibial insert grooves under 1764 N; osteomyelitis recurrence with transfemoral amputation 2/10; screw breakage with revision 1/10; LEFS 20.1 vs 8.9, P < 0.001 in remaining 8
Benady et al. 2026. Bone & joint open. PMID 41956475	lower_limb_other	critical-sized defect lattice implant	titanium	am_metal	imaging+comp	clinical_series	29	29	early complications 3/29; late complications 4/29; limb salvage 27/29; median MSTS 80% (IQR 60% to 87%)
Zhang et al. 2026. Journal of orthopaedic translation. PMID 42006909	spine	intervertebral disc implant	polymer_	am_polymer	comp	bench	0	6	G65N-implanted FSU stress 0.00009–26.600 MPa under compression and 0.00067–54.090 MPa under flexion;

Study	Region	Implant	Material	Manufacturing	Pillars	Tier	Patients	Analysed units	Headline result
			other						endplate pressure 2.59 MPa; axial displacement 1.86 mm
Etik et al. 2026. Scientific reports. PMID 42032041	cmf	subperiosteal jaw implant	peek	not_manufactured	comp	in_silico	0	4	peak implant von Mises stress 531.44 MPa for 1.0 mm framework/1.5 mm screw under posterior oblique loading; maximum displacement 0.0381 mm
Zhang et al. 2026. Scientific reports. PMID 42045386	cmf	mandibular reconstruction implant	titanium	am_metal	comp	bench	0	3	peak tensile strain 871.67 ± 55.94 με solid vs 787.32 ± 6.64 με QDC and 779.98 ± 11.02 με Hex-vase; peak compressive strain −795.85 ± 12.76 με Hex-vase
Prikule et al. 2026. Journal of clinical medicine. PMID 42074623	cmf	orbital floor mesh	titanium	unclear	imaging+comp	clinical_comparative	74	74	postoperative overcorrection mean 1479 mm3 control vs 1531 mm3 3D; undercorrection 1921 mm3 vs 853 mm3; reoperation 5/34 control vs 0/40 3D, p = 0.017; persistent diplopia 4/34 vs 1/40 at 6 mo
Kim et al. 2026. Journal of clinical medicine. PMID 42123027	cmf	chin augmentation implant	pmma	cast	imaging+comp	clinical_series	20	20	mean advancement discrepancy 1.02 mm (planned 5.04 mm vs actual 4.02 mm); r = 0.928, p = 0.001; GAIS exceptional or very improved 18/20; transient numbness 2/20 resolved within 2 mo; no major complications
Chandra et al. 2026. Journal of cranio-maxillo-facial surgery : official publication of the European Association for Cranio-Maxillo-Facial Surgery. PMID 42160799	cmf	TMJ implant	unclear	not_manufactured	comp	in_silico	0	15	simplified models reduced maximum principal bone strain up to 50% and implant von Mises stress up to 44%
Chen et al. 2026. Orthopaedic surgery. PMID 42165180	spine	vertebral body replacement prosthesis	titanium	not_manufactured	comp	in_silico	0	3	peak prosthesis von Mises stress 316.7 MPa with artificial pedicle, 182.5 MPa stand-alone, and 638.6 MPa titanium mesh cage
Sharma et al. 2026. Proceedings of the Institution of Mechanical Engineers. Part H, Journal of engineering in medicine. PMID 42169235	knee	tibial insert	polymer_other	unclear	comp	bench	0	.	surface finish improved about 80%; Ra 26.87 μm, Rq 33.58 μm, Rsk -1.19, Rku 3.12
El Morsy et al. 2026. The Journal of craniofacial surgery. PMID 42183622	cmf	orbital floor implant	multiple	unclear	imaging+comp	rct	24	24	no significant between-group differences in orbital volume restoration or reconstruction accuracy; complications 3 in titanium group and 0 in zirconia group
Du et al. 2026. North American Spine Society journal. PMID 42199851	spine	interbody cage	titanium	am_metal	comp	cadaver_phantom	0	16	median failure force 876.5 N vs 443.7 N; 97.57% increase; p = .03

Study	Region	Implant	Material	Manufacturing	Pillars	Tier	Patients	Analysed units	Headline result
Choudhary et al. 2026. Journal of materials science. Materials in medicine. PMID 42262441	cranial	cranial implant	unclear	unclear	comp	bench	0	9	minimum dimensional deviation 5.41%; Vickers microhardness 64.66 to 81 N/mm²; 20.17% increase
Ead et al. 2026. Clinical biomechanics (Bristol, Avon). PMID 42302371	lower_limb_other	total talus implant	multiple	not_manufactured	comp	in_silico	0	4	cobalt chrome implants had higher peak contact pressures and smaller contact areas than native talus; PCU-coated implants more closely matched native models
Ebrahimzadeh Dehaghani et al. 2026. Computers in biology and medicine. PMID 42308584	hip	paediatric proximal femoral osteotomy plate	titanium	not_manufactured	comp	in_silico	0	1	distal cortical strain energy density increased 25% vs solid plates; all implants below yield-strength range with micromotion within threshold
Yáñez González-Cuéllar et al. 2026. Computer methods and programs in biomedicine. PMID 42320193	hip	short femoral stem	unclear	not_manufactured	ai+comp	in_silico	0	392	mean absolute error reduced approximately 30% vs random forest
Aguilar et al. 2026. Craniomaxillofacial trauma & reconstruction. PMID 42346039	cmf	customized maxillary fixation plate	titanium	am_metal	comp	clinical_comparative	64	64	median landmark displacement 0.7-4.28 mm; 18/64 had displacement ≥2.0 mm; instability 28.6% minimally invasive vs 33.3% conventional; OR 1.23, 95% CI 0.32-4.65
Juneja et al. 2026. Bioengineering (Basel, Switzerland). PMID 42351864	cmf	maxillary-zygomatic defect implant	peek	am_polymer	ai+comp	bench	0	2377	DSC 0.778; HD95 3.453 mm; peak von Mises stress 386.13 MPa at 400 N; fatigue life 4.7 million cycles at 100 N; inference 0.060 s/volume; manual reinforcement under 2 min
Catarzi et al. 2026. Medicina (Kaunas, Lithuania). PMID 42356056	cmf	two-piece orbital implant	titanium	am_metal	imaging+comp	clinical_series	17	17	mean implant deviation 0.982 ± 0.107 mm; all implants within 1.5 mm; diplopia resolved 17/17; enophthalmos fully corrected 15/17; no major complications or revisions
Abu-Sirhan et al. 2026. Journal of cranio-maxillo-facial surgery : official publication of the European Association for Cranio-Maxillo-Facial Surgery. PMID 42413458	cmf	maxillary osteosynthesis plates	unclear	unclear	comp	clinical_series	63	63	transfer accuracy within ±1-2 mm at all landmarks; postoperative complications were rare and managed conservatively
Elter et al. 2026. Journal of cranio-maxillo-facial surgery : official publication of the European Association for Cranio-Maxillo-Facial Surgery. PMID 42418929	cmf	frontofacial fixation plates	unclear	unclear	comp	clinical_series	8	8	mean deviations 1.58-1.73 mm for frontal and 1.15-1.30 mm for maxillary segments; mean operative time 484.9 ± 84.2 min; tracheostomy dependency decreased from 50% to 25%; no major complications
Tang et al. 2026. BMC oral health. PMID 42437915	cmf	combined intraosseous-subperiosteal jaw	titaniu	not_manufactured	comp	in_silico	0	4	six-abutment design reduced peak screw von Mises stress by approximately 33% and

Study	Region	Implant	Material	Manufacturing	Pillars	Tier	Patients	Analysed units	Headline result
		implant	m						implant stress by 25.5% versus ultra-short implants
Austin et al. 2026. International journal of oral and maxillofacial surgery. PMID 42448534	cmf	spheno-orbital reconstruction implant	peek	unclear	imaging+comp	clinical_series	13	13	mean Hertel difference 4.3 mm preoperatively vs 2.1 mm postoperatively; mean orbital volume difference 1.9 cm3 vs 2.4 cm3; symptomatic proptosis or diplopia improved in 9/10
Abdeldayem et al. 2026. Maxillofacial plastic and reconstructive surgery. PMID 42479267	cmf	mandibular reconstruction plate	titanium	machined	imaging+comp	clinical_series	30	30	menton discrepancy 0.053 ± 0.266 mm; overall asymmetry index 41.91 ± 5.32 mm preoperatively vs 6.48 ± 2.46 mm postoperatively; stable occlusion 28/30; graft survival 29/30; plate loosening requiring removal 1/30
Riad et al. 2026. BMC oral health. PMID 42482012	cmf	mandibular fracture fixation plate	titanium	machined	imaging+comp	clinical_series	6	6	patient-level MAE 0.48 mm, SD 0.21 mm, range 0.25-0.81 mm; no infection, plate exposure, non-union, malunion, hardware fracture, persistent malocclusion, neurosensory disturbance, or reoperation in 6/6
Abdellatif et al. 2026. BMC oral health. PMID 42498926	cmf	mandibular fracture fixation plates	titanium	machined	imaging+comp	rct	26	26	bony landmark deviation 0.044 ± 0.14 mm vs 0.69 ± 0.50 mm; dental landmark deviation 0.04 ± 0.13 mm vs 0.47 ± 0.33 mm; surgical time 1.46 ± 0.18 h vs 1.80 ± 0.34 h; wound dehiscence 0/13 vs 1/13; immediate occlusal disharmony 0/13 vs 2/13
Blatt et al. 2026. Journal of cranio-maxillo-facial surgery : official publication of the European Association for Cranio-Maxillo-Facial Surgery. PMID 42551059	cmf	mandibular reconstruction plate	unclear	unclear	comp	in_silico	0	12	normalized combined maximum shear and von Mises stress median 0.8, maximum 0.95, IQR 0.66-0.91
Rouhiainen et al. 2026. JPRAS open. PMID 42565001	cmf	maxillary fixation plate	unclear	machined	comp	clinical_comparative	59	59	horizontal relapse 0.28 mm vs 0.90 mm; vertical relapse 0.81 mm vs 1.60 mm
Abd Elfattah et al. 2026. BMC oral health. PMID 42568091	cmf	mandibular angle fracture plate	titanium	not_manufactured	comp	in_silico	0	2	plate von Mises stress 326.19 MPa vs 417.48 MPa; cortical principal stress 121.03 MPa vs 87.759 MPa; displacement 157.32 µm vs 131.47 µm
De Oliveira Gorla et al. 2026. The British journal of oral & maxillofacial surgery. PMID 42608239	cmf	orbital implant	peek	unclear	comp	clinical_comparative	10	10	planned-treated volume discrepancy median 1.50 cm3 navigation vs 0.20 cm3 freehand; translational deviations <2 mm; design and manufacturing within 4 weeks; clinical success 6/9; persistent diplopia 3/9
Bertram et al. 2026. BMC Oral Health. PMID	cmf	patient-specific maxillary	titani	am_metal	imaging+	clinical_comp	26	26	mean translational deviation 0.46 ± 0.33 mm vs 0.79 ±

Study	Re gi on	Implant	M ate ria l	Manufa cturing	Pilla rs	Tier	Pa tie nts	Analy sed units	Headline result
42638112		osteosynthesis plate	u m		com p	arative			0.58 mm; rotational deviation 0.82° ± 0.91° vs 1.24° ± 0.85°; operating time 152.1 ± 15.26 min vs 142.1 ± 16.31 min (p = 0.1739)
Smeets et al. 2026. International journal of oral and maxillofacial surgery. PMID 42674940	cm f	chin plate and β-TCP gap implant	m ult ipl e	unclear	imag ing+ com p	clinical _series	10	10	mean translational deviations <0.5 mm; rotational discrepancies <1° except β-TCP implant pitch 1.7°
Carlos et al. 2026. Digital Repository (Polytechnic University of Cartagena). Design, optimization, and virtual validation of personalized mandibular prosthes	cm f	mandibular osteosynthesis plate	tit ani u m	not_ma nufactu red	ai+i magi ng+c omp	in_silic o	0	.	Pseudo Dice near 1; maximum von Mises stress below material yield strength; maximum displacement below maximum-load conditions

Table S2. Studies excluded at assessment

ID	Study	Code	Quote
doi:10.1007/s12663-023-01950-3	Manekar et al. 2023. Journal of Maxillofacial and Oral Surgery. doi:10.1007/s12663-023-01950-3	duplicate_report	EXCLUDE: same report as pmid:37534346 (second identifier); pmid:37534346 is the charted copy
doi:10.1007/s13770-025-00730-z	Choi et al. 2025. Tissue Engineering and Regenerative Medicine. doi:10.1007/s13770-025-00730-z	duplicate_report	EXCLUDE: same report as pmid:40512299 (second identifier); pmid:40512299 is the charted copy
doi:10.1016/j.dental.2024.07.008	Oberoi et al. 2024. Dental Materials. doi:10.1016/j.dental.2024.07.008	duplicate_report	EXCLUDE: same report as pmid:39084954 (second identifier); pmid:39084954 is the charted copy
doi:10.1016/j.ijom.2021.05.011	De Moor et al. 2022. International Journal of Oral and Maxillofacial Surgery. doi:10.1016/j.ijom.2021.05.011	duplicate_report	EXCLUDE: same report as pmid:34059405 (second identifier); pmid:34059405 is the charted copy
doi:10.1016/j.jcms.2015.10.004	Pinheiro et al. 2015. Journal of Cranio-Maxillofacial Surgery. doi:10.1016/j.jcms.2015.10.004	duplicate_report	EXCLUDE: same report as pmid:26596361 (second identifier); pmid:26596361 is the charted copy
doi:10.1016/j.jmbbm.2020.103700	Li et al. 2020. Journal of the Mechanical Behavior of Biomedical Materials. doi:10.1016/j.jmbbm.2020.103700	duplicate_report	EXCLUDE: same report as pmid:32279847 (second identifier); pmid:32279847 is the charted copy
doi:10.1016/j.medengphy.2025.104321	Dunbar et al. 2025. Medical Engineering & Physics. doi:10.1016/j.medengphy.2025.104321	duplicate_report	EXCLUDE: same report as pmid:40180533 (second identifier); pmid:40180533 is the charted copy
doi:10.1016/j.procir.2024.08.047	Xu et al. 2024. Procedia CIRP. doi:10.1016/j.procir.2024.08.047	not_psi	EXCLUDE: This paper explores the design of orthopaedic bone plates using Generative Design (GD) and Topological Optimization (TO), aimed at eventual production through DED.
doi:10.1016/j.prosdent.2026.02.038	Kohansal et al. 2026. The Journal of Prosthetic Dentistry. doi:10.1016/j.prosdent.2026.02.038	duplicate_report	EXCLUDE: same report as pmid:42020225 (second identifier); pmid:42020225 is the charted copy
doi:10.1016/j.wneu.2021.01.102	Huys et al. 2021. World Neurosurgery. doi:10.1016/j.wneu.2021.01.102	duplicate_report	EXCLUDE: same report as pmid:33529765 (second identifier); pmid:33529765 is the charted copy
doi:10.1038/s41597-021-00806-0	Li et al. 2021. Scientific Data. doi:10.1038/s41597-021-00806-0	no_evaluation	EXCLUDE: The dataset introduced in this study contains 240 data pairs and can be used for training deep learning models for automatic cranial implant design.
doi:10.1038/s41598-021-94980-1	Carnicero et al. 2021. Scientific Reports. doi:10.1038/s41598-021-94980-1	duplicate_report	EXCLUDE: same report as pmid:34321582 (second identifier); pmid:34321582 is the charted copy
doi:10.1055/s-0034-1395385	Aquilina et al. 2015. Craniomaxillofacial Trauma & Reconstruction. doi:10.1055/s-0034-1395385	duplicate_report	EXCLUDE: same report as pmid:26000081 (second identifier); pmid:26000081 is the charted copy
doi:10.1055/s-0037-1600868	Aghdasi et al. 2017. Journal of Neurological Surgery Part B Skull Base. doi:10.1055/s-0037-1600868	not_psi	EXCLUDE: This technology is expected to facilitate pre-surgical planning for skull base surgery and allow integration into navigation guidance systems.
doi:10.1055/s-0043-1767095	Matin-Mann et al. 2023. Laryngo-Rhino-Otologie. doi:10.1055/s-0043-1767095	not_skeletal	EXCLUDE: The aim of this study was to validate our semi-automated segmentation and implant design approach, of the round window niche (RWN) and the round window membrane (RWM) for use in the development of patient individualized round window niche implants (RNI).
doi:10.1097/gox.0000000000004081	Kittichokechai et al. 2022. Plastic and Reconstructive Surgery - Global Open. doi:10.1097/gox.0000000000004081	duplicate_report	EXCLUDE: same report as pmid:35186634 (second identifier); pmid:35186634 is the charted copy
doi:10.1097/scs.0000000000012936	El Morsy et al. 2026. Journal of Craniofacial Surgery. doi:10.1097/scs.0000000000012936	duplicate_report	EXCLUDE: same report as pmid:42183622 (second identifier); pmid:42183622 is the charted copy

ID	Study	Code	Quote
doi:10.1101/2021.07.22.21260825	Dot et al. 2021. . doi:10.1101/2021.07.22.21260825	duplicate_preprint	EXCLUDE: same report as pmid:35037088 (preprint, repository or version copy); pmid:35037088 is the charted copy
doi:10.1101/2025.04.28.651047	Chandra et al. 2025. . doi:10.1101/2025.04.28.651047	not_psi	EXCLUDE: For each intact mandible model (three models: Model 1, Model 2 and Model 3), corresponding implanted models were developed with two types of stock TMJ implants (Narrow and Standard).
doi:10.1109/embc48229.2022.9871069	Sulakhe et al. 2022. 2022 44th Annual International Conference of the IEEE Engineering in Medicine & Biology Society (EMBC). doi:10.1109/embc48229.2022.9871069	duplicate_report	EXCLUDE: same report as pmid:36085744 (second identifier); pmid:36085744 is the charted copy
doi:10.1109/iccvw60793.2023.00114	Kwarciak et al. 2023. . doi:10.1109/iccvw60793.2023.00114	no_method	EXCLUDE: The generated skulls may improve the automatic design of personalized cranial implants for real medical cases.
doi:10.1109/tiptekno68206.2025.11270164	Onat et al. 2025. . doi:10.1109/tiptekno68206.2025.11270164	no_method	EXCLUDE: The proposed approach shows strong potential for integration into clinical workflows, providing high-fidelity 3D reconstructions suitable for patient-specific cranial implant manufacturing.
doi:10.1115/1.4062509	Choudhury et al. 2023. Journal of Engineering and Science in Medical Diagnostics and Therapy. doi:10.1115/1.4062509	not_psi	EXCLUDE: A mandible model is chosen with a diagonal length of 82 mm and created perpendicular fracture gaps measuring 1 mm to 3 mm on the left buccolingual section of the solid model.
doi:10.11159/mvml25.134	Durrani et al. 2025. Proceedings of the World Congress on Electrical Engineering and Computer Systems and Science. doi:10.11159/mvml25.134	no_evaluation	EXCLUDE: These models and optimization loops will be implemented in future phases of the research.
doi:10.1117/12.2580719	Li et al. 2021. . doi:10.1117/12.2580719	no_evaluation	EXCLUDE: We introduce a fully automatic system for cranial implant design, a common task in cranioplasty operations.
doi:10.1155/2016/8242535	Anssari Moin et al. 2016. International Journal of Dentistry. doi:10.1155/2016/8242535	duplicate_report	EXCLUDE: same report as pmid:27274727 (second identifier); pmid:27274727 is the charted copy
doi:10.1177/09544119221114729	Sandeep et al. 2022. Proceedings of the Institution of Mechanical Engineers, Part H: Journal of Engineering in Medicine. doi:10.1177/09544119221114729	duplicate_report	EXCLUDE: same report as pmid:35880901 (second identifier); pmid:35880901 is the charted copy
doi:10.1186/s41205-025-00260-3	Nayak et al. 2025. 3D Printing in Medicine. doi:10.1186/s41205-025-00260-3	duplicate_report	EXCLUDE: same report as pmid:40067641 (second identifier); pmid:40067641 is the charted copy
doi:10.1302/2046-3758.83.bjr-2018-0193.r1	Koh et al. 2019. Bone & Joint Research. doi:10.1302/2046-3758.83.bjr-2018-0193.r1	duplicate_report	EXCLUDE: same report as pmid:30997041 (second identifier); pmid:30997041 is the charted copy
doi:10.1371/journal.pone.0325587.t001	Aziliz et al. 2025. Figshare. doi:10.1371/journal.pone.0325587.t001	source_type	EXCLUDE: Segmentation accuracy.
doi:10.1371/journal.pone.0325587.t003	Aziliz et al. 2025. Figshare. doi:10.1371/journal.pone.0325587.t003	source_type	EXCLUDE: Full bone reconstruction accuracy.
doi:10.1515/bmt-2023-0017	Grothues et al. 2023. Biomedical Engineering / Biomedizinische Technik. doi:10.1515/bmt-2023-0017	duplicate_report	EXCLUDE: same report as pmid:37185164 (second identifier); pmid:37185164 is the charted copy
doi:10.1515/cdbme-2025-0137	Schnabel et al. 2025. Current Directions in Biomedical Engineering. doi:10.1515/cdbme-2025-0137	no_method	EXCLUDE: The enhanced resolution facilitates more accurate geometric extraction and material mapping for subsequent finite element analyses.
doi:10.17683/ijomam/issue22.v2.2	Anonymous et al. 2025. International Journal of Mechatronics and Applied Mechanics. doi:10.17683/ijomam/issue22.v2.2	case_report	EXCLUDE: Within this study, two typical clinical cases with complexity in design and development of patient-specific implants were presented, with successful outcomes and enhanced quality of medical treatments for complex bone reconstruction.

ID	Study	Code	Quote
doi:10.17918/00010717	Barla et al. 2024. . doi:10.17918/00010717	not_psi	EXCLUDE: The project’s deliverables include a detailed statistical shape model and design envelopes representing pelvic shapes across percentiles, serving as valuable resources for researchers, engineers, and surgeons.
doi:10.18063/ijb.v8i1.437	Lin et al. 2021. International Journal of Bioprinting. doi:10.18063/ijb.v8i1.437	duplicate_report	EXCLUDE: same report as pmid:35187275 (second identifier); pmid:35187275 is the charted copy
doi:10.18260/1-2–42175	DeCarvalho et al. 2024. . doi:10.18260/1-2–42175	not_psi	EXCLUDE: This paper will look at studying a femur from a singular patient to analyze the possibility of creating an implant using additive manufacturing.
doi:10.18502/keg.v2i2.602	Mohammed et al. 2017. KnE Engineering. doi:10.18502/keg.v2i2.602	no_evaluation	EXCLUDE: The size and geometric precision of the final implant was also evaluated by reference to a FDM fabricated representative model of the patients surrounding skeletal anatomy and found to be an excellent fit.
doi:10.20944/preprints202107.0110.v1	Sharma et al. 2021. . doi:10.20944/preprints202107.0110.v1	duplicate_preprint	EXCLUDE: same report as pmid:34441859 (preprint, repository or version copy); pmid:34441859 is the charted copy
doi:10.20944/preprints202207.0017.v1	Dinu et al. 2022. . doi:10.20944/preprints202207.0017.v1	no_evaluation	EXCLUDE: The aim of this paper was to evaluate the orbital soft tissue after postraumatic reconstruction of the orbital walls fractures.
doi:10.20944/preprints202307.1391.v1	Moncayo-Matute et al. 2023. . doi:10.20944/preprints202307.1391.v1	case_report	EXCLUDE: The article reports on a patient who required a cranial protection system.
doi:10.20944/preprints202407.0500.v1	Moya et al. 2024. . doi:10.20944/preprints202407.0500.v1	duplicate_preprint	EXCLUDE: same report as doi:10.3390/technologies12080125 (preprint, repository or version copy); doi:10.3390/technologies12080125 is the charted copy
doi:10.20944/preprints202501.2283.v1	Andres et al. 2025. . doi:10.20944/preprints202501.2283.v1	duplicate_preprint	EXCLUDE: same report as pmid:40428165 (preprint, repository or version copy); pmid:40428165 is the charted copy
doi:10.21203/rs.3.pex-1384/v1	Bertani et al. 2021. . doi:10.21203/rs.3.pex-1384/v1	source_type	EXCLUDE: We present a detailed step-by-step approach for the low-cost production and surgical implantation of cranial prostheses, aimed at restoring aesthetics, cerebral protection, and facilitating neurological rehabilitation.
doi:10.21203/rs.3.rs-1290007/v1	Min et al. 2022. . doi:10.21203/rs.3.rs-1290007/v1	duplicate_preprint	EXCLUDE: same report as pmid:35264178 (preprint, repository or version copy); pmid:35264178 is the charted copy
doi:10.21203/rs.3.rs-145748/v1	Badiali et al. 2021. . doi:10.21203/rs.3.rs-145748/v1	duplicate_preprint	EXCLUDE: same report as pmid:34834588 (preprint, repository or version copy); pmid:34834588 is the charted copy
doi:10.21203/rs.3.rs-1590857/v1	Fernandes et al. 2022. . doi:10.21203/rs.3.rs-1590857/v1	duplicate_preprint	EXCLUDE: same report as pmid:35869276 (preprint, repository or version copy); pmid:35869276 is the charted copy
doi:10.21203/rs.3.rs-262939/v1	Lin et al. 2021. . doi:10.21203/rs.3.rs-262939/v1	duplicate_preprint	EXCLUDE: same report as pmid:34521397 (preprint, repository or version copy); pmid:34521397 is the charted copy
doi:10.21203/rs.3.rs-3280673/v1	Xue et al. 2023. . doi:10.21203/rs.3.rs-3280673/v1	duplicate_preprint	EXCLUDE: same report as pmid:38233785 (preprint, repository or version copy); pmid:38233785 is the charted copy
doi:10.21203/rs.3.rs-3286588/v1	Ayhan et al. 2023. . doi:10.21203/rs.3.rs-3286588/v1	not_psi	EXCLUDE: This regenerated 3D model is based on all the mean values of the 11 patients’ data and contains all anatomically relevant points.
doi:10.21203/rs.3.rs-3297532/v1	Telha et al. 2023. . doi:10.21203/rs.3.rs-3297532/v1	not_psi	EXCLUDE: Both fixation mini plates models had the same conventional 4-hole L-shape design with similar thicknesses of 0.5 mm and

ID	Study	Code	Quote
			0.7 mm and placement vectors at the piriform rims and the zygomaticomaxillary buttress, respectively.
doi:10.21203/rs.3.rs-4145453/v1	Jakimiuk et al. 2024. . doi:10.21203/rs.3.rs-4145453/v1	duplicate_preprint	EXCLUDE: same report as pmid:39693002 (preprint, repository or version copy); pmid:39693002 is the charted copy
doi:10.21203/rs.3.rs-4160020/v1	Sun et al. 2024. . doi:10.21203/rs.3.rs-4160020/v1	duplicate_preprint	EXCLUDE: same report as pmid:39242632 (preprint, repository or version copy); pmid:39242632 is the charted copy
doi:10.21203/rs.3.rs-4241140/v1	Sun et al. 2024. . doi:10.21203/rs.3.rs-4241140/v1	duplicate_preprint	EXCLUDE: same report as pmid:39242632 (preprint, repository or version copy); pmid:39242632 is the charted copy
doi:10.21203/rs.3.rs-7535092/v1	Mei et al. 2025. . doi:10.21203/rs.3.rs-7535092/v1	not_psi	EXCLUDE: Based on the extent of resection, replacement materials (TMC/3D-PAVB) of appropriate dimensions were selected and integrated, with a minor quantity of autologous cancellous bone fragments strategically implanted into the intervertebral space to augment the fusion.
doi:10.21203/rs.3.rs-858174/v1	LEUNG et al. 2021. . doi:10.21203/rs.3.rs-858174/v1	duplicate_preprint	EXCLUDE: same report as pmid:36401069 (preprint, repository or version copy); pmid:36401069 is the charted copy
doi:10.21203/rs.3.rs-94887/v2	Wang et al. 2021. . doi:10.21203/rs.3.rs-94887/v2	duplicate_preprint	EXCLUDE: same report as pmid:33761991 (preprint, repository or version copy); pmid:33761991 is the charted copy
doi:10.2139/ssrn.4484792	Moncayo-Matute et al. 2023. SSRN Electronic Journal. doi:10.2139/ssrn.4484792	duplicate_preprint	EXCLUDE: same report as pmid:37562162 (preprint, repository or version copy); pmid:37562162 is the charted copy
doi:10.2139/ssrn.4499911	Singh et al. 2023. SSRN Electronic Journal. doi:10.2139/ssrn.4499911	duplicate_preprint	EXCLUDE: same report as pmid:37544200 (preprint, repository or version copy); pmid:37544200 is the charted copy
doi:10.21597/jist.1159127	ŞENSOY et al. 2022. Iğdır Üniversitesi Fen Bilimleri Enstitüsü Dergisi. doi:10.21597/jist.1159127	not_psi	EXCLUDE: A previously created Computer-Aided Design (CAD) of virtual spinal surgery model was used for the FEA studies conducted within the scope of the current study.
doi:10.25258/ijddt.16.11s.78	Tiwari et al. 2026. International Journal of Drug Delivery Technology. doi:10.25258/ijddt.16.11s.78	not_psi	EXCLUDE: Future studies can benefit from the utilization of individual anatomical features.
doi:10.26041/fhnw-14094	Maintz et al. 2024. Institutional Repository (IHS Vienna). doi:10.26041/fhnw-14094	duplicate_preprint	EXCLUDE: same report as pmid:39671058 (preprint, repository or version copy); pmid:39671058 is the charted copy
doi:10.26226/morressier.5c8f90a0b5d368000a26b6c7	Garbarino et al. 2019. . doi:10.26226/morressier.5c8f90a0b5d368000a26b6c7	no_method	EXCLUDE: In this research, a general framework is presented for automatic extraction of the pelvic anatomical coordinate system (CS) and bony landmarks from patientu2019s computed-tomography (CT) data.
doi:10.31661/jbpe.v0i0.2504-1912	Montazeriani et al. 2026. Journal of Biomedical Physics and Engineering. doi:10.31661/jbpe.v0i0.2504-1912	not_psi	EXCLUDE: This study aimed to develop an automated deep learning-based method for CT image reconstruction of zygomatic fractures to support surgical planning and navigation.
doi:10.33612/diss.1135250381	Wel et al. 2024. . doi:10.33612/diss.1135250381	source_type	EXCLUDE: This thesis presents new research aimed at improving Virtual Surgical Planning (VSP) for orthognathic surgery, with a focus on enhancing accuracy and streamlining the virtual workflow.
doi:10.33612/diss.1426903730	Oldhoff et al. 2025. . doi:10.33612/diss.1426903730	source_type	EXCLUDE: This dissertation investigated the application of patient-specific implants (PSIs) in trauma surgery.
doi:10.33612/diss.231382708	Meesters et al. 2022. . doi:10.33612/diss.231382708	source_type	EXCLUDE: Proefschrift ter verkrijging van de graad van doctor aan de Rijksuniversiteit Groningen op gezag van de rector magnificus prof. dr. E. Sterken en volgens besluit van het

ID	Study	Code	Quote
			College voor Promoties.
doi:10.33612/diss.835211514	Merema et al. 2023. . doi:10.33612/diss.835211514	source_type	EXCLUDE: This thesis focuses on the development, implementation, evaluation, and optimization of 3D-virtual surgical planning (VSP) workflows and patient specific implants (PSI) for oral and maxillofacial surgery (OMFS).
doi:10.3390/app142210396	Zhu et al. 2024. Applied Sciences. doi:10.3390/app142210396	no_method	EXCLUDE: This study investigated the influence of muscle forces on FE predictions of fixation screw pullout and fatigue failure in a custom pelvic implant.
doi:10.3390/cryst15070650	Alkentar et al. 2025. Crystals. doi:10.3390/cryst15070650	not_psi	EXCLUDE: This database was created with the intent of providing a partially patient-specific solution, taking into account individual factors such as the patient's body weight.
doi:10.3390/ma15124342	Ingawale et al. 2022. Materials. doi:10.3390/ma15124342	duplicate_report	EXCLUDE: same report as pmid:35744401 (second identifier); pmid:35744401 is the charted copy
doi:10.3390/ma16010398	Soloviev et al. 2023. Materials. doi:10.3390/ma16010398	duplicate_report	EXCLUDE: same report as pmid:36614737 (second identifier); pmid:36614737 is the charted copy
doi:10.3390/ma18214822	Yüceer-Çetiner et al. 2025. Materials. doi:10.3390/ma18214822	duplicate_report	EXCLUDE: same report as pmid:41227783 (second identifier); pmid:41227783 is the charted copy
doi:10.3390/polym15173620	Moncayo-Matute et al. 2023. Polymers. doi:10.3390/polym15173620	duplicate_report	EXCLUDE: same report as pmid:37688247 (second identifier); pmid:37688247 is the charted copy
doi:10.36922/ijb025400410	Xu et al. 2025. International Journal of Bioprinting. doi:10.36922/ijb025400410	not_psi	EXCLUDE: For this study, the mean tibia shape model was exported in Standard Tessellation Language (STL) format and imported into Autodesk Fusion 360 v2.0 (Autodesk Inc., United States of America).
doi:10.4055/cios25232	Lee et al. 2026. Clinics in Orthopedic Surgery. doi:10.4055/cios25232	duplicate_report	EXCLUDE: same report as pmid:41938871 (second identifier); pmid:41938871 is the charted copy
doi:10.4103/njms.njms_155_23	Datarkar et al. 2024. National Journal of Maxillofacial Surgery. doi:10.4103/njms.njms_155_23	duplicate_report	EXCLUDE: same report as pmid:39234141 (second identifier); pmid:39234141 is the charted copy
doi:10.4317/medoral.27065	Akkoyun et al. 2025. Medicina Oral Patología Oral y Cirugia Bucal. doi:10.4317/medoral.27065	duplicate_report	EXCLUDE: same report as pmid:40121675 (second identifier); pmid:40121675 is the charted copy
doi:10.48550/arxiv.2006.00980	Li et al. 2020. arXiv (Cornell University). doi:10.48550/arxiv.2006.00980	duplicate_preprint	EXCLUDE: same report as doi:10.1117/12.2580719 (preprint, repository or version copy); doi:10.1117/12.2580719 is the charted copy
doi:10.48550/arxiv.2204.06310	Marek et al. 2022. arXiv (Cornell University). doi:10.48550/arxiv.2204.06310	duplicate_preprint	EXCLUDE: same report as pmid:36257198 (preprint, repository or version copy); pmid:36257198 is the charted copy
doi:10.48550/arxiv.2308.04883	Kwarciak et al. 2023. arXiv (Cornell University). doi:10.48550/arxiv.2308.04883	duplicate_preprint	EXCLUDE: same report as doi:10.1109/iccvw60793.2023.00114 (preprint, repository or version copy); doi:10.1109/iccvw60793.2023.00114 is the charted copy
doi:10.48550/arxiv.2406.06372	Marek et al. 2024. arXiv (Cornell University). doi:10.48550/arxiv.2406.06372	duplicate_preprint	EXCLUDE: same report as pmid:39265478 (preprint, repository or version copy); pmid:39265478 is the charted copy
doi:10.48550/arxiv.2406.17487	Smit et al. 2024. arXiv (Cornell University). doi:10.48550/arxiv.2406.17487	duplicate_preprint	EXCLUDE: same report as pmid:39186906 (preprint, repository or version copy); pmid:39186906 is the charted copy
doi:10.5137/1019-5149.jtn.49659-25.3	Bozyiğit et al. 2025. Turkish Neurosurgery. doi:10.5137/1019-5149.jtn.49659-25.3	duplicate_report	EXCLUDE: same report as pmid:41553224 (second identifier); pmid:41553224 is the charted copy

ID	Study	Code	Quote
doi:10.5204/thesis.eprints.251499	Herath et al. 2024. Queensland University of Technology. doi:10.5204/thesis.eprints.251499	source_type	EXCLUDE: This thesis developed a standardized workflow for designing patient-specific bone scaffold implants to treat large bone defects.
doi:10.5281/zenodo.18811309	Ismailov et al. 2026. Zenodo (CERN European Organization for Nuclear Research). doi:10.5281/zenodo.18811309	duplicate_report	EXCLUDE: same report as doi:10.5281/zenodo.18811308 (second identifier); doi:10.5281/zenodo.18811308 is the charted copy
doi:10.5281/zenodo.19017939	Apoorva Saini et al. 2026. Zenodo (CERN European Organization for Nuclear Research). doi:10.5281/zenodo.19017939	source_type	EXCLUDE: PATIENT-SPECIFIC IMPLANTS VERSUS CONVENTIONAL PLATING SYSTEMS IN GENIOPLASTY: A COMPREHENSIVE COMPARATIVE ANALYSIS
doi:10.5281/zenodo.19017940	Apoorva Saini et al. 2026. Zenodo (CERN European Organization for Nuclear Research). doi:10.5281/zenodo.19017940	duplicate_report	EXCLUDE: same report as doi:10.5281/zenodo.19017939 (second identifier); doi:10.5281/zenodo.19017939 is the charted copy
doi:10.5281/zenodo.19542555	Rossi et al. 2023. Zenodo (CERN European Organization for Nuclear Research). doi:10.5281/zenodo.19542555	duplicate_report	EXCLUDE: same report as doi:10.5281/zenodo.19542554 (second identifier); doi:10.5281/zenodo.19542554 is the charted copy
doi:10.5281/zenodo.19562531	Axrorov et al. 2026. Zenodo (CERN European Organization for Nuclear Research). doi:10.5281/zenodo.19562531	duplicate_report	EXCLUDE: same report as doi:10.5281/zenodo.19562530 (second identifier); doi:10.5281/zenodo.19562530 is the charted copy
doi:10.53555/b6xn7a40	Anwar et al. 2024. Journal of Population Therapeutics and Clinical Pharmacology. doi:10.53555/b6xn7a40	not_psi	EXCLUDE: The study’s main objective is to find the patient-specific factors and implant design considerations in PFR for non-oncologic hip salvage.
doi:10.5445/ir/1000190624	Salem et al. 2026. Repository KITopen (Karlsruhe Institute of Technology). doi:10.5445/ir/1000190624	duplicate_preprint	EXCLUDE: same report as doi:10.1109/access.2026.3663618 (preprint, repository or version copy); doi:10.1109/access.2026.3663618 is the charted copy
doi:10.55981/metalurgi.2023.738	Mayasari et al. 2023. Metalurgi. doi:10.55981/metalurgi.2023.738	not_psi	EXCLUDE: This study further analyzes material stress, particularly to compute the VMS (Von Misses stress) of the Ti6Al4V bone plate.
doi:10.5604/01.3001.0054.8694	Patrycja et al. 2024. Bio-Algorithms and Med-Systems. doi:10.5604/01.3001.0054.8694	case_report	EXCLUDE: A 32-year-old male patient, who sustained a mandible injury, served as a case study.
doi:10.58837/chula.the.2024.1000	Kamboonsri et al. 2024. . doi:10.58837/chula.the.2024.1000	source_type	EXCLUDE: TYPES: dissertation
doi:10.6084/m9.figshare.c.8613367	Riad et al. 2026. Figshare. doi:10.6084/m9.figshare.c.8613367	duplicate_preprint	EXCLUDE: same report as pmid:42482012 (preprint, repository or version copy); pmid:42482012 is the charted copy
doi:10.6084/m9.figshare.c.8613367.v1	Riad et al. 2026. Figshare. doi:10.6084/m9.figshare.c.8613367.v1	duplicate_preprint	EXCLUDE: same report as pmid:42482012 (preprint, repository or version copy); pmid:42482012 is the charted copy
doi:10.64951/jmdnt.2026.2.16	Yildirim et al. 2026. Journal of Medicine and Dentistry. doi:10.64951/jmdnt.2026.2.16	source_type	EXCLUDE: This narrative translational review proposes an end-to-end research framework for artificial intelligence-assisted and three-dimensional printing-supported maxillofacial trauma surgery.
doi:10.65204/djms-mfba-p	Rasheed et al. 2025. Dijlah Journal of Medical Sciences P-ISSN 3078-3178 E-ISSN 3078-8625. doi:10.65204/djms-mfba-p	duplicate_report	EXCLUDE: same report as doi:10.65204/djms-multi-factor-biomechanical (second identifier); doi:10.65204/djms-multi-factor-biomechanical is the charted copy
doi:10.7939/r36q1sn87	Trovato et al. 2016. University of Alberta Library. doi:10.7939/r36q1sn87	not_psi	EXCLUDE: This study explores the feasibility of developing a generic talus bone prosthetic for patients in need of talar replacements.
pmid:24909393	Thien et al. 2015. World neurosurgery. PMID	no_method	EXCLUDE: To characterize complication and

ID	Study	Code	Quote
	24909393		failure rates and outcomes of patients who underwent cranioplasty with polyetheretherketone (PEEK) and titanium implants and to compare complication and failure rates between the 2 implants.
pmid:25556524	Kantelhardt et al. 2015. International journal of computer assisted radiology and surgery. PMID 25556524	not_psi	EXCLUDE: The resulting implant positions were used as operative plan for image guidance and finally implemented in a physical spine model.
pmid:26363532	Bah et al. 2015. Medical engineering & physics. PMID 26363532	not_psi	EXCLUDE: The proposed methodology is a valuable tool for automatically generating a large specific population of femurs, targeting specific patients, supporting implant design and femoral reconstructive surgery.
pmid:26405891	Liu et al. 2015. Bio-medical materials and engineering. PMID 26405891	no_method	EXCLUDE: This paper presents a computer graphics method to automatically extract AFC from 3D hip bone models reconstructed from CT images.
pmid:26432286	Davis et al. 2015. Medical engineering & physics. PMID 26432286	not_psi	EXCLUDE: This method presents a possible solution for the characterization of characteristic thickness along the length of the bone and may also aid in the development of orthopedic implant design and subject specific finite element models.
pmid:26598243	Scolozzi et al. 2015. Revue de stomatologie, de chirurgie maxillo-faciale et de chirurgie orale. PMID 26598243	case_report	EXCLUDE: Four patients had surgical splints and cutting guides for correction of maxillomandibular asymmetries, three had surgical cutting guides and customized internal distractors for correction of severe maxillary deficiencies and three had custom-made implants for additional chin contouring and/or mandibular defects following bimaxillary osteotomies and sliding genioplasty.
pmid:26803563	Liu et al. 2016. Computer methods and programs in biomedicine. PMID 26803563	no_method	EXCLUDE: On this condition, we can reconstruct the personalized femoral head 3D models and design the personalized femoral head prosthesis.
pmid:27167515	Anchieta et al. 2016. International journal of computer assisted radiology and surgery. PMID 27167515	case_report	EXCLUDE: The surgery which illustrates the case was conducted in a university hospital of Medicine School of the Federal University of Sao Paulo-UNIFESP, which is public and free.
pmid:27431399	Serra et al. 2016. Biofabrication. PMID 27431399	not_psi	EXCLUDE: 3D-printing is a promising technique for medical applications and this study paves the way for future development of customised implants in spinal surgical applications.
pmid:27800592	Mathew et al. 2016. Progress in orthodontics. PMID 27800592	not_psi	EXCLUDE: Then, a custom-fabricated bone-borne palatal expander (BBPE) was constructed which consisted of a modified HYRAX expander (Leone, Italy) which enables microimplant placement and was laser scanned to create a 3D model.
pmid:27981068	Lim et al. 2016. Journal of medical imaging (Bellingham, Wash.). PMID 27981068	not_psi	EXCLUDE: Validation against expert segmentation was performed on 20 clinical knee MRI volumes and achieved a mean dice similarity coefficient of 0.949.
pmid:28199931	Ackland et al. 2017. Journal of the mechanical behavior of biomedical materials. PMID 28199931	case_report	EXCLUDE: The Melbourne prosthetic TMJ was developed for a 58-year-old female recipient with end-stage osteoarthritis of the TMJ.
pmid:28254310	Didier et al. 2017. Materials science & engineering. C, Materials for biological applications. PMID 28254310	not_psi	EXCLUDE: Indeed, the important feature is the curvature and the no-plate topography of the skull bone, so the bone is modeled by a semi-cylinder.

ID	Study	Code	Quote
pmid:28427318	Zhang et al. 2017. Proceedings of the Institution of Mechanical Engineers. Part H, Journal of engineering in medicine. PMID 28427318	no_method	EXCLUDE: In this study, we quantified the different effects of applying the medial and lateral loads separately versus the traditional total axial load application on contact mechanics and wear prediction of a patient-specific knee prosthesis.
pmid:28585457	Dharme et al. 2017. Journal of medical engineering & technology. PMID 28585457	not_psi	EXCLUDE: Based on L9 orthogonal array, nine hip implant shapes with three different geometric parameters were modelled.
pmid:28623521	Lim et al. 2017. Child's nervous system : ChNS : official journal of the International Society for Pediatric Neurosurgery. PMID 28623521	non_huma n	EXCLUDE: Each specimen was implanted in the femur of a rabbit and was evaluated using histological measurements and push-out test.
pmid:28747263	Diaconu et al. 2017. Journal of cranio-maxillo-facial surgery : official publication of the European Association for Cranio-Maxillo-Facial Surgery. PMID 28747263	no_method	EXCLUDE: The aim of this study is to analyze the validity and reliability of CT orbital volume measurements.
pmid:29408000	Park et al. 2018. Computers in biology and medicine. PMID 29408000	not_psi	EXCLUDE: Three different bone regions with well-known physiological loadings are selected to illustrate the method.
pmid:30286374	Entezari et al. 2019. Journal of the mechanical behavior of biomedical materials. PMID 30286374	not_psi	EXCLUDE: The proposed method is implicating to different clinical applications, such as the optimal selection of patient-specific fixation plates and screw system.
pmid:30337077	Volpe et al. 2018. Computer methods and programs in biomedicine. PMID 30337077	case_report	EXCLUDE: The methodology has been tested on a complex case study.
pmid:30366309	Minnema et al. 2018. Computers in biology and medicine. PMID 30366309	not_psi	EXCLUDE: The aim of the present study was to develop and train a convolutional neural network (CNN) for bone segmentation in computed tomography (CT) scans.
pmid:30520006	Chen et al. 2019. Medical & biological engineering & computing. PMID 30520006	not_psi	EXCLUDE: This fast and robust segmentation of trabecular and cortical bone boundary of the femur and tibia has the potential of providing a basis for surgical planning and more accurate finite element models of the knee joint.
pmid:30562250	Osterhoff et al. 2019. Journal of orthopaedic trauma. PMID 30562250	not_psi	EXCLUDE: In this cross-sectional study, an anatomically preshaped acetabular fracture implant was fitted on 3D surface models of 516 pelvises from a preexisting bone database using a software tool for automated implant fitting (SOMA, Stryker Orthopaedic Modeling and Analytics) of a CAD model of the implant.
pmid:30801978	Chatterjee et al. 2019. International journal for numerical methods in biomedical engineering. PMID 30801978	not_psi	EXCLUDE: The optimum implant geometry varied for different bone condition, and the findings act as guideline for designing patient-specific implant.
pmid:30881789	Han et al. 2018. Plastic and reconstructive surgery. Global open. PMID 30881789	case_report	EXCLUDE: Patient 3 was an 18-year-old man who had received right partial maxillectomy for chondrosarcoma 9 years before presenting to our department; the procedure had resulted in a medial maxilla defect on the right side of the face.
pmid:30928205	Gibbons et al. 2019. Journal of biomechanics. PMID 30928205	not_psi	EXCLUDE: The statistical shape-function model generated instantaneous joint mechanics predictions using a maximum of 130 training simulations, making it ideally suited for integration into a patient-specific design and alignment optimization pipeline.
pmid:31231678	Hollister et al. 2016. ACS biomaterials science & engineering. PMID 31231678	non_huma n	EXCLUDE: A total of 15 pigs were implanted with 3D printed splint, 5 pigs for each wall thickness design.
pmid:31367783	Saponaro et al. 2020. Child's nervous system : ChNS : official journal of the International	no_method	EXCLUDE: The aim of this study was to evaluate the overall volumetric results in

ID	Study	Code	Quote
	Society for Pediatric Neurosurgery. PMID 31367783		patients with facial imbalance after onlay custom implant positioning for mandible and fronto-orbital reconstructions.
pmid:31482715	Taghizadeh et al. 2019. Computer methods in biomechanics and biomedical engineering. PMID 31482715	no_method	EXCLUDE: Since anatomical correspondences are propagated during the segmentation process, this approach can directly yield anatomical measurements, provide design parameters for personalized surgical instruments, or determine the bone geometry to manufacture patient-specific implants.
pmid:31559166	Durastanti et al. 2019. Quantitative imaging in medicine and surgery. PMID 31559166	not_psi	EXCLUDE: The final scope is to characterise these diagnostic devices and the registrations procedures in the perspective of subject-specific modelling and custom-made prosthesis design.
pmid:31655451	Huang et al. 2019. Clinical biomechanics (Bristol, Avon). PMID 31655451	not_psi	EXCLUDE: Three types of reconstruction were explored, and corresponding finite element models were simulated: four-rod reconstruction, four-rod plus anterior column reconstruction, and 3D-printed total sacrum prosthesis reconstruction.
pmid:31741290	Epasto et al. 2019. Medical & biological engineering & computing. PMID 31741290	case_report	EXCLUDE: The cage, made of Ti6Al4V ELI alloy, was produced via electron beam melting (EBM) process and surgically implanted in a female subject, 50 years old.
pmid:31783282	Prasadh et al. 2020. Journal of the mechanical behavior of biomedical materials. PMID 31783282	not_psi	EXCLUDE: Finite element analysis was performed, and geometric data obtained from a human-sized mandible.
pmid:31843280	Abo Sharkh et al. 2020. Journal of oral and maxillofacial surgery : official journal of the American Association of Oral and Maxillofacial Surgeons. PMID 31843280	not_psi	EXCLUDE: A reconstructed jaw model was printed in-house using a commercially available fused deposition modeling-based desktop 3-dimensional (3D) printer (Qidi Technology, Zhejiang, China) and used to prebend the reconstruction plate.
pmid:32155859	Bolshakov et al. 2020. Materials (Basel, Switzerland). PMID 32155859	non_human	EXCLUDE: The femur bones of rabbits were used.
pmid:32242531	Campioni et al. 2020. Annali dell'Istituto superiore di sanita. PMID 32242531	not_psi	EXCLUDE: The objects were designed as prototypes for design validation and with a possible application in preoperative surgical planning on mandible models.
pmid:32383353	Zhang et al. 2020. Orthopaedic surgery. PMID 32383353	not_psi	EXCLUDE (A2): The prosthetic stems were sized from the medullary-cavity diameter, while the tooth-mechanism geometry was otherwise a common design.
pmid:32506737	Wang et al. 2020. The international journal of medical robotics + computer assisted surgery : MRCAS. PMID 32506737	no_evaluation	EXCLUDE: With EasyImplant, a complete porous implant model can be designed in a short time.
pmid:33072504	Vignesh et al. 2020. Journal of oral biology and craniofacial research. PMID 33072504	case_report	EXCLUDE: Here we present an interesting case report where FEA was done to biomechanically evaluate patient specific total joint replacement prosthesis for bilateral TMJ replacement.
pmid:33080431	Lewin et al. 2020. Journal of the mechanical behavior of biomedical materials. PMID 33080431	not_psi	EXCLUDE: The models were validated with previously obtained experimental data for two different CaP-Ti implant designs (D1 and D2), in which generically shaped implant specimens were loaded in compression at either quasi-static (1 mm/min) or impact (5 kg, 1.52 m/s) loading rates.
pmid:33122820	Bozkurt et al. 2020. Scientific reports. PMID 33122820	not_psi	EXCLUDE: Spring implantation was simulated using spring/dashpot link elements in MSC Marc, by specifying spring stiffness (1.2 mm wire diameter springs were used in Patient 1, and 1.4 mm wire diameter in Patient 2 and Patient 3) and initial force in a compressed

ID	Study	Code	Quote
			spring according to the characteristics reported in 39.
pmid:33153138	Ferràs-Tarragó et al. 2020. Journal of clinical medicine. PMID 33153138	not_psi	EXCLUDE: The objective of this study is to evaluate the symmetry of both femurs in adults to determine the error made when using the healthy side as a reference in the planning of derotational femoral osteotomies.
pmid:33187871	Koper et al. 2021. Journal of the mechanical behavior of biomedical materials. PMID 33187871	not_psi	EXCLUDE: A computer-aided design (CAD) model with a segmental defect was created based on the geometry of a polyurethane mandible model.
pmid:33452611	Li et al. 2021. Medical & biological engineering & computing. PMID 33452611	not_psi	EXCLUDE: These findings can be used in future studies to assess the feasibility of patient-specific implants using the mirrored contralateral hemipelvis as a template for unilateral pelvic fracture fixation.
pmid:33494021	Kang et al. 2021. Journal of the mechanical behavior of biomedical materials. PMID 33494021	case_report	EXCLUDE: Finally, a clinical case report proved the successful application of new functional repair method with good stability and post-operative performance.
pmid:33498498	Park et al. 2021. Micromachines. PMID 33498498	no_evaluation	EXCLUDE: Single material bone replacement shapes are provided with their corresponding multi-material topology optimization results for visual comparison.
pmid:33647729	Marcián et al. 2021. Journal of the mechanical behavior of biomedical materials. PMID 33647729	no_method	EXCLUDE: The effect of image resolution on mechanical simulations of bone-implant interaction has not been thoroughly studied yet.
pmid:33784838	Tümer et al. 2021. The Journal of hand surgery, European volume. PMID 33784838	not_psi	EXCLUDE: We studied the three-dimensional (3-D) shape variations and symmetry of the lunate to evaluate whether a contralateral shape-based approach to design patient-specific implants for treatment of Kienböck's disease is accurate.
pmid:33864971	Yao et al. 2021. Computers in biology and medicine. PMID 33864971	case_report	EXCLUDE: Then, the P-Plate was manufactured via electron beam melting and implanted into the foot of a patient.
pmid:33914952	Baines et al. 2022. Journal of orthopaedic research : official publication of the Orthopaedic Research Society. PMID 33914952	not_psi	EXCLUDE: Subject-specific computational models of the pelvic bones were constructed from the CT data.
pmid:34191377	Youssefian et al. 2022. Journal of orthopaedic research : official publication of the Orthopaedic Research Society. PMID 34191377	no_method	EXCLUDE: These results provide insight into the implementation of a robust platform for designing patient-specific implants capable of maintaining or modifying the stress in bones.
pmid:34715640	Wieja et al. 2022. Journal of the mechanical behavior of biomedical materials. PMID 34715640	no_method	EXCLUDE: Based on the results of the modelling, in a subsequent optimization process section forces at the interface between the bone tissue and jawbone implant can be determined and used to optimize the design of the jawbone implant.
pmid:34795980	Khomutinnikova et al. 2021. Sovremennye tekhnologii v meditsine. PMID 34795980	case_report	EXCLUDE: In practice, the technology has now been tested in 2 patients with severe post-traumatic deformity of ZOC due to displacement of fragments and presence of wide bone defects in the orbital floor.
pmid:34983845	Bastek et al. 2022. Proceedings of the National Academy of Sciences of the United States of America. PMID 34983845	not_psi	EXCLUDE: Second, we apply our design framework to synthetic bone.
pmid:35072825	Spalthoff et al. 2022. International journal of implant dentistry. PMID 35072825	not_skeletal	EXCLUDE: For this study, a simplified protocol was used, and only the digital datasets of the temporary prostheses were used for all analyses.
pmid:35245699	Izri et al. 2022. Computers in biology and	not_psi	EXCLUDE: A cubic structure with two rigid surfaces on the top and bottom is outlined to

ID	Study	Code	Quote
	medicine. PMID 35245699		separate the effect of the hip implant cross-sectional area variations.
pmid:35669917	Chen et al. 2022. Frontiers in medicine. PMID 35669917	not_psi	EXCLUDE: In this study, we aim to further study a three-dimensional (3D) CNN-based method to perform knee bone and cartilage segmentation.
pmid:35685746	Shilo et al. 2022. Plastic and reconstructive surgery. Global open. PMID 35685746	case_report	EXCLUDE: Two cases of Luhr III mandibles were treated using a novel crib-shaped PSI with simultaneous iliac-crest free bone graft.
pmid:35835610	Ebker et al. 2022. The British journal of oral & maxillofacial surgery. PMID 35835610	no_evaluation	EXCLUDE: These devices were successfully applied in all cases, and no major complications were experienced.
pmid:35872223	Preda et al. 2022. Journal of dentistry. PMID 35872223	no_method	EXCLUDE: Automated segmentation of the maxillofacial complex could act as an alternative to the conventional segmentation techniques for improving the efficiency of the digital workflows.
pmid:35880901	Choudhury et al. 2022. Proceedings of the Institution of Mechanical Engineers. Part H, Journal of engineering in medicine. PMID 35880901	not_psi	EXCLUDE (A2): Implants varied only in thread length, diameter, and pitch across strong, weak, and normal basal-bone conditions
pmid:35893304	Dinu et al. 2022. Journal of personalized medicine. PMID 35893304	no_evaluation	EXCLUDE: The aim of this paper was to evaluate the orbital soft tissue after posttraumatic reconstruction of the orbital walls' fractures.
pmid:35950785	Li et al. 2023. Journal of prosthodontics : official journal of the American College of Prosthodontists. PMID 35950785	non_human	EXCLUDE: In addition, animal experiments were performed to verify the feasibility of the implant design.
pmid:35995899	Yun et al. 2023. Journal of digital imaging. PMID 35995899	not_psi	EXCLUDE: In this paper, we propose a method for improving the inter-slice resolution to reduce the aliasing effect of the 3D-reconstructed orbital bone.
pmid:36013222	Merema et al. 2022. Journal of personalized medicine. PMID 36013222	no_method	EXCLUDE: With this study, we aimed to propose a contemporary method to determine the patient-specific intrinsic strength value of the elevator muscles.
pmid:36114907	Nogueira-Reis et al. 2023. Clinical oral investigations. PMID 36114907	not_psi	EXCLUDE: The automated segmentation of these structures simultaneously could act as a valuable tool in clinical orthodontics, implant rehabilitation, and any oral or maxillofacial surgical procedures, where visualization of MVP and its relationship with surrounding structures is a necessity for reaching an accurate diagnosis and patient-specific treatment planning.
pmid:36185133	Moncayo-Matute et al. 2022. Heliyon. PMID 36185133	case_report	EXCLUDE: A 19-year-old patient presents with a gunshot wound to the left fronto-orbital region.
pmid:36207344	Glatzeder et al. 2022. Scientific reports. PMID 36207344	not_psi	EXCLUDE: Thus, it can be concluded that prefabricated spacers enable the surgeon to find the right selection for each patient without the need for expensive custom-made spacer implants.
pmid:36246391	Vega et al. 2022. Frontiers in bioengineering and biotechnology. PMID 36246391	no_method	EXCLUDE: Once validated, this computational approach could ultimately influence surgical, rehabilitation, and implant design decisions to meet the unique clinical needs of individual pelvic sarcoma patients.
pmid:36310474	Zhang et al. 2022. Sheng wu yi xue gong cheng xue za zhi = Journal of biomedical engineering = Shengwu yixue gongchengxue zazhi. PMID 36310474	not_psi	EXCLUDE: This paper provides a new technology for rapid 3D modeling of bone geometry, which is helpful to promote rapid biomechanical analysis for patients, and provides a new idea for the selection of

ID	Study	Code	Quote
			orthopedic implants and the rapid research and development of customized implants.
pmid:36442275	Miljanovic et al. 2022. Computers in biology and medicine. PMID 36442275	no_evaluation	EXCLUDE: Finite Element Analysis (FEA) was used to evaluate the mechanical behaviour of the 3D-printed surgical guide during dental implant placement.
pmid:36662087	Gregory et al. 2023. Journal of functional biomaterials. PMID 36662087	no_evaluation	EXCLUDE: This proof-of-concept work aims to illustrate the accurate 3D printing of the bone scaffold constructs which in future can be seeded with stem cells such as bone marrow mesenchymal stem cells [50 , 51 , 52] for bone regeneration and eventual total replacement of the implant.
pmid:36698627	Guo et al. 2022. Frontiers in bioengineering and biotechnology. PMID 36698627	case_report	EXCLUDE: In the retrospective clinical study, four patients who had undergone prosthetic reconstruction were included.
pmid:36777247	Naghavi et al. 2023. Frontiers in bioengineering and biotechnology. PMID 36777247	non_human	EXCLUDE: The implant was designed for a large, left, fourth-generation artificial composite femoral bone (Model 3406, Sawbones, Pacific Research Laboratories Inc.™, Vashon, United States), and developed to have as perfectly fit as possible within the adjacent cortical bone tissue.
pmid:36816944	Tel et al. 2023. Frontiers in oncology. PMID 36816944	case_report	EXCLUDE: 3 patients were enrolled in this series between January 2021 and June 2022 and fulfilled the following requirements: surgically determined broad skeletal defect with simultaneous involvement of the midface and the mandible; simultaneous use of two osseous flaps for reconstruction; entirely digital workflow.
pmid:36829617	Wysocki et al. 2023. Bioengineering (Basel, Switzerland). PMID 36829617	not_psi	EXCLUDE: The incorporation of patient-specific statistical reconstructions of healthy anatomical structures into hip prosthesis development and surgical planning could help to ensure that patients have positive outcomes following hip arthroplasty.
pmid:36898626	Wang et al. 2023. World neurosurgery. PMID 36898626	not_psi	EXCLUDE: This study proposed a new innovative design method for interbody fusion cages, which not only provides new insights into the innovative design of interbody fusion cages but may also guide the customized design of interbody fusion cages in different pathological environments.
pmid:37034247	Nakhaei et al. 2023. Frontiers in bioengineering and biotechnology. PMID 37034247	not_psi	EXCLUDE: The generic osteosynthesis plate was fabricated by Selective Laser Melting (SLM) from a titanium alloy (Ti6Al4V ELI) using an SLM® 125 HL - SLM Solutions Machine.
pmid:37266733	Dóczi et al. 2023. Biomechanics and modeling in mechanobiology. PMID 37266733	no_method	EXCLUDE: The patient’s postoperative and control CT scans were utilized for the qualitative verification of the results.
pmid:37323484	Park et al. 2023. International journal of bioprinting. PMID 37323484	not_psi	EXCLUDE: The present study entailed mechanical and microstructural analyses for Ti-6Al-4V solid-mesh hybrid structures produced by electron beam melting (EBM).
pmid:37323501	Mao et al. 2023. International journal of bioprinting. PMID 37323501	case_report	EXCLUDE: This paper reports a clinical case on the application of FEA in designing patient-specific tantalum prostheses with an appropriate pore size and wire diameter for knee joint revision.
pmid:37347629	Hohlmann et al. 2023. IEEE transactions on ultrasonics, ferroelectrics, and frequency control. PMID 37347629	not_psi	EXCLUDE: A fully-automatic and robust processing pipeline is proposed, generating full bone models from 3D freehand ultrasound scanning.

ID	Study	Code	Quote
pmid:37688247	Moncayo-Matute et al. 2023. Polymers. PMID 37688247	case_report	EXCLUDE: This article reports on a patient who required a cranial protection system.
pmid:37712186	Wang et al. 2023. Orthopaedic surgery. PMID 37712186	not_psi	EXCLUDE: A 3D computer-aided design (CAD) model of the left tibia was obtained based on a composite tibia model (#3402; Sawbones, Pacific Research Labs, Vashon, WA, USA).
pmid:37928878	Ayhan et al. 2023. International journal of medical sciences. PMID 37928878	not_psi	EXCLUDE: This re-generated 3D model is based on all the mean values of the 11 patients' data and contains all anatomically relevant points.
pmid:38116196	Zhu et al. 2023. Frontiers in bioengineering and biotechnology. PMID 38116196	case_report	EXCLUDE: The surgical procedures were performed by the same surgeon (CL Zhang), and these procedures include a single case of Type I and a single case of Type II.
pmid:38143713	Yu et al. 2023. 3D printing and additive manufacturing. PMID 38143713	not_psi	EXCLUDE: Results show that the additively manufactured chain mail sheet is not only able to exhibit a natural strain-stiffening rotational response but also is able to reproduce natural anisotropy of three natural disc specimens in the six most common rotational scenarios in daily life.
pmid:38151940	Zhang et al. 2023. Sheng wu yi xue gong cheng xue za zhi = Journal of biomedical engineering = Shengwu yixue gongchengxue zazhi. PMID 38151940	not_psi	EXCLUDE: Therefore, the method we proposed allows a more accurate 3D bone reconstruction, which facilitates the development of personalized prosthesis design, precise positioning, and quick biomechanical analysis.
pmid:38274145	Taniguchi et al. 2023. JBJS essential surgical techniques. PMID 38274145	source_type	EXCLUDE: Total talar replacement is performed with the patient in a supine position.
pmid:38361509	Akhtar et al. 2024. Journal of clinical orthopaedics and trauma. PMID 38361509	no_evaluation	EXCLUDE: The algorithm can infer implant specifications that align with patient-specific bone characteristics by training the AI model on a diverse dataset.
pmid:38394650	Decker et al. 2024. Journal of neurosurgery. Spine. PMID 38394650	not_psi	EXCLUDE: A tether pedicle screw (TPS) enables individual stepless pretensioning and is placed at one or two levels above the upper instrumented vertebra (UIV+1 and UIV+2, respectively).
pmid:38398354	Sigron et al. 2024. Journal of clinical medicine. PMID 38398354	no_method	EXCLUDE: In this study, the software was used to measure and compare healthy orbits.
pmid:38408764	De Angelis et al. 2024. Journal of orthopaedic research : official publication of the Orthopaedic Research Society. PMID 38408764	no_method	EXCLUDE: Postop computed tomography scans taken within 1 week of surgery and at 1-year postsurgery were co-registered and analyzed.
pmid:38430381	Fishman et al. 2024. International journal of computer assisted radiology and surgery. PMID 38430381	no_method	EXCLUDE: For both implant and skull shapes, the inner and outer cortical surfaces were segmented, and the thickness between them was measured.
pmid:38486844	Mccullough et al. 2024. IEEE journal of translational engineering in health and medicine. PMID 38486844	not_psi	EXCLUDE: These numbers are not finalized design requirements, as bone size will vary across patients; instead they represent target values when identifying the design space of suitable caged hinge designs.
pmid:38659643	Betti et al. 2024. Frontiers in bioengineering and biotechnology. PMID 38659643	not_psi	EXCLUDE: The information obtained could be adopted to support the design of novel prosthetic devices able to more adequately match the anatomy of the canal with respect to the current commercial solutions.
pmid:38762148	Fleet et al. 2024. Journal of shoulder and elbow surgery. PMID 38762148	not_psi	EXCLUDE: This technology can serve as a useful template to visually represent the premorbid healthy glenoid in patients with severe glenoid bony erosions.

ID	Study	Code	Quote
pmid:38784769	Namvar et al. 2024. Frontiers in bioengineering and biotechnology. PMID 38784769	non_human	EXCLUDE: Here, we report the results of our in silico biomechanical testing completed for 9 sheep using preoperative CT data.
pmid:38896405	Vanslambrouck et al. 2024. International journal of computer assisted radiology and surgery. PMID 38896405	not_psi	EXCLUDE: Nevertheless, the results underscore the potential of GPMM-based reconstruction as a promising alternative for designing patient-specific implants.
pmid:38992884	Ramesh et al. 2024. Journal of orthopaedic research : official publication of the Orthopaedic Research Society. PMID 38992884	no_method	EXCLUDE: We aimed to better understand the bone remodeling that occurs following acetabular reconstruction.
pmid:39040498	Lin et al. 2024. Frontiers in bioengineering and biotechnology. PMID 39040498	not_psi	EXCLUDE: We designed a novel 3D-printed glenohumeral fusion prosthesis, verified its feasibility and safety, and explored the potential improvement in biomechanical stability through a lateral plate fixation with it by finite element analysis and biomechanical experiments, providing mechanical evidence for its clinical application.
pmid:39086493	Van der Wel et al. 2024. Plastic and reconstructive surgery. Global open. PMID 39086493	case_report	EXCLUDE: The result at the follow-up consultation 4 months after surgery was satisfactory for the patient.
pmid:39101231	Xu et al. 2024. Orthopaedic surgery. PMID 39101231	not_psi	EXCLUDE: The screw-rod system, 3D-printed prosthesis, titanium mesh cage, and bone graft were sketched in Abaqus.
pmid:39175032	Deng et al. 2024. Journal of orthopaedic surgery and research. PMID 39175032	not_psi	EXCLUDE: The porous UKA knee models were generated based on fixed-bearing UKA (Zimmer Inc., Warsaw, Indiana, USA), and native knee models.
pmid:39244030	He et al. 2025. Journal of stomatology, oral and maxillofacial surgery. PMID 39244030	no_method	EXCLUDE: Twelve genioplasty procedures were designed and implemented on 3D-printed resin mandibular models by the same surgeon using three different approaches: AR+3DT group (AR+PSI), 3DT group (patient-specific titanium plate), and a traditional free-hand group(FH group).
pmid:39429595	Humayun et al. 2024. Quantitative imaging in medicine and surgery. PMID 39429595	no_method	EXCLUDE: While this study has focused on knee bone segmentation, future research could explore the segmentation and geometrical modeling of other anatomical structures within the knee joint, such as ligaments and cartilage.
pmid:39487496	Acar et al. 2024. BMC oral health. PMID 39487496	not_psi	EXCLUDE (A2): Manufacturer CAD implant models with fixed dimensions were placed into a patient-derived maxillary model rather than anatomy-derived implant geometry
pmid:39489075	Eghan-Acquah et al. 2024. Computer methods and programs in biomedicine. PMID 39489075	not_psi	EXCLUDE: This study introduces a novel approach to refine implant selection by integrating personalized neuromusculoskeletal modelling with finite element analysis.
pmid:39593734	Kim et al. 2024. Bioengineering (Basel, Switzerland). PMID 39593734	not_psi	EXCLUDE: The proposed method was validated using a 50 μm high-resolution image of an artificial proximal femur obtained from previous research.
pmid:39603155	Alomar et al. 2025. Journal of the mechanical behavior of biomedical materials. PMID 39603155	not_psi	EXCLUDE: In this work, a new design of a mandibular reconstruction plate is proposed to promote osseointegration, while providing the necessary mechanical support during healing.
pmid:39628649	Herath et al. 2024. Frontiers in bioengineering and biotechnology. PMID 39628649	case_report	EXCLUDE: The first two clinical cases took place during the development of the workflow and hence, scaffolds were designed appropriately using the workflow and were subsequently manufactured and surgically implanted successfully.
pmid:39746033	Jiao et al. 2025. ACS applied materials &	not_psi	EXCLUDE: A synthetic biomimetic lumbar

ID	Study	Code	Quote
	interfaces. PMID 39746033		spine model (Sawbones, WA, USA) was used to test the developed deployable cage.
pmid:39757350	Lee et al. 2025. Journal of orthopaedic research : official publication of the Orthopaedic Research Society. PMID 39757350	not_psi	EXCLUDE: This model consisted of a patient-specific implant designed to address a superior-medial defect created in a standard pelvic geometry.
pmid:39892636	Xu et al. 2025. Bone. PMID 39892636	not_psi	EXCLUDE: Therefore, this study aimed to create a biomechanical research tool to predict the personalized shape and density of the paediatric femur using a statistical shape and density model for a population of children aged from 4 to 18 years old.
pmid:39902431	Kudva et al. 2025. Journal of maxillofacial and oral surgery. PMID 39902431	no_evaluat ion	EXCLUDE: The surgical results achieved were satisfactory in terms of both form and function.
pmid:40024182	Wan et al. 2025. Computers in biology and medicine. PMID 40024182	not_psi	EXCLUDE: Patient-specific finite element (FE) models were developed using CT data from three representative cases - two with Brown Class II defects and one with a Class III defect - reconstructed with single, double, and triple fibula segments.
pmid:40102993	Wu et al. 2025. Journal of orthopaedic surgery and research. PMID 40102993	non_huma n	EXCLUDE: When the “Fill” command from the “Tools” menu is executed, a 3D solid model of the pig femur is obtained.
pmid:40137323	Remus et al. 2025. Journal of functional morphology and kinesiology. PMID 40137323	no_method	EXCLUDE: A prosthesis of the distal surface could then be designed using a CT scan of the patient’s unaffected wrist and produced by 3D printing technology.
pmid:40180533	Dunbar et al. 2025. Medical engineering & physics. PMID 40180533	no_method	EXCLUDE: In a collaborative effort between a clinical and an engineering research team, this study evaluated whether patient-specific biomechanical modeling could predict, in a blinded fashion, the mode and location of a clinically-observed mechanical failure.
pmid:40481259	Andres et al. 2025. Scientific reports. PMID 40481259	not_psi	EXCLUDE: For example, the model can be adjusted to change the thickness of an intramedullary nail, place or remove screws, add or remove plates, or predict wrist motion outcomes before and after a planned reconstruction surgery.
pmid:40483235	Ten Brink et al. 2025. International journal of oral and maxillofacial surgery. PMID 40483235	no_method	EXCLUDE: By integrating these parameters into implant design and surgical planning, this method offers a step forward in developing personalized surgical strategies that could improve postoperative outcomes in mandibular reconstruction.
pmid:40503891	Goker et al. 2025. Journal of surgical oncology. PMID 40503891	not_psi	EXCLUDE: This study aims to test and compare machine-learning models for the prediction of early tumor endoprosthetic implant survival.
pmid:40512299	Choi et al. 2025. Tissue engineering and regenerative medicine. PMID 40512299	not_psi	EXCLUDE: This study proposed a novel method for optimizing the implantation angle of bone scaffolds, thereby enhancing their mechanical performance and integration with the surrounding bone tissue.
pmid:40529899	Zhang et al. 2025. Journal of orthopaedic translation. PMID 40529899	not_psi	EXCLUDE: For the convenience of choosing the most suitable 3D-SAVB for patient, a series of lordosis angle (0°–8°) and gradient height (20 mm–50 mm) were set.
pmid:40567253	Liang et al. 2025. Frontiers in bioengineering and biotechnology. PMID 40567253	case_report	EXCLUDE: Finally, we summarized the preliminary outcomes of three pediatric cases using the GFRS endoprostheses for reconstruction.
pmid:40578009	Khanna et al. 2025. Journal of clinical	not_psi	EXCLUDE: Advanced models can be applied

ID	Study	Code	Quote
	neuroscience : official journal of the Neurosurgical Society of Australasia. PMID 40578009		to customized, patient-oriented design, micro-structure mimicking manufacturing, pre-surgery planning, clinical follow-up, testing, and overall implant success.
pmid:40600341	Mohseni-Dargah et al. 2025. Head & neck. PMID 40600341	no_method	EXCLUDE: This study estimates the patient-specific loading limits in compromised mandibular reconstruction using finite element analysis to guide safe trismus therapy.
pmid:40681392	Coyle et al. 2025. Journal of cranio-maxillo-facial surgery : official publication of the European Association for Cranio-Maxillo-Facial Surgery. PMID 40681392	not_psi	EXCLUDE: This study introduces Amanita, a pioneering bionic design for a fully 3D-printed cranial implant made of polyether-ether-ketone (PEEK) functionalized with bioactive glass granules.
pmid:40694406	Jana et al. 2025. Journal of biomechanical engineering. PMID 40694406	not_psi	EXCLUDE: A large numerical campaign has been conducted on 18 geometries.
pmid:40715922	Shi et al. 2025. 3D printing in medicine. PMID 40715922	case_report	EXCLUDE: We reviewed three cases of Paprosky type III acetabular defects reconstructed using personalized 3D-printed porous titanium alloy augments.
pmid:40852617	S L et al. 2025. Advances in orthopedics. PMID 40852617	not_psi	EXCLUDE: This study proposes a novel framework that integrates CT-based imaging data with machine learning algorithms to predict patient-specific Young's modulus values.
pmid:40863475	Luo et al. 2025. Journal of imaging. PMID 40863475	not_skeletal	EXCLUDE: These implants provide mechanical support to prevent soft tissue overgrowth-related restenosis and, when loaded with therapeutic agents, can deliver antimicrobial drugs locally to reduce infection risk.
pmid:41040412	Li et al. 2025. Frontiers in bioengineering and biotechnology. PMID 41040412	non_human	EXCLUDE: Using fourth-generation composite femurs simulating 10 cm defects (n = 16), we compared axial compression, torsion, four-point bending stiffness, and cyclic fatigue performance against intact bones (Group D) and diaphyseal fractures without defects (Group C).
pmid:41074944	Raith et al. 2025. Clinical oral investigations. PMID 41074944	not_psi	EXCLUDE: This study demonstrates that AI-driven segmentation can generate anatomically valid edentulous mandibles from dentate CT scans with high accuracy.
pmid:41145678	Heins et al. 2025. Clinical oral investigations. PMID 41145678	no_method	EXCLUDE: Future research should focus on external validation of the current model.
pmid:41274196	Mallinos et al. 2026. Clinical biomechanics (Bristol, Avon). PMID 41274196	not_psi	EXCLUDE: Patient-specific, deterministic computational models were developed based on ten Lenke 1 AIS surgical cases.
pmid:41336765	Blomenkamp et al. 2025. Annual International Conference of the IEEE Engineering in Medicine and Biology Society. IEEE Engineering in Medicine and Biology Society. Annual International Conference. PMID 41336765	not_psi	EXCLUDE: The segmentation of anatomical regions provides a basis for patient-specific analysis and biomechanical modelling of the spine as well as developing of patient-specific implants.
pmid:41344151	Yoon et al. 2026. Hearing research. PMID 41344151	not_psi	EXCLUDE: This technology is expected to enhance the accuracy of biomechanical finite element simulations and serves as a foundational step toward advancing precision medicine in otologic surgery and custom prosthesis design.
pmid:41384766	Das et al. 2026. Biomaterials science. PMID 41384766	non_human	EXCLUDE: Physicochemical characterization, in vitro and in vivo biological assessment, especially site-specific implantation in a rabbit model, revealed the ability of the scaffold to accelerate bone remodelling.
pmid:41462789	Gomez Garcia et al. 2025. Children (Basel,	case_report	EXCLUDE: Virtual surgery predominated in

ID	Study	Code	Quote
	Switzerland). PMID 41462789		Group 2 (4 patients) and one in Group 1, combined with cutting guides for margins and Individual Prosthetic Solutions (IPS) prosthesis fitting (two patients: titanium and PEEK).
pmid:41498823	Lee et al. 2026. Biomedical microdevices. PMID 41498823	not_psi	EXCLUDE: Two types of test specimens were prepared based on the evaluation criteria: Type I (10 × 10 × 0.8 mm) for tests such as appearance, dimensions, porosity, mass, PCL content, and molecular weight, and Type II (10 × 30 × 0.8 mm) for tensile load testing.
pmid:41506030	Huppe et al. 2026. Computers in biology and medicine. PMID 41506030	not_psi	EXCLUDE: The method has the potential to achieve substantial radiation dose reduction compared to conventional CT while preserving sufficient anatomical detail for potential clinical tasks such as patient-specific implant planning and bone quality triage.
pmid:41553224	Bozyigit et al. 2026. Turkish neurosurgery. PMID 41553224	not_psi	EXCLUDE: A synthetic database of spinal bone Young modulus values was used, incorporating anatomical regions (cervical, thoracic, lumbar) and patient-specific factors (age, bone density, health status).
pmid:41597520	Tousidonis et al. 2026. Medicina (Kaunas, Lithuania). PMID 41597520	no_evaluation	EXCLUDE: Finally, the outcomes reported are predominantly process and utilization metrics; comparative clinical endpoints and validated patient-reported outcome measures (PROMs) were not prospectively collected.
pmid:41682908	Youssef et al. 2026. Journal of clinical medicine. PMID 41682908	case_report	EXCLUDE: Three clinical cases were analyzed using MAGIC-SSM-based reference alignment, with distance mapping applied when indicated.
pmid:41784751	Zhang et al. 2026. Medical & biological engineering & computing. PMID 41784751	not_psi	EXCLUDE: It provides critical osteotomy positioning parameters and design references for patient-specific implants, and enhances the accuracy and efficiency of joint replacement surgeries.
pmid:41800358	Ko et al. 2026. Frontiers in oral health. PMID 41800358	not_psi	EXCLUDE: After that, conventional plates were used for fixation, followed by the removal of the TPSP (Figure 1M).
pmid:41848582	Zhang et al. 2026. Orthopaedic surgery. PMID 41848582	not_psi	EXCLUDE: This work provides robust morphometric evidence to enhance understanding of clavicular variability and may inform both clinical decision-making and the development of anatomically congruent implants.
pmid:41861487	Pendzik et al. 2026. Biofabrication. PMID 41861487	no_evaluation	EXCLUDE: No biomechanical simulations or biological evaluations were performed at this stage, as the focus was deliberately placed on validating the functional design and manufacturing process.
pmid:41886180	Guven et al. 2026. Medical & biological engineering & computing. PMID 41886180	not_psi	EXCLUDE: This study introduces X2B and X2BR, two complementary neural implicit frameworks for reconstructing 3D skeletal anatomy from a single planar X-ray.
pmid:41938871	Lee et al. 2026. Clinics in orthopedic surgery. PMID 41938871	case_report	EXCLUDE: We selected 2 patients using the criteria mentioned above.
pmid:41976849	Cebrián-Carretero et al. 2026. Journal of clinical medicine. PMID 41976849	no_evaluation	EXCLUDE: Patient-specific implants were used in a limited subset of cases where reconstruction could not be adequately achieved with standard implants.
pmid:42020225	Kohansal et al. 2026. The Journal of prosthetic dentistry. PMID 42020225	case_report	EXCLUDE: A partial-mandible PSI with an extended fixation wing was designed, fabricated, and implanted in a 19-year-old patient after hemimandibulectomy.
pmid:42144585	Elayah et al. 2026. BMC surgery. PMID	not_psi	EXCLUDE: This retrospective study was

ID	Study	Code	Quote
	42144585		designed to validate a virtual reduction workflow.
pmid:42169041	Dere et al. 2026. BMC oral health. PMID 42169041	not_psi	EXCLUDE (A2): The six alternative implant configurations were modeled on Visible Human Project tomography rather than an individual patient's anatomy
pmid:42179769	Li et al. 2026. Biomaterials translational. PMID 42179769	case_report	EXCLUDE: First, the results presented are based on a single clinical case, which limits the ability to draw broader conclusions at this stage.
pmid:42210774	Prados-Privado et al. 2026. International journal for numerical methods in biomedical engineering. PMID 42210774	not_psi	EXCLUDE (A2): The synthetic cohort varied bone quality and loading, with optimisation prescribing parameters such as wider diameters for low-density bone
pmid:42233609	Gironi et al. 2026. Computer methods in biomechanics and biomedical engineering. PMID 42233609	not_psi	EXCLUDE: This study presents a patient-specific finite element and mechanobiological framework to evaluate early bone healing with reconstruction plates and miniplates.
pmid:42316149	Zhang et al. 2026. BMC biotechnology. PMID 42316149	not_psi	EXCLUDE: The femur model utilized in this study was the built-in standard femur template provided in Materialise Mimics 2020 software, which is a generic anatomical demonstration model for research and simulation purposes.
pmid:42317473	Iio et al. 2026. JSES international. PMID 42317473	not_psi	EXCLUDE: We aimed to develop a combined statistical shape model (SSM) of the scapula and humerus reflecting covariation, and to quantify subgroup differences by sex and stature with corresponding point clouds.
pmid:42345722	Türk et al. 2026. Biomimetics (Basel, Switzerland). PMID 42345722	not_psi	EXCLUDE: The three-dimensional (3D) maxillary geometry was reconstructed using orthographic and cross-sectional images obtained from open-access digital anatomical sources [16 , 17 , 18].
pmid:42407172	Gironi et al. 2026. Journal of the mechanical behavior of biomedical materials. PMID 42407172	no_method	EXCLUDE: A clinical reference finite element model (M-COU) representing the reconstructed mandible under physiological clenching conditions was used as an active design tool to derive the validation setup.
pmid:42539780	Hussein et al. 2026. Frontiers in surgery. PMID 42539780	case_report	EXCLUDE: Secondary reconstruction with PSIs was performed in three patients (42.9%).
pmid:42570496	Wan et al. 2026. Oral oncology. PMID 42570496	not_psi	EXCLUDE: Five mandibular reconstruction cases using vascularised osseous free flaps were analysed.
title:1ae5734e965933fa	Moncayo-Matute et al. 2022. . Surgical planning and finite element analysis for the neurocraneal protection in	duplicate_report	EXCLUDE: same report as pmid:36185133 (second identifier); pmid:36185133 is the charted copy
title:42023028a9de6872	S. et al. 2025. Dialnet (Universidad de la Rioja). Development of a Patient-Specific Plate for Hoffa Fracture:: CT-Based Segmentati	no_evaluation	EXCLUDE: The 3D-printed implant demonstrated accurate anatomical fit and alignment, confirmed through visual inspection and feedback from an orthopedic surgeon.
title:62dd0a1a93fe84af	Joyce et al. 2018. Orthopaedic Proceedings. Segmentation Accuracy of the Knee Joint Using MRI Technology	not_psi	EXCLUDE: This study was conducted to quantify the accuracy of MRI-based segmentation of the knee joint.
title:7c999d4b2b066c35	Ferguson et al. 2023. The Sydney eScholarship Repository (The University of Sydney). Next-Generation Scaffold-Based Implants for Mandibular Reconstructions: Patient-	non_human	EXCLUDE: Animal models (in silico, in vitro, and in vivo) are used for the research, development, test, and evaluation of bone implant prototypes.
title:837ea219c36fe570	Berghe et al. 2018. Orthopaedic Proceedings. AUTOMATIC RECONSTRUCTION OF LARGE ACETABULAR BONE DEFECTS USING STATISTICAL SHAP	not_psi	EXCLUDE: To illustrate the clinical use of the reconstruction method, one hemi-pelvis with a substantial defect was reconstructed.
title:973472df08a69edc	Kotaniemi et al. 2024. Työväentutkimus Vuosikirja. Complications, associated risk	source_type	EXCLUDE: This thesis consists of a series of retrospective studies comprising altogether 230

ID	Study	Code	Quote
	factors, and stability of the outcome of orthogna		patients operated on with either Le Fort I osteotomy or bimaxillary osteotomy in the Department of Oral and Maxillofacial Diseases, Head and Neck Centre, or in the Department of Plastic Surgery, Cleft Palate and Craniofacial Centre, Helsinki University Hospital (HUH), Helsinki, Finland, during 2006–2020.
title:bef7a4d6ad704fa2	Prasadh et al. 2021. . Biomechanics of alloplastic mandible reconstruction using biomaterials: The effe	duplicate_report	EXCLUDE: same report as pmid:31783282 (second identifier); pmid:31783282 is the charted copy
title:d5a82fbc298ba79c	Altamimi et al. 2019. . Novel bone fixation implants minimising stress shielding	source_type	EXCLUDE: TYPES: Dissertation
title:f9899d4ceab458c1	Kormi et al. 2026. Työväentutkimus Vuosikirja. Supporting Technologies in the Assessment and Reconstruction of Bony Orbit	source_type	EXCLUDE: This thesis assesses novel technologies of personalized medicine in orbital trauma and cleft patients.

Table S3. Technical credibility items

Study	Tier	Independent	Ground truth	Uncertainty	Reproducible inputs	Files available	Multiple anatomies
Kang et al. 2018. Biomechanics and modeling in mechanobiology. PMID 29730771	bench	y	y	y	y	n	n
Pontiki et al. 2022. Annual International Conference of the IEEE Engineering in Medicine and Biology Society. IEEE Engineering in Medicine and Biology Society. Annual International Conference. PMID 36085707	cadaver_phantom	y	y	y	y	y	y
Chaubey et al. 2025. . doi:10.1145/3774521.3774531	imaging_dataset	y	y	n	y	y	y
Shash et al. 2025. Scientific reports. PMID 39788988	in_silico	y	n	y	y	u	n
Shash et al. 2025. Journal of materials science. Materials in medicine. PMID 41436650	in_silico	y	y	y	y	u	n
Abel et al. 2015. The British journal of oral & maxillofacial surgery. PMID 25703687	in_silico	n	n	u	u	u	n
Aquilina et al. 2015. Craniomaxillofacial trauma & reconstruction. PMID 26000081	in_silico	n	n	u	u	u	n
Qin et al. 2015. Journal of cranio-maxillo-facial surgery : official publication of the European Association for Cranio-Maxillo-Facial Surgery. PMID 26174791	in_silico	n	n	n	u	u	y
Pinheiro et al. 2015. Journal of cranio-maxillo-facial surgery : official publication of the European Association for Cranio-Maxillo-Facial Surgery. PMID 26596361	in_silico	n	n	u	u	u	n
Gattinger et al. 2016. Current Directions in Biomedical Engineering. doi:10.1515/cdbme-2016-0025	bench	u	y	n	u	u	y
Sutradhar et al. 2016. Medical & biological engineering & computing. PMID 26660897	bench	y	y	u	u	u	y
Moiduddin et al. 2016. Technology and health care : official journal of the European Society for Engineering and Medicine. PMID 26835729	bench	y	y	u	u	u	y
Anssari Moin et al. 2016. International journal of dentistry. PMID 27274727	in_silico	n	n	y	y	n	n
Jahadakbar et al. 2016. Bioengineering (Basel, Switzerland). PMID 28952598	cadaver_phantom	y	y	y	y	n	y
Gutwald et al. 2017. Computer methods in biomechanics and biomedical engineering. PMID 27887036	bench	y	y	y	y	n	y
Xu et al. 2017. Medical engineering & physics. PMID 28629601	in_silico	n	n	u	u	u	n
Dai et al. 2018. Journal of Mechanics in Medicine and Biology. doi:10.1142/s0219519418500240	bench	u	u	n	n	u	u
Dahake et al. 2018. The international journal of medical robotics + computer assisted surgery : MRCAS. PMID 28868631	in_silico	u	n	y	u	u	y
Hingsammer et al. 2018. Journal of the mechanical behavior of biomedical materials. PMID 28888931	in_silico	n	n	n	u	u	n

Study	Tier	Independent	Ground truth	Uncertainty	Reproducible inputs	Files available	Multiple anatomies
Chen et al. 2018. Journal of cranio-maxillo-facial surgery : official publication of the European Association for Cranio-Maxillo-Facial Surgery. PMID 30025603	in_silico	n	n	n	u	u	n
Omidi et al. 2019. Engineering Solid Mechanics. doi:10.5267/j.esm.2018.10.001	in_silico	n	n	n	y	n	n
Gillingham et al. 2019. SUNScholar (Stellenbosch University). Retrospective study on mandible morphology towards improving implant design	imaging_dataset	y	y	n	u	u	y
Oldhoff et al. 2019. Research Repository (Delft University of Technology). Design and optimisation of an additively manufactured patient-specific partial m	imaging_dataset	n	y	y	u	u	y
Suchyta et al. 2020. Journal of Clinical and Translational Science. doi:10.1017/cts.2020.325	cadaver_phantom	u	y	n	n	u	y
Kargarnejad et al. 2020. . doi:10.1109/icbme51989.2020.9319325	in_silico	n	n	n	u	u	n
Seebach et al. 2020. Journal of Medical Devices. doi:10.1115/1.4049193	bench	u	y	n	u	u	y
Wang et al. 2020. Applied Sciences. doi:10.3390/app10144738	in_silico	n	y	u	u	u	n
Dahake et al. 2020. The international journal of medical robotics + computer assisted surgery : MRCAS. PMID 31675463	cadaver_phantom	u	y	n	u	u	u
Cheng et al. 2020. Journal of the mechanical behavior of biomedical materials. PMID 32279058	in_silico	n	n	y	u	u	n
Li et al. 2020. Journal of the mechanical behavior of biomedical materials. PMID 32279847	bench	y	y	n	u	u	n
Katschnig et al. 2020. Polymers. PMID 32764496	cadaver_phantom	n	y	n	y	u	n
Liu et al. 2020. ACS biomaterials science & engineering. PMID 33449664	bench	y	y	u	u	u	y
Park et al. 2021. Journal of Computational Design and Engineering. doi:10.1093/jcde/qwab047	in_silico	n	n	u	u	u	n
Cholkar et al. 2021. ESAFORM 2021. doi:10.25518/esaform21.756	in_silico	n	n	y	y	n	n
Song et al. 2021. Journal of the mechanical behavior of biomedical materials. PMID 33310682	bench	n	n	y	u	u	y
Guo et al. 2021. Experimental and therapeutic medicine. PMID 33732321	bench	y	y	n	y	n	y
Lang et al. 2021. PloS one. PMID 34101755	cadaver_phantom	y	y	n	y	n	y
Pinheiro et al. 2021. Scientific reports. PMID 34234245	in_silico	n	n	y	y	n	n
Carnicero et al. 2021. Scientific reports. PMID 34321582	in_silico	n	n	y	y	n	n
Sharma et al. 2021. Journal of clinical medicine. PMID 34441859	bench	n	n	y	y	n	y
Lin et al. 2021. BMC oral health. PMID 34521397	in_silico	n	n	y	y	n	n
Zhong et al. 2021. Journal of the mechanical	in_sil	n	n	u	u	u	n

Study	Tier	Independent	Ground truth	Uncertainty	Reproducible inputs	Files available	Multiple anatomies
behavior of biomedical materials. PMID 34563812	ico						
Kargarnejad et al. 2021. Computer methods in biomechanics and biomedical engineering. PMID 34657530	in_silico	n	n	y	u	u	n
Shaikh et al. 2021. Dentistry journal. PMID 34677171	bench	y	y	n	y	n	n
Shi et al. 2021. Computers in biology and medicine. PMID 34741907	in_silico	u	n	n	u	u	u
Karlagina et al. 2022. . doi:10.21203/rs.3.rs-2252495/v1	bench	n	y	y	y	n	y
Tiwari et al. 2022. Applied Sciences. doi:10.3390/app12063003	in_silico	n	y	u	u	u	n
De Moor et al. 2022. International journal of oral and maxillofacial surgery. PMID 34059405	in_silico	n	n	y	y	n	n
Gass et al. 2022. Journal of anatomy. PMID 34658032	imaging_dataset	y	y	y	y	n	y
Lin et al. 2022. European journal of oral sciences. PMID 35014088	in_silico	u	n	y	u	u	u
Dot et al. 2022. European radiology. PMID 35037088	imaging_dataset	y	y	n	y	y	y
Shen et al. 2022. Materials (Basel, Switzerland). PMID 35057294	bench	n	y	y	y	n	n
Lin et al. 2022. International journal of bioprinting. PMID 35187275	bench	y	y	y	y	n	y
Cheng et al. 2022. Technology and health care : official journal of the European Society for Engineering and Medicine. PMID 35275582	in_silico	n	n	y	u	u	n
Chen et al. 2022. BioMed research international. PMID 35342763	bench	y	y	y	y	n	y
Steiert et al. 2022. Neurosurgical review. PMID 35441994	cadaver_phantom	y	y	n	y	n	y
Li et al. 2022. ACS biomaterials science & engineering. PMID 35443132	bench	u	y	n	u	u	u
Van Kootwijk et al. 2022. Journal of the mechanical behavior of biomedical materials. PMID 35660552	bench	y	y	n	u	u	u
Ingawale et al. 2022. Materials (Basel, Switzerland). PMID 35744401	in_silico	n	n	y	y	u	n
Zhong et al. 2022. Computer methods and programs in biomedicine. PMID 35872386	in_silico	n	n	n	u	u	n
Tran et al. 2022. Journal of clinical medicine. PMID 36142953	cadaver_phantom	y	y	y	n	y	n
Merema et al. 2022. Journal of personalized medicine. PMID 36143224	cadaver_phantom	y	y	y	y	n	y
Nimmawitt et al. 2022. Materials (Basel, Switzerland). PMID 36143689	bench	y	y	y	y	n	n
Rahimov et al. 2022. Annals of maxillofacial surgery. PMID 36199454	cadaver_phantom	n	y	y	y	n	y
Wan et al. 2022. Journal of the mechanical behavior of biomedical materials. PMID	in_sil	n	n	y	u	u	n

Study	Tier	Independent	Ground truth	Uncertainty	Reproducible inputs	Files available	Multiple anatomies
36302272	ico						
Ruf et al. 2022. Frontiers in bioengineering and biotechnology. PMID 36466355	in_silico	n	n	y	y	n	n
Zheng et al. 2022. Frontiers in bioengineering and biotechnology. PMID 36507282	bench	n	n	y	y	n	y
Schottey et al. 2023. Annals of 3D Printed Medicine. doi:10.1016/j.stlm.2023.100107	in_silico	n	n	y	u	u	n
Abed Hashmi et al. 2023. International Journal of Science and Research (IJSR). doi:10.21275/sr23722105819	in_silico	n	n	y	y	n	n
Dutta et al. 2023. Medical engineering & physics. PMID 36792242	in_silico	n	n	y	u	u	n
Yang et al. 2023. Frontiers in bioengineering and biotechnology. PMID 36845197	in_silico	n	n	y	y	n	n
Moiduddin et al. 2023. Polymers. PMID 36850170	cadaver_phantom	n	y	y	y	n	n
Tel et al. 2023. Scientific reports. PMID 37495645	imaging_dataset	u	n	y	y	n	y
Manekar et al. 2023. Journal of maxillofacial and oral surgery. PMID 37534346	in_silico	n	n	n	u	u	n
Banerjee et al. 2023. Journal of biomedical materials research. Part B, Applied biomaterials. PMID 37624362	in_silico	u	u	y	u	u	u
Van Kootwijk et al. 2023. Journal of the mechanical behavior of biomedical materials. PMID 37678107	cadaver_phantom	u	y	y	u	u	y
Zhong et al. 2023. Journal of the mechanical behavior of biomedical materials. PMID 37875041	in_silico	n	n	y	u	u	n
Mineo et al. 2024. IOP Conference Series Materials Science and Engineering. doi:10.1088/1757-899x/1306/1/012017	bench	y	u	y	u	u	u
Nourishirazi et al. 2024. Journal of Craniomaxillofacial Research. doi:10.18502/jcr.v10i4.15313	in_silico	n	n	n	u	u	n
Demir et al. 2024. Computer methods in biomechanics and biomedical engineering. PMID 36920278	in_silico	n	n	n	u	u	n
Ruf et al. 2024. Computers in biology and medicine. PMID 38064852	in_silico	n	n	n	u	u	n
Xue et al. 2024. BMC oral health. PMID 38233785	in_silico	n	n	y	y	n	n
Chen et al. 2024. Journal of dental sciences. PMID 38303793	in_silico	n	n	y	y	n	n
Longhitano et al. 2024. Journal of the mechanical behavior of biomedical materials. PMID 38340477	in_silico	n	n	n	u	u	n
Maintz et al. 2024. 3D printing in medicine. PMID 38639834	cadaver_phantom	n	n	n	y	u	y
Oberoi et al. 2024. Dental materials : official publication of the Academy of Dental Materials. PMID 39084954	in_silico	n	n	y	u	u	n
Datarkar et al. 2024. National journal of	in_sil	n	n	n	y	n	n

Study	Tier	Independent	Ground truth	Uncertainty	Reproducible inputs	Files available	Multiple anatomies
maxillofacial surgery. PMID 39234141	ico						
Parhiz et al. 2024. BioMed research international. PMID 39238768	in_silico	n	n	y	y	n	n
Sabelis et al. 2024. Journal of cranio-maxillo-facial surgery : official publication of the European Association for Cranio-Maxillo-Facial Surgery. PMID 39266434	imaging_dataset	y	n	n	u	u	y
C.-H. et al. 2025. International Journal of Bioprinting. doi:10.36922/ijb025260250	bench	y	y	y	u	u	y
Dogan et al. 2025. Computers in biology and medicine. PMID 39602978	in_silico	n	n	u	u	u	y
Merema et al. 2025. Scientific reports. PMID 39753636	bench	y	y	y	y	u	n
Dubron et al. 2025. Scientific reports. PMID 39814827	imaging_dataset	y	y	y	y	u	y
Nayak et al. 2025. 3D printing in medicine. PMID 40067641	in_silico	n	n	n	n	u	u
Mejía Rodríguez et al. 2025. The Journal of prosthetic dentistry. PMID 40121172	in_silico	n	n	y	u	u	n
Akkoyun et al. 2025. Medicina oral, patologia oral y cirugia bucal. PMID 40121675	in_silico	n	n	y	y	n	n
Cheng et al. 2025. International journal of oral and maxillofacial surgery. PMID 40199670	in_silico	n	n	n	u	u	y
Sahni et al. 2025. Proceedings of the Institution of Mechanical Engineers. Part H, Journal of engineering in medicine. PMID 40326366	bench	u	y	n	u	u	y
Kasapoglu et al. 2025. International journal of medical sciences. PMID 40386052	in_silico	n	n	n	y	n	n
Andres et al. 2025. Bioengineering (Basel, Switzerland). PMID 40428165	bench	y	y	y	y	n	y
Ruf et al. 2025. Computers in biology and medicine. PMID 40614522	in_silico	u	n	n	u	u	n
Hijazi et al. 2025. Journal of the mechanical behavior of biomedical materials. PMID 40753708	in_silico	n	n	y	y	u	n
Roshdy et al. 2025. Computers in biology and medicine. PMID 40779986	in_silico	n	n	n	y	u	n
Chang et al. 2025. Frontiers in bioengineering and biotechnology. PMID 40909220	bench	y	y	y	y	n	y
Zhong et al. 2025. Journal of biomechanical engineering. PMID 40913392	in_silico	n	n	y	y	n	n
Beisekenov et al. 2025. Journal of functional biomaterials. PMID 41003421	bench	y	y	y	y	y	y
Orassi et al. 2025. Clinical oral investigations. PMID 41114876	in_silico	n	y	y	u	u	n
Yüceer-Çetiner et al. 2025. Materials (Basel, Switzerland). PMID 41227783	in_silico	n	n	n	y	u	n
Sagar et al. 2025. Journal of cranio-maxillo-facial surgery : official publication of the European Association for Cranio-Maxillo-Facial Surgery. PMID 41266230	in_silico	n	n	n	y	u	n
Canko et al. 2025. BMC oral health. PMID 41272638	in_silico	n	n	n	y	u	n

Study	Tier	Independent	Ground truth	Uncertainty	Reproducible inputs	Files available	Multiple anatomies
Ampaites et al. 2026. Results in Engineering. doi:10.1016/j.rineng.2026.110026	bench	y	y	y	u	u	y
Zhong et al. 2026. Virtual and Physical Prototyping. doi:10.1080/17452759.2026.2653335	cadaver_phantom	n	n	y	u	u	y
Gürlek et al. 2026. Journal of Craniofacial Surgery. doi:10.1097/scs.0000000000013117	in_silico	n	n	n	u	u	n
Etik et al. 2026. . doi:10.21203/rs.3.rs-8848013/v1	in_silico	n	n	y	y	n	n
Mejía Rodríguez et al. 2026. The Journal of prosthetic dentistry. PMID 40930860	in_silico	n	n	y	u	u	y
Zhong et al. 2026. Annals of biomedical engineering. PMID 41191275	bench	y	y	n	y	n	n
Wood-Bailey et al. 2026. Archives of oral biology. PMID 41218231	in_silico	n	n	y	y	y	n
Türk et al. 2026. Journal of stomatology, oral and maxillofacial surgery. PMID 41285248	in_silico	u	n	n	u	u	u
Wang et al. 2026. Dental materials : official publication of the Academy of Dental Materials. PMID 41554658	bench	n	y	n	y	u	u
Pulino et al. 2026. Craniomaxillofacial trauma & reconstruction. PMID 41725913	in_silico	n	n	y	y	n	n
Sun et al. 2026. Journal of cranio-maxillo-facial surgery : official publication of the European Association for Cranio-Maxillo-Facial Surgery. PMID 41759452	in_silico	n	n	n	u	u	n
Ruf et al. 2026. Frontiers in bioengineering and biotechnology. PMID 41768128	in_silico	n	n	y	y	u	n
Oteiza et al. 2026. Craniomaxillofacial trauma & reconstruction. PMID 41892802	cadaver_phantom	y	y	y	y	n	y
Etik et al. 2026. Scientific reports. PMID 42032041	in_silico	n	n	y	y	n	n
Zhang et al. 2026. Scientific reports. PMID 42045386	bench	y	y	n	y	n	y
Chandra et al. 2026. Journal of cranio-maxillo-facial surgery : official publication of the European Association for Cranio-Maxillo-Facial Surgery. PMID 42160799	in_silico	u	n	y	u	u	u
Juneja et al. 2026. Bioengineering (Basel, Switzerland). PMID 42351864	bench	y	y	y	y	u	y
Tang et al. 2026. BMC oral health. PMID 42437915	in_silico	n	n	y	u	n	n
Blatt et al. 2026. Journal of cranio-maxillo-facial surgery : official publication of the European Association for Cranio-Maxillo-Facial Surgery. PMID 42551059	in_silico	n	n	n	u	u	y
Abd Elfattah et al. 2026. BMC oral health. PMID 42568091	in_silico	y	n	n	y	n	n
Carlos et al. 2026. Digital Repository (Polytechnic University of Cartagena). Design, optimization, and virtual validation of personalized mandibular prosthes	in_silico	u	y	n	n	u	y
Zanotti et al. 2015. Acta neurochirurgica. PMID 25588747	in_silico	n	n	y	u	u	n
Tan et al. 2016. Journal of neurosurgery.	cadav	n	y	u	u	u	n

Study	Tier	Independent	Ground truth	Uncertainty	Reproducible inputs	Files available	Multiple anatomies
PMID 26566203	er_phantom						
Díaz et al. 2017. IFMBE Proceedings. doi:10.1007/978-981-10-4086-3_102	in_silico	n	n	y	y	n	n
Phanindra Bogu et al. 2017. Medical & biological engineering & computing. PMID 28474182	in_silico	n	n	y	u	u	n
Bogu et al. 2017. Acta of bioengineering and biomechanics. PMID 28552926	in_silico	n	n	y	u	u	n
Chen et al. 2017. Scientific reports. PMID 28646207	in_silico	n	y	y	n	n	y
Morais et al. 2018. Portuguese National Funding Agency for Science, Research and Technology (RCAAP Project by FCT). Automated computer-aided design of cranial implants: a deep learning approach	imaging_dataset	u	y	y	u	n	y
Moiduddin et al. 2019. Applied Sciences. doi:10.3390/app9122513	bench	y	y	u	u	u	u
Oldřich et al. 2019. arXiv (Cornell University). doi:10.48550/arxiv.1911.08805	imaging_dataset	u	y	u	u	u	u
Li et al. 2021. arXiv (Cornell University). doi:10.48550/arxiv.2108.05269	imaging_dataset	u	u	u	y	y	y
De Santis et al. 2021. Materials (Basel, Switzerland). PMID 33401673	bench	y	y	n	y	u	y
Huys et al. 2021. World neurosurgery. PMID 33529765	in_silico	u	n	n	u	u	y
Sharma et al. 2021. Frontiers in physiology. PMID 33897455	bench	n	y	n	y	n	n
Li et al. 2021. Medical image analysis. PMID 34340106	imaging_dataset	y	y	u	u	y	y
Liu et al. 2021. IEEE robotics and automation letters. PMID 34395869	cadaver_phantom	y	y	n	y	n	y
Sharma et al. 2021. International journal of molecular sciences. PMID 34445228	bench	n	y	n	y	n	y
Mattavelli et al. 2021. World neurosurgery. PMID 34450324	cadaver_phantom	y	y	y	u	u	y
Montemurro et al. 2021. International journal of environmental research and public health. PMID 34639256	cadaver_phantom	y	y	n	y	n	n
Jindal et al. 2022. Proceedings of the Institution of Mechanical Engineers Part L Journal of Materials Design and Applications. doi:10.1177/14644207221104875	in_silico	n	n	n	u	u	u
Msallem et al. 2022. Materials (Basel, Switzerland). PMID 35269201	bench	y	y	n	y	n	y
Mian et al. 2022. Polymers. PMID 35335596	bench	n	y	n	y	n	n
Sulakhe et al. 2022. Annual International Conference of the IEEE Engineering in Medicine and Biology Society. IEEE Engineering in Medicine and Biology Society. Annual International Conference. PMID 36085744	in_silico	u	u	u	u	u	u

Study	Tier	Independent	Ground truth	Uncertainty	Reproducible inputs	Files available	Multiple anatomies
Wodzinski et al. 2022. Computer methods and programs in biomedicine. PMID 36257198	imaging_dataset	y	y	n	y	y	y
Pisaneschi et al. 2023. Progress in Additive Manufacturing. doi:10.1007/s40964-023-00452-5	bench	y	y	y	y	n	y
Jindal et al. 2023. Proceedings of the Institution of Mechanical Engineers Part L Journal of Materials Design and Applications. doi:10.1177/14644207231155761	in_silico	n	n	y	u	u	n
Tantisatirapong et al. 2023. Scientific reports. PMID 36801943	imaging_dataset	u	y	n	n	u	y
Matin-Mann et al. 2023. Journal of imaging. PMID 36826970	cadaver_phantom	y	y	n	y	n	y
Marzola et al. 2023. Computer methods and programs in biomedicine. PMID 37023601	imaging_dataset	u	u	n	u	u	y
Sharma et al. 2023. Journal of clinical medicine. PMID 37048579	cadaver_phantom	y	y	n	y	n	y
Fortunato et al. 2023. Bioengineering (Basel, Switzerland). PMID 37237631	cadaver_phantom	y	y	n	y	y	n
Xu et al. 2023. Acta neurochirurgica. PMID 37351673	cadaver_phantom	y	y	n	u	y	y
Singh et al. 2023. Journal of the mechanical behavior of biomedical materials. PMID 37544200	cadaver_phantom	n	n	y	u	u	y
Jindal et al. 2023. Journal of the mechanical behavior of biomedical materials. PMID 37556925	in_silico	n	n	y	u	u	n
Moncayo-Matute et al. 2023. Journal of the mechanical behavior of biomedical materials. PMID 37562162	in_silico	n	n	y	u	u	n
Moiduddin et al. 2023. Journal of functional biomaterials. PMID 37623673	cadaver_phantom	n	y	n	y	n	n
Haque et al. 2023. Biomimetics (Basel, Switzerland). PMID 37887630	in_silico	n	n	y	y	u	n
Wu et al. 2023. Frontiers in bioengineering and biotechnology. PMID 38149174	imaging_dataset	y	y	n	y	n	y
S et al. 2024. Procedia Computer Science. doi:10.1016/j.procs.2024.04.312	imaging_dataset	y	y	u	u	u	y
Kesornsri et al. 2024. IEEE Access. doi:10.1109/access.2024.3415173	imaging_dataset	u	y	n	u	u	y
Mejía Rodríguez et al. 2024. Designs. doi:10.3390/designs8020031	in_silico	u	n	u	n	u	n
Moya et al. 2024. Technologies. doi:10.3390/technologies12080125	bench	y	u	u	u	u	y
Zhang et al. 2024. Journal of materials chemistry. B. PMID 39246118	bench	u	y	u	u	u	u
Wodzinski et al. 2024. Computers in	imaging_da	y	y	n	u	u	y

Study	Tier	Independent	Ground truth	Uncertainty	Reproducible inputs	Files available	Multiple anatomies
biology and medicine. PMID 39265478	taset						
Shash et al. 2024. BMC musculoskeletal disorders. PMID 39563300	in_silico	y	y	y	y	n	n
Wodzinski et al. 2024. Annual International Conference of the IEEE Engineering in Medicine and Biology Society. IEEE Engineering in Medicine and Biology Society. Annual International Conference. PMID 40040189	imaging_dataset	u	y	u	u	u	y
Pisaneschi et al. 2025. Progress in Additive Manufacturing. doi:10.1007/s40964-025-01098-1	bench	y	y	y	y	y	y
Jindal et al. 2025. Proceedings of the Institution of Mechanical Engineers Part L Journal of Materials Design and Applications. doi:10.1177/14644207241312947	in_silico	n	n	y	u	u	n
Rasheed et al. 2025. Dijlah Journal of Medical Sciences P-ISSN 3078-3178 E-ISSN 3078-8625. doi:10.65204/djms-multi-factor-biomechanical	in_silico	n	n	y	n	n	u
Maintz et al. 2025. International journal of computer assisted radiology and surgery. PMID 39671058	cadaver_phantom	y	y	n	y	n	y
Wodzinski et al. 2025. Computer methods and programs in biomedicine. PMID 40009971	imaging_dataset	u	y	u	u	n	y
Juneja et al. 2025. Computers in biology and medicine. PMID 40483854	imaging_dataset	y	y	n	y	u	y
Ekşi Altan et al. 2025. Proceedings of the Institution of Mechanical Engineers. Part H, Journal of engineering in medicine. PMID 40876815	bench	y	y	n	u	u	n
Salem et al. 2026. IEEE Access. doi:10.1109/access.2026.3663618	bench	y	y	n	u	u	y
Nguyen et al. 2026. Applied Sciences. doi:10.3390/app16104763	imaging_dataset	u	y	y	y	u	y
Guo et al. 2026. University of Alberta Library. doi:10.7939/83976	bench	u	y	n	u	u	y
Zouggar et al. 2026. Journal of the mechanical behavior of biomedical materials. PMID 41418444	bench	u	y	u	u	u	u
Choudhary et al. 2026. Journal of materials science. Materials in medicine. PMID 42262441	bench	n	y	n	y	u	y
Saravana Kumar et al. 2017. Proceedings of the Institution of Mechanical Engineers. Part H, Journal of engineering in medicine. PMID 28056711	in_silico	n	n	n	u	u	n
Dharme et al. 2017. The International journal of artificial organs. PMID 28106226	bench	y	y	y	u	u	y
Abdelaal et al. 2019. The International journal of artificial organs. PMID 30537876	in_silico	n	n	u	u	u	n
Леонид et al. 2020. IOP Conference Series Materials Science and Engineering. doi:10.1088/1757-899x/986/1/012035	in_silico	n	n	y	u	u	n
Marta et al. 2020. Repositori digital de la UPF (Universitat Pompeu Fabra). General	imaging_da	u	y	n	n	u	y

Study	Tier	Independent	Ground truth	Uncertainty	Reproducible inputs	Files available	Multiple anatomies
deformable 3D hip prosthesis model creation for patient-specific implant	taset						
Corona-Castuera et al. 2021. ACS omega. PMID 33748604	bench	n	y	y	y	n	y
Solórzano-Requejo et al. 2022. Materials (Basel, Switzerland). PMID 35057160	bench	n	n	y	y	y	y
Snekhalatha et al. 2023. Biomedical Engineering: Applications, Basis and Communications. doi:10.4015/s1016237223500175	in_silico	n	n	y	u	u	n
Sun et al. 2023. Computer methods in biomechanics and biomedical engineering. PMID 36036151	in_silico	n	n	y	u	n	n
Singh et al. 2023. Medical engineering & physics. PMID 36965999	bench	y	y	n	u	u	n
Rana et al. 2023. Journal of the mechanical behavior of biomedical materials. PMID 37244075	bench	u	y	n	u	u	u
G. et al. 2024. International Journal of Civil Environmental and Agricultural Engineering. doi:10.34256/ijceae2324	in_silico	n	n	u	u	n	u
Scherb et al. 2024. DS 133: Proceedings of the 35th Symposium Design for X (DFX2024). doi:10.35199/dfx2024.02	in_silico	n	n	y	y	n	n
Müller et al. 2024. Scientific reports. PMID 38459092	bench	n	y	y	y	u	n
Zhao et al. 2024. Materials (Basel, Switzerland). PMID 39410466	bench	y	y	y	y	n	y
Dehaghani et al. 2025. . doi:10.25904/1912/5981	bench	y	y	y	u	u	y
Alkentar et al. 2025. Applied Sciences. doi:10.3390/app15073522	in_silico	u	y	u	u	u	u
Mao et al. 2025. Journal of the mechanical behavior of biomedical materials. PMID 40494102	bench	u	u	n	u	u	u
Banis et al. 2025. Journal of experimental orthopaedics. PMID 41180554	cadaver_phantom	y	y	y	y	n	y
Safavi et al. 2025. Journal of orthopaedic surgery and research. PMID 41239450	bench	n	n	y	y	u	n
Ebrahimzadeh Dehaghani et al. 2026. Computers in biology and medicine. PMID 42308584	in_silico	n	n	u	u	u	n
Yáñez González-Cuéllar et al. 2026. Computer methods and programs in biomedicine. PMID 42320193	in_silico	y	y	y	u	u	y
RONG et al. 2015. Journal of Mechanics in Medicine and Biology. doi:10.1142/s0219519415400527	in_silico	n	n	u	u	u	n
Koh et al. 2017. Bone & joint research. PMID 28947604	in_silico	y	y	n	y	n	n
Kang et al. 2017. Journal of the American Academy of Orthopaedic Surgeons. Global research & reviews. PMID 30211354	in_silico	n	n	y	y	n	n
Khosravipour et al. 2018. Proceedings of the Institution of Mechanical Engineers. Part H, Journal of engineering in medicine. PMID 29191076	bench	n	y	y	y	n	n
Kang et al. 2018. Bone & joint research.	in_sil	y	y	y	y	n	n

Study	Tier	Independent	Ground truth	Uncertainty	Reproducible inputs	Files available	Multiple anatomies
PMID 29305427	ico						
Koh et al. 2018. Journal of experimental orthopaedics. PMID 30578465	in_silico	y	y	n	y	n	n
Hosni et al. 2019. UNC Libraries. doi:10.17615/s5er-hk90	in_silico	n	n	n	u	u	n
Koh et al. 2019. Artificial organs. PMID 30375662	in_silico	u	u	n	u	u	n
Koh et al. 2019. Bone & joint research. PMID 30997041	in_silico	n	n	y	y	n	n
Koh et al. 2019. Journal of experimental orthopaedics. PMID 31161463	in_silico	y	y	n	u	u	n
Wang et al. 2019. Proceedings of the Institution of Mechanical Engineers. Part H, Journal of engineering in medicine. PMID 31319772	in_silico	n	n	y	u	u	n
Saeidi et al. 2019. Tuwhera (Auckland University of Technology). Numerical and Experimental Investigation of a New Load-sharing Implant for Early	cadaver_phantom	n	y	n	u	u	y
Koh et al. 2020. BioMed research international. PMID 32258105	in_silico	u	y	y	y	n	n
Wang et al. 2020. Proceedings of the Institution of Mechanical Engineers. Part H, Journal of engineering in medicine. PMID 32723134	in_silico	y	y	y	y	n	n
Kilian et al. 2021. Bio-Design and Manufacturing. doi:10.1007/s42242-021-00153-4	cadaver_phantom	n	n	n	y	n	n
Wang et al. 2021. Computer methods in biomechanics and biomedical engineering. PMID 32840135	in_silico	n	n	y	u	u	u
Balato et al. 2021. Journal of personalized medicine. PMID 34683181	imaging_dataset	y	n	n	y	n	y
MacLeod et al. 2021. Communications medicine. PMID 36737534	in_silico	y	y	y	y	y	y
Kanagalingam et al. 2022. Progress in Additive Manufacturing. doi:10.1007/s40964-022-00342-2	bench	y	y	n	y	n	y
Xiang et al. 2022. . doi:10.21203/rs.3.rs-1599305/v1	in_silico	n	n	y	y	n	n
Korkmaz et al. 2022. Biomedizinische Technik. Biomedical engineering. PMID 35727116	bench	u	y	y	u	u	n
Alshewaier et al. 2023. Journal of Mechanics in Medicine and Biology. doi:10.1142/s021951942340016x	in_silico	u	n	y	u	u	y
Kladovasilakis et al. 2023. Technologies. doi:10.3390/technologies11020058	in_silico	n	n	u	u	u	n
Burge et al. 2023. Scientific reports. PMID 36849812	imaging_dataset	y	y	y	y	u	y
Grothues et al. 2023. Biomedizinische Technik. Biomedical engineering. PMID 37185164	imaging_dataset	u	y	n	u	u	y
Zhang et al. 2023. Materials (Basel, Switzerland). PMID 37445036	bench	y	y	y	y	n	y
Zhang et al. 2024. . doi:10.21203/rs.3.rs-4729506/v1	in_silico	n	n	y	y	n	n

Study	Tier	Independent	Ground truth	Uncertainty	Reproducible inputs	Files available	Multiple anatomies
S. et al. 2024. . doi:10.62791/20368	in_silico	n	n	y	u	n	n
Assink et al. 2024. European journal of trauma and emergency surgery : official publication of the European Trauma Society. PMID 37391531	cadaver_phantom	y	y	y	y	n	y
DeCarvalho et al. 2024. Medical & biological engineering & computing. PMID 38433178	in_silico	n	n	y	u	y	n
Nourishirazi et al. 2024. Journal of orthopaedics. PMID 38983674	in_silico	u	n	y	u	u	y
Piovan et al. 2024. Journal of orthopaedic surgery and research. PMID 39068461	in_silico	n	n	y	y	u	n
Ovcharenko et al. 2025. Mechanics and Advanced Technologies. doi:10.20535/2521-1943.2025.9.2(105).336223	in_silico	n	n	n	u	u	n
Shang et al. 2025. . doi:10.21203/rs.3.rs-6931418/v1	bench	n	n	y	y	n	n
Tanveer et al. 2025. Applied Sciences. doi:10.3390/app152212099	in_silico	n	n	n	u	u	u
Alshewaier et al. 2025. PloS one. PMID 39899500	in_silico	n	n	y	y	u	n
Guezou-Philippe et al. 2025. PloS one. PMID 40561157	imaging_dataset	y	y	n	y	n	y
Sharma et al. 2026. Proceedings of the Institution of Mechanical Engineers. Part H, Journal of engineering in medicine. PMID 42169235	bench	u	y	n	u	u	u
Wilk et al. 2017. American Journal of Engineering and Applied Sciences. doi:10.3844/ajeassp.2017.13.19	bench	y	y	y	y	n	y
Fan et al. 2018. Proceedings of the Institution of Mechanical Engineers. Part H, Journal of engineering in medicine. PMID 29216793	in_silico	u	n	y	u	u	n
Zhang et al. 2019. Computer methods in biomechanics and biomedical engineering. PMID 31304779	bench	u	y	n	u	u	u
Zhu et al. 2020. . doi:10.21203/rs.2.23817/v1	in_silico	n	n	y	y	u	n
Wong et al. 2020. Applied Sciences. doi:10.3390/app10124098	in_silico	n	n	y	u	u	n
Soni et al. 2020. Indian journal of orthopaedics. PMID 32257031	in_silico	n	n	n	y	n	n
Guoqing et al. 2021. Journal of the mechanical behavior of biomedical materials. PMID 33652236	bench	u	y	n	u	u	u
Wong et al. 2021. Journal of orthopaedic translation. PMID 33981572	in_silico	n	y	y	y	n	n
Wu et al. 2021. Biosensors. PMID 35049632	bench	y	y	y	y	n	y
Al-Tamimi et al. 2022. Applied Sciences. doi:10.3390/app122010569	in_silico	n	n	y	u	u	n
Liu et al. 2022. Medical & biological engineering & computing. PMID 35237925	in_silico	u	n	y	u	u	y
Wu et al. 2022. Journal of biomedical materials research. Part B, Applied	bench	u	y	n	u	u	y

Study	Tier	Independent	Ground truth	Uncertainty	Reproducible inputs	Files available	Multiple anatomies
biomaterials. PMID 35297154							
Liu et al. 2022. Frontiers in surgery. PMID 36090321	in_silico	n	n	n	y	n	n
He et al. 2023. . doi:10.20944/preprints202307.0273.v1	imaging_dataset	u	y	u	u	u	y
Shams et al. 2023. European journal of orthopaedic surgery & traumatology : orthopedie traumatologie. PMID 35001211	in_silico	u	n	y	u	u	y
Chen et al. 2023. Journal of orthopaedic translation. PMID 36313976	in_silico	y	y	y	y	n	n
Kladovasilakis et al. 2023. Journal of the mechanical behavior of biomedical materials. PMID 36965217	cadaver_phantom	n	y	n	y	n	n
Julie et al. 2024. Orthopaedic Proceedings. doi:10.1302/1358-992x.2024.18.082	bench	u	n	n	u	u	y
García-Ávila et al. 2024. Journal of the mechanical behavior of biomedical materials. PMID 38492502	bench	u	u	u	y	u	n
Seo et al. 2024. Medical engineering & physics. PMID 38906577	bench	n	y	n	u	u	n
Betti et al. 2024. Journal of orthopaedic surgery and research. PMID 39210457	imaging_dataset	u	n	n	y	y	y
Wolf et al. 2024. Materials (Basel, Switzerland). PMID 39274630	bench	n	y	y	y	n	y
Po-Kuei et al. 2025. Materials & Design. doi:10.1016/j.matdes.2025.113668	bench	u	y	n	u	u	y
Oldhoff et al. 2025. European journal of trauma and emergency surgery : official publication of the European Trauma Society. PMID 39856352	cadaver_phantom	y	y	y	y	u	y
Li et al. 2025. Foot and ankle surgery : official journal of the European Society of Foot and Ankle Surgeons. PMID 39947960	in_silico	n	n	y	u	u	n
Li et al. 2025. Journal of biomechanical engineering. PMID 39992375	in_silico	n	n	y	u	u	n
Zeng et al. 2025. European journal of medical research. PMID 40517264	in_silico	y	y	y	y	n	n
Ruan et al. 2025. Journal of orthopaedic surgery and research. PMID 40629372	in_silico	y	y	y	y	n	y
Ead et al. 2026. Clinical biomechanics (Bristol, Avon). PMID 42302371	in_silico	y	y	u	u	u	y
George et al. 2018. . doi:10.1109/raics.2018.8635093	bench	n	n	n	u	u	y
Rana et al. 2021. Proceedings of the Institution of Mechanical Engineers. Part H, Journal of engineering in medicine. PMID 34227419	in_silico	n	n	n	u	u	y
Fuchs et al. 2023. Clinical oral investigations. PMID 36864278	cadaver_phantom	y	y	y	y	n	y
Mazzocchetti et al. 2024. Scientific reports. PMID 39191797	imaging_dataset	n	y	n	y	u	y
Seo et al. 2025. Scientific reports. PMID 41131218	bench	y	y	n	y	n	y
Xu et al. 2020. Computer methods in biomechanics and biomedical engineering.	bench	y	u	n	n	u	u

Study	Tier	Independent	Ground truth	Uncertainty	Reproducible inputs	Files available	Multiple anatomies
PMID 32660275							
Helguero et al. 2022. Procedia CIRP. doi:10.1016/j.procir.2022.06.065	in_silico	u	n	n	u	u	u
Benady et al. 2023. Materials & Design. doi:10.1016/j.matdes.2023.111605	in_silico	u	n	n	u	u	u
Mohseni-Dargah et al. 2024. Journal of the mechanical behavior of biomedical materials. PMID 38237204	cadaver_phantom	y	y	u	u	u	u
Zhou et al. 2025. Computer methods and programs in biomedicine. PMID 40107082	in_silico	u	n	u	u	u	y
Vanden Berghe et al. 2017. Computer methods in biomechanics and biomedical engineering. PMID 27957883	imaging_dataset	u	y	n	u	u	u
Iqbal et al. 2017. Proceedings of the Institution of Mechanical Engineers. Part H, Journal of engineering in medicine. PMID 28639517	in_silico	n	n	y	u	u	n
Леонид et al. 2019. Vibroengineering PROCEDIA. doi:10.21595/vp.2019.20961	in_silico	n	n	n	y	n	n
Barbera et al. 2019. The International journal of artificial organs. PMID 31122108	bench	y	y	y	u	u	u
Iqbal et al. 2019. Medical engineering & physics. PMID 31229384	in_silico	n	n	y	u	u	n
Guo et al. 2019. Zhongguo gu shang = China journal of orthopaedics and traumatology. PMID 31277543	in_silico	n	n	n	u	u	n
Solovev et al. 2020. IOP Conference Series Materials Science and Engineering. doi:10.1088/1757-899x/747/1/012076	in_silico	n	n	y	n	u	n
Meynen et al. 2020. Computer methods in biomechanics and biomedical engineering. PMID 32619099	imaging_dataset	y	y	n	y	n	y
Rijn et al. 2020. Research Repository (Delft University of Technology). Sacral anchoring of the LUMiC prosthesis: Development of a protocol to in vitro	bench	u	y	y	u	u	n
Леонид et al. 2021. Russian journal of biomechanics. doi:10.15593/rjbiomech/2021.4.07	in_silico	n	n	n	u	u	n
Asmaria et al. 2021. Jurnal Penelitian Fisika dan Aplikasinya (JPFA). doi:10.26740/jpfa.v11n1.p83-93	in_silico	n	n	n	y	n	n
Meesters et al. 2021. Journal of personalized medicine. PMID 34442407	cadaver_phantom	y	y	n	y	n	y
Maslov et al. 2021. Materials (Basel, Switzerland). PMID 34832464	in_silico	n	n	y	y	n	n
Kang et al. 2022. Journal of orthopaedics. PMID 36090783	bench	y	y	n	y	n	y
Chai et al. 2022. OTA international : the open access journal of orthopaedic trauma. PMID 36569106	imaging_dataset	n	n	n	n	y	y
Babazadeh-Naseri et al. 2022. Rice Digital Scholarship Archive (Rice University). Computational Framework for Designing Patient-specific, 3D-printable, and Stress	in_silico	n	n	y	u	u	u
Soloviev et al. 2023. Materials (Basel, Switzerland). PMID 36614737	in_silico	n	n	y	y	n	n
Zhu et al. 2023. Medical engineering &	in_sil	n	n	y	u	u	n

Study	Tier	Independent	Ground truth	Uncertainty	Reproducible inputs	Files available	Multiple anatomies
physics. PMID 36792235	ico						
Danielli et al. 2023. Journal of the mechanical behavior of biomedical materials. PMID 36801786	bench	y	y	y	u	u	y
Chai et al. 2023. 3D printing and additive manufacturing. PMID 37886407	imaging_dataset	u	y	n	u	u	y
Lv et al. 2023. Journal of orthopaedic surgery and research. PMID 37936217	in_silico	n	n	y	y	u	n
Babazadeh-Naseri et al. 2023. Medical engineering & physics. PMID 37985018	in_silico	n	n	n	u	u	n
Lee et al. 2024. Journal of orthopaedic research : official publication of the Orthopaedic Research Society. PMID 38669091	in_silico	n	n	n	u	u	n
Garner et al. 2024. Journal of orthopaedic research : official publication of the Orthopaedic Research Society. PMID 39004739	in_silico	u	n	y	u	u	y
Zhu et al. 2024. Frontiers in bioengineering and biotechnology. PMID 39234264	in_silico	y	y	y	y	n	n
Sun et al. 2024. Scientific reports. PMID 39242632	in_silico	n	n	y	y	n	n
Javier et al. 2025. Journal of Orthopedic Research and Therapy. doi:10.29011/2575-8241.001385	cadaver_phantom	y	y	y	n	n	y
Zhu et al. 2025. Journal of orthopaedic surgery and research. PMID 39856781	in_silico	n	n	y	y	u	y
Ceddia et al. 2025. Materials (Basel, Switzerland). PMID 40141579	in_silico	n	n	n	y	n	n
Li et al. 2025. Bone & joint research. PMID 41186144	in_silico	n	n	y	y	u	n
Fritz et al. 2026. Journal of Medical Devices. doi:10.1115/1.4071170	bench	y	y	y	u	n	u
Oldhoff et al. 2026. Journal of orthopaedic research : official publication of the Orthopaedic Research Society. PMID 40985989	bench	y	y	n	y	n	y
Eraly et al. 2016. Journal of shoulder and elbow surgery. PMID 26700554	cadaver_phantom	y	y	u	u	u	y
Tilton et al. 2020. Additive manufacturing. doi:10.1016/j.addma.2020.101137	bench	y	y	n	u	u	u
György et al. 2021. . doi:10.21203/rs.3.rs-798715/v1	in_silico	n	n	n	y	n	n
Schader et al. 2022. Journal of shoulder and elbow surgery. PMID 34298147	in_silico	n	n	n	u	u	y
Wan et al. 2022. Frontiers in bioengineering and biotechnology. PMID 36532579	in_silico	n	n	y	y	n	n
Capelli et al. 2016. The Spine Journal. doi:10.1016/j.spinee.2016.01.049	in_silico	n	n	y	n	u	n
Zhang et al. 2016. Orthopaedic surgery. PMID 27627721	cadaver_phantom	y	y	y	u	u	y
Wang et al. 2018. Proceedings of the Institution of Mechanical Engineers. Part H, Journal of engineering in medicine. PMID 29383959	in_silico	y	y	y	y	n	n

Study	Tier	Independent	Ground truth	Uncertainty	Reproducible inputs	Files available	Multiple anatomies
Calvo-Echenique et al. 2018. Computer methods and programs in biomedicine. PMID 29903488	in_silico	n	n	y	u	u	n
Biswas et al. 2020. Current medical imaging. PMID 32410539	in_silico	u	n	y	u	u	n
La Barbera et al. 2021. Clinical biomechanics (Bristol, Avon). PMID 33317937	in_silico	n	n	y	u	u	n
Liu et al. 2021. Journal of biomaterials applications. PMID 33349103	in_silico	u	u	u	u	u	n
Wang et al. 2021. Journal of orthopaedic surgery and research. PMID 33761991	in_silico	y	y	y	y	n	n
Kang et al. 2021. Materials science & engineering. C, Materials for biological applications. PMID 34225889	bench	u	y	y	u	u	y
Ren et al. 2021. Zhongguo gu shang = China journal of orthopaedics and traumatology. PMID 34423622	in_silico	n	n	u	u	u	n
Vanaclocha-Saiz et al. 2022. ACS applied bio materials. PMID 35014829	in_silico	y	n	y	y	n	y
Von Atzigen et al. 2022. Medical image analysis. PMID 35139481	cadaver_phantom	u	y	n	u	u	u
Fernandes et al. 2022. Scientific reports. PMID 35869276	cadaver_phantom	n	y	n	y	u	y
Zhao et al. 2022. Medical engineering & physics. PMID 36068031	in_silico	u	n	u	u	u	u
Xu et al. 2022. BMC musculoskeletal disorders. PMID 36329424	in_silico	y	y	y	y	n	n
Gong et al. 2022. Frontiers in bioengineering and biotechnology. PMID 36466328	in_silico	y	y	n	y	n	n
Rossi et al. 2023. Zenodo (CERN European Organization for Nuclear Research). doi:10.5281/zenodo.19542554	cadaver_phantom	y	y	u	u	u	y
Sun et al. 2023. Heliyon. PMID 36747923	in_silico	y	y	y	y	u	n
Wang et al. 2023. Frontiers in bioengineering and biotechnology. PMID 37744254	in_silico	n	n	y	y	u	n
Kang et al. 2024. Frontiers in bioengineering and biotechnology. PMID 38966191	in_silico	n	y	y	y	u	n
Kaye et al. 2024. World neurosurgery. PMID 38996963	cadaver_phantom	u	n	n	u	u	y
Smit et al. 2024. Journal of the mechanical behavior of biomedical materials. PMID 39186906	bench	n	y	y	u	u	y
Smit et al. 2024. Frontiers in bioengineering and biotechnology. PMID 39318669	in_silico	u	y	y	y	n	y
Zhu et al. 2025. Applied Sciences. doi:10.3390/app15105384	in_silico	n	n	u	n	u	n
Jitkla et al. 2025. Computers in biology and medicine. PMID 39700854	in_silico	u	n	u	u	u	u
Yu et al. 2025. Journal of orthopaedic surgery and research. PMID 41023710	in_silico	n	y	y	y	n	y

Study	Tier	Independent	Ground truth	Uncertainty	Reproducible inputs	Files available	Multiple anatomies
Shen et al. 2025. 3D printing in medicine. PMID 41128973	bench	y	y	n	y	n	y
De Cet et al. 2026. Medical & biological engineering & computing. PMID 41838281	in_silico	n	n	y	y	n	n
Zhang et al. 2026. Journal of orthopaedic translation. PMID 42006909	bench	y	y	n	y	n	y
Chen et al. 2026. Orthopaedic surgery. PMID 42165180	in_silico	y	y	n	y	n	n
Du et al. 2026. North American Spine Society journal. PMID 42199851	cadaver_phantom	y	y	n	y	n	y
Willing et al. 2018. Orthopaedic Proceedings. DESIGN OPTIMISATION OF PATIENT-SPECIFIC DISTAL HUMERAL HEMIARTHROPLASTY IMPLANTS	in_silico	n	y	n	y	u	y
Caiti et al. 2019. Medical & biological engineering & computing. PMID 30588576	in_silico	y	n	y	y	n	y
Zhong et al. 2019. Microscopy research and technique. PMID 30629776	in_silico	n	n	n	u	u	n
Luenam et al. 2019. Journal of orthopaedic surgery (Hong Kong). PMID 30798711	cadaver_phantom	u	y	u	u	u	y
Vancleef et al. 2022. Journal of orthopaedic research : official publication of the Orthopaedic Research Society. PMID 34668224	bench	n	y	y	y	n	n
Wang et al. 2023. Zhongguo xiu fu chong jian wai ke za zhi = Zhongguo xiufu chongjian waike zazhi = Chinese journal of reparative and reconstructive surgery. PMID 37460178	cadaver_phantom	n	n	y	u	u	n
Bregoli et al. 2023. Medical engineering & physics. PMID 37536840	cadaver_phantom	u	y	y	u	u	u
Vitković et al. 2023. Materials (Basel, Switzerland). PMID 37570113	cadaver_phantom	n	y	n	y	n	n
Nazlı et al. 2024. Orthopaedic Proceedings. doi:10.1302/1358-992x.2024.18.002	cadaver_phantom	u	y	y	u	u	y
Honigmann et al. 2024. . doi:10.21203/rs.3.rs-4049296/v1	imaging_dataset	n	n	y	n	n	n
Wang et al. 2024. Journal of shoulder and elbow surgery. PMID 38182026	in_silico	y	y	n	u	u	y
Oldhoff et al. 2024. European journal of trauma and emergency surgery : official publication of the European Trauma Society. PMID 38261077	cadaver_phantom	y	y	n	y	n	y
Yurteri et al. 2024. Archives of orthopaedic and trauma surgery. PMID 39008077	in_silico	n	n	y	u	u	n
Jakimiuk et al. 2024. 3D printing in medicine. PMID 39693002	cadaver_phantom	y	y	n	y	n	y
П. et al. 2025. Orthopaedic Proceedings. doi:10.1302/1358-992x.2025.8.050	cadaver_phantom	y	y	y	u	u	y
Honigmann et al. 2025. .	cadaver_ph	n	n	y	y	n	n

Study	Tier	Independent	Ground truth	Uncertainty	Reproducible inputs	Files available	Multiple anatomies
doi:10.21203/rs.3.rs-5401931/v1	antom						
Honigmann et al. 2025. Bioengineering (Basel, Switzerland). PMID 41301138	cadaver_phantom	u	n	y	y	u	n
Xiao et al. 2026. Bone & joint research. PMID 41880204	in_silico	y	y	y	y	u	n

Table S4. Risk of bias per clinical-tier study

Study	Tier	Tool	Judgements	Overall	Support
Ismailov et al. 2026. Zenodo (CERN European Organization for Nuclear Research). doi:10.5281/zenodo.18811308	clinical_comparative	robins_i	serious; low; low; no_information; no_information; moderate; no_information	serious	abstract only; D1 the complexity-group comparison used an unadjusted t-test despite its focus on complex and secondary defects; D2 patients were consecutive; D3 Brown classes explicitly defined the groups; D4 no informat
Block et al. 2024. . doi:10.53846/goediss-10683	clinical_comparative	robins_i	serious; no_information; low; no_information; no_information; moderate; no_information	serious	abstract only; D1 planning procedure assignment was unexplained and no confounder adjustment was reported; D2 recruitment and consecutive inclusion were not described; D3 commercial and in-house procedures were clearly c
Yildirim et al. 2025. Journal of Medicine and Dentistry. doi:10.64951/jmdnt.2026.1.04	clinical_comparative	robins_i	serious; low; low; no_information; no_information; low; no_information	serious	abstract only; D1 sequence was selected by clinical indication without reported confounder adjustment; D2 the cohort was consecutive; D3 sequence groups were explicitly classified; D4 no information on deviations or coin
Ciocca et al. 2015. Journal of cranio-maxillo-facial surgery : official publication of the European Association for Cranio-Maxillo-Facial Surgery. PMID 25434288	clinical_comparative	robins_i	serious; no_information; low; no_information; no_information; moderate; no_information	serious	abstract only; D1 non-randomized groups of five had no baseline comparability assessment or confounder adjustment
Tarsitano et al. 2016. Journal of cranio-maxillo-facial surgery : official publication of the European Association for Cranio-Maxillo-Facial Surgery. PMID 27107476	clinical_comparative	robins_i	serious; no_information; low; no_information; no_information; moderate; no_information	serious	abstract only; D1: CAD/CAM and traditional reconstruction groups were compared without reported baseline comparability or confounding adjustment
Tarsitano et al. 2016. Journal of cranio-maxillo-facial surgery : official publication of the European Association for Cranio-Maxillo-Facial Surgery. PMID 27193477	clinical_comparative	robins_i	serious; no_information; low; no_information; no_information; moderate; no_information	serious	abstract only; D1: two non-randomized cohorts were compared without baseline characteristics or confounding adjustment
De Maesschalck et al. 2017. European archives of oto-rhino-laryngology : official journal of the European Federation of Oto-Rhino-Laryngological Societies (EUFOS) : affiliated with the German Society for Oto-Rhino-Laryngology - Head and Neck Surgery. PMID 27501991	clinical_comparative	robins_i	serious; no_information; low; no_information; no_information; moderate; no_information	serious	abstract only; D1: retrospective CAS and control groups were compared without confounder adjustment
Zimmerer et al. 2016. Journal of cranio-maxillo-facial surgery : official publication of the European Association for Cranio-Maxillo-Facial Surgery. PMID 27519662	clinical_comparative	robins_i	serious; no_information; low; no_information; no_information; moderate; no_information	serious	abstract only; individualized implants overlapped with navigation, making attribution to implant type difficult
Kim et al. 2017. Journal of cranio-maxillo-facial surgery : official publication of the European Association for Cranio-Maxillo-Facial Surgery. PMID	clinical_comparative	robins_i	serious; no_information; low; no_information; no_information; moderate; no_information	serious	abstract only; retrospective RP and conventional groups were compared without reported control of baseline or treatment-selection confounding

Study	Tier	Tool	Judgements	Overall	Support
28434826					
Fan et al. 2017. Graefe's archive for clinical and experimental ophthalmology = Albrecht von Graefes Archiv fur klinische und experimentelle Ophthalmologie. PMID 28786025	clinical_comparative	robins_i	serious; no_information; low; no_information; no_information; moderate; no_information	serious	abstract only; retrospective 3-D-assisted and traditional groups were compared without reported confounding control
Kim et al. 2018. Journal of plastic, reconstructive & aesthetic surgery : JPRAS. PMID 29233510	clinical_comparative	robins_i	serious; no_information; low; no_information; no_information; moderate; no_information	serious	abstract only; intervention assignment followed comminuted versus intact inferomedial strut status, with no confounding adjustment
Liu et al. 2019. The Journal of arthroplasty. PMID 30497901	clinical_comparative	robins_i	serious; no_information; low; no_information; no_information; moderate; no_information	serious	abstract only; non-random allocation of 19 patients per group had no reported adjustment for clinical or tumour-related confounding
Rückschloß et al. 2019. Journal of cranio-maxillo-facial surgery : official publication of the European Association for Cranio-Maxillo-Facial Surgery. PMID 30894302	clinical_comparative	robins_i	serious; no_information; low; no_information; no_information; low; no_information	serious	abstract only; retrospective non-randomised groups of nine had no reported confounding control
Desai et al. 2019. The Journal of craniofacial surgery. PMID 30950944	clinical_comparative	robins_i	serious; no_information; low; no_information; no_information; moderate; no_information	serious	abstract only; D1 the retrospective technique comparison reported no baseline comparability or confounding adjustment
Kim et al. 2019. Annals of plastic surgery. PMID 31082835	clinical_comparative	robins_i	low; no_information; low; low; no_information; low; no_information	low	abstract only; within-patient software comparison limits confounding; interventions were clearly classified; both methods used the same CT images; orbital volumes were objectively measured
Liu et al. 2020. The British journal of oral & maxillofacial surgery. PMID 32156446	clinical_comparative	robins_i	serious; no_information; low; no_information; no_information; moderate; no_information	serious	abstract only; 18 retrospectively selected patients were divided into groups without reported allocation criteria or confounding adjustment
Sigron et al. 2020. Journal of clinical medicine. PMID 32455967	clinical_comparative	robins_i	serious; serious; low; no_information; serious; moderate; no_information	serious	D1: sequential treatment groups differed substantially in baseline orbital volumes without adjustment; D2: only 22 of 30 patients with complete records and imaging were included; D5: eight patients were excluded for inco
Yang et al. 2021. Annals of surgical oncology. PMID 32572853	clinical_comparative	robins_i	serious; serious; low; no_information; serious; moderate; moderate	serious	D1: prospective intervention patients were compared with historical controls without adjusted confounding control and with markedly different CT timing; D2: recruitment periods and post-intervention exclusions differed;
Lee et al. 2020. Plastic and reconstructive surgery. PMID 32590512	clinical_comparative	robins_i	moderate; moderate; low; no_information; no_information; low; no_information	moderate	abstract only; D1: multivariable regression was used but adjusted confounders were not identified; D2: eligibility and participant-selection details were not reported

Study	Tier	Tool	Judgements	Overall	Support
Badiali et al. 2020. Scientific reports. PMID 32770002	clinical_comparative	robins_i	serious; moderate; low; low; low; moderate; moderate	serious	guide design was confounded with study phase because the first 12 patients received design A and the last 10 received split-mouth designs B and C
Rahimov et al. 2020. Annals of maxillofacial surgery. PMID 32855907	clinical_comparative	robins_i	serious; serious; low; no_information; no_information; moderate; no_information	serious	D1: virtual-planning and historical-control groups came from overlapping but different calendar periods without confounding adjustment; D2: eligibility and consecutive inclusion were not reported
Olate et al. 2021. The Journal of craniofacial surgery. PMID 32858611	clinical_comparative	robins_i	serious; no_information; low; no_information; no_information; moderate; no_information	serious	abstract only; D1 stock-versus-PSI assignment was non-randomized with no baseline comparability assessment or confounder adjustment
Van Doremalen et al. 2021. Archives of orthopaedic and trauma surgery. PMID 33128609	clinical_comparative	robins_i	serious; moderate; low; no_information; serious; low; no_information	serious	D1: non-randomised groups with heterogeneous surgeons and no confounding adjustment; D5: one intervention participant was excluded after postoperative infection
Karanxha et al. 2021. Journal of cranio-maxillo-facial surgery : official publication of the European Association for Cranio-Maxillo-Facial Surgery. PMID 33277160	clinical_comparative	robins_i	serious; low; low; no_information; low; moderate; no_information	serious	abstract only; participants were non-randomly divided by transfer method without reported confounding adjustment
Neuhaus et al. 2021. International journal of oral and maxillofacial surgery. PMID 33707038	clinical_comparative	robins_i	no_information; no_information; low; no_information; no_information; low; no_information	low	abstract only; D3: navigation status clearly classified as with or without navigation; D6: outcomes used objective imaging fusion and specified clinical measures
Harding et al. 2022. International journal of oral and maxillofacial surgery. PMID 33941394	clinical_comparative	robins_i	serious; no_information; low; no_information; no_information; moderate; no_information	serious	abstract only; D1 non-randomized custom and conventional plate cohorts had no baseline comparability assessment or confounder adjustment
Knitschke et al. 2021. Cancers. PMID 34208555	clinical_comparative	robins_i	serious; moderate; low; no_information; no_information; moderate; no_information	serious	non-VSP cases were treated in 2002–2015 whereas VSP began in 2015, larger polysegmental defects were more frequent with VSP, and analyses were unadjusted
Sigron et al. 2021. Journal of clinical medicine. PMID 34441806	clinical_comparative	robins_i	moderate; moderate; low; moderate; serious; moderate; no_information	serious	follow-up ranged from one day to almost two years and patients stopped attending when symptom-free, threatening completeness of functional outcomes
Kang et al. 2021. Frontiers in oncology. PMID 34540688	clinical_comparative	robins_i	serious; serious; low; no_information; low; moderate; no_information	serious	VSP selection was unadjusted for confounding; patients whose flap did not survive were excluded after intervention
Möllmann et al. 2021. Frontiers in oncology. PMID 34900674	clinical_comparative	robins_i	serious; moderate; low; no_information; serious; moderate; no_information	serious	Treatment choice depended on surgeon discretion and case complexity in a heterogeneous cohort without confounding adjustment; the key accuracy comparison used t(56) from 94 patients without reporting reasons

Study	Tier	Tool	Judgements	Overall	Support
					for missing
Timoshchuk et al. 2022. Journal of oral and maxillofacial surgery : official journal of the American Association of Oral and Maxillofacial Surgeons. PMID 35007501	clinical_comparative	robins_i	serious; no_information; low; no_information; no_information; moderate; no_information	serious	abstract only; retrospective implant groups compared using unadjusted Fisher and Wilcoxon tests
Pu et al. 2021. Frontiers in oncology. PMID 35059310	clinical_comparative	robins_i	serious; moderate; low; no_information; moderate; moderate; no_information	serious	Despite matching on sex, age, anthropometrics, tumour size, region and stage, retrospective treatment choice and no adjusted analysis left important confounding uncontrolled.
Guo et al. 2022. International orthopaedics. PMID 35061064	clinical_comparative	robins_i	serious; no_information; low; no_information; no_information; moderate; no_information	serious	abstract only; the retrospective nonrandomised groups were compared without matching or confounding adjustment.
Baldia et al. 2022. Acta neurochirurgica. PMID 35107617	clinical_comparative	robins_i	serious; no_information; low; no_information; no_information; moderate; no_information	serious	abstract only; D1 no baseline comparability or adjustment for confounding across the three non-randomised prosthesis groups
Jones et al. 2022. Journal of oral and maxillofacial surgery : official journal of the American Association of Oral and Maxillofacial Surgeons. PMID 35151639	clinical_comparative	robins_i	serious; no_information; low; no_information; no_information; moderate; no_information	serious	abstract only; retrospective groups of 13 versus 69 were compared without reported confounding adjustment
Hu et al. 2022. Journal of orthopaedic surgery and research. PMID 35264178	clinical_comparative	robins_i	serious; serious; low; no_information; low; moderate; no_information	serious	Five prosthesis patients and five controls were nonrandomly selected with matching only on tumour size and no confounding adjustment; controls were retrospectively selected and incomplete follow-up was excluded.
Liokatis et al. 2022. Laryngoscope investigative otolaryngology. PMID 35434332	clinical_comparative	robins_i	serious; low; low; serious; moderate; moderate; moderate	serious	D1: the non-random five-versus-three-patient groups had heterogeneous deformities without confounding adjustment; D4: one PSI placed differently from plan was excluded from the accuracy analysis
Rodríguez-Arias et al. 2022. Journal of personalized medicine. PMID 35743715	clinical_comparative	robins_i	serious; low; low; low; no_information; moderate; moderate	serious	D1: technique choice was associated with malignant etiology and treatment timing, while clinical outcomes were not adequately adjusted for confounding
Chepurnyi et al. 2022. Journal of cranio-maxillo-facial surgery : official publication of the European Association for Cranio-Maxillo-Facial Surgery. PMID 35963834	clinical_comparative	robins_i	serious; serious; low; no_information; no_information; moderate; no_information	serious	abstract only; D1: non-random intervention assignment was unadjusted despite potential baseline differences; D2: automated-group patients and retrospectively selected controls were recruited differently
Cassoni et al. 2022. International journal of environmental research and public health. PMID 36142107	clinical_comparative	robins_i	serious; moderate; low; low; low; moderate; moderate	serious	D1: the retrospective five-per-group comparison did not control for procedure complexity or other confounding
Spalthoff et al. 2022. Frontiers in oncology. PMID 36212406	clinical_comparative	robins_i	serious; serious; low; no_information; no_information; low; no_information	serious	unmeasured confounding was acknowledged and patient variables were not assessed;

Study	Tier	Tool	Judgements	Overall	Support
					multiple-implant reconstructions were excluded because their communication was assumed to be more difficult
Kerkfeld et al. 2022. Journal of personalized medicine. PMID 36294792	clinical_comparative	robins_i	serious; no_information; low; no_information; low; low; no_information	serious	treatment groups differed by laterognathia, syndromic status, and deformity severity, while additional PEEK augmentation was preferentially used for more severe deformities
Knitschke et al. 2022. Cancers. PMID 36497255	clinical_comparative	robins_i	serious; serious; low; no_information; low; moderate; no_information	serious	plate type was confounded with treatment era because PSI replaced conventional plates after 2015; inclusion required successful reconstruction and at least two available scans
Landau Prat et al. 2023. Seminars in ophthalmology. PMID 36639878	clinical_comparative	robins_i	moderate; no_information; low; no_information; no_information; no_information; no_information	moderate	abstract only; retrospective non-random implant selection leaves residual confounding despite regression adjustment
Gong et al. 2023. Journal of surgical oncology. PMID 36825890	clinical_comparative	robins_i	serious; no_information; low; no_information; no_information; moderate; no_information	serious	abstract only; D1 retrospective angle-defined groups of 11 patients each had no stated control of treatment-selection confounding
Kim et al. 2023. BMC oral health. PMID 37138237	clinical_comparative	robins_i	serious; moderate; low; no_information; low; moderate; no_information	serious	D1 intervention assignment was not randomized and no baseline comparability assessment or confounder adjustment was reported
Pietzka et al. 2023. Journal of personalized medicine. PMID 37240877	clinical_comparative	robins_i	serious; moderate; low; no_information; moderate; moderate; no_information	serious	D1 conventional treatment was used in 2010–2014 and PSI in 2015–2019, with no adjustment for temporal or surgeon-related confounding
Pietzka et al. 2023. Journal of personalized medicine. PMID 37241016	clinical_comparative	robins_i	serious; moderate; low; moderate; moderate; moderate; no_information	serious	D1 implant use changed over calendar time, fracture complexity differed, and newer imaging was used more often with PSI without confounding adjustment
Kim et al. 2023. Annals of plastic surgery. PMID 37713150	clinical_comparative	robins_i	serious; no_information; low; no_information; no_information; moderate; no_information	serious	abstract only; D1 retrospective non-random groups of 12 and 23 with no stated control of treatment-selection confounding
Kuehle et al. 2023. Journal of clinical medicine. PMID 37762977	clinical_comparative	robins_i	serious; moderate; low; low; no_information; moderate; moderate	serious	D1 serious: retrospective treatment selection was unadjusted despite planned movement correlating with accuracy deviations
Merta et al. 2024. Journal of cranio-maxillo-facial surgery : official publication of the European Association for Cranio-Maxillo-Facial Surgery. PMID 38129183	clinical_comparative	robins_i	serious; no_information; low; no_information; no_information; moderate; no_information	serious	abstract only; D1 serious: retrospective fixation groups were compared without reported confounder adjustment
Consorti et al. 2024. Journal of cranio-maxillo-facial surgery : official publication of the European Association for Cranio-Maxillo-Facial Surgery. PMID 38368208	clinical_comparative	robins_i	serious; no_information; low; no_information; no_information; moderate; no_information	serious	abstract only; D1 serious: retrospective non-randomised groups with no confounder adjustment reported

Study	Tier	Tool	Judgements	Overall	Support
Krasovsky et al. 2024. Journal of cranio-maxillo-facial surgery : official publication of the European Association for Cranio-Maxillo-Facial Surgery. PMID 38388230	clinical_comparative	robins_i	serious; no_information; low; no_information; no_information; moderate; no_information	serious	abstract only; D1 serious: retrospective treatment allocation with no confounder adjustment reported
Hu et al. 2024. Journal of orthopaedic surgery and research. PMID 38654343	clinical_comparative	robins_i	serious; serious; low; no_information; low; moderate; no_information	serious	Serious confounding because treatment depended on patient preference, cost, and production time without adjustment; groups were retrospectively selected for comparability and complete follow-up
D'Alpaos et al. 2024. Journal of personalized medicine. PMID 38793041	clinical_comparative	robins_i	serious; serious; low; no_information; low; moderate; no_information	serious	Serious confounding from heterogeneous indications without adjustment; control-group selection and eligibility are not described
Zhao et al. 2024. Regenerative biomaterials. PMID 38854680	clinical_comparative	robins_i	serious; serious; low; no_information; serious; moderate; serious	serious	Serious baseline confounding was uncontrolled; seven of 40 enrolled patients were unexplained; comparative clinical results were not reported by intervention group
Ettinger et al. 2024. Journal of oral and maxillofacial surgery : official journal of the American Association of Oral and Maxillofacial Surgeons. PMID 38981580	clinical_comparative	robins_i	moderate; moderate; low; no_information; no_information; low; no_information	moderate	abstract only; residual confounding remains despite adjustment; exclusion for unavailable variables may select participants
Zhang et al. 2024. Zhonghua wai ke za zhi [Chinese journal of surgery]. PMID 39090062	clinical_comparative	robins_i	serious; moderate; low; no_information; low; moderate; no_information	serious	abstract only; serious confounding because reconstruction groups followed differing defects and no confounder adjustment is reported
Huang et al. 2025. The Journal of bone and joint surgery. American volume. PMID 39729975	clinical_comparative	robins_i	serious; low; low; no_information; low; moderate; no_information	serious	Serious confounding: lumbosacral fixation was introduced after observed failures and had shorter follow-up
Le et al. 2025. The Journal of craniofacial surgery. PMID 39819879	clinical_comparative	robins_i	serious; serious; low; moderate; serious; moderate; no_information	serious	D1 non-overlapping historical groups and group-specific procedures were unadjusted; D2 CT availability selected 9/36 PBP and 9/17 PSP patients; D5 large differential exclusions for unavailable CT data were not addressed
Mohamed et al. 2025. Journal of oral and maxillofacial surgery : official journal of the American Association of Oral and Maxillofacial Surgeons. PMID 40480278	clinical_comparative	robins_i	serious; moderate; low; no_information; no_information; moderate; no_information	serious	D1 Cox adjustment covered only age, sex and preoperative diagnosis despite non-random plate assignment
Chen et al. 2025. BMC ophthalmology. PMID 40826038	clinical_comparative	robins_i	serious; low; low; no_information; low; moderate; no_information	serious	D1 no confounding adjustment was performed and authors state material selection may reflect surgeon experience and patient needs
Ren et al. 2025. Head & face medicine. PMID 41039504	clinical_comparative	robins_i	moderate; no_information; low; no_information; low; moderate; no_information	moderate	D1 weighting balanced six measured covariates but cannot address unmeasured confounding; D6 assessor blinding was

Study	Tier	Tool	Judgements	Overall	Support
					unreported and Hertel measurement was described as operator-dependent
Zoabi et al. 2026. Journal of oral and maxillofacial surgery : official journal of the American Association of Oral and Maxillofacial Surgeons. PMID 41344382	clinical_comparative	robins_i	serious; moderate; low; no_information; no_information; moderate; no_information	serious	abstract only; planning technique depended on case specifics and designer preference without confounder adjustment
Lv et al. 2025. Journal of orthopaedic surgery and research. PMID 41361902	clinical_comparative	robins_i	serious; serious; low; no_information; no_information; moderate; no_information	serious	implant choice was non-random with groups of 3 versus 18, markedly different follow-up, and no confounder adjustment; eligibility required more than 24 months of follow-up
Adell-Gómez et al. 2026. Journal of cranio-maxillo-facial surgery : official publication of the European Association for Cranio-Maxillo-Facial Surgery. PMID 41506104	clinical_comparative	robins_i	serious; serious; low; no_information; no_information; moderate; no_information	serious	abstract only; prospective PSI patients were compared with retrospective controls without confounder adjustment; differential selection across cohorts was not addressed
Troise et al. 2026. Journal of stomatology, oral and maxillofacial surgery. PMID 41544967	clinical_comparative	robins_i	serious; no_information; low; no_information; no_information; moderate; no_information	serious	abstract only; the non-randomized case-control comparison reported no baseline balance or confounding adjustment
Yang et al. 2026. Cancers. PMID 41595190	clinical_comparative	robins_i	serious; moderate; low; moderate; serious; moderate; no_information	serious	Plate choice depended on graft complexity and no adjusted comparison was reported; 29 of 111 patients lacked data for accuracy analysis
Kim et al. 2026. Korean journal of neurotrauma. PMID 41657642	clinical_comparative	robins_i	serious; low; low; low; low; moderate; no_information	serious	Implant allocation depended on consent, groups differed in surgery timing and diagnosis, and no confounding adjustment was performed
Mäntynen et al. 2026. Maxillofacial plastic and reconstructive surgery. PMID 41779306	clinical_comparative	robins_i	serious; low; low; low; low; moderate; no_information	serious	Protocol allocation followed case-specific radiologist assessment, age predicted allocation, and outcome comparisons were unadjusted
Prikule et al. 2026. Journal of clinical medicine. PMID 42074623	clinical_comparative	robins_i	serious; serious; low; no_information; serious; serious; no_information	serious	Historical and prospective groups had no confounding adjustment; only 74 of 216 patients with complete records and adequate CT were analyzed; retrospective long-term follow-up was incomplete; clinical documentation diffe
Aguilar et al. 2026. Craniomaxillofacial trauma & reconstruction. PMID 42346039	clinical_comparative	robins_i	serious; moderate; low; low; no_information; moderate; no_information	serious	plate design and segmentation were observational exposures analyzed without confounder adjustment; subgroup denominators were inconsistent with the stated cohort total
Rouhiainen et al. 2026. JPRAS open. PMID 42565001	clinical_comparative	robins_i	serious; moderate; low; moderate; serious; moderate; no_information	serious	surgeon-preference allocation produced differences in advancement, ANB, ethnicity, surgeons, and protocols without adjustment; 13/72 patients were excluded for missing or mistimed imaging
De Oliveira Gorla et al. 2026.	clinical_com	robins_i	serious; no_information; low;	seri	abstract only; retrospective

Study	Tier	Tool	Judgements	Overall	Support
The British journal of oral & maxillofacial surgery. PMID 42608239	parative		no_information; moderate; moderate; no_information	ous	navigation versus freehand groups of five had no reported confounding control
Bertram et al. 2026. BMC Oral Health. PMID 42638112	clinical_comparative	robins_i	serious; serious; low; low; low; moderate; no_information	serious	D1: logistical allocation and no adjusted analysis left case complexity and timing confounding; D2: eligibility excluded intraoperative plan changes and positioning complications after intervention
Du et al. 2020. Engineering. doi:10.1016/j.eng.2020.02.019	clinical_series	jbi_case_series	unclear; unclear; unclear; unclear; unclear; no; no; no; unclear; unclear	0/10	abstract only; Q1 no inclusion criteria; Q2 no standard condition measure reported; Q3 no identification method reported; Q4 consecutive inclusion not stated; Q5 completeness not stated; Q6 no demographics; Q7 no clinica
Elsharabasy et al. 2026. Journal of Dentistry. doi:10.1016/j.jdent.2026.106453	clinical_series	jbi_case_series	unclear; unclear; yes; unclear; unclear; no; yes; no; unclear; no	2/10	abstract only; Q1 complex defects specified without explicit criteria; Q2 standard condition measurement not described; Q3 contrast-enhanced CT was required; Q4 consecutive inclusion not stated; Q5 completeness not state
Palmisciano et al. 2025. Journal of Neurological Surgery Part B Skull Base. doi:10.1055/s-0045-1803877	clinical_series	jbi_case_series	unclear; unclear; yes; unclear; unclear; no; no; no; unclear; no	1/10	abstract only; “complex” fractures were undefined; standard measurement was unstated; preoperative CT supported valid identification; consecutive and complete inclusion were unstated; demographics conflicted (7/10 report
Daum et al. 2025. FACE. doi:10.1177/27325016251352171	clinical_series	jbi_case_series	yes; unclear; unclear; unclear; yes; no; yes; yes; unclear; yes	5/10	abstract only; Q1 all adults undergoing the specified procedure during 2010–2024 were eligible; Q2 standard condition measurement not described; Q3 condition-identification methods not described; Q4 consecutive inclusion
D’Alpaos et al. 2023. . doi:10.20944/preprints202307.1681.v1	clinical_series	jbi_case_series	unclear; unclear; unclear; unclear; unclear; unclear; unclear; no; unclear; unclear	0/10	abstract only; no eligibility, diagnostic, consecutive or complete inclusion, demographic, clinical-site, outcome-result, or analysis details; 14 treated patients but only 12 implants described
Moncayo-Matute et al. 2025. . doi:10.20944/preprints202507.1208.v1	clinical_series	jbi_case_series	unclear; unclear; unclear; unclear; unclear; unclear; unclear; unclear; unclear; unclear	0/10	abstract only; Q1 inclusion criteria not reported; Q2 standard condition measurement not reported; Q3 identification method not reported; Q4 consecutive inclusion not reported; Q5 complete inclusion not reported; Q6 demo
Nuermaimaiti et al. 2026. . doi:10.21203/rs.3.rs-10388339/v1	clinical_series	jbi_case_series	yes; yes; yes; yes; no; yes; yes; yes; yes; yes	9/10	Q1: detailed eligibility criteria reported; Q2: tumors were histologically confirmed; Q3: core-needle biopsy or resection

Study	Tier	Tool	Judgements	Overall	Support
					histopathology identified tumors; Q4: 27 patients were consecutively screened; Q5: six patients we
Yj et al. 2021. . doi:10.21203/rs.3.rs-141473/v1	clinical_series	jbi_case_series	yes; yes; yes; unclear; unclear; yes; yes; yes; yes; yes	8/10	Malignant periacetabular tumors treated during a defined period with imaging, histology, and staging; demographics, clinical details, outcomes, Tianjin site, and descriptive analyses reported; consecutive and complete in
Abdeldayem et al. 2026. . doi:10.21203/rs.3.rs-9337194/v1	clinical_series	jbi_case_series	yes; unclear; unclear; unclear; unclear; yes; yes; yes; yes; yes	6/10	Q1: detailed eligibility criteria reported; Q2: no standardized lesion measurement described; Q3: diagnostic identification method unstated; Q4: consecutiveness unstated; Q5: complete inclusion not established; Q6: age,
Haneef et al. 2026. International Journal of Applied Dental Sciences. doi:10.22271/oral.2026.v12.i6a.2425	clinical_series	jbi_case_series	yes; yes; yes; unclear; unclear; no; no; yes; yes; no	5/10	Q1 explicit inclusion and exclusion criteria; Q2 standardized anthropometry and named rating systems; Q3 high-resolution CT documented defects; Q4 consecutive inclusion not stated; Q5 completeness not stated; Q6 no age o
Axrorov et al. 2026. Zenodo (CERN European Organization for Nuclear Research). doi:10.5281/zenodo.19562530	clinical_series	jbi_case_series	unclear; unclear; unclear; unclear; unclear; no; no; yes; unclear; unclear	1/10	abstract only; Q1 no inclusion criteria; Q2 standard condition measurement not described; Q3 CT was described for planning rather than condition identification; Q4 consecutive inclusion not stated; Q5 completeness not st
Gondra et al. 2026. . doi:10.64898/2026.04.02.26349771	clinical_series	jbi_case_series	yes; yes; yes; yes; yes; unclear; no; yes; yes; yes	8/10	Q1: cranial-defect eligibility stated; Q2: volumetric CT measured defects; Q3: CT segmentation identified defects; Q4: patients were consecutive; Q5: no patients were excluded after adoption; Q6: participant demographics
Gander et al. 2015. Journal of cranio-maxillo-facial surgery : official publication of the European Association for Cranio-Maxillo-Facial Surgery. PMID 25465486	clinical_series	jbi_case_series	unclear; unclear; unclear; unclear; unclear; unclear; unclear; yes; unclear; unclear	1/10	abstract only; Q1 inclusion criteria not reported; Q2 standard condition measurement not reported; Q3 identification method not reported; Q4 consecutive inclusion not reported; Q5 complete inclusion not reported; Q6 demo
Wilde et al. 2015. International journal of computer assisted radiology and surgery. PMID 25843949	clinical_series	jbi_case_series	unclear; unclear; unclear; unclear; unclear; unclear; unclear; yes; unclear; unclear	1/10	abstract only; Q1 no explicit inclusion criteria; Q2 no standard condition measure described; Q3 identification method not described; Q4 consecutive inclusion not stated; Q5 complete inclusion not stated; Q6 demographics
Schepers et al. 2015. Journal of cranio-maxillo-facial surgery : official publication of the European Association for Cranio-	clinical_series	jbi_case_series	unclear; unclear; unclear; yes; unclear; unclear; yes; yes; unclear; yes	4/10	abstract only; Q1 inclusion criteria not reported; Q2 standard condition measurement not reported; Q3 identification

Study	Tier	Tool	Judgements	Overall	Support
Maxillo-Facial Surgery. PMID 25911122					method not reported; Q4 seven consecutive patients stated; Q5 complete inclusion not established; Q6 de
Manrique et al. 2015. The Journal of craniofacial surgery. PMID 25974770	clinical_series	jbi_case_series	unclear; unclear; unclear; unclear; unclear; no; yes; yes; unclear; yes	3/10	abstract only; Q1: no explicit inclusion criteria; Q2: no standardized condition measure described; Q3: identification method unstated; Q4: consecutiveness unstated; Q5: completeness unstated; Q6: age alone incompletely
Sun et al. 2015. Hua xi kou qiang yi xue za zhi = Huaxi kouqiang yixue zazhi = West China journal of stomatology. PMID 26281256	clinical_series	jbi_case_series	unclear; yes; yes; unclear; unclear; unclear; unclear; yes; unclear; unclear	3/10	abstract only; Q1: no inclusion criteria; Q2: routine multiplanar CT assessment reported; Q3: CT and three-dimensional CT identified the condition; Q4: consecutiveness unstated; Q5: completeness unstated; Q6: demographic
Zhang et al. 2015. The Journal of craniofacial surgery. PMID 26594999	clinical_series	jbi_case_series	unclear; yes; yes; unclear; unclear; unclear; unclear; yes; unclear; unclear	3/10	abstract only; Q1 eligibility criteria were not stated; Q2 defects were measured using CT; Q3 CT is a valid defect-identification method; Q4 consecutive inclusion was not stated; Q5 complete inclusion was not stated; Q6
Baumann et al. 2015. Journal of oral and maxillofacial surgery : official journal of the American Association of Oral and Maxillofacial Surgeons. PMID 26608137	clinical_series	jbi_case_series	yes; unclear; unclear; unclear; unclear; unclear; yes; yes; unclear; unclear	3/10	abstract only; Q1: orbital-defect eligibility criteria stated; Q2: no standardized condition measure described; Q3: identification method unstated; Q4: consecutiveness unstated; Q5: completeness unstated; Q6: demographic
Suojanen et al. 2016. Journal of cranio-maxillo-facial surgery : official publication of the European Association for Cranio-Maxillo-Facial Surgery. PMID 27769722	clinical_series	jbi_case_series	unclear; unclear; unclear; unclear; unclear; unclear; yes; unclear; unclear; unclear	1/10	abstract only; Q1 inclusion criteria not reported; Q2 standard condition measurement not reported; Q3 identification method not reported; Q4 consecutive inclusion not reported; Q5 complete inclusion not reported; Q6 demo
Oh et al. 2016. The Journal of craniofacial surgery. PMID 28005746	clinical_series	jbi_case_series	unclear; yes; yes; yes; yes; no; unclear; yes; no; yes	6/10	abstract only; inclusion criteria were not specified; demographics, detailed clinical information and site were not reported
Moser et al. 2017. Journal of cranio-maxillo-facial surgery : official publication of the European Association for Cranio-Maxillo-Facial Surgery. PMID 28011181	clinical_series	jbi_case_series	unclear; unclear; unclear; unclear; unclear; unclear; yes; yes; unclear; unclear	2/10	abstract only; Q1 explicit eligibility criteria were not stated; Q2 a standard defect-measurement method was not specified; Q3 defect identification method was not specified; Q4 consecutive inclusion was not stated; Q5 c
Luongo et al. 2016. BioMed research international. PMID 28070512	clinical_series	jbi_case_series	yes; yes; yes; unclear; unclear; yes; yes; yes; yes; yes	8/10	consecutive and complete inclusion across the three centres were not reported
Mommaerts et al. 2016. Annals of maxillofacial surgery. PMID 28299273	clinical_series	jbi_case_series	unclear; no; no; unclear; unclear; no; yes; yes; yes; yes	4/10	inclusion and consecutive or complete enrolment were not reported; condition assessment

Study	Tier	Tool	Judgements	Overall	Support
					used unvalidated aesthetic assumptions; full participant demographics were not reported
Fu et al. 2017. Journal of oral and maxillofacial surgery : official journal of the American Association of Oral and Maxillofacial Surgeons. PMID 28463685	clinical_series	jbi_case_series	unclear; yes; yes; unclear; unclear; no; unclear; yes; no; na	3/10	abstract only; inclusion, consecutive and complete enrolment were not reported; demographics, detailed clinical information and site were not reported
Rana et al. 2017. Journal of cranio-maxillo-facial surgery : official publication of the European Association for Cranio-Maxillo-Facial Surgery. PMID 28552201	clinical_series	jbi_case_series	unclear; unclear; unclear; unclear; unclear; no; unclear; yes; no; yes	2/10	abstract only; inclusion, ascertainment, consecutive and complete enrolment were not reported; demographics, detailed clinical information and participating sites were not reported
Li et al. 2017. International journal of oral and maxillofacial surgery. PMID 28552440	clinical_series	jbi_case_series	yes; unclear; unclear; no; no; yes; yes; yes; no; yes	5/10	explicit inclusion and exclusion criteria reported; age and sex reported; skeletal classes and asymmetry reported; CT accuracy outcomes and clinical follow-up reported; descriptive analysis was justified by the sample of
Byrne et al. 2017. The Journal of hand surgery. PMID 28709790	clinical_series	jbi_case_series	unclear; yes; yes; yes; yes; no; yes; yes; no; unclear	6/10	abstract only; inclusion criteria were not specified; participant demographics and site were not reported; statistical methods were not described
Heufelder et al. 2017. Journal of cranio-maxillo-facial surgery : official publication of the European Association for Cranio-Maxillo-Facial Surgery. PMID 28793965	clinical_series	jbi_case_series	unclear; unclear; unclear; yes; yes; no; unclear; yes; no; yes	4/10	abstract only; inclusion and condition-identification methods were not reported; demographics, detailed clinical information and site were not reported
Wang et al. 2018. Aesthetic plastic surgery. PMID 29260271	clinical_series	jbi_case_series	unclear; unclear; unclear; unclear; unclear; no; unclear; yes; no; yes	2/10	abstract only; inclusion, ascertainment, consecutive and complete enrolment were not reported; demographics, detailed clinical information and site were not reported
Yang et al. 2018. Oral oncology. PMID 29496055	clinical_series	jbi_case_series	unclear; unclear; unclear; unclear; unclear; unclear; unclear; yes; unclear; yes	2/10	abstract only; six-month adverse events, follow-up, and reconstruction accuracy were clearly summarized with appropriate descriptive statistics; other checklist information was not reported
Day et al. 2018. Plastic and reconstructive surgery. Global open. PMID 29707444	clinical_series	jbi_case_series	yes; unclear; unclear; unclear; yes; yes; yes; yes; yes; yes	7/10	all patients receiving the technologies at the tertiary referral centre were included, but consecutive enrolment and standard condition ascertainment were unclear; group demographics, diagnoses, clinical details, complic
Alike et al. 2016. Zhongguo xiu fu chong jian wai ke za zhi = Zhongguo xiufu chongjian waike zazhi = Chinese journal of reparative and reconstructive surgery. PMID 29786331	clinical_series	jbi_case_series	unclear; unclear; unclear; unclear; unclear; yes; yes; yes; unclear; yes	4/10	abstract only; Q1: no inclusion criteria; Q2: no measurement standard stated; Q3: identification methods unstated; Q4: consecutiveness unstated; Q5: completeness unstated; Q6: age and sex reported; Q7: diagnoses, sites,

Study	Tier	Tool	Judgements	Overall	Support
Chana Rodríguez et al. 2018. Revista espanola de cirugia ortopedica y traumatologia (English ed.). PMID 29807784	clinical_series	jbi_case_series	unclear; unclear; unclear; unclear; unclear; no; no; yes; no; yes	2/10	abstract only; eligibility, condition ascertainment, consecutive enrolment and complete inclusion unclear; demographics and clinical details absent; operative and CT outcomes clearly reported; site details absent; descri
Dimitroulis et al. 2018. Journal of cranio-maxillo-facial surgery : official publication of the European Association for Cranio-Maxillo-Facial Surgery. PMID 29859818	clinical_series	jbi_case_series	yes; unclear; unclear; yes; yes; yes; yes; yes; no; unclear	6/10	abstract only; adults with Category 5 disease during a defined period were eligible and consecutively included; ascertainment methods unclear; sex, age and clinical information reported; outcomes and 12–24-month follow-u
Morales-Gómez et al. 2018. Journal of neurosurgery. PMID 29905512	clinical_series	jbi_case_series	yes; yes; yes; yes; unclear; unclear; yes; yes; unclear; yes	7/10	abstract only; Q1 CT-based cranioplasty over one year defined inclusion; Q2 defects were measured using CT-based modelling; Q3 CT is a valid defect-identification method; Q4 the 22 patients were explicitly consecutive; Q
Kärkkäinen et al. 2018. The British journal of oral & maxillofacial surgery. PMID 30220611	clinical_series	jbi_case_series	yes; unclear; unclear; unclear; unclear; no; no; yes; no; yes	3/10	abstract only; isolated orbital fractures treated under the Helsinki protocol defined eligibility; ascertainment, consecutive enrolment and complete inclusion unclear; demographics and clinical details absent; outcomes a
Vlok et al. 2018. South African journal of surgery. Suid-Afrikaanse tydskrif vir chirurgie. PMID 30264941	clinical_series	jbi_case_series	yes; yes; yes; unclear; unclear; unclear; unclear; yes; unclear; yes	5/10	abstract only; Q1 patients requiring custom-implant cranioplasty were specified; Q2 cranial defects were reconstructed by three-dimensional CT; Q3 CT is a valid defect-identification method; Q4 only the prospective subgr
Weber et al. 2019. International orthopaedics. PMID 30306217	clinical_series	jbi_case_series	yes; unclear; unclear; unclear; unclear; no; no; yes; no; yes	3/10	abstract only; Paprosky III revision cases defined eligibility; standard ascertainment, consecutive enrolment and complete inclusion unclear; demographics and clinical details absent; CT-based outcomes clearly reported;
Ridwan-Pramana et al. 2019. The Journal of craniofacial surgery. PMID 30676441	clinical_series	jbi_case_series	unclear; unclear; unclear; unclear; unclear; unclear; unclear; yes; unclear; yes	2/10	Q1 eligibility criteria not stated; Q2 standard defect measurement not described; Q3 defect identification method not described; Q4 consecutive inclusion not stated; Q5 complete inclusion not stated; Q6 participant demog
Đurić et al. 2019. British journal of neurosurgery. PMID 30732480	clinical_series	jbi_case_series	unclear; unclear; unclear; unclear; unclear; unclear; unclear; yes; unclear; yes	2/10	abstract only; Q1 eligibility criteria not stated; Q2 standard defect measurement not described; Q3 defect identification method not described; Q4 consecutive inclusion not stated; Q5 complete

Study	Tier	Tool	Judgements	Overall	Support
					inclusion not stated; Q6 pa
Maricevich et al. 2019. Surgical neurology international. PMID 30775055	clinical_series	jbi_case_series	yes; yes; yes; yes; yes; yes; yes; yes; yes; no	9/10	Q10 chi-square proportion testing did not account for the paired preoperative and postoperative symptom observations
Low et al. 2019. The Journal of craniofacial surgery. PMID 31261343	clinical_series	jbi_case_series	yes; unclear; unclear; unclear; unclear; no; no; yes; no; unclear	2/10	abstract only; partial bone-flap defect defined eligibility; no standard condition measure or identification method reported; consecutive and complete inclusion unclear; demographics and clinical details absent; outcomes
Li et al. 2020. International journal of oral and maxillofacial surgery. PMID 31353173	clinical_series	jbi_case_series	unclear; unclear; unclear; unclear; unclear; unclear; unclear; yes; unclear; unclear	1/10	abstract only; Q1–Q7: eligibility, measurement validity, recruitment, demographics, and clinical information were insufficiently reported; Q9: site demographics were absent; Q10: statistical methods were not described
Chen et al. 2020. Ophthalmic plastic and reconstructive surgery. PMID 31789791	clinical_series	jbi_case_series	unclear; yes; yes; unclear; unclear; yes; yes; yes; unclear; unclear	5/10	abstract only; Q1: inclusion criteria were not stated; Q4–Q5: consecutive and complete inclusion were not reported; Q9: site demographics were absent; Q10: statistical methods were not described
Seier et al. 2020. International journal of oral and maxillofacial surgery. PMID 31870519	clinical_series	jbi_case_series	unclear; unclear; unclear; unclear; unclear; unclear; unclear; yes; unclear; yes	2/10	abstract only; Q1–Q7: eligibility, ascertainment, recruitment, demographics, and detailed clinical information were not reported; Q9: site demographics were absent
Mangano et al. 2020. 3D printing in medicine. PMID 31915946	clinical_series	jbi_case_series	yes; yes; yes; yes; yes; yes; yes; yes; unclear; yes	9/10	Q1 explicit eligibility criteria reported; Q2 atrophy assessed using standardized radiography and CBCT; Q3 CBCT identified residual-bone dimensions; Q4 all eligible patients during the stated period were considered; Q5 a
El Morsy et al. 2020. Clinical implant dentistry and related research. PMID 32103625	clinical_series	jbi_case_series	unclear; yes; unclear; unclear; unclear; unclear; unclear; yes; unclear; unclear	2/10	abstract only; Q1: inclusion criteria were not stated; Q3–Q7: identification, recruitment, demographics, and clinical details were insufficiently reported; Q9: site demographics were absent; Q10: statistical methods were
Merema et al. 2020. Oral diseases. PMID 32176821	clinical_series	jbi_case_series	yes; yes; yes; unclear; unclear; yes; yes; yes; unclear; yes	7/10	Q4–Q5: consecutive and complete inclusion were not stated; Q9: presenting-site demographics were not reported
Durand-Hill et al. 2020. Journal of orthopaedic research : official publication of the Orthopaedic Research Society. PMID 32427351	clinical_series	jbi_case_series	unclear; yes; unclear; unclear; unclear; yes; yes; yes; unclear; yes	5/10	abstract only; Q1: inclusion criteria were not stated; Q3: defect-identification criteria were not described; Q4–Q5: consecutive and complete inclusion were not reported; Q9: site demographics were not reported
Rückschloß et al. 2020. Journal of cranio-maxillo-facial surgery : official publication of the	clinical_series	jbi_case_series	unclear; unclear; unclear; unclear; unclear; unclear; yes; yes;	2/10	abstract only; Q1 precise inclusion criteria not reported; Q2 standard condition measurement

Study	Tier	Tool	Judgements	Overall	Support
European Association for Cranio-Maxillo-Facial Surgery. PMID 32505528			unclear; unclear		not reported; Q3 identification method not reported; Q4 consecutive inclusion not reported; Q5 complete inclusion not reported;
Boccalatte et al. 2020. Oral and maxillofacial surgery. PMID 32518971	clinical_series	jbi_case_series	unclear; unclear; unclear; unclear; unclear; unclear; unclear; yes; unclear; yes	2/10	abstract only; Q1 inclusion criteria not reported; Q2 standard condition measurement not reported; Q3 identification method not reported; Q4 consecutive inclusion not reported; Q5 complete inclusion not reported; Q6 demo
Wong et al. 2021. International journal of oral and maxillofacial surgery. PMID 32919821	clinical_series	jbi_case_series	yes; unclear; unclear; yes; yes; yes; unclear; yes; unclear; yes	6/10	abstract only; 30 consecutive adults requiring Le Fort I surgery were included, sex and age reported, quantitative accuracy outcomes provided, and descriptive analysis appropriate; diagnostic methods, detailed clinical i
Blumer et al. 2021. Journal of oral and maxillofacial surgery : official journal of the American Association of Oral and Maxillofacial Surgeons. PMID 33010215	clinical_series	jbi_case_series	unclear; unclear; unclear; unclear; unclear; unclear; yes; yes; unclear; unclear	2/10	abstract only; Q1–Q6: eligibility, ascertainment, consecutive or complete inclusion, and demographics were not reported; Q9: site demographics were not reported; Q10: analysis methods were not described
Burastero et al. 2020. Archives of orthopaedic and trauma surgery. PMID 33170352	clinical_series	jbi_case_series	unclear; unclear; unclear; unclear; unclear; unclear; yes; yes; unclear; unclear	2/10	abstract only; Q1 inclusion criteria not reported; Q2 standard condition measurement not reported; Q3 identification method not reported; Q4 consecutive inclusion not reported; Q5 complete inclusion not reported; Q6 demo
Freigang et al. 2020. BMC musculoskeletal disorders. PMID 33234122	clinical_series	jbi_case_series	yes; yes; yes; yes; no; yes; yes; yes; unclear; yes	8/10	Q5: two patients were lost to follow-up; Q9: presenting-site demographics were not clearly reported
Mian et al. 2021. Oral and maxillofacial surgery. PMID 33237435	clinical_series	jbi_case_series	unclear; unclear; unclear; unclear; unclear; unclear; unclear; yes; unclear; yes	2/10	abstract only; Q1: eligibility criteria not reported; Q2: standard measurement of end-stage disease not reported; Q3: diagnostic methods not reported; Q4: consecutive inclusion not stated; Q5: complete inclusion not stat
Peng et al. 2020. BioMed research international. PMID 33335928	clinical_series	jbi_case_series	yes; yes; yes; unclear; unclear; yes; yes; yes; yes; unclear	7/10	Q1: explicit inclusion and exclusion criteria; Q2: tumors assessed with radiography, CT, MRI, bone scan, and biopsy; Q3: pathology and imaging validly identified diagnoses; Q4: consecutive inclusion not stated; Q5: eligi
Wu et al. 2021. World neurosurgery. PMID 33359737	clinical_series	jbi_case_series	unclear; unclear; unclear; unclear; unclear; unclear; unclear; yes; unclear; unclear	1/10	abstract only; Q1: eligibility criteria not reported; Q2: standard condition measurement not reported; Q3: tumor-identification methods not reported; Q4: consecutive inclusion not stated; Q5: complete inclusion not state
Dvoracek et al. 2021. Plastic and	clinical_series	jbi_case	unclear; yes; yes; unclear;	3/1	abstract only; Q1: eligibility

Study	Tier	Tool	Judgements	Overall	Support
reconstructive surgery. PMID 33370061	s	_series	unclear; unclear; unclear; yes; unclear; unclear	0	criteria not reported; Q2: orbital fractures and volumes assessed by CT; Q3: CT was a valid identification method; Q4: consecutive inclusion not stated; Q5: complete inclusion not stated; Q
Schön et al. 2021. World neurosurgery. PMID 33418118	clinical_series	jbi_case_series	yes; unclear; unclear; yes; yes; unclear; yes; yes; unclear; yes	6/10	abstract only; Q2–Q3 standard condition measurement and identification methods were not reported; Q6 demographics were not reported; Q9 the clinical site was not reported
Zavattero et al. 2021. The Laryngoscope. PMID 33452834	clinical_series	jbi_case_series	unclear; unclear; unclear; unclear; no; unclear; unclear; yes; unclear; unclear	1/10	abstract only; Q1: eligibility criteria not reported; Q2: standard condition measurement not reported; Q3: diagnostic methods not reported; Q4: consecutive inclusion not stated; Q5: only 47 of 65 included patients were a
Maydanshahi et al. 2021. Biomedical materials (Bristol, England). PMID 33524959	clinical_series	jbi_case_series	unclear; unclear; unclear; unclear; unclear; unclear; unclear; yes; unclear; unclear	1/10	abstract only; Q1 inclusion criteria not reported; Q2 standard condition measurement not reported; Q3 identification method not reported; Q4 consecutive inclusion not reported; Q5 complete inclusion not reported; Q6 demo
Da Silva Júnior et al. 2021. 3D printing in medicine. PMID 33548008	clinical_series	jbi_case_series	yes; yes; yes; yes; yes; yes; yes; yes; yes; yes	10/10	Sixteen consecutive patients had CT-defined defects, complete demographic and clinical reporting, clear follow-up outcomes, an identified site, and appropriate descriptive analysis
Kozakiewicz et al. 2021. Materials (Basel, Switzerland). PMID 33578685	clinical_series	jbi_case_series	yes; yes; yes; yes; yes; unclear; yes; yes; unclear; yes	8/10	Q1: delayed post-traumatic cases were defined; Q2: CT, facial scanning, and the ODI scale were used; Q3: high-resolution CT and clinical history were valid; Q4: consecutive inclusion explicitly stated; Q5: the consecutiv
Basu et al. 2021. Journal of clinical neuroscience : official journal of the Neurosurgical Society of Australasia. PMID 33581784	clinical_series	jbi_case_series	unclear; unclear; unclear; unclear; unclear; unclear; unclear; yes; unclear; yes	2/10	abstract only; Q1–Q7 eligibility, condition identification, consecutive or complete inclusion, full demographics, and clinical details were not reported; Q9 the single site was unnamed
IJpma et al. 2021. JAMA network open. PMID 33599774	clinical_series	jbi_case_series	yes; yes; yes; yes; yes; yes; yes; yes; yes; yes	10/10	Q1: explicit fracture eligibility criteria; Q2: fractures measured using standardized thin-slice CT; Q3: Letournel-Judet classification and CT were valid; Q4: all consecutive patients included; Q5: all eligible consecuti
Brouwer de Koning et al. 2021. Scientific reports. PMID 33633247	clinical_series	jbi_case_series	yes; yes; yes; unclear; unclear; yes; yes; yes; yes; yes	8/10	Q1: planned segmental or hemimandibulectomy defined eligibility; Q2: conditions were assessed through CT and pathology; Q3: diagnostic and pathology methods were valid; Q4: consecutive inclusion not stated; Q5: eligible-
Mu et al. 2021. International	clinical_serie	jbi_case	yes; yes; yes; unclear; unclear;	7/1	Q1: explicit inclusion and

Study	Tier	Tool	Judgements	Overall	Support
orthopaedics. PMID 33666766	s	_series	yes; yes; yes; unclear; yes	0	exclusion criteria; Q2: symptoms and radiography, CT, or MRI used; Q3: imaging methods validly identified talar necrosis and collapse; Q4: consecutive inclusion not stated; Q5: complete inclusi
Agarwal et al. 2022. European journal of trauma and emergency surgery : official publication of the European Trauma Society. PMID 33683380	clinical_series	jbi_case_series	unclear; unclear; unclear; unclear; unclear; unclear; unclear; yes; unclear; unclear	1/10	abstract only; Q1: eligibility criteria not reported; Q2: standard condition measurement not reported; Q3: diagnostic methods not reported; Q4: consecutive inclusion not stated; Q5: complete inclusion not stated; Q6: dem
Kormi et al. 2020. Journal of Craniofacial Surgery. PMID 33705044	clinical_series	jbi_case_series	unclear; unclear; unclear; unclear; unclear; no; no; yes; unclear; yes	2/10	abstract only; Q1 one-year cohort but no criteria; Q2 standard condition measurement not described; Q3 fracture-identification method not described; Q4 consecutive inclusion not stated; Q5 completeness not stated; Q6 no
Zhang et al. 2021. Bioactive materials. PMID 33898870	clinical_series	jbi_case_series	yes; yes; yes; unclear; unclear; unclear; yes; yes; unclear; yes	6/10	Q1: explicit defect and follow-up criteria; Q2: defect size and outcomes measured radiographically; Q3: CT and radiography identified defects; Q4: consecutive enrolment not stated; Q5: eligible-case completeness not stat
Kobayashi et al. 2021. Neurologia medico-chirurgica. PMID 34092749	clinical_series	jbi_case_series	yes; yes; yes; yes; yes; yes; yes; yes; yes; yes	10/10	explicit criteria and CT-based identification were reported; all 18 consecutive patients were included; demographics, clinical information, outcomes, Japanese institute and appropriate descriptive analysis were reported
Yang et al. 2021. Zhonghua wai ke za zhi [Chinese journal of surgery]. PMID 34102730	clinical_series	jbi_case_series	unclear; unclear; unclear; unclear; unclear; yes; yes; yes; yes; no	4/10	abstract only; eligibility, diagnostic ascertainment and consecutive or complete inclusion were not reported; demographics, clinical information, outcomes and center were reported; analysis was questionable for eight pat
Shah et al. 2021. The Journal of craniofacial surgery. PMID 34224462	clinical_series	jbi_case_series	unclear; unclear; unclear; unclear; unclear; unclear; unclear; yes; unclear; unclear	1/10	abstract only; eligibility, diagnostic ascertainment, recruitment, demographics, clinical information, site and analysis were not reported; quantitative reconstruction outcomes were reported
Järvinen et al. 2021. The Journal of craniofacial surgery. PMID 34260465	clinical_series	jbi_case_series	unclear; unclear; unclear; unclear; unclear; unclear; yes; yes; unclear; unclear	2/10	abstract only; eligibility, diagnostic ascertainment, consecutive or complete inclusion, demographics, site and analysis were not reported; interventions and outcomes were described
Li et al. 2021. Zhonghua kou qiang yi xue za zhi = Zhonghua kouqiang yixue zazhi = Chinese journal of stomatology. PMID	clinical_series	jbi_case_series	unclear; unclear; unclear; unclear; unclear; unclear; unclear; yes; yes; unclear	2/10	abstract only; eligibility, diagnostic methods, recruitment completeness, demographics and analysis were not reported;

Study	Tier	Tool	Judgements	Overall	Support
34275217					outcomes and hospital site were reported
Atef et al. 2022. Oral and maxillofacial surgery. PMID 34328575	clinical_series	jbi_case_series	unclear; yes; unclear; unclear; unclear; unclear; yes; yes; unclear; unclear	3/10	abstract only; Q1 explicit inclusion criteria absent; Q2 standardized CT superimposition measured changes; Q3 deformity-identification method absent; Q4 consecutive inclusion not stated; Q5 complete inclusion not stated;
Wang et al. 2021. World journal of surgical oncology. PMID 34365976	clinical_series	jbi_case_series	yes; yes; yes; unclear; yes; yes; yes; yes; unclear; yes	8/10	indications and imaging or biopsy methods reported; consecutive inclusion not stated; all 15 patients completed follow-up; demographics, clinical information and outcomes reported; presenting site details unclear
Chepurnyi et al. 2021. Annals of maxillofacial surgery. PMID 34522661	clinical_series	jbi_case_series	yes; yes; yes; unclear; yes; yes; yes; yes; yes; no	8/10	clear criteria and CT-based assessment; consecutive recruitment not stated; all 11 eligible cases included; demographics, clinical data, outcomes and Kyiv site reported; analyses did not account for paired orbital measur
Antúnez-Conde et al. 2021. Frontiers in oncology. PMID 34676161	clinical_series	jbi_case_series	yes; unclear; unclear; unclear; unclear; yes; yes; yes; yes; no	5/10	explicit inclusion criteria; diagnostic ascertainment and consecutive or complete inclusion not reported; demographics, clinical data, outcomes and Madrid site reported; small-group tests ignored implant clustering
Yoon et al. 2021. Korean journal of neurotrauma. PMID 34760819	clinical_series	jbi_case_series	yes; yes; yes; unclear; unclear; yes; yes; yes; yes; unclear	7/10	Q4 consecutive inclusion was not reported; Q5 inclusion of all eligible patients was not established; Q10 statistical tests underlying reported comparisons were not identified
Badiali et al. 2021. Journal of personalized medicine. PMID 34834588	clinical_series	jbi_case_series	unclear; yes; yes; unclear; unclear; yes; yes; yes; yes; yes	7/10	Inclusion criteria were not explicit; consecutive inclusion was not stated; complete inclusion was not stated.
Pöppe et al. 2022. Acta neurochirurgica. PMID 34873659	clinical_series	jbi_case_series	yes; yes; yes; yes; yes; yes; yes; yes; yes; yes	10/10	Fourteen consecutive patients with CT-defined defects were completely included, with demographics, clinical information, outcomes, site, and descriptive analyses reported
Alqussair et al. 2022. The Journal of craniofacial surgery. PMID 34907951	clinical_series	jbi_case_series	yes; unclear; unclear; unclear; unclear; unclear; yes; yes; unclear; yes	4/10	abstract only; Q1 diagnosis, procedure, imaging availability, site and dates defined eligibility; Q2 reliability of the condition measure not reported; Q3 diagnostic method for skeletal Class II malocclusion absent; Q4 c
Arafat et al. 2022. The Journal of craniofacial surgery. PMID 35119415	clinical_series	jbi_case_series	unclear; unclear; unclear; unclear; unclear; unclear; yes; yes; unclear; unclear	2/10	abstract only; inclusion criteria and condition-assessment methods were not reported; consecutive and complete inclusion were not stated; demographics and clinical site were not reported; statistical

Study	Tier	Tool	Judgements	Overall	Support
					analysis was not des
Lv et al. 2022. Journal of plastic, reconstructive & aesthetic surgery : JPRAS. PMID 35183467	clinical_series	jbi_case_series	unclear; unclear; unclear; unclear; unclear; unclear; unclear; yes; unclear; unclear	1/10	abstract only; inclusion criteria were not reported; condition measurement and identification methods were not described; consecutive and complete inclusion were not stated; demographics and clinical information were not
Kittichokechai et al. 2022. Plastic and reconstructive surgery. Global open. PMID 35186634	clinical_series	jbi_case_series	yes; yes; yes; unclear; yes; yes; yes; yes; yes; yes	9/10	Consecutive inclusion was not stated.
Jeong et al. 2022. Polymers. PMID 35215652	clinical_series	jbi_case_series	yes; yes; yes; unclear; unclear; yes; yes; yes; unclear; yes	7/10	Q1 explicit inclusion and exclusion criteria reported; Q2 defects and outcomes assessed with standardized CT methods; Q3 etiologies and Brown defect categories identified the condition; Q4 consecutive inclusion not state
Dowgierd et al. 2022. Oral oncology. PMID 35255279	clinical_series	jbi_case_series	unclear; unclear; unclear; unclear; unclear; yes; yes; yes; unclear; yes	4/10	abstract only; inclusion criteria were not explicit; standard condition measurement was not described; diagnostic identification methods were not described; consecutive inclusion was not stated; complete inclusion was no
Park et al. 2022. The Journal of craniofacial surgery. PMID 35261367	clinical_series	jbi_case_series	yes; yes; yes; yes; unclear; unclear; yes; yes; unclear; yes	7/10	Complete inclusion was not stated; demographic values were incompletely reported; site or clinic demographics were not reported.
Lizio et al. 2022. Clinical oral implants research. PMID 35305283	clinical_series	jbi_case_series	yes; yes; yes; unclear; unclear; yes; yes; yes; yes; yes	8/10	Consecutive inclusion was not stated; complete inclusion was not stated.
Moellmann et al. 2022. Journal of clinical medicine. PMID 35407653	clinical_series	jbi_case_series	yes; yes; yes; unclear; yes; yes; yes; yes; yes; no	8/10	Q4: consecutive inclusion was not stated; Q10: numerous unadjusted correlation tests included inconsistent P-value reporting
Ali et al. 2022. Maxillofacial plastic and reconstructive surgery. PMID 35524015	clinical_series	jbi_case_series	yes; unclear; unclear; yes; unclear; yes; yes; yes; yes; yes	7/10	Q2: the need for full temporalis-flap reconstruction was not described as standardized; Q3: methods identifying the underlying lesions were not reported; Q5: complete inclusion was not established
Liu et al. 2022. Frontiers in bioengineering and biotechnology. PMID 35721863	clinical_series	jbi_case_series	yes; yes; unclear; unclear; unclear; yes; yes; yes; yes; no	6/10	Q3: diagnostic criteria for osteomyelitis and nonunion were not described; Q4: consecutive inclusion was not stated; Q5: complete inclusion was not established; Q10: chi-square comparisons used groups of only five or six
Ashraf et al. 2022. Surgical neurology international. PMID 35855120	clinical_series	jbi_case_series	yes; yes; yes; unclear; unclear; yes; yes; yes; unclear; yes	7/10	Q4 consecutive inclusion was not reported; Q5 inclusion of all eligible patients was not established; Q9 the clinical site was not identified
Mekcha et al. 2023. Journal of	clinical_series	jbi_case	unclear; yes; yes; unclear;	4/1	abstract only; Q1 explicit

Study	Tier	Tool	Judgements	Overall	Support
prosthodontic research. PMID 35858803	s	_series	unclear; unclear; unclear; yes; unclear; yes	0	inclusion criteria absent; Q2 ridge dimensions measured using CBCT-derived DICOM data; Q3 CBCT validly identified ridge deficiencies; Q4 consecutive inclusion not stated; Q5 complete inclusion
Woo et al. 2022. The Journal of craniofacial surgery. PMID 36041093	clinical_series	jbi_case_series	yes; unclear; unclear; unclear; unclear; unclear; no; yes; unclear; unclear	2/10	abstract only; Q2: no standard condition measurement was described; Q3: no diagnostic identification method was described; Q4: consecutive inclusion was not stated; Q5: complete inclusion was not established; Q6: demogra
Hu et al. 2022. BMC musculoskeletal disorders. PMID 36076197	clinical_series	jbi_case_series	yes; yes; yes; yes; unclear; yes; yes; yes; yes; no	8/10	Q5: complete inclusion after the stated exclusions was not established; Q10: the statistical method for the reported clinical score comparison was not specified
Abel et al. 2022. Journal of oral and maxillofacial surgery : official journal of the American Association of Oral and Maxillofacial Surgeons. PMID 36116544	clinical_series	jbi_case_series	unclear; unclear; unclear; unclear; yes; yes; yes; yes; no; yes	5/10	abstract only; all cases in the stated single-surgeon period were assessed; age and sex reported; procedures and sequencing reported; planned-versus-achieved outcomes with confidence intervals reported; statistical tests
Demes et al. 2023. Journal of stomatology, oral and maxillofacial surgery. PMID 36184071	clinical_series	jbi_case_series	yes; unclear; unclear; yes; unclear; no; no; yes; unclear; yes	4/10	abstract only; Q2: no standard condition measurement was described; Q3: no diagnostic identification method was described; Q5: complete inclusion was not established; Q6: sex and other demographics were omitted; Q7: clin
Wandell et al. 2023. Journal of oral and maxillofacial surgery : official journal of the American Association of Oral and Maxillofacial Surgeons. PMID 36202213	clinical_series	jbi_case_series	yes; unclear; unclear; unclear; unclear; yes; no; yes; yes; yes	5/10	abstract only; Q2: no standard condition measurement was described; Q3: no diagnostic identification method was described; Q4: consecutive recruitment was not stated; Q5: complete inclusion was not established; Q7: clini
Wang et al. 2022. BMC surgery. PMID 36203147	clinical_series	jbi_case_series	yes; yes; yes; unclear; unclear; yes; yes; yes; yes; no	7/10	Q4: consecutive inclusion was not stated; Q5: complete inclusion was not established; Q10: a t-test compared groups of three and five without reported assumption checks
Rios et al. 2022. Journal of clinical medicine. PMID 36233363	clinical_series	jbi_case_series	yes; yes; yes; yes; yes; yes; yes; yes; yes; no	9/10	Fisher’s exact tests were used to compare average continuous discrepancies without a stated justification
He et al. 2023. Oral surgery, oral medicine, oral pathology and oral radiology. PMID 36241591	clinical_series	jbi_case_series	unclear; unclear; unclear; unclear; unclear; unclear; unclear; yes; yes; yes	3/10	abstract only; eligibility, diagnostic methods, consecutive and complete inclusion, demographics, and detailed clinical characteristics were not reported
Annino et al. 2022. Oral oncology. PMID 36384079	clinical_series	jbi_case_series	yes; unclear; unclear; unclear; unclear; yes; yes; yes; yes; yes	6/10	abstract only; standardized identification of

Study	Tier	Tool	Judgements	Overall	Support
					osteoradionecrosis was not described; consecutive inclusion was unstated; accuracy was evaluable in only 18 of 26 cases
Gamieldien et al. 2023. European journal of orthopaedic surgery & traumatology : orthopedie traumatologie. PMID 36385681	clinical_series	jbi_case_series	unclear; unclear; unclear; unclear; unclear; unclear; yes; yes; unclear; yes	3/10	abstract only; eligibility and defect-identification methods were not reported; consecutive or complete inclusion was unstated; sex and institutional details were absent
Leung et al. 2023. Clinical oral investigations. PMID 36401069	clinical_series	jbi_case_series	unclear; yes; unclear; unclear; unclear; unclear; unclear; yes; unclear; yes	3/10	abstract only; eligibility and condition-identification methods were absent; consecutive and complete inclusion were unstated; demographics, clinical characteristics, and site details were incomplete
Annino et al. 2022. Laryngoscope investigative otolaryngology. PMID 36544971	clinical_series	jbi_case_series	yes; yes; yes; yes; no; yes; yes; yes; yes; yes	9/10	ten of 70 cases were excluded, including seven without postoperative CT and two with major plan deviations
Merta et al. 2023. Journal of plastic, reconstructive & aesthetic surgery : JPRAS. PMID 36563638	clinical_series	jbi_case_series	unclear; yes; unclear; unclear; unclear; unclear; unclear; yes; unclear; yes	3/10	abstract only; eligibility and diagnostic methods were not reported; consecutive or complete inclusion was not stated; demographics, detailed clinical information, and setting were insufficiently reported
Shao et al. 2023. BMC musculoskeletal disorders. PMID 36698116	clinical_series	jbi_case_series	yes; yes; yes; unclear; unclear; yes; yes; yes; yes; yes	8/10	consecutive enrolment was not stated; completeness of inclusion was not demonstrated
Patel et al. 2023. Journal of maxillofacial and oral surgery. PMID 36776348	clinical_series	jbi_case_series	yes; yes; yes; unclear; unclear; unclear; yes; yes; unclear; yes	6/10	Q1 prior partial or total maxillectomy and at least six months disease-free defined; Q2–Q3 clinical and radiographic disease-free assessment used; Q7 implant planning and placement described; Q8 scheduled follow-up and c
Bevini et al. 2023. Journal of clinical medicine. PMID 36902725	clinical_series	jbi_case_series	yes; unclear; unclear; unclear; no; yes; yes; yes; yes; yes	6/10	Q1 prospective eligibility by disease and required mandibular resection defined; Q5 one of 11 enrolled patients was excluded after follow-up dropout; Q6 age and sex reported; Q7 disease and reconstruction workflow descri
Chowdhury et al. 2023. Journal of maxillofacial and oral surgery. PMID 37041951	clinical_series	jbi_case_series	unclear; unclear; unclear; unclear; unclear; unclear; unclear; unclear; yes; unclear	1/10	abstract only; Q9 AFDC, New Delhi was identified as the clinical site
Hanusrichter et al. 2023. International orthopaedics. PMID 37145143	clinical_series	jbi_case_series	unclear; unclear; unclear; unclear; unclear; unclear; unclear; yes; unclear; unclear	1/10	abstract only; Q8 follow-up, reoperations, loosening and patient-reported outcomes clearly reported
Lv et al. 2023. Frontiers in bioengineering and biotechnology. PMID 37214294	clinical_series	jbi_case_series	yes; yes; yes; yes; yes; yes; yes; no; yes; no	8/10	Q1 primary malignant tumours undergoing total sacrectomy with implant defined; Q2–Q3 biopsy established pathology; Q4–Q5 12 consecutive patients reviewed; Q6–Q7 age, sex, diagnoses and operative details reported; Q8 cont
Abdelazeem et al. 2023. World	clinical_series	jbi_case	yes; unclear; unclear; unclear;	4/1	abstract only; Q1 unilateral

Study	Tier	Tool	Judgements	Overall	Support
neurosurgery. PMID 37302708	s	_series	unclear; unclear; yes; yes; unclear; yes	0	frontal 3D-planned cranioplasty cases from 2017–2019 defined; Q7 reconstruction timing and technique described; Q8 complications and cosmetic and radiological symmetry reported; Q10 counts an
Csámer et al. 2023. Scientific reports. PMID 37491550	clinical_series	jbi_case_series	unclear; yes; yes; unclear; unclear; yes; yes; yes; yes; yes	7/10	Q1 inclusion criteria were not stated; Q4 consecutive inclusion was not reported; Q5 complete inclusion of eligible patients was not established
Ebker et al. 2023. The British journal of oral & maxillofacial surgery. PMID 37517940	clinical_series	jbi_case_series	unclear; unclear; unclear; unclear; unclear; unclear; unclear; yes; unclear; yes	2/10	abstract only; Q8 IMCU transfer, hospital stay and major complications reported; Q10 proportions and average stay summarize the series
Jirangkul et al. 2024. Archives of orthopaedic and trauma surgery. PMID 37658855	clinical_series	jbi_case_series	yes; unclear; unclear; unclear; unclear; unclear; yes; yes; unclear; yes	4/10	abstract only; Q1 acute displaced fractures and explicit exclusions; Q7 patient-specific modelling and MIPO described; Q8 union, scores, removals and complications reported; Q10 means and ranges summarize the series
Zhao et al. 2023. BMC oral health. PMID 37730602	clinical_series	jbi_case_series	unclear; unclear; unclear; unclear; unclear; yes; no; yes; no; yes	3/10	Q1-Q5 unclear: eligibility, diagnostic methods, and consecutive or complete inclusion were not specified; Q7 no: participant clinical characteristics were not clearly reported; Q9 no: site demographics were absent
Liu et al. 2023. Frontiers in bioengineering and biotechnology. PMID 37790252	clinical_series	jbi_case_series	yes; yes; yes; unclear; unclear; yes; yes; yes; yes; yes	8/10	Q4 unclear: consecutive inclusion not stated; Q5 unclear: complete inclusion of all eligible patients not stated
Imai et al. 2023. The British journal of oral & maxillofacial surgery. PMID 37863724	clinical_series	jbi_case_series	unclear; yes; unclear; unclear; unclear; unclear; unclear; yes; unclear; unclear	2/10	abstract only; Q1 eligibility criteria absent; Q2 accuracy measured in three axes using set landmarks; Q3 condition-identification method absent; Q4 consecutive inclusion not stated; Q5 complete inclusion not stated; Q6
Sun et al. 2023. BMC oral health. PMID 37940900	clinical_series	jbi_case_series	yes; yes; yes; unclear; unclear; yes; yes; yes; yes; no	7/10	Q4-Q5 unclear: consecutive and complete inclusion were not established; Q10 no: an unspecified Student's t-test was used for paired preoperative and postoperative measurements
Zhao et al. 2023. Journal of clinical medicine. PMID 38137607	clinical_series	jbi_case_series	yes; yes; yes; unclear; unclear; no; yes; yes; yes; yes	7/10	Q4 unclear: consecutive inclusion not stated; Q5 unclear: complete inclusion not stated; Q6 no: individual ages and other participant demographics were not reported
Li et al. 2024. Orthopaedic surgery. PMID 38296795	clinical_series	jbi_case_series	yes; yes; yes; unclear; unclear; yes; yes; yes; no; yes	7/10	Q4 unclear: consecutive inclusion not stated; Q5 unclear: complete inclusion of all eligible patients not stated; Q9 no: site or clinic demographics not described
Zhang et al. 2024. Frontiers in bioengineering and	clinical_serie	jbi_case	unclear; yes; yes; unclear;	4/1	Q1 unclear: explicit eligibility criteria absent; Q4-Q5 unclear:

Study	Tier	Tool	Judgements	Overall	Support
biotechnology. PMID 38333079	s	_series	unclear; yes; yes; no; no; no	0	consecutive and complete inclusion not stated; Q8 no: follow-up duration and cohort size conflict across sections; Q9 no: site demographics absent; Q10 no:
Khalaf et al. 2024. Journal of cranio-maxillo-facial surgery : official publication of the European Association for Cranio-Maxillo-Facial Surgery. PMID 38368205	clinical_series	jbi_case_series	unclear; unclear; unclear; unclear; unclear; no; no; yes; no; unclear	1/10	abstract only; Q1-Q5 unclear: eligibility, diagnostic methods, and completeness not reported; Q6-Q7 no: participant demographics and clinical information not clearly reported; Q9 no: site demographics absent; Q10 unclear
Yan et al. 2024. Aesthetic plastic surgery. PMID 38409347	clinical_series	jbi_case_series	unclear; unclear; unclear; unclear; unclear; no; yes; yes; unclear; unclear	2/10	abstract only; Q1: no inclusion criteria; Q2: no standardized condition measure described; Q3: identification method unstated; Q4: consecutiveness unstated; Q5: completeness unstated; Q6: age alone incompletely described
Iratwar et al. 2024. Journal of biomaterials applications. PMID 38423069	clinical_series	jbi_case_series	unclear; unclear; unclear; unclear; unclear; unclear; unclear; yes; unclear; unclear	1/10	abstract only; Q1–Q7 eligibility, identification, consecutive or complete inclusion, demographics, and clinical details were not reported; Q9 participating sites were unnamed; Q10 analysis methods were not reported
Li et al. 2024. Journal of orthopaedic surgery and research. PMID 38561755	clinical_series	jbi_case_series	yes; yes; yes; unclear; unclear; yes; yes; yes; no; yes	7/10	Q4 unclear: consecutive inclusion not stated; Q5 unclear: complete inclusion of all eligible patients not stated; Q9 no: site or clinic demographics not reported
Hu et al. 2024. BMC musculoskeletal disorders. PMID 38755628	clinical_series	jbi_case_series	yes; yes; yes; yes; no; yes; yes; yes; yes; no	8/10	Consecutive screening, diagnosis, clinical characteristics, site, and outcomes are detailed; four patients lost to follow-up were excluded; independent-samples tests were specified for pre-post outcomes
Chen et al. 2024. BMC musculoskeletal disorders. PMID 38880911	clinical_series	jbi_case_series	yes; yes; yes; yes; yes; yes; yes; yes; unclear; yes	9/10	Eleven consecutive patients met explicit criteria with detailed clinical and follow-up reporting; institutional demographics are not described
Amin et al. 2024. Surgical neurology international. PMID 39108397	clinical_series	jbi_case_series	yes; yes; yes; no; unclear; yes; yes; yes; yes; yes	8/10	Q4 data were collected purposively, not consecutively; Q5 inclusion of all eligible cases was not reported
Geroski et al. 2024. Medical engineering & physics. PMID 39160013	clinical_series	jbi_case_series	unclear; unclear; unclear; unclear; unclear; unclear; unclear; yes; unclear; unclear	1/10	abstract only; surgical fit is reported for all five patients; eligibility, diagnosis, sampling, demographics, site, follow-up, and analysis details are absent
Moris et al. 2025. Journal of stomatology, oral and maxillofacial surgery. PMID 39182846	clinical_series	jbi_case_series	yes; unclear; unclear; unclear; unclear; unclear; unclear; yes; yes; unclear	3/10	abstract only; eligibility dates and setting are stated and outcomes are reported; diagnostic, clinical, inclusion-completeness, demographic, and analysis details are absent
Liu et al. 2025. Orthopaedic	clinical_serie	jbi_case	yes; yes; yes; unclear; unclear;	7/1	Inclusion and diagnostic methods, patient characteristics, site, and

Study	Tier	Tool	Judgements	Overall	Support
surgery. PMID 39429061	s	_series	yes; yes; yes; yes; no	0	outcomes are reported; consecutive and complete inclusion are unclear; ANOVA was used for paired preoperative and follow-up scores
Caruso et al. 2024. Craniomaxillofacial trauma & reconstruction. PMID 39553819	clinical_series	jbi_case_series	yes; unclear; unclear; unclear; unclear; unclear; unclear; yes; unclear; unclear	2/10	abstract only; Luhr class III and treatment dates define inclusion; outcomes are reported; other checklist details are not reported
Trott et al. 2025. Head & neck. PMID 39731307	clinical_series	jbi_case_series	yes; unclear; unclear; unclear; unclear; yes; yes; yes; unclear; unclear	4/10	abstract only; Q1: reconstruction type and study dates defined eligibility; Q2: no standardized defect measurement described; Q3: condition-identification methods unstated; Q4: consecutiveness unstated; Q5: completeness
Li et al. 2024. Biomaterials translational. PMID 39734704	clinical_series	jbi_case_series	yes; yes; yes; unclear; yes; yes; yes; yes; yes; yes	9/10	Q4 consecutive enrolment was not stated
Saloniemi et al. 2025. Oral and maxillofacial surgery. PMID 39853440	clinical_series	jbi_case_series	yes; yes; yes; yes; yes; yes; yes; yes; yes; yes	10/10	Q1 all-case criteria were explicit; Q2-Q3 fractures were assessed by CT; Q4-Q5 all eligible PSI cases in the period were included; Q6-Q7 demographics and clinical details were tabulated; Q8 follow-up outcomes were report
Rios et al. 2025. Journal of cranio-maxillo-facial surgery : official publication of the European Association for Cranio-Maxillo-Facial Surgery. PMID 39875225	clinical_series	jbi_case_series	unclear; unclear; unclear; unclear; unclear; unclear; yes; yes; unclear; unclear	2/10	abstract only; Q1 criteria were absent; Q2 standard measurement was unstated; Q3 identification methods were unstated; Q4 consecutive inclusion was unstated; Q5 complete inclusion was unstated; Q6 patient demographics we
Yang et al. 2025. Scientific reports. PMID 39900958	clinical_series	jbi_case_series	yes; yes; yes; unclear; unclear; yes; yes; yes; yes; yes	8/10	Q4 consecutive enrolment was not stated; Q5 numbers screened and excluded were not reported
Sun et al. 2025. International orthopaedics. PMID 39921749	clinical_series	jbi_case_series	yes; yes; unclear; unclear; unclear; unclear; yes; yes; unclear; yes	5/10	abstract only; Q3 identification methods were unstated; Q4 consecutive inclusion was unstated; Q5 complete inclusion was unstated; Q6 patient demographics were absent; Q9 the site was described only as “our hospital”
Vaziri et al. 2025. European journal of orthopaedic surgery & traumatology : orthopedie traumatologie. PMID 40047935	clinical_series	jbi_case_series	unclear; unclear; unclear; unclear; unclear; unclear; yes; yes; unclear; unclear	2/10	abstract only; Q1 criteria were absent; Q2 standard measurement was unstated; Q3 identification methods were unstated; Q4 consecutive inclusion was unstated; Q5 complete inclusion was unstated; Q6 patient demographics we
Benady et al. 2025. Medicina (Kaunas, Lithuania). PMID 40142287	clinical_series	jbi_case_series	yes; unclear; unclear; unclear; unclear; yes; yes; yes; yes; yes	6/10	Q2 standard and reliable condition measurement was not described; Q3 diagnostic methods were not described; Q4 consecutive inclusion was not stated; Q5 the screened and eligible population was not reported
Rakityansky et al. 2025. Zhurnal voprosy neirokhirurgii imeni N.	clinical_serie	jbi_case	unclear; unclear; unclear; unclear; unclear; unclear; yes; no; yes;	2/1	abstract only; Q1 criteria were absent; Q2 standard measurement

Study	Tier	Tool	Judgements	Overall	Support
N. Burdenko. PMID 40183614	s	_series	unclear	0	was unstated; Q3 diagnostic methods were unstated; Q4 consecutive inclusion was unstated; Q5 complete inclusion was unstated; Q6 patient demographics were a
Sarigul Sezenoz et al. 2025. European journal of ophthalmology. PMID 40232250	clinical_series	jbi_case_series	unclear; unclear; unclear; unclear; unclear; unclear; yes; yes; yes; yes	4/10	abstract only; Q1 criteria were absent; Q2 standard measurement was unstated; Q3 identification methods were unstated; Q4 consecutive inclusion was unstated; Q5 complete inclusion was unstated; Q6 patient demographics we
Li et al. 2025. Orthopaedic surgery. PMID 40254773	clinical_series	jbi_case_series	yes; yes; yes; unclear; yes; yes; yes; yes; unclear; yes	8/10	Q4 consecutive enrolment was not stated; Q9 the site was described only as “our hospital”
Hu et al. 2025. Orthopaedic surgery. PMID 40310728	clinical_series	jbi_case_series	yes; yes; yes; unclear; no; yes; yes; yes; unclear; no	6/10	Q1 explicit criteria stated; Q2-Q3 pathology, staging and imaging used; Q4 consecutive inclusion unreported; Q5 patients with incomplete follow-up were excluded; Q6-Q7 demographics and clinical details reported; Q8 syste
Łoginoff et al. 2025. Journal of cranio-maxillo-facial surgery : official publication of the European Association for Cranio-Maxillo-Facial Surgery. PMID 40399182	clinical_series	jbi_case_series	yes; yes; yes; unclear; unclear; no; yes; yes; unclear; yes	6/10	abstract only; Q1 severe atrophy and treatment eligibility stated; Q2-Q3 Cawood-Howell classification and CT used; Q4-Q5 consecutive and complete inclusion unreported; Q6 demographics absent; Q7 treatment details reporte
Gupta et al. 2025. Journal of hand and microsurgery. PMID 40503244	clinical_series	jbi_case_series	yes; yes; yes; unclear; unclear; yes; yes; yes; unclear; yes	7/10	Q1 malunion, restricted motion and pain criteria stated; Q2-Q3 radiographs and CT used; Q4-Q5 consecutive and complete inclusion unreported; Q6-Q7 patient and clinical details tabulated; Q8 radiographic and one-year func
Kang et al. 2025. Scientific reports. PMID 40702034	clinical_series	jbi_case_series	yes; yes; yes; unclear; no; yes; yes; yes; unclear; yes	7/10	Q1 detailed criteria stated; Q2-Q3 Ficat classification and imaging used; Q4 consecutive recruitment unreported; Q5 interim outcomes include only three of eight approved patients; Q6-Q7 demographics and clinical characte
Jarayabhand et al. 2025. Journal of orthopaedic surgery (Hong Kong). PMID 40726307	clinical_series	jbi_case_series	unclear; unclear; unclear; unclear; unclear; no; yes; yes; unclear; no	2/10	abstract only; Q1-Q5 eligibility, diagnostic methods and completeness unreported; Q6 demographics absent; Q7 indications and anatomical sites reported; Q8 union, complications and 6-24-month follow-up reported; Q9 site c
Shi et al. 2025. 3D printing in medicine. PMID 40736597	clinical_series	jbi_case_series	yes; yes; yes; yes; yes; yes; yes; yes; unclear; no	8/10	Q1 eligible tumor and procedure criteria stated; Q2-Q3 imaging and pathology used; Q4-Q5 nine consecutive cases included; Q6-Q7 demographics and clinical details tabulated; Q8 scheduled

Study	Tier	Tool	Judgements	Overall	Support
					follow-up and outcomes reported; Q
Bao et al. 2025. The Journal of craniofacial surgery. PMID 40845376	clinical_series	jbi_case_series	unclear; unclear; unclear; unclear; unclear; no; unclear; yes; unclear; yes	2/10	abstract only; Q1-Q5 recruitment and diagnostic methods unreported; Q6 age and other demographics absent; Q7 clinical histories not reported; Q8 complications and satisfaction reported; Q9 site characteristics absent; Q1
Moncayo-Matute et al. 2025. Journal of personalized medicine. PMID 41003100	clinical_series	jbi_case_series	no; unclear; yes; yes; unclear; yes; yes; yes; yes; yes	7/10	Q1 defined criteria absent; Q2 standardized measurement across heterogeneous conditions unclear; Q3 CT and clinical examinations identified conditions; Q4 seven cases called consecutive; Q5 completeness unclear because c
Tehli et al. 2026. Operative neurosurgery (Hagerstown, Md.). PMID 41090924	clinical_series	jbi_case_series	unclear; yes; yes; unclear; unclear; yes; yes; yes; unclear; yes	6/10	abstract only; Q1 criteria unreported; Q2-Q3 histopathology confirmed all diagnoses; Q4-Q5 consecutive and complete inclusion unreported; Q6 age and sex reported; Q7 condition and intervention described; Q8 outcomes and
Ngo et al. 2026. European spine journal : official publication of the European Spine Society, the European Spinal Deformity Society, and the European Section of the Cervical Spine Research Society. PMID 41094110	clinical_series	jbi_case_series	yes; yes; unclear; unclear; unclear; no; yes; yes; unclear; unclear	4/10	abstract only; identification method, consecutive and complete sampling were not reported; demographics and site characteristics were absent; statistical tests were not identified
Wang et al. 2025. Frontiers in bioengineering and biotechnology. PMID 41194981	clinical_series	jbi_case_series	yes; yes; yes; unclear; unclear; yes; yes; yes; yes; no	7/10	consecutive and complete inclusion were not stated; clinical significance claims lacked a described statistical analysis
Guo et al. 2025. Frontiers in bioengineering and biotechnology. PMID 41383719	clinical_series	jbi_case_series	yes; yes; yes; yes; yes; yes; yes; yes; unclear; yes	9/10	the institutional database yielded all consecutive eligible patients with complete follow-up, but presenting-site demographics were not reported
Liokatis et al. 2025. Maxillofacial plastic and reconstructive surgery. PMID 41385022	clinical_series	jbi_case_series	yes; yes; yes; unclear; unclear; yes; yes; yes; yes; no	7/10	patients were not stated to be consecutively or completely included; Friedman’s test was used for the high-versus-low osteotomy comparison
Tang et al. 2025. Zhongguo xiu fu chong jian wai ke za zhi = Zhongguo xiufu chongjian waike zazhi = Chinese journal of reparative and reconstructive surgery. PMID 41397797	clinical_series	jbi_case_series	unclear; yes; yes; unclear; unclear; yes; yes; yes; unclear; yes	6/10	abstract only; explicit inclusion criteria, consecutive and complete sampling, and site characteristics were not reported
Bayram et al. 2026. The Journal of craniofacial surgery. PMID 41499545	clinical_series	jbi_case_series	unclear; yes; yes; unclear; unclear; no; yes; yes; unclear; unclear	4/10	abstract only; inclusion, consecutive and complete sampling not reported; demographics omit age; site characteristics and statistical methods unclear
Chintalapudi et al. 2026. The Journal of craniofacial surgery.	clinical_series	jbi_case_series	unclear; yes; unclear; unclear; unclear; no; yes; yes; unclear;	3/10	abstract only; inclusion, diagnostic method, consecutive and complete sampling not

Study	Tier	Tool	Judgements	Ov eral l	Support
PMID 41524259			unclear		reported; demographics limited to sample size; site characteristics and statistical methods unclear
Wüster et al. 2026. Oral and maxillofacial surgery. PMID 41634287	clinical_series	jbi_case_series	yes; unclear; unclear; unclear; unclear; yes; yes; yes; yes; yes	6/10	Diagnostic ascertainment methods were not described; consecutive or complete inclusion was not reported
Thanh et al. 2026. Bioengineering (Basel, Switzerland). PMID 41749792	clinical_series	jbi_case_series	yes; yes; yes; unclear; unclear; yes; yes; yes; unclear; yes	7/10	Consecutive or complete inclusion was not reported; clinic demographics were not described
Do et al. 2026. World journal of surgical oncology. PMID 41795068	clinical_series	jbi_case_series	yes; unclear; unclear; yes; yes; yes; yes; yes; yes; yes	8/10	Methods used to establish the underlying conditions were not described
Ghorab et al. 2026. Oral and maxillofacial surgery. PMID 41840235	clinical_series	jbi_case_series	unclear; unclear; unclear; unclear; unclear; unclear; unclear; yes; unclear; unclear	1/10	abstract only; inclusion and diagnostic methods, consecutive or complete recruitment, demographics, clinical details, site characteristics, and statistical methods were not reported
Liu et al. 2026. BMC musculoskeletal disorders. PMID 41943026	clinical_series	jbi_case_series	yes; unclear; unclear; unclear; unclear; yes; yes; yes; yes; no	5/10	Osteomyelitis diagnostic criteria were not described; consecutive and complete inclusion were not reported; ANOVA was used for paired preoperative-postoperative scores among eight surviving patients
Benady et al. 2026. Bone & joint open. PMID 41956475	clinical_series	jbi_case_series	yes; unclear; unclear; unclear; unclear; yes; yes; yes; yes; yes	6/10	Diagnostic ascertainment methods were not described; consecutive or complete inclusion was not reported
Kim et al. 2026. Journal of clinical medicine. PMID 42123027	clinical_series	jbi_case_series	yes; unclear; unclear; yes; unclear; yes; yes; yes; unclear; no	5/10	Condition-identification standards were not described; completeness of eligible-patient inclusion was not reported; clinic demographics were absent; Pearson correlation does not assess agreement
Catarzi et al. 2026. Medicina (Kaunas, Lithuania). PMID 42356056	clinical_series	jbi_case_series	yes; yes; yes; unclear; unclear; yes; yes; yes; unclear; yes	7/10	full text reported explicit eligibility, CT-based fracture identification, demographics, clinical details, follow-up outcomes, and appropriate analyses; consecutive or complete inclusion and named site information were n
Abu-Sirhan et al. 2026. Journal of cranio-maxillo-facial surgery : official publication of the European Association for Cranio-Maxillo-Facial Surgery. PMID 42413458	clinical_series	jbi_case_series	unclear; unclear; unclear; unclear; unclear; yes; unclear; yes; unclear; unclear	2/10	abstract only; age, sex, OSAS prevalence, accuracy, and complications were reported, but eligibility, identification, recruitment, site, clinical-detail, and statistical-method information was absent
Elter et al. 2026. Journal of cranio-maxillo-facial surgery : official publication of the European Association for Cranio-Maxillo-Facial Surgery. PMID 42418929	clinical_series	jbi_case_series	unclear; unclear; unclear; unclear; unclear; unclear; unclear; yes; yes; yes	3/10	abstract only; outcomes, the Charité site, and descriptive analyses were reported, but eligibility, case identification, recruitment completeness, and participant details were not
Austin et al. 2026. International journal of oral and maxillofacial surgery. PMID 42448534	clinical_series	jbi_case_series	yes; unclear; unclear; yes; unclear; unclear; unclear; yes; unclear; yes	4/10	abstract only; adult eligibility, consecutive inclusion, outcomes, and paired analysis were reported, but diagnostic methods, completeness, demographics,

Study	Tier	Tool	Judgements	Overall	Support
					clinical details, and site were not
Abdeldayem et al. 2026. Maxillofacial plastic and reconstructive surgery. PMID 42479267	clinical_series	jbi_case_series	yes; unclear; unclear; unclear; unclear; yes; yes; yes; yes; yes	6/10	full text reported eligibility, demographics, clinical characteristics, site, 12-month outcomes, and appropriate analyses; no information established standardized lesion identification or consecutive complete inclusion
Riad et al. 2026. BMC oral health. PMID 42482012	clinical_series	jbi_case_series	yes; yes; yes; yes; yes; yes; yes; yes; yes; yes	10/10	full text reported explicit eligibility, CT-based identification, six consecutive complete cases, participant and site details, complete follow-up, and appropriate descriptive analyses
Smeets et al. 2026. International journal of oral and maxillofacial surgery. PMID 42674940	clinical_series	jbi_case_series	yes; unclear; unclear; unclear; unclear; unclear; unclear; yes; unclear; yes	3/10	abstract only; eligibility, outcomes, and reproducibility analysis were reported, but recruitment, demographics, clinical details, and site were not
Alshikh et al. 2026. . doi:10.21203/rs.3.rs-8841053/v1	rct	rob2	some_concerns; high; low; low; some_concerns	high	D2: groups received different autogenous:xenograft ratios (60:40 versus 70:30), explicitly acknowledged as potentially influencing volumetric outcomes
Shuang et al. 2016. Medicine. PMID 26817880	rct	rob2	some_concerns; low; low; low; some_concerns	some_concerns	“randomly divided” without sequence generation or allocation concealment; no protocol or prespecified analysis plan reported
Raisian et al. 2017. Reviews on recent clinical trials. PMID 28828975	rct	rob2	some_concerns; some_concerns; low; some_concerns; some_concerns	some_concerns	abstract only; random-sequence generation and concealment were not reported; deviations and analysis were not described; assessor blinding was not reported; no protocol or prespecified analysis was reported
Maini et al. 2018. Archives of orthopaedic and trauma surgery. PMID 29368178	rct	rob2	some_concerns; some_concerns; some_concerns; some_concerns; some_concerns	some_concerns	abstract only; computerized randomization stated but no allocation concealment or baseline balance reported; no information on deviations or missing data; X-ray and CT outcomes reported without assessor blinding; no prot
Schneider et al. 2019. Clinical oral investigations. PMID 30443778	rct	rob2	some_concerns; some_concerns; some_concerns; some_concerns; some_concerns	some_concerns	abstract only; D1 randomized designation lacked sequence and concealment details; D2 no information on deviations or analysis by assignment; D3 no information on losses from randomization to analysis; D4 no information o
Li et al. 2021. Plastic and reconstructive surgery. PMID 34705785	rct	rob2	low; some_concerns; some_concerns; low; some_concerns	some_concerns	six randomized participants were excluded despite an intention-to-treat claim; outcomes were available for 58 of 64 participants with unequal final group sizes; no protocol or prespecified reporting plan reported

Study	Tier	Tool	Judgements	Overall	Support
Sánchez-Jáuregui et al. 2022. Journal of cranio-maxillo-facial surgery : official publication of the European Association for Cranio-Maxillo-Facial Surgery. PMID 35760659	rct	rob2	some_concerns; some_concerns; some_concerns; some_concerns; some_concerns	some_concerns	abstract only; no information on sequence generation or concealment; no information on deviations; no information on outcome completeness; no information on assessor blinding; no information on prespecified reporting
Van der Wel et al. 2023. International journal of oral and maxillofacial surgery. PMID 36202719	rct	rob2	some_concerns; some_concerns; high; some_concerns; some_concerns	high	abstract only; only 27 of 58 protocol completers provided the 1-year outcome
Bevini et al. 2025. Journal of cranio-maxillo-facial surgery : official publication of the European Association for Cranio-Maxillo-Facial Surgery. PMID 39933970	rct	rob2	some_concerns; some_concerns; some_concerns; low; some_concerns	some_concerns	abstract only; D1 “blind randomization” lacked sequence and concealment details; D2 no information on deviations; D3 no analyzed-group counts or attrition information; D5 no prespecified protocol or analysis plan was rep
Sheta et al. 2025. BMC oral health. PMID 41176602	rct	rob2	some_concerns; some_concerns; low; some_concerns; some_concerns	some_concerns	sealed envelopes were reported without sequence-generation or concealment details; intervention deviations and analysis population were not reported; only the data analyst was blinded; the trial was retrospectively regis
El Morsy et al. 2026. The Journal of craniofacial surgery. PMID 42183622	rct	rob2	some_concerns; some_concerns; some_concerns; some_concerns; some_concerns	some_concerns	abstract only; no information on sequence generation or concealment, deviations, attrition, assessor blinding, or prespecified outcome analyses
Abdellatif et al. 2026. BMC oral health. PMID 42498926	rct	rob2	low; low; low; low; some_concerns	some_concerns	no information establishing that the reported analyses were prespecified before outcome data were available

Table S5. Funding, conflict of interest and industry authorship (full-text records; amendment A7)

Item	Category	Studies
Funding	industry	4
Funding	mixed	5
Funding	none stated	19
Funding	public	34
Funding	unclear	233
Funding	unclear abstract only	344
Conflict of interest	declared	9
Conflict of interest	none declared	57
Conflict of interest	unclear	229
Conflict of interest	unclear abstract only	344
Industry author	no	45
Industry author	unclear	589
Industry author	yes	5

Table S6. Outcome-by-study disposition for the meta-analysis test (one line per study and outcome; measures not pooled are named)

Study	Outcome	Measure used	Alternative measures reported	Comparator class (charted)	Pooled
pmid:42498926	accuracy	bony landmark deviation relative to Frankfort horizontal plane (mm)	bony landmark deviation relative to midsagittal plane (mm); bony landmark deviation relative to coronal plane (mm); overall bony landmark deviation across all planes (mm); dental landmark deviation relative to Frankfort horizontal p; dental landmark deviation relative to midsagittal plane (mm); dental landmark deviation relative to coronal plane (mm); overall dental landmark deviation across all planes (mm)	conventional_implant	yes
pmid:42074623	revision_or_removal	secondary surgical revision for implant malposition	.	conventional_implant	yes
pmid:41657642	infection	postoperative infection	.	conventional_implant	yes
pmid:41361902	operative_time	operative duration (minutes)	.	conventional_implant	yes
pmid:41361902	blood_loss	intraoperative blood loss (mL)	.	conventional_implant	no
pmid:41361902	revision_or_removal	revision following rod breakage	.	conventional_implant	yes
pmid:41039504	function	best-corrected visual acuity, logMAR	.	conventional_implant	no
pmid:40826038	operative_time	operative duration (minutes)	.	manual_design	no
pmid:40826038	complications_any	patients with any postoperative complication	.	manual_design	no
pmid:40826038	infection	postoperative infection	.	manual_design	no
pmid:39819879	operative_time	total operation time (minutes)	.	conventional_implant	yes
pmid:39819879	accuracy	mediolateral displacement of affected condyle (mm)	coronal roll deviation of condylar axis (degrees); transverse yaw deviation of condylar axis (degrees); sagittal pitch deviation of condylar axis (degrees); intercondylar distance deviation (mm); intercondylar angle deviation (degrees); angular deviation of reconstructed bone segments (degrees)	conventional_implant	yes
pmid:38793041	complications_any	patients with any acute or delayed implant-related complication	.	conventional_implant	yes
pmid:38793041	revision_or_removal	reoperation to reposition a displaced implant	.	conventional_implant	yes
pmid:38793041	infection	wound-site infection	.	conventional_implant	yes
pmid:3865	operative_tim	operation duration (minutes)	.	conventional_implant	yes

Study	Outcome	Measure used	Alternative measures reported	Comparator class (charted)	Pooled
4343	e				
pmid:38654343	blood_loss	intraoperative blood loss (mL)	.	conventional_implant	no
pmid:38654343	complications_any	patients with any postoperative complication	.	conventional_implant	yes
pmid:38654343	revision_or_removal	prosthesis revision for loosening or screw failure	.	conventional_implant	yes
pmid:38654343	function	Musculoskeletal Tumor Society score (MSTS-93)	Harris Hip Score (HHS)	conventional_implant	no
pmid:37762977	accuracy	anteroposterior deviation from planned position (mm)	craniocaudal deviation from planned position (mm); pitch deviation from planned position (degrees)	conventional_implant	yes
pmid:37241016	revision_or_removal	secondary operation for implant malposition	.	conventional_implant	yes
pmid:36142107	accuracy	RMSE 3D error between planned and postoperative maxillary position (mm)	.	conventional_implant	yes
pmid:35434332	accuracy	Median 3D distance between planned and postoperative bone-segment position (mm)	.	alternative_method	no
pmid:35434332	revision_or_removal	Plate removal	.	alternative_method	no
pmid:35264178	function	Musculoskeletal Tumor Society (MSTS) score	.	conventional_implant	no
pmid:35059310	operative_time	Operating time (min)	.	conventional_implant	yes
pmid:35059310	blood_loss	Intraoperative bleeding (mL)	.	conventional_implant	no
pmid:35059310	complications_any	Patients with a postoperative complication	.	conventional_implant	yes
pmid:35059310	function	MSTS-93 score	.	conventional_implant	no
pmid:34900674	operative_time	Operating time (min)	.	conventional_implant	yes
pmid:34900674	complications_any	Patients with documented postoperative complications	.	conventional_implant	yes
pmid:34900674	accuracy	D1 change in bilateral lateralmost condylar-point distance (mm)	D2 change in bilateral medialmost condylar-point distance (m; D3 change in bilateral incisura mandibulae distance (mm); D5 change in bilateral processus coronoideus distance (mm); D6 change in bilateral gonion distance (mm)	conventional_implant	yes
pmid:34441806	operative_time	Duration from incision to suture (min)	.	conventional_implant	yes
pmid:34441806	complications_any	Patients with postoperative ectropion adverse event	.	conventional_implant	yes
pmid:34208555	operative_time	Operating time (min)	.	conventional_implant	yes
pmid:33128609	operative_time	Operation time (h:mm:ss; SD in parentheses)	.	conventional_implant	yes
pmid:33128609	complications_any	Patients with any complication	.	conventional_implant	yes
pmid:33128609	revision_or_removal	Plate removal or revision	.	conventional_implant	yes
pmid:3312	infection	Implant-site infection	.	conventional_implant	yes

Study	Outcome	Measure used	Alternative measures reported	Comparator class (charted)	Pooled
8609					
pmid:32855907	operative_time	Procedure duration for isolated malar-bone fracture (h)	Procedure duration for isolated orbital-wall fracture (h); Procedure duration for combined malar, orbital-wall, and oth	conventional_implant	no
pmid:32455967	operative_time	Surgery time from skin incision to skin closure (min)	.	conventional_implant	yes
doi:10.21203/rs.3.rs-8841053/v1	infection	Donor-site infection	.	conventional_implant	yes
pmid:32572853	accuracy	Absolute distance deviation of reconstructed maxilla or mandible (mm)	Distance deviation between preoperative and postoperative bi; Distance deviation of transplanted bone-graft segments (mm); Distance deviation of maxillary or mandibular resection oste	conventional_implant	yes
pmid:32572853	blood_loss	Estimated intraoperative blood loss (mL)	.	conventional_implant	no
pmid:32572853	operative_time	Total operative time (min)	.	conventional_implant	no
pmid:32572853	revision_or_removal	Plate removal	.	conventional_implant	yes
pmid:32572853	infection	Wound infection	.	conventional_implant	yes
pmid:37138237	accuracy	Mean linear distance discrepancy across 11 anatomical reference points (mm)	.	conventional_implant	yes
pmid:37138237	operative_time	Operation time from entering to leaving the operating room (h)	.	conventional_implant	yes
pmid:37138237	complications_any	Patients with a significant postoperative complication	.	conventional_implant	yes
pmid:37138237	revision_or_removal	Plate revision or replacement	.	conventional_implant	yes
pmid:26817880	operative_time	Operative time (min)	.	conventional_implant	yes
pmid:26817880	complications_any	Patients with any complication	.	conventional_implant	yes
pmid:26817880	infection	Wound infection	.	conventional_implant	yes
pmid:34705785	accuracy	Centroid position discrepancy of the maxilla (mm)	Mediolateral translation discrepancy of the maxilla (mm); Anteroposterior translation discrepancy of the maxilla (mm); Superoinferior translation discrepancy of the maxilla (mm); Pitch orientation discrepancy of the maxilla (degrees); Roll orientation discrepancy of the maxilla (degrees); Yaw orientation discrepancy of the maxilla (degrees)	alternative_method	no
pmid:34705785	blood_loss	Intraoperative blood loss (mL)	.	alternative_method	no
pmid:34705785	infection	Infection	.	alternative_method	no

Table S7. Artificial intelligence studies: evaluation data, leakage risk and prediction-model reporting (two independent coders; `contested` means the coders differed, which for these items generally means the report does not say)

Item	Value	All AI studies (n=36)	Cranial studies (n=17)
Evaluation dataset named	autoimplant	2	2
Evaluation dataset named	skullbreak	4	4
Evaluation dataset named	skullfix	6	6
Evaluation dataset named	public other	9	5
Evaluation dataset named	private	16	5
Evaluation dataset named	synthetic	14	7
Evaluation dataset named	unclear	8	5
Risk that evaluation data were not independent	unclear	17	11
Risk that evaluation data were not independent	contested	11	3
Risk that evaluation data were not independent	moderate	4	1
Risk that evaluation data were not independent	high	2	2
Risk that evaluation data were not independent	low	2	0
Calibration reported	na	26	15
Calibration reported	contested	4	2
Calibration reported	not reported	4	0
Calibration reported	reported	2	0
External validation	unclear	12	7
External validation	no	11	4
External validation	contested	9	5
External validation	yes	4	1
Missing-data handling reported	not reported	27	15
Missing-data handling reported	reported	6	1
Missing-data handling reported	contested	3	1
Sample size justified	contested	14	10
Sample size justified	not justified	12	2
Sample size justified	unclear	8	4
Sample size justified	justified	2	1
Used in the care pathway of a real patient	no	32	15
Used in the care pathway of a real patient	yes	2	1
Used in the care pathway of a real patient	contested	1	0
Used in the care pathway of a real patient	unclear	1	1
